# Verdiperstat in Amyotrophic Lateral Sclerosis: Results From the Randomized HEALEY ALS Platform Trial

**DOI:** 10.1001/jamaneurol.2024.5249

**Published:** 2025-02-17

**Authors:** Writing Committee for the HEALEY ALS Platform Trial, Jinsy Andrews, Sabrina Paganoni, Eric A. Macklin, Lori B. Chibnik, Melanie Quintana, Benjamin R. Saville, Michelle A. Detry, Matteo Vestrucci, Joseph Marion, Anna McGlothlin, Eufrosina Young, Marianne Chase, Lindsay Pothier, Brittney Harkey, Hong Yu, Alex Sherman, Jeremy Shefner, Meghan Hall, Gale Kittle, Mariah R. Connolly, James D. Berry, Derek D’Agostino, Eric Tustison, Elisa Giacomelli, Erica Scirocco, Gustavo Alameda, Eduardo Locatelli, Doreen Ho, Adam Quick, Daragh Heitzman, Senda Ajroud-Driss, Stanley H. Appel, Sheetal Shroff, Jonathan Katz, Kevin Felice, Nicholas J. Maragakis, Zachary Simmons, Stephen A. Goutman, Nicholas Olney, Timothy Miller, Joseph Americo Fernandes, Hristelina Ilieva, Omar Jawdat, Michael D Weiss, Laura Foster, Tuan Vu, Shafeeq Ladha, Margaret Ayo Owegi, Daniel S. Newman, Ximena Arcila-Londono, Carlayne E. Jackson, Andrea Swenson, Terry Heiman-Patterson, James Caress, Dominic Fee, Amanda Peltier, Richard Lewis, Jeffrey Rosenfeld, David Walk, Kristin Johnson, Matthew Elliott, Edward J. Kasarskis, Seward Rutkove, Courtney E. McIlduff, Richard Bedlack, Lauren Elman, Namita A. Goyal, Kourosh Rezania, Paul Twydell, Michael Benatar, Jonathan Glass, Jeffrey A. Cohen, Vovanti Jones, Lindsay Zilliox, James P. Wymer, Said R. Beydoun, Jaimin Shah, Gary L. Pattee, Jennifer Martinez-Thompson, Shakti Nayar, Volkan Granit, Mary Donohue, Katheryn Grossman, Daniel J Campbell, Irfan A Qureshi, Merit E. Cudkowicz, Suma Babu

**Affiliations:** 1 Columbia University, New York, New York; 2 Sean M. Healey and AMG Center for ALS & the Neurological Clinical Research Institute, Massachusetts General Hospital, Harvard Medical School, Boston, Massachusetts; 3 Spaulding Rehabilitation Hospital, Harvard Medical School; 4 Harvard T.H. Chan School of Public Health, Department of Epidemiology, Boston, Massachusetts; 5 Berry Consultants, Austin, Texas; 6 SUNY Upstate, Syracuse, New York; 7 Barrow Neurological Institute, Phoenix, Arizona; 8 Holy Cross Hospital, Fort Lauderdale, Florida; 9 Nova Southeastern University, Davie, Florida; 10 Ohio State University, Columbus; 11 Texas Neurology, Dallas; 12 Northwestern University, Chicago, Illinois; 13 Methodist Neurological Institute, Houston, Texas; 14 California Pacific Medical Center, San Francisco; 15 Hospital for Special Care, New Britain, Connecticut; 16 Johns Hopkins University, Baltimore, Maryland; 17 Penn State Milton S. Hershey Medical Center, Hershey, Pennsylvania; 18 University of Michigan, Ann Arbor; 19 Providence ALS Clinic, Portland, Oregon; 20 Washington University, St Louis, Missouri; 21 University of Nebraska Medical Center, Omaha, Nebraska; 22 Jefferson Weinberg ALS Center, Philadelphia, Pennsylvania; 23 University of Kansas Medical Center, Kansas City; 24 University of Washington, Seattle; 25 University of Colorado Anschutz, Aurora; 26 University of South Florida, College of Medicine, Tampa; 27 University of Massachusetts, Worcester, Worcester; 28 Henry Ford Hospital, Detroit, Michigan; 29 University of Texas Health Science Center, Houston; 30 University of Iowa, Iowa City; 31 Temple University, Philadelphia, Pennsylvania; 32 Wake Forest University School of Medicine, Winston-Salem, North Carolina; 33 Medical College of Wisconsin, Milwaukee; 34 Vanderbilt University Medical Center, Nashville, Tennessee; 35 Cedars-Sinai Medical Center, New York, New York; 36 Loma Linda University School of Medicine, Loma Linda, California; 37 University of Minnesota/Twin Cities ALS Research Consortium, Minneapolis; 38 Ochsner Health System, Lafayette, Louisiana; 39 University of Virginia, Charlottesville; 40 University of Kentucky, Lexington; 41 Beth Israel Deaconess Medical Center, Boston, Massachusetts; 42 Duke University, Durham, North Carolina; 43 University of Pennsylvania, Philadelphia; 44 University of California, Irvine Medical Center, Orange; 45 University of Chicago, Chicago, Illinois; 46 Spectrum Health Medical Group, Philadelphia, Pennsylvania; 47 University of Miami, Miami, Florida; 48 Emory University, Atlanta, Georgia; 49 Dartmouth-Hitchcock Medical Center, Lebanon, New Hampshire; 50 University of Missouri, Columbia; 51 University of Maryland School of Medicine, Baltimore; 52 University of Florida, Gainesville; 53 University of Southern California, Los Angeles; 54 Mayo Clinic, Jacksonville, Florida; 55 Neurology Associates, Lincoln, Nebraska; 56 Mayo Clinic, Rochester, Minnesota; 57 Georgetown University, Washington DC; 58 Biohaven, New Haven, Connecticut

## Abstract

**Importance:**

Myeloperoxidase is one of the most abundant peroxidase enzymes in activated myeloid cells. Myeloperoxidase inhibitors may have a clinical benefit in amyotrophic lateral sclerosis (ALS) by slowing neurodegeneration via reduced neuroinflammation and oxidative stress.

**Objective:**

To determine the safety, tolerability, and efficacy of verdiperstat, a selective myeloperoxidase inhibitor, in ALS.

**Design, Settings, and Participants:**

Verdiperstat was tested as a regimen of the HEALEY ALS Platform Trial, a multicenter, double-blind, perpetual platform design, randomized clinical trial, with sharing of trial infrastructure and placebo data across multiple regimens. The study was conducted at 54 ALS referral centers across the US from July 2020 to April 2022. Adult participants with a diagnosis of clinically possible, probable, laboratory-supported probable, or definite ALS defined by the revised El Escorial criteria were randomized to verdiperstat or regimen-specific placebo. An additional group of participants concurrently randomized to placebo from other regimens was included in the analyses.

**Interventions:**

Eligible participants were randomized in a 3:1 ratio to receive oral verdiperstat, 600 mg, twice daily or matching placebo for a planned placebo-controlled duration of 24 weeks.

**Main Outcomes and Measures:**

The primary efficacy outcome was change from baseline through week 24 in disease severity, as measured by a joint model of ALS Functional Rating Scale–Revised and survival, with the treatment effect quantified by the disease rate ratio (DRR), with DRR less than 1 indicating a slowing in disease progression of verdiperstat relative to placebo.

**Results:**

A total of 167 participants (mean [SD] age, 58.5 [11.4] years; 59 [35.3%] female; 108 [64.6%] male) were randomized to either verdiperstat (126 [75.4%]) or to placebo (41 [25.6%]). Among the participants randomized to the verdiperstat regimen, 130 (78%) completed the trial. The estimated DRR was 0.98 (95% credible interval, 0.77-1.24; posterior probability = 0.57 for slowing of disease progression [DRR <1]). Verdiperstat was estimated to slow progression by 2% vs placebo (95% credible interval, −23% to 24%; posterior probability 0.57). Verdiperstat was overall safe and well tolerated. Common adverse events in the verdiperstat group were nausea, insomnia, and elevated thyrotropin levels.

**Conclusions and Relevance:**

Results demonstrate that treatment with verdiperstat was unlikely to alter disease progression in ALS.

**Trial Registration:**

Clinical Trial Identifiers: NCT04297683 and NCT04436510

## Key Points

**Question:**

What is the effect of verdiperstat, a myeloperoxidase inhibitor, in amyotrophic lateral sclerosis?

**Findings:**

In the HEALEY ALS Platform Trial, a multicenter randomized clinical trial, 167 participants were randomized to verdiperstat or regimen-specific placebo, with an additional 81 participants concurrently randomized to placebo from other regimens. This regimen did not meet its prespecified primary efficacy end point, and there were no significant differences between active and placebo in the secondary end points.

**Meaning:**

Results show that in amyotrophic lateral sclerosis, the use of verdiperstat did not impact the progression of the disease.

## Teaser

This randomized clinical trial investigated the effect of verdiperstat, a myeloperoxidase inhibitor, in amyotrophic lateral sclerosis.

## Introduction

Verdiperstat is a selective, brain-permeable irreversible inhibitor of the myeloperoxidase (MPO) enzyme,^1^ an abundant peroxidase enzyme in activated myeloid cells, including activated microglia.^2^ In health, MPO catalyzes the production of hypochlorous acid, a key contributor to the oxygen-dependent activity of phagocytes. In systemic and neurological disease states, MPO-induced oxidative stress affects cell signaling and cell-cell interactions, contributing to an inflammatory cascade.^2,3,4,5,6^

Increased expression of MPO was found in neuronal and glial cells from Alzheimer disease (AD), Parkinson disease (PD), and multiple sclerosis from autopsy studies.^7^ Higher plasma MPO levels compared with age-matched healthy controls were observed in AD.^8^ Transgenic mouse models of AD, PD, and multiple system atrophy (MSA) showed increased expression of MPO in disease-relevant brain regions and associated iron-mediated cellular injury via ferroptosis and/or neuroinflammation^4,5,6^^,^ In AD and PD mouse models, lower MPO levels and activity were associated with reduced oxidative stress, reduced neuroinflammation, or improved disease-related phenotypes (less motor or cognitive decline).^5,9,10^ Furthermore, inhibition of MPO with verdiperstat demonstrated a reduction of neuroinflammation and oxidative stress in other neurodegenerative mouse models.^11^

The MPO/hypochlorous acid neuroinflammatory pathway was activated in motor neurons in an in vivo ALS mouse study, where motor performance improved after MPO inhibition.^12^ Postmortem MPO levels were higher in human amyotrophic lateral sclerosis (ALS) brain tissue than in controls without ALS.^13^ Altogether, these studies are supportive of the possibility that MPO activation may be deleterious in ALS and that its inhibition may be beneficial. An extended-release (ER) formulation of verdiperstat was previously found to be well tolerated up to doses of 900 mg twice a day in MSA and PD trials, supporting the pharmacokinetic, pharmacodynamic, and safety profile from these studies,^14^ and supported selection of a 600-mg twice-daily dose in this ALS trial.

Herein, we report the results of the HEALEY ALS Platform Trial regimen testing verdiperstat for the treatment of ALS.

## Methods

### Trial Design and Oversight

Verdiperstat was evaluated as a regimen (regimen B) of the HEALEY ALS Platform Trial, a double-blind, placebo-controlled, randomized clinical trial conducted at 52 Northeast ALS Consortium (NEALS) centers in the US between July 2020 and April 2022. The trial was conducted in accordance with Good Clinical Practice guidelines of the International Conference on Harmonization and ethical principles of the Declaration of Helsinki. Protocol approval was provided for all trial sites by a central investigational review board, the Partners Human Research Committee.^15^ All participants were required to provide written informed consent before screening.

The trial was designed by and conducted through the NEALS, a global collaborative trial network,^15^ in collaboration with Biohaven Pharmaceuticals. Participant-level data were obtained at each trial site and then sent to the Coordination Center at Massachusetts General Hospital (MGH). An independent data and safety monitoring board reviewed the unblinded safety data throughout the trial. Statistical analyses were performed by Berry Consultants and by the MGH Biostatistics team. Biohaven Pharmaceuticals provided the active drug and the matching placebo and was involved in the trial design, data analysis, and manuscript development. The master trial protocol is provided in Supplement 1. This study followed the Consolidated Standards of Reporting Trials (CONSORT) reporting guidelines.

### Trial Participants

Eligibility criteria for the platform trial included adults with a diagnosis of clinically possible, probable, laboratory-supported probable, or definite ALS defined by the revised El Escorial criteria,^16^ disease duration of 36 months or less, a vital capacity of 50% or greater predicted for age, height, and sex, the ability to swallow pills and liquids, and either no use of riluzole and/or edaravone or stable dosing of riluzole and/or edaravone for more than 30 days or 1 cycle, respectively. Participants were excluded from the verdiperstat regimen if taking strong cytochrome P450 1A2 (CYP1A2) or CYP3A4 inhibitors for more than 2 weeks. Participants self-identified with the following races and ethnicities: Asian, Black or African American, Hispanic or Latino, not Hispanic or Latino, White, or other (included multiracial, not reported, or unknown). Additional details are available in the trial protocol, which is available in Supplement 2.

### Trial Interventions and Procedures

Eligible participants were randomized in a 3:1 ratio to receive oral verdiperstat, 600 mg, twice daily or matching placebo within strata of edaravone and riluzole use for a planned placebo-controlled duration of 24 weeks. Additional details are provided in Supplement 1 and the eMethods in Supplement 3. Active drug (verdiperstat, 600 mg ER) and placebo were provided for oral administration twice a day. Clinic or phone visits were conducted at baseline and every 4 weeks thereafter through week 24, with a final phone follow-up at week 28. The full schedule of activities outlined in the study protocol (Supplement 2 and the eMethods in Supplement 3).

### Outcomes

The primary efficacy outcome was the change from baseline through week 24 in disease severity, as measured by a joint model of ALS Functional Rating Scale–Revised (ALSFRS-R)^17,18,19^ and survival. Secondary clinical efficacy outcomes (in hierarchical order) were analyzed in the full-analysis set (FAS) dataset (ie, the primary analysis population) and included 24-week change in isometric muscle strength as measured by hand-held dynamometry (HHD) of lower limb muscles and time to death or death-equivalent events (tracheostomy or permanent assisted ventilation more than 22 hours daily for more than 7 consecutive days). Additional outcome details are provided in the statistical analysis plan (Supplement 1, Supplement 2, and the eMethods in Supplement 3).^20,21,22,23^

### Sample Size Justification

Clinical trial simulation was used to quantify operating characteristics for each regimen within the master protocol.^24^ A sample size of 160 per regimen with randomization 3:1 active to placebo and sharing of controls across at least 3 concurrently enrolling regimens was shown to provide approximately 80% power for a 30% slowing in disease progression (common across mortality and function) and a 1-sided type I error less than 2.5%.^24,25^

### Analysis Populations

The primary analysis population, referred to as the FAS, included all participants randomized to the active treatment arm in the verdiperstat regimen, all participants randomized to the placebo arm within the verdiperstat regimen, and placebo participants from contributing regimens, referred to as shared placebo. The statistical analysis plan contains further details (Supplement 1 and Supplement 2).

### Statistical Analysis

The primary analysis is a bayesian shared parameter model of function and survival that provides an integrated estimate of the increase or decrease in the rate of disease progression among participants on verdiperstat relative to control.^24,25^ The model has components for function (measured by the ALSFRS-R) and survival that are linked through an integrated estimate of disease slowing in treatment relative to controls across the 2 end points (denoted as the disease rate ratio [DRR]). A DRR of 1 corresponds to no difference between treatments, whereas DRR values of less than 1 indicate slowing in disease progression on treatment relative to placebo. In the functional component, ALSFRS-R data are measured for those who have survived using a linear repeated measure model that adjusts for baseline covariates and accommodates potential differences in the shared control across regimens. The survival component is measured through an exponential proportional hazards model. The shared treatment effect between the ALSFRS-R and mortality components allows the participants who were lost to follow-up due to mortality to inform treatment effect estimates beyond their censored ALSFRS-R longitudinal data. The degree to which treatment effects on mortality inform the shared treatment effect parameter depends on the mortality rate within the study. Models included covariates for months since ALS symptom onset, prebaseline slope of ALSFRS-R, and baseline edaravone and riluzole use. Primary efficacy analyses used available baseline and postbaseline data for all participants, including those who discontinued trial drug but continued in the trial. When reporting DRR values with credible interval (CrI), please note that the term *CrI* is used in bayesian analyses and refers to the central 95% interval of the posterior distribution. Posterior probability is an estimate from bayesian analyses and refers to the distribution of the parameter of interest after updating the prior distribution with the likelihood of the observed data. Data analysis was conducted using SAS software, version 9.4 (SAS Institute), R, version 4.1.2 (R Project for Statistical Computing), and JAGS, version 4.2.0 (open source). A 2-sided *P* value <.05 was considered statistically significant.

Additional details about the primary and secondary analysis methods are available in the Supplement 1, Supplement 2, and the eMethods in Supplement 3.

## Results

### Trial Participants

**Figure 1.**
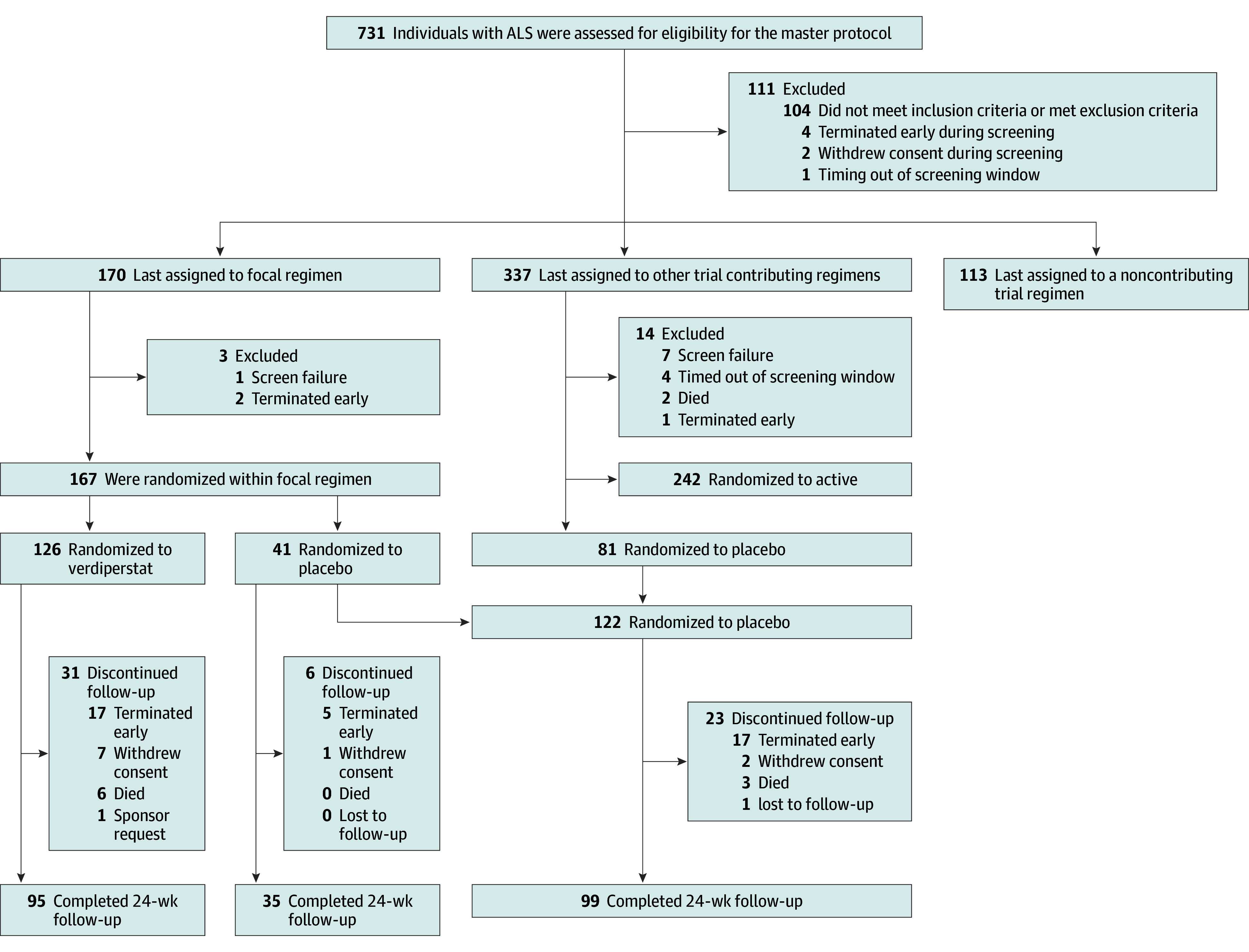
Consolidated Standards of Reporting Trials (CONSORT) Diagram Participants could have multiple reasons for exclusion from the master protocol and could screen for the master protocol more than once. The most common reasons for exclusion were as follows: (1) not meeting the criterion for slow vital capacity ≥50% (61/127 [48% of person-visits]), (2) having a clinically significant unstable medical condition (other than amyotrophic lateral sclerosis [ALS]) that would pose a risk to the participant (36/127 [28% of person-visits]), and (3) having used investigational treatments for ALS within 5 half-lives (if known) or 30 days (whichever is longer) before the master protocol screening visit (24/127 [19% of person-visits]). The 122 participants randomized to placebo included both the 81 from other regimens and the 41 randomized to placebo in this regimen.

A total of 731 participants were screened for the HEALEY ALS Platform Trial during recruitment for the verdiperstat regimen, of whom 170 were randomly assigned to the verdiperstat regimen. The primary analysis included 167 participants (mean [SD] age, 58.5 [11.4] years; 59 [35.3%] female; 108 [64.6%] male) randomized to either verdiperstat (126 [75.4%]) or to placebo (41 [25.6%]). An additional 122 concurrently randomized participants were assigned to placebo in other regimens (Figure 1). Among the participants randomized to the verdiperstat regimen, 130 (78%) completed the trial. Participants self-identified with the following races and ethnicities: 7 Asian (2.4%), 10 Black or African American (3.5%), 13 Hispanic or Latino (4.5%), 273 not Hispanic or Latino (94.5%), 265 White (91.7%), 7 other race (2.4%), 3 ethnicity not reported (1.0%).

Baseline demographic and disease characteristics are summarized in Table 1. The verdiperstat group, the regimen-specific placebo group, and the shared placebo group were balanced across baseline characteristics, including the concomitant use of riluzole and edaravone at study entry. At trial entry, a total of 219 participants (76%) were receiving riluzole, 70 (24%) were receiving edaravone, and 63 (21%) were receiving both riluzole and edaravone. No participants used sodium phenylbutyrate/tauroursodiol. Twenty-seven randomized participants had an ALSFRS-R bulbar subdomain scores less than 12, indicating some dysphagia at trial entry, but all eligible participants were able to swallow study capsules per site investigator’s discretion as per the eligibility criteria.

**Table 1.** Demographic and Baseline Characteristics of Participants in HEALEY ALS Platform Trial Verdiperstat Regimen and Combined Placebos

Statistic	No. (%)		
	Verdiperstat (n = 126)	Combined placebo (n = 122)^a^	Regimen-specific placebo (n = 41)^b^
Sex			
Female	47 (37.3)	40 (37.3)	12 (29.3)
Male	79 (62.7)	82 (67.2)	29 (70.7)
Race, No. (%)			
Asian	5 (4.0)	1 (0.8)	1 (2.4)
Black or African American	4 (3.2)	4 (3.3)	2 (4.9)
White	116 (92.1)	112 (91.8)	37 (90.2)
Other^c^	1 (0.8)	5 (4.1)	1 (2.4)
Ethnicity, No. (%)			
Hispanic or Latino	8 (6.3)	5 (4.1)	0
Not Hispanic or Latino	116 (92.1)	116 (95.1)	41 (100.0)
Not reported	2 (1.6)	1 (0.8)	0
Age at study entry, mean (SD), y	58.1 (11.54)	57.2 (11.74)	57.3 (11.07)
Bulbar onset, No. (%)	20 (15.9%)	19 (15.6)	7 (17.1)
El Escorial criteria, No. (%)			
Clinically definite ALS	45 (35.7)	48 (39.3)	18 (43.9)
Clinically probable ALS	53 (42.1)	28 (23.0)	11 (26.8)
Clinically probable ALS–laboratory supported	20 (15.9)	37 (30.3)	8 (19.5)
Clinically possible ALS	8 (6.3)	9 (7.4)	4 (9.8)
King’s stage, No. (%)			
1	13 (10.3)	27 (22.1)	9 (22.0)
2	39 (31.0)	32 (26.2)	10 (24.4)
3	36 (28.6)	27 (22.1)	14 (34.1)
4a/4b	2 (1.6)	0	0
4b	36 (28.6)	36 (29.5)	8 (19.5)
Riluzole and edaravone use at baseline			
Riluzole use	95 (75.4)	93 (76.2)	31 (75.6)
Edaravone use	28 (22.2)	31 (25.4)	11 (26.8)
Riluzole and edaravone use	25 (19.80)	28 (23.00)	10 (24.40)
Time since ALS symptom onset, mean (SD), mo	21.2 (8.93)	22.2 (8.39)	22.5 (7.91)
Time since ALS diagnosis, mean (SD), mo	10.5 (5.96)	10.3 (5.88)	10.5 (6.15)
Slow vital capacity–PPN, mean (SD), %	77.2 (20.37)	75.5 (16.51)	74.5 (17.50)
ALSFRS-R score, mean (SD)			
ALSFRS-R, total	34.3 (6.45)	35.2 (6.64)	34.3 (6.88)
ALSFRS-R, bulbar	10.2 (2.30)	10.2 (2.07)	9.9 (2.28)
ALSFRS-R, fine motor	7.1 (3.04)	7.6 (3.16)	7.1 (3.05)
ALSFRS-R, gross motor	6.7 (3.07)	7.1 (3.04)	7.0 (3.12)
ALSFRS-R, combined motor	13.9 (5.35)	14.7 (5.43)	14.1 (5.42)
ALSFRS-R, respiratory	10.3 (2.28)	10.2 (2.14)	10.3 (2.17)
Prebaseline ALSFRS-R slope, mean (SD), points/mo	0.75 (0.50)	0.63 (0.38)	0.64 (0.30)
Body mass index, mean (SD)^d^	26.4 (5.13)	27.8 (5.25)	27.1 (4.89)
Weight, median (IQR), kg	77.5 (67.1-86.6)	84.2 (70.9-93.8)	77.0 (70.7-90.1)
Serum NfL, median (IQR), pg/mL^e^	74.5 (52.3-107.3)	64.9 (46.0-98.7)	62.9 (47.0-83.2)

Abbreviations: ALS, amyotrophic lateral sclerosis; ALSFRS-R, Amyotrophic Lateral Sclerosis Functional Rating Scale–Revised; NfL, neurofilament light; PPN, percent predicted normal.

^a^ Combined placebos include regimen-specific placebo and shared placebos from other regimens.

^b^ The regimen was conducted before US Food and Drug Administration approval of sodium phenylbutyrate/tauroursodiol.

^c^ Other race includes multiracial, not reported, and unknown.

^d^ Calculated as weight in kilograms divided by height in meters squared.

^e^ NfL was analyzed using Quanterix Simoa platform.

### Primary Outcome

**Figure 2.**
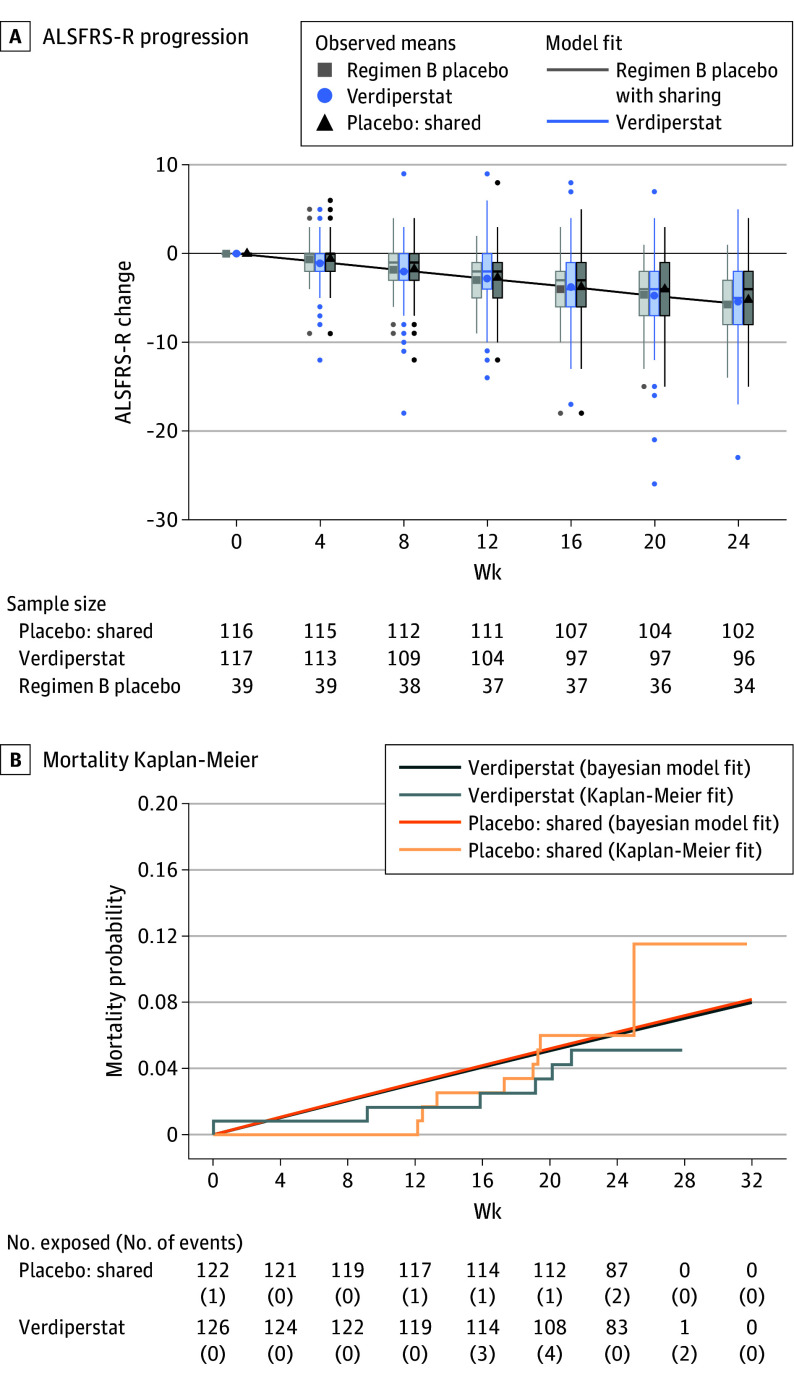
Change in Disease Severity Over 24 Weeks as Measured by Amyotrophic Lateral Sclerosis Functional Rating Scale–Revised (ALSFRS-R) and Survival (Primary Outcome) A, Box plots of the ALSFRS-R change from baseline over time for the active treatment, the regimen placebo, and the shared placebo. Points are the raw mean values at each visit. The solid lines are the model-estimated change in ALSFRS-R over time, adjusting for covariates. The estimate for the placebo regimen shares information from participants receiving placebo in other regimens. The numbers below the panel show the number of participants with known ALSFRS-R outcomes at each visit. The summary excludes all ALSFRS-R data from participants who died or had a death-equivalent event (PAV). B, Kaplan-Meier curves for death/PAV per arm and the model estimated exponential curves for the active treatment and pooled placebo. The numbers below the panel indicate the number of participants exposed and the number of death/PAV events per arm.

The estimated DRR was 0.98 (95% CrI, 0.77-1.24; posterior probability = 0.57 for slowing of disease progression [DRR<1]), which corresponds to a 2% slowing in the rate of progression in combined function, measured as ALSFRS-R, and survival for treated participants relative to placebo. The Combined Analysis of Function and Survival model showed the same results of no difference between verdiperstat group and placebo. At week 24, the change from baseline in the mean (SD) slopes of ALSFRS-R total score in the FAS dataset among survivors was −1.02 (0.09) vs −1.05 (0.09) points per month for verdiperstat vs shared placebo, respectively. The estimated rate of deaths or death equivalent events per month was 0.01 (posterior SD, 0) for verdiperstat vs 0.01 (posterior SD, 0) for shared placebo, respectively (Figure 2 and Table 2). Sensitivity analyses using the same bayesian shared-parameter model using data from the verdiperstat regimen alone (126 verdiperstat vs 41 regimen-specific placebo, 0 placebo from other regimens), data using shared placebo only from other orally administered study drug regimens (126 verdiperstat vs 82 shared placebo from the verdiperstat and other concurrent regimen [regimen C testing oral CNM-Au8]; CNM-Au8, tested in the HEALEY ALS Platform Trial, is a catalytically active, gold nanocrystal neuroprotective agent that enhances intracellular energy metabolism and reduces oxidative stress), and using a per-protocol analysis set (126 verdiperstat vs 122 shared placebo participants) showed similar nonsignificant results (posterior probability of DRR <1 ranged from 0.57-0.71). Verdiperstat was estimated to slow progression by 2% vs placebo (95% CrI, −23% to 24%; posterior probability 0.57). The study passed the every 12 weekly interim analyses 3 times, without meeting futility criteria. A summary of ALSFRS-R by visit for each treatment group is presented in eTable 1 in Supplement 3.

**Table 2.** Primary Analysis: Bayesian Shared Parameter Model^a^

Parameter	Posterior quantities				
	Median	Mean	95% credible interval^b^	Pr (DRR <1)^c^	Pr (DRR <0.9)^c^
DRR	0.98	0.98 (0.12)	0.77 to 1.24	0.57	0.23
ALSFRS-R slopes (points per month)					
Regimen placebo with sharing	−1.04	−1.05 (0.09)	−1.22 to −0.88	NA	NA
Verdiperstat	−1.02	−1.02 (0.09)	−1.20 to −0.85	NA	NA
Mortality event rate (events per month)					
Shared placebo	0.01	0.01 (0)	0.01 to 0.02	NA	NA
Verdiperstat	0.01	0.01 (0)	0.01 to 0.02	NA	NA
Covariates					
Time since onset of symptoms, mo	0.99	1.00 (0.05)	0.90 to 1.10	NA	NA
Prebaseline slope	1.27	1.27 (0.07)	1.14 to 1.42	NA	NA
Edaravone use	1.28	1.29 (0.13)	1.05 to 1.57	NA	NA
Riluzole use	0.85	0.85 (0.09)	0.68 to 1.04	NA	NA

Abbreviations: ALSFRS-R, Amyotrophic Lateral Sclerosis Functional Rating Scale–Revised; DRR, disease rate reduction; NA, not applicable; Pr, posterior probability.

^a^ Model adjusted for time since symptom onset, prebaseline ASLFRS-R slope, riluzole use at baseline, edaravone use at baseline.

^b^ Credible interval is used in bayesian analyses and refers to the central 95% interval of the posterior distribution.

^c^ Pr (DRR <1) and Pr (DRR <0.9) refer to posterior probabilities. It is an estimate from bayesian analyses and refers to the distribution of the parameter of interest after updating the prior distribution with the likelihood of the observed data.

### Secondary Outcomes

This study was not powered on the secondary outcomes including survival, and there were no group differences seen between verdiperstat and placebo groups for change from baseline in any of the secondary outcomes including hand-held dynamometry (HHD) upper limb, slow vital capacity, HHD lower limb, and ALSFRS-R total score (Table 3). There were no group differences between the active and placebo groups for mortality outcomes (Figure 2 and Table 3).

**Table 3.** Key Primary and Secondary Outcome Results Using the Repeated-Measures Model for Functional End Points and Cox Proportional Hazards Model for Survival Analyses

**End point**	24-wk Change estimate		Difference from shared placebo	
	**Verdiperstat, mean (SE)**	**Shared placebo, mean (SD)**	**Difference (SE) [95% CI]**	***P* value**
**Repeated-measures model**				
Full-analysis set population^a^				
No.	126	122	NA	NA
ALSFRS-R total score	−6.26 (0.55)	−5.95 (0.52)	−0.31 (0.74) [−1.76 to 1.15]	.68
HHD–upper (% change)	−29.29 (3.37)	−31.34 (3.12)	2.05 (4.47) [−6.73 to 10.83]	.65
SVC (% predicted)	−7.76 (1.52)	−8.05 (1.42)	0.29 (2.05) [−3.74 to 4.32]	.89
HHD–lower (% change)	−19.49 (3.75)	−21.78 (3.54)	2.30 (5.1) [−7.71 to 12.31]	.65
**End point**	**24-wk Change estimate**		**Difference from shared placebo**	
	**Verdiperstat**	**Regimen-only placebo**	**Difference (SE) [95% CI]**	***P* value**
Efficacy regimen only population^b^				
No.	126	41		
ALSFRS-R total score	−6.58 (0.6)	−6.69 (0.96)	0.11 (1.11) [−2.08 to 2.30]	.92
HHD–upper (% change)	−31.67 (3.35)	−39.91 (5.27)	8.24 (6.13) [−3.88 to 20.36]	.18
SVC (% predicted)	−8.34 (1.454)	−10.64 (2.28)	2.30 (2.69) [−3.01 to 7.61]	.39
HHD–lower (% change)	−19.77 (3.98)	−21.57 (6.28)	1.80 (7.31) [−12.65 to 16.25]	.81
**End point**	**24-wk Events**		**Comparison with shared placebo**	
	**Verdiperstat**	**Shared placebo**	**Hazard ratio (95% CI)**	***P* value**
**Cox proportional hazards model**				
Full-analysis set population				
No.	126	122	NA	NA
Survival, unadjusted, No./total No.	7/126	5/121	1.75 (0.60 to 5.69)	.32
Survival, adjusted^c^	NA	NA	1.54 (0.52 to 5.10)	.45
**End point**	**24-wk Events**		**Comparison with RGB placebo**	
	**Verdiperstat**	**Regimen-only placebo**	**Hazard ratio (95% CI)**	***P* value**
Efficacy regimen-only population				
No.	126	41	NA	NA
Survival, unadjusted, No./total No.	7/126	2/41	1.50 (0.39 to 9.88)	.60
Survival, adjusted^d^	NA	NA	1.53 (0.39 to 10.1)	.59

Abbreviations: ALSFRS-R, Amyotrophic Lateral Sclerosis Functional Rating Scale–Revised; HHD, hand-held dynamometer; RGB, regimen B; SVC, slow vital capacity.

^a^ Full-analysis set = participants randomized to RGB plus shared placebo from regimens B and C.

^b^ Efficacy regimen-only dataset = participants randomized to active and placebo in RGB only.

^c^ Models adjusted for sex, time since symptom onset, prebaseline ALSFRS-R slope, riluzole use at baseline, edaravone use at baseline.

^d^ Models adjusted for time since symptom onset, prebaseline ALSFRS-R slope, edaravone use at baseline, riluzole use at baseline and their interactions with visit.

### Blinding

Among the 145 participants receiving verdiperstat who were assessed at final disposition, the blinded site investigators correctly guessed assignments of 55% of participants (68% [26 of 38] assigned to placebo and 50% [54 of 107] assigned to verdiperstat; *P* value for association between true and guessed assignment by Fisher exact test = .06).

### Safety and Tolerability

There were 4 treatment-emergent adverse effects (TEAEs) resulting in death during the double-blind portion of the trial in the group treated with verdiperstat, none of which were attributed to verdiperstat. There were 2 treatment-related serious AEs in the group treated with verdiperstat, namely neutropenic fever (2 participants), which resolved after drug discontinuation.

The median (Q1-Q3) drug exposure duration in the group treated with verdiperstat (126 participants) was 167 (110-169) days. Early drug discontinuation and permanent dose reduction occurred more frequently in the verdiperstat group than in the placebo group (17% [21 of 126] and 11% [14 of 126], respectively, compared with 7% [8 of 122] and 2.5% [3 of 122], respectively, in shared placebo).

The 3 most common TEAEs that were assessed as related to study drug and led to early drug discontinuation were nausea (verdiperstat, 4.0% [5 of 126]; placebo, 0.8% [1 of 122]), fatigue (verdiperstat, 1.6% [2 of 126]; placebo, 0.8% [1 of 122]), and dizziness (verdiperstat, 0.8% [1 of 126]; placebo, 0%). Similarly, the 3 most common TEAEs that were related to study drug and led to permanent dose reduction were urticaria (verdiperstat, 0.8% [1 of 126]; placebo, 0%), tonsilitis (verdiperstat, 0.8% [1 of 126]; placebo, 0%), and tinnitus (verdiperstat, 0.8% [1 of 126]; placebo, 0%),

The TEAEs observed in this trial are summarized in eTable 2 in Supplement 3. The incidence of TEAEs of nausea (73 vs 26 events/100 participant-years taking verdiperstat and placebo, respectively), insomnia (51 vs 4 events/100 participant-years taking verdiperstat and placebo, respectively), and elevated thyrotropin level (22 vs 2 events/100 participant-years taking verdiperstat and placebo, respectively) were more common in the verdiperstat group vs placebo. There were 4 instances of thyrotropin levels greater than or equal to 10 mIU/L in 3 participants in the verdiperstat group and no instances in the placebo group. Of these 3 participants, 2 had normal free triiodothyronine (T_3_) and free thyroxine (T_4_) levels, and 1 did not have them reported. There was no notable weight loss in the verdiperstat treated group. Mean (SD) changes in weight over 24 weeks from baseline did not differ between groups (−3.4 [0.6] kg vs −2.9 [0.6] kg; difference = −0.44 kg; 95% CI, −2.1 to 1.2 kg; *P* = .59).

## Discussion

Verdiperstat, 600 mg, twice daily ER oral formulation tested as a regimen of the HEALEY ALS Platform Trial did not meet its prespecified primary efficacy criterion and was not significantly different than placebo for secondary end points. Verdiperstat was generally safe and well tolerated. Notable TEAEs attributed to verdiperstat were nausea, insomnia, and elevated thyrotropin levels. Neutropenic fever was observed in 2 participants and attributed to verdiperstat. The trial successfully met its target enrollment of 160 participants in 14 months, accrued the prespecified shared placebo from other regimens, and read out the top-line results just over 2 years after the first randomization. This trial serves as an example of using the platform trial design and shared infrastructure, to rapidly screen multiple novel experimental products in parallel for efficacy signal, and arrive at a go or no-go decision for a large confirmatory RCT.

The verdiperstat-attributable AEs of nausea and insomnia were similar to observations in clinical trials in PD.^26^ Rates of AEs of headache and fatigue were comparable between verdiperstat and placebo and are common observations in clinical practice in ALS. Elevated thyrotropin level was observed at a slightly higher frequency in this trial compared with the PD trial of verdiperstat.^26^ Of note, hypothyroidism was monitored as an AE of special interest in this trial based on preclinical studies showing that verdiperstat has off-target effects on thyroid peroxidase, which is structurally similar to MPO.

The pathological role and cellular origin of MPO in the central nervous system of human ALS remains unclear.^7^ Our expectation of benefit from verdiperstat in ALS is derived from other neurodegenerative disorders with overlapping biological pathways such as neuroinflammation, oxidative stress, and autophagy, as well as preclinical data in ALS models. Although verdiperstat and a few other experimental drugs targeting the neuroinflammation pathway or targets have not demonstrated efficacy in single-agent trials in ALS, there may still be value in pursuing additional experimental therapies modulating the neuroinflammation pathway in this disease. As elucidated in the recent review article by Maragakis et al,^27^ it is important to recognize that neuroinflammation, oxidative stress, and autophagy disease mechanisms and molecular targets in ALS have complex and dynamic interactions in different individuals and at different stages of the disease. The net direction and magnitude of change observed after a single agent experimental therapy targeting any of these pathways may not always be helpful to conclude lack of clinical benefit of an experimental therapy or pathway. Thus, it is possible that verdiperstat may be insufficient to impact ALS outcomes when used in isolation. Alternatively, it may be necessary to select for individuals who have higher MPO activation than others to more effectively target the use of verdiperstat. In addition, it is possible that specific forms of ALS, such as those associated with mutations in the *SOD1* gene, may be more susceptible to inflammation-induced oxidative stress^13^ and be better candidates for treatment with an agent such as verdiperstat. Future research studies are needed to test different compounds in the same mechanistic pathway or combination therapies using a multipronged approach to target more than 1 disease pathway simultaneously for better disease modulation. It is important to continue to invest in the development of robust preclinical models targeting specific ALS pathways, generate robust preclinical data to support clinical phase testing and importantly to continue the development of target engagement biomarkers for various implicated disease pathways. Taken together, these approaches would serve to improve the efficiency of bench-to-bedside translational efforts for novel experimental products in ALS, in addition to utilizing innovative adaptive clinical trial designs such as the platform trial design to accelerate therapeutic development in ALS.

### Limitations

This study has some limitations. First, this study recruited participants with different ALS phenotypes. Although this can be an appropriate in studies of agents with potential broad effects across the full spectrum of ALS, it may mask potential benefits in subgroups of responders with specific clinical or biological characteristics. Second, the 24-week duration of placebo-controlled treatment may be too short to evaluate investigational products that might require longer to have an effect on disease outcomes.

## Conclusions

In this randomized clinical trial, despite the negative results of this particular regimen, it is worth mentioning that the adaptive platform trial design efficiently answered the important question about clinical efficacy of verdiperstat in ALS. In this respect, the overall platform trial achieved its main objective of rapidly screening experimental drugs for clinical efficacy signal in an adequately powered phase 2 randomized clinical trial and excluding drugs and/or targets if not relevant in ALS. This and other scientific, statistical, and operational efficiencies that the platform trial paradigm brings to ALS therapeutic development is detailed in the review by Paganoni et al.^24^

## Notes

Supplement 1.
Master Trial Protocol.

Supplement 2.
Revised Trial Protocol.

Supplement 3.
eMethods.
**eTable 1.** ALSFRS-R by Visit **eTable 2.** Summary of Treatment-Emergent Adverse Events

Supplement 4. **Nonauthor Collaborators.** HEALEY ALS Platform Trial Study Group

Supplement 5.
Data Sharing Statement.
