# Supplementary material for: Verdiperstat in Amyotrophic Lateral Sclerosis: Results From the Randomized HEALEY ALS Platform Trial
Source: JAMA Neurol. 2025 Feb 17;82(4):333–43. doi: 10.1001/jamaneurol.2024.5249 (PMC11833655; doi:10.1001/jamaneurol.2024.5249)
Supplement: Supplement 1. — Master Trial Protocol. [file jamaneurol-e245249-s001.pdf]

HEALEY ALS Platform Trial

**Regulatory Sponsor:**	Merit Cudkowicz, MD, MSc<br>Healey Center for ALS at Mass General
**IND Number:**	144126
**Funding Sponsor:**	Healey Center for ALS at Mass General
**Protocol Date:**	12/15/2022
**Version Number:**	5.0

TABLE OF CONTENTS

TABLE OF CONTENTS 2

PROTOCOL APPROVAL SIGNATURE PAGE 6

STATEMENT OF COMPLIANCE 7

SIGNATURE PAGE 8

LIST OF ABBREVIATIONS 9

PROTOCOL SUMMARY 11

SCHEDULE OF ACTIVITIES 14

STUDY WORKFLOW 17

1 ETHICS/PROTECTION OF HUMAN PARTICIPANTS 19

1.1 Institutional Review Board 19
1.2 Ethical Conduct of Study 19
1.3 Participant Information and Consent 19
1.4 Changes in Conduct of the Study 20
1.4.1 Protocol Amendments 20
1.4.2 Premature Termination of Study Sites 20
1.5 Protocol Adherence 21

2 INTRODUCTION 22

2.1 Background Information and Rationale 22
2.2 Platform Trials: an efficient strategy to accelerate drug development and scientific discovery 23
2.3 Investigational Product Profile 23

3 OBJECTIVES AND ENDPOINTS 25

4 STUDY DESIGN 27

4.1 Overall Study Design and Plan 27

5 STUDY POPULATION 29

5.1 Number of Research Participants 29
5.2 Master Protocol Inclusion and Exclusion Criteria 29
5.2.1 Master Protocol Inclusion Criteria 29
5.2.2 Master Protocol Exclusion Criteria 30

6 PARTICIPANT SELECTION AND ENROLLMENT 32

6.1 Identifying Participants 32
6.2 Consenting Participants 32
Once received at the site, the individual who consented the participant via phone or telemedicine signs the informed consent form. 33
6.3 Ineligible Participants 33
6.4 Randomization 33

6.4.1 Randomization Procedures 33
6.4.2 Treatment Allocation 34
6.5 Discontinuation of Treatment and Terminations 35
6.5.1 Discontinuation of Investigational Product 35
6.5.2 Termination of Individual Research Participants 35
6.5.3 Termination of the Master Protocol or a Regimen by the Investigational New Drug (IND) Holder/ Sponsor 36
7 INVESTIGATIONAL PRODUCT AND PLACEBO 37
7.1 Investigational Product 37
7.1.1 Investigational Product Manufacturer 37
7.1.2 Labeling and Packaging 37
7.1.3 Acquisition and Storage 37
7.1.4 Destruction of Investigational Product 38
7.1.5 Investigator's Brochure 38
7.2 Dosage Changes 38
7.3 Participant Compliance 38
7.4 Overdose 38
7.5 Other Medications 38
7.5.1 Prohibited Medications 39
8 STUDY SCHEDULE 40
8.1 Master Protocol Screening Visit 41
8.1.1 Regimen Assignment and Within-regimen Randomiation 42
8.1.2 Screen Failures and Re-Assignment 42
8.2 Regimen Specific Screening Visit 43
8.3 Baseline Visit 43
8.4 Week 2 Telephone Visit 44
8.5 Week 4 Visit 44
8.6 Week 8 Visit 44
8.7 Week 12 Telephone Visit 45
8.8 Week 16 Visit 45
8.9 Week 20 Telephone Visit 46
8.10 Week 24 Visit 46
8.11 Follow-Up Safety Call 46
8.12 Early Termination Visit & Follow-up Safety Call for Early Terminations 47
8.13 Protocol Deviations 47
8.14 Missed Visits and Procedures 47
8.15 Recording Deaths and Vital Status Determination 48
9 CLINICAL ASSESSMENTS AND OUTCOME MEASURES 49
9.1 Clinical Variables 49
9.1.1 Vital Signs and Anthropometrics 49
9.1.2 Clinical Safety Laboratory Tests 49
9.1.3 12-Lead ECG 50
9.1.4 Physical Examination 50
9.1.5 Neurological Examination 50
9.1.6 Columbia-Suicide Severity Rating Scale 50
9.2 Endpoints 51
9.2.1 ALS Functional Rating Scale - Revised 51

9.2.2 Slow Vital Capacity 52
9.2.3 Measures of Muscle Strength 52
9.2.4 Training and Validation 52
9.2.5 Patient Reported Outcomes 52
9.3 Biofluid Collection 53
9.3.1 DNA Collection 53
9.3.2 Blood Biomarker Sample Collection 53
9.3.3 Urine Collection 53
9.3.4 Lumbar Puncture 53
10 SAFETY AND ADVERSE EVENTS 55
10.1 Definitions of AEs, Suspected Adverse Drug Reactions & SAEs 55
10.1.1 Adverse Event and Suspected Adverse Drug Reactions 55
10.1.2 Serious Adverse Events 56
10.2 Assessment and Recording of Adverse Events 57
10.2.1 Assessment of Adverse Events 57
10.2.2 Relatedness of Adverse Event to Investigational Product 58
10.2.3 Recording of Adverse Events 58
10.3 Adverse Events and Serious Adverse Events - Reportable Events 58
11 STATISTICAL CONSIDERATIONS 60
11.1 Statistical Design Overview 60
11.2 Analysis Plan 61
11.2.1 Primary Analysis Population and Shared Control 61
11.2.2 Primary Efficacy Analysis 62
11.2.3 Handling of Missing Data 62
11.2.4 Handling of Re-Screened and Repeat Participants 63
11.2.5 Inclusion of Multiple Doses 63
11.3 Interim Analyses and Trial Adaptations 63
11.3.1 Regimen Success 63
11.3.2 Regimen Futility 64
11.4 Secondary, Exploratory, and Sensitivity Analyses 64
11.4.1 Secondary Efficacy Analyses 64
11.4.2 Secondary Endpoints 64
11.4.3 Exploratory Analyses 64
11.4.4 Sensitivity Analyses 64
11.5 Safety Analyses 64
11.6 Sample Size Justification 65
12 DATA COLLECTION, MANAGEMENT, MONITORING, AND REPORTING 66
12.1 Quality Assurance 66
12.2 Role of Data Management 66
12.2.1 Data Collection 66
12.2.2 Data Entry and Edit Checks 66
12.2.3 Data Lock Process 67
12.3 Role of Study Monitors 67
12.3.1 Clinical Monitoring 67
12.3.2 Monitoring Report 67

12.4 Data and Safety Monitoring Board 67
12.5 Neurological Global Unique Identifier 68
12.6 Data Handling and Record Keeping 69
12.6.1 Confidentiality 69
12.6.2 Retention of Records 69
12.7 Reporting, Publications, and Notification of Results 70
12.7.1 Publication Policy 70
12.7.2 Data and Sample Sharing 70
12.7.3 Trial Registration 70

13 APPENDICES 71

Appendix I: El Escorial World Federation of Neurology Criteria for the Diagnosis of ALS 71
Appendix II: ALS Functional Rating Scale – Revised (ALSFRS-R) 73
Appendix III: Columbia-Suicide Severity Rating Scale (C-SSRS) Baseline Version 76
Appendix IV: Columbia-Suicide Severity Rating Scale (C-SSRS) Since Last Visit Version 79

REFERENCES 82

PROTOCOL APPROVAL SIGNATURE PAGE

HEALEY ALS Platform Trial

The undersigned accept the content of this protocol in accordance with the appropriate regulations and agree to adhere to it throughout the execution of the study.

**Merit Cudkowicz, MD, MSc**<br>Principal Investigator<br>Director, Healey Center for ALS at Mass General	**Signature**	**Date**
**Sabrina Paganoni, MD, PhD**<br>Co-Principal Investigator<br>Healey Center for ALS at Mass General	**Signature**	**Date**
**Marianne Kearney Chase**<br>Director of Research Operations, Healey Center for ALS at Mass General	**Signature**	**Date**

STATEMENT OF COMPLIANCE

This study will be conducted in compliance with the protocol, International Council for Harmonisation of Technical Requirements for Pharmaceuticals for Human Use (ICH), Guideline for Good Clinical Practice (GCP), and applicable regulatory requirements, including United States Code of Federal Regulations (CFR) Title 45 CFR Part 46 and Title 21 CFR Parts 50, 56, and 312.

SIGNATURE PAGE

I have read the attached ALS Platform Trial protocol entitled, "HEALEY ALS **Platform Trial**" dated **12/15/2022** (Version **5.0**) and agree to abide by all described protocol procedures. I agree to comply with the ICH Guideline on GCP, applicable FDA regulations and guidelines, including those identified in Title 21 CFR Parts 11, 50, 54, and 312, single Institutional Review Board (IRB) guidelines and policies, and the Health Insurance Portability and Accountability Act (HIPAA).

By signing the protocol, I agree to keep all information provided in strict confidence and to request the same from my staff. Study documents will be stored appropriately to ensure their confidentiality. I will not disclose such information to others without authorization, except to the extent necessary to conduct the study.

Site Name: ______________________________

Site Investigator: ______________________________

Signed: ____________________ Date: ____________

LIST OF ABBREVIATIONS

ADR	Adverse Drug Reaction
AE	Adverse Event
ALS	Amyotrophic Lateral Sclerosis
ALSFRS-R	ALS Functional Rating Scale-Revised
ATE	Active Treatment Extension
CC	Coordination Center
CDC	Centers for Disease Control and Prevention
CFR	Code of Federal Regulations
CRF	Case Report Form
CSF	Cerebrospinal Fluid
C-SSRS	Columbia-Suicide Severity Rating Scale
DCC	Data Coordination Center
DSMB	Data and Safety Monitoring Board
DNA	Deoxyribonucleic Acid
eCRF	Electronic Case Report Form
ECG	Electrocardiogram
EDC	Electronic Data Capture
FVC	Forced Vital Capacity
FDA	Food and Drug Administration
GCP	Good Clinical Practice
GMP	Good Manufacturing Practices
HHD	Hand Held Dynamometry
HIPAA	Health Insurance Portability and Accountability Act
ICF	Informed Consent Form
ICH	International Council for Harmonisation of Technical Requirements for Registration of Pharmaceuticals for Human Use
IND	Investigational New Drug Application
IRB	Institutional Review Board
IRT	Interactive Response Technology
ISF	Investigator Site File
ITT	Intent-to-Treat
LFTs	Liver Function Tests
LP	Lumbar Puncture
MGB	Mass General Brigham
MM	Medical Monitor
MoA	Mechanism of Action
MOP	Manual of Procedures
N	Number (typically refers to number of participants)

NCRI	Neurological Clinical Research Institute
NEALS	Northeast ALS Consortium
NeuroGUID	Neurological Global Unique Identifier
NfL	Neurofilament Light Chain
NIH	National Institutes of Health
OHRP	Office for Human Research Protections
PAV	Permanent Assisted Ventilation
PHI	Protected Health Information
PI	Principal Investigator
PK	Pharmacokinetic
RA	Reliance Agreement
RSA	Regimen-Specific Appendix
SAE	Serious Adverse Event
SDTs	Source Document Templates
SI	Site Investigator
sIRB	Single Institutional Review Board
SOA	Schedule of Activities
SOP	Standard Operating Procedure
SUSAR	Serious, Unexpected, Suspected Adverse Drug Reaction
SVC	Slow Vital Capacity
TEC	Therapy Evaluation Committee
TMF	Trial Master File
US	United States
VC	Vital Capacity
WOCBP	Women of Childbearing Potential

PROTOCOL SUMMARY

Study Title
HEALEY ALS Platform Trial

Study Indication
Amyotrophic Lateral Sclerosis (ALS)

Phase of Development
Phase 2/3

Rationale and Study Design
The HEALEY ALS Platform Trial is a perpetual multi-center, multi-regimen clinical trial evaluating the safety and efficacy of investigational products for the treatment of ALS.

The trial is designed as a perpetual platform trial. This means that there is a single Master Protocol dictating the conduct of the trial.

The Master Protocol describes the overall framework of the platform trial, including the target population, inclusion and exclusion criteria, regimen assignment and randomization schemes, study endpoints, schedule of assessments, trial design, the mechanism for adding and removing interventions, and the statistical methodology and recommended statistical methods for evaluating interventions.

Interventions (i.e., investigational products) are tested in trial regimens. Each trial regimen is described in its own Regimen-Specific Appendix (RSA) to the Master Protocol. The RSA will describe the nature of the intervention and its mechanism of action (MoA) including the mode and frequency of administration, dosage, the specific target population (to be selected within the pre-defined subsets of the Master Protocol), additional enrollment criteria (if any), sample size, and other specific intervention-related information and assessments (safety or other assessments that may be in addition to those outlined in the Master Protocol).

Allocation to Regimens
Participants that provide consent to the Master Protocol will be screened for Master Protocol-level inclusion and exclusion criteria. Those determined eligible will be randomly assigned with equal probability among all active regimens. This perpetual platform trial will continue enrolling research participants if there are regimens that are enrolling. As soon as pre-defined criteria for futility or success (if applicable) are met, or the target number of randomized participants in a regimen has been reached, enrollment will stop in that regimen.

Number of Planned Participants and Treatment Arms
The sample size enrolled in each regimen will be determined based on the specifics of the intervention, anticipated effect size, the expected variability in the enrolling population, and

number of treatment arms (e.g., dosage within a regimen, if applicable). Those participants that meet the Master Protocol inclusion and exclusion criteria will be randomly assigned to a regimen. Within each regimen, participants will then be randomized a second time in a 3:1 ratio to active treatment or matching placebo.

Planned Number of Sites
Research participants will be enrolled from up to approximately 80 centers in the US.

Treatment Duration
Treatment duration of the placebo-controlled period is a maximum of 24-weeks for each regimen. The duration of the Active Treatment Extension (ATE) period will be described in the respective RSAs.

Post-treatment Follow-up Duration
Will be described in the RSAs.

Study Objectives and Endpoints
Default Primary Efficacy Objective:
To evaluate the efficacy of multiple investigational products, as compared to placebo, on ALS disease progression.

Secondary Efficacy Objective:

- To evaluate the effect of multiple investigational products on selected secondary measures of disease progression.

Safety Objective:

- To evaluate the safety of multiple investigational products for people with ALS.

Exploratory Efficacy Objective:

- To evaluate the effect of multiple investigational products on selected biomarkers and endpoints.

Default Primary Efficacy Endpoint:
Change from baseline through week 24 in disease severity as measured by the ALS Functional Rating Scale-Revised (ALSFRS-R) total score and survival.

Secondary Efficacy Endpoints:

- Change from baseline through week 24 in respiratory function as assessed by slow vital capacity (SVC).
- Change from baseline through week 24 in muscle strength as measured isometrically using hand-held dynamometry (HHD) and grip strength.
- Survival.

Safety Endpoints:

- Treatment-emergent adverse and serious adverse events.
- Changes in laboratory values and treatment-emergent and clinically significant laboratory abnormalities.
- Changes in ECG parameters and treatment-emergent and clinically significant ECG abnormalities.
- Treatment-emergent suicidal ideation and suicidal behavior.

Exploratory Efficacy Endpoints:

- Changes in biofluid biomarkers.
- Changes in patient reported outcomes.

Investigational Products

Investigational products will be tested at different times (in parallel and sequentially) as described in this Master Protocol. This Master Protocol describes the common framework of the study. Each investigational product will have its own RSA to the protocol.

SCHEDULE OF ACTIVITIES

The Master Protocol sets out the minimum visit and assessment requirements that must be incorporated into each RSA. As per the Schedule of Activities (SOA) below, visits occur every 4 weeks and will be clinic-, phone-, or telemedicine-based, as applicable. Additional assessments may be added and will be described in each RSA. The Master Protocol allows a 24-week duration of placebo-controlled treatment for an intervention and an additional Active Treatment Extension (ATE) period, as described in each RSA.

Activity	Master Protocol Screening[1]	Regimen Specific Screening[1]	Baseline	Week 2	Week 4[13]	Week 8[13]	Week 12	Week 16[13]	Week 20	Week 24	Follow-Up Safety Call
	Clinic	Clinic	Clinic	Phone	Clinic	Clinic	Phone	Clinic	Phone	Clinic	Phone
	-42 to -1 Days	Refer to RSA	Day 0	Day 14 ±3	Day 28 ±7	Day 56 ±7	Day 84 ±3	Day 112 ±7	Day 140 ±3	Day 168 ±7	Refer to RSA
Written Informed Consent[2]	X	X									
Inclusion/Exclusion Review	X	X[3]									
Regimen Specific Screening procedure(s)		X									
ALS & Medical History	X										
Demographics	X										
Physical Examination	X										
Neurological Exam	X										
Vital Signs[4]	X		X		X	X		X		X	
Slow Vital Capacity	X[12]		X			X		X		X	
Muscle Strength Assessment			X			X		X		X	
ALSFRS-R	X		X		X	X	X	X	X	X	
Patient Reported Outcomes[10]			X			X		X		X	
12-Lead ECG	X									X	
Clinical Safety Labs[5]	X		X		X	X		X		X	
Concomitant Medication Review	X	X	X		X	X	X	X	X	X	X
Adverse Event Review[6]	X	X	X	X	X	X	X	X	X	X	X

Activity	Master Protocol Screening[1]	Regimen Specific Screening[1]	Baseline	Week 2	Week 4[13]	Week 8[13]	Week 12	Week 16[13]	Week 20	Week 24	Follow-Up Safety Call
	Clinic	Clinic	Clinic	Phone	Clinic	Clinic	Phone	Clinic	Phone	Clinic	**Phone**
	-42 to -1 Days	Refer to RSA	Day 0	Day 14 ±3	Day 28 ±7	Day 56 ±7	Day 84 ±3	Day 112 ±7	Day 140 ±3	Day 168 ±7	Refer to RSA
Columbia-Suicide Severity Rating Scale			X		X	X		X		X	
Biomarker Blood Collection			X			X		X		X	
Biomarker Urine Collection			X			X		X		X	
DNA Collection[7] (optional)			X								
CSF Collection[11]			X					X			
Assignment to a regimen	X										
Randomization within a regimen		X									
Administer/Dispense Study Drug			X[8]			X		X			
Study Drug Accountability/Compliance				X[14]	X	X	X[14]	X	X[14]	X	
Exit Questionnaire										X	
Vital Status Determination										X[9]	

[1] Master Protocol Screening procedures must be completed within 42 days to 1 day prior to the Baseline Visit. Refer to RSA for details about RSA Specific Screening procedures, if any, should be performed.
[2] During the Master Protocol Screening Visit, participants will be consented via the Master Protocol informed consent form (ICF). After a participant is assigned to a regimen, participants will be consented a second time via the regimen-specific ICF.
[3] At the Regimen Specific Screening Visit, participants will have regimen-specific inclusion and exclusion criteria assessed, if applicable.
[4] Vital signs include weight, systolic and diastolic pressure, respiratory rate, heart rate and temperature. Height in cm measured at Master Protocol Screening Visit only.
[5] Clinical safety labs include hematology (CBC with differential), complete chemistry panel, liver function tests, thyroid function and urinalysis. Coagulation panel will occur at the Master Protocol Screening and Week 16 Visits or as in the RSA. Serum pregnancy testing will occur in women of child-bearing potential at the Master Protocol Screening Visit and as necessary during the study. Pregnancy testing is only repeated as applicable if there is a concern for pregnancy.
[6] Adverse events that occur after signing Master Protocol consent form will be recorded.
[7] The DNA sample can be collected after the Baseline Visit if a baseline sample is not obtained or the sample is not usable.
[8] Administer first dose of study drug only after Baseline Visit procedures are completed
[9] Vital status, defined as a determination of date of death or death equivalent or date last known alive, will be determined for each randomized participant at the end of the placebo-controlled period of their follow-up (generally the Week 24 Visit, as indicated). If at that time the participant is alive, his or her vital status should be determined again at the time of the last participant's last visit (LPLV) of the placebo-controlled period of a given regimen. We may also ascertain vital status at later time points by using publicly available data sources as described in section 8.15.b

[10] Each RSA will detail which PRO is collected at each Visit. Specific PRO collection may differ from this Schedule of Activities.
[11] CSF collection may differ from the Schedule of Activities, and will be performed as described in each RSA.
[12] If required due to pandemic-related or other restrictions, Forced Vital Capacity (FVC) performed by a Pulmonary Function Laboratory evaluator may be used for eligibility (Master Protocol Screening ONLY).
[13] Visit may be conducted via phone or telemedicine with remote services instead of in-person if needed to protect the safety of the participant due to a pandemic, disability or other medical reason.
[14] Drug accountability will not be done at phone visits. A drug compliance check in will be conducted during phone visits to ensure participant is taking drug per dose regimen and to note any report of missed doses.

STUDY WORKFLOW

Figure 1. Example Master Protocol Schema

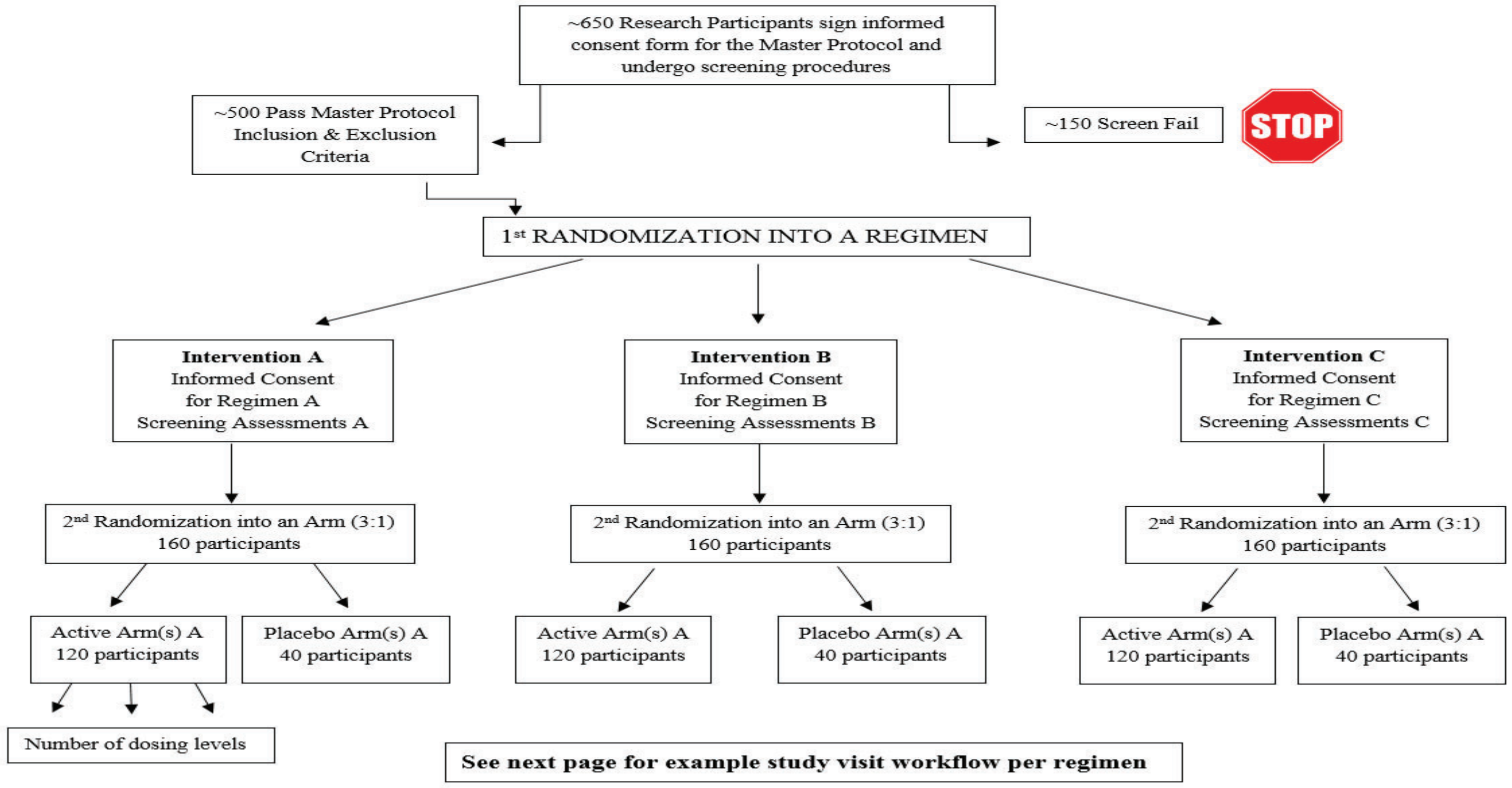

Figure 1 represents an example of the study schema assuming three concurrently enrolling regimens. The Master Protocol schema will change depending on the number of regimens that are enrolling at any given time. Intervention A investigates an active treatment with multiple dosing levels. In this example, regimen sample size is 160 participants, actual regimen sample size will be specified in each RSA.

Figure 2. Example Study Visit Workflow per Regimen

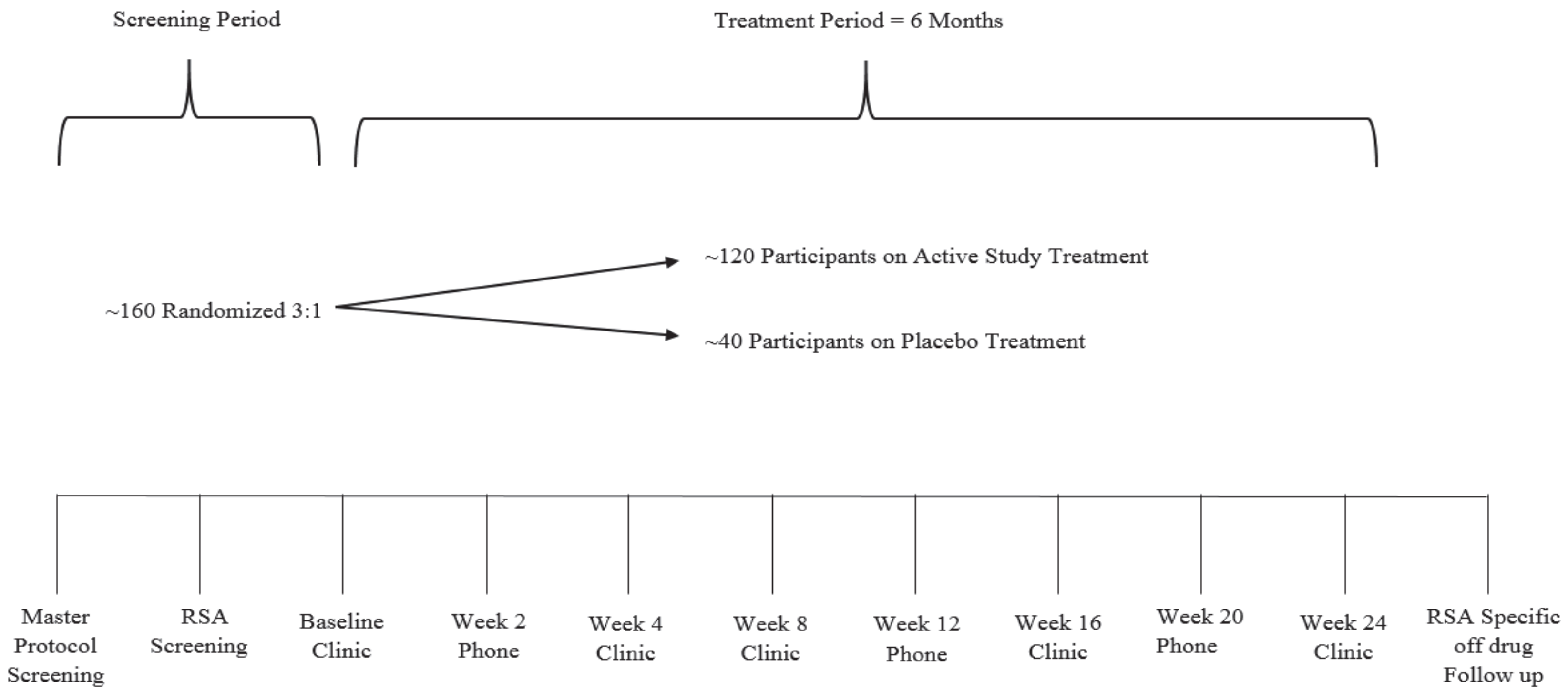

Figure 2 represents an example of the study visits that will occur over 24 weeks for all participants that are assigned to a regimen. In this example, regimen sample size is 160 participants, actual regimen sample size will be specified in each RSA.

1 ETHICS/PROTECTION OF HUMAN PARTICIPANTS

1.1 Institutional Review Board

This Master Protocol will be conducted in compliance with current GCP and Title 21 Part 56 of the United States of America CFR relating to IRBs. The Mass General Brigham (MGB) IRBs located in Boston, MA, collectively known as the MGB Human Research System (MGB HRS), have been selected by the Northeast ALS Consortium (NEALS) to serve as the single IRB (sIRB) for the Master Protocol. NEALS is a non-profit group of researcher institutions, each of which is a NEALS Member, who collaboratively conduct clinical research in Amyotrophic Lateral Sclerosis and other motor neuron diseases. Sites participating in this Master Protocol must have an executed sIRB Authorization Agreement (Reliance Agreement or RA) to rely on the MGB HRS to participate.

1.2 Ethical Conduct of Study

The study will be conducted in accordance with GCP defined by the ICH and the ethical principles of the Declaration of Helsinki.

The study will be conducted in compliance with the protocol. The protocol and any amendments as well as the participant informed consent will receive sIRB approval prior to initiation of the study.

Study personnel involved in conducting this study will be qualified to perform their respective task(s) as confirmed by the site and collection of required documentation.

This study will not use the services of study personnel where sanctions have been invoked or where there has been scientific misconduct or fraud (e.g., loss of medical licensure, debarment).

1.3 Participant Information and Consent

This study will be conducted in compliance with Title 21 Part 50 of the United States of America CFR, Federal Regulations and ICH Guidance Documents pertaining to informed consent. At the first visit, prior to initiation of any study-related procedures not performed as clinic visit standard of care (e.g., vital signs, weight), participants will be informed about the nature and purpose of the Master Protocol, participation/termination conditions, and risks and benefits. Participants will be given adequate time to ask questions and become familiar with the Master Protocol prior to providing consent to participate. Participants will give their documented informed consent to

participate in the Master Protocol and will be provided with a copy of the fully executed Master Protocol consent form for their records.

Participants meeting Master Protocol eligibility criteria will be randomly assigned to a regimen.

Participants will then be informed about the intervention specific to their assigned regimen and any participation/termination conditions or risks and benefits specific to that regimen. Participants will give their written consent to participate in their assigned regimen and will be provided with a copy of the fully executed regimen-specific consent form for their records. Participants meeting additional eligibility criteria required by their assigned regimen, if any, will be randomized in a 3:1 ratio to receive either active treatment or matching placebo in that regimen.

In some situations, an individual may be re-assigned to multiple different regimens, if eligible. The procedures detailing the re-assignment process can be found in Section 8.1.2 Screen Failures and Re-Assignment.

1.4 Changes in Conduct of the Study

1.4.1 Protocol Amendments

Any change to the Master Protocol will be documented in a protocol amendment, issued by the Sponsor. Master Protocol amendments will be submitted for approval to the sIRB prior to implementation. Written informed re-consent for continued participation in the study may be required by participants already enrolled in the Master Protocol.

As regimens are added to the Platform Trial, they will be submitted for approval to the sIRB prior to implementation. Each regimen will be added as an appendix to the Master Protocol prior to any participant being assigned to that regimen. Addition of regimens will not require re-consent. Regimens that are stopped for futility or ended early due to success (if applicable) will not result in an amendment to the Master Protocol.

1.4.2 Premature Termination of Study Sites

The Sponsor reserves the right to terminate the participation of individual study sites. Conditions that may warrant termination include, but are not limited to, insufficient adherence to protocol requirements and failure to enter participants at an acceptable rate.

1.5 Protocol Adherence

Each Site Investigator (SI) must adhere to the Master Protocol detailed in this document and agree that any changes to the protocol must be approved by the sIRB. Each SI will be responsible for enrolling into the Master Protocol only those study participants who have met all Master Protocol eligibility criteria, and for enrolling into a Regimen only those study participants who have met the additional eligibility criteria (if any) of the corresponding RSA.

2 INTRODUCTION

2.1 Background Information and Rationale

ALS is a progressive, fatal neurodegenerative disease. ALS is characterized by motor neuron loss resulting in muscle weakness and atrophy, disability, and eventually death from failure of the ventilatory muscles[1]. The median age of onset is 55 years and average survival is 3-5 years after onset of first symptoms[2]. While the incidence of ALS is comparable to that of multiple sclerosis (approximately 2/100,000), its prevalence is much lower because of its rapid progression (about 5/100,000)[3]. The only FDA-approved disease modifying medications, riluzole, edaravone, and Relyvrio confer only a modest survival benefit[4].

While therapies for ALS remain limited, basic and translational ALS research has resulted in numerous influential discoveries in recent years, including breakthroughs in genetics and progress in our understanding of disease mechanisms, therapeutic targets, and biomarkers. These discoveries have led to a large pipeline of potential therapies that await testing in clinical trials, suggesting that we are at a time of great opportunity to translate advances in the understanding of ALS into meaningful treatments for people.

Given the poor prognosis and dearth of effective treatments, clinical trials are of primary importance for people with ALS, their families, their providers, and the entire ALS clinical and scientific community[5]. Clinical trials, however, can be complex, time-consuming, and expensive. Challenges to ALS clinical trials are both operational and scientific, and innovative clinical trials are needed to accelerate the path to effective treatments[6-9].

Operational challenges include inefficiencies in institutional approvals and trial management that may lead to delays in study start-up, slow execution times, and sub-optimal recruitment and retention[10]. Barriers to trial access are so substantial that less than 10% of the ALS population is estimated to participate in clinical research[11, 12]. Fortunately, experience in selected disease-specific networks and federally-funded trial networks demonstrates that logistical trial challenges are surmountable with an infrastructure designed for efficiency and collaboration. In fact, clinical trial networks can be very productive if built with appropriate infrastructure, leadership, and cooperation. Several of these networks leverage efficient infrastructure models, such as the use of a single IRB, Master Clinical Trial Site Agreements, and centralized recruitment and retention strategies[13].

Scientific challenges to ALS therapy development include disease rarity, patient heterogeneity, lack of validated surrogate endpoints, and a relatively rapid disease course. To overcome some of these barriers, the ALS trial community has stepped up biomarker discovery efforts and devoted substantial resources to the development of novel outcome measures for use in early phase clinical trials[14, 15]. Routine incorporation of these novel endpoints into clinical trials is a critical step forward and holds the promise to improve the chances of success of the drug development pipeline. Yet, progress is hindered by the lack of coordination among different biomarker discovery and outcome measure development efforts[15]. Several previous and current research projects target different patient populations and analyze novel biomarkers/outcome measures using different assays and procedures, and also include variable schedules of assessments. A

major, coordinated collaboration that includes academia, industry, and patient-advocacy organizations is needed to innovate the ALS clinical trial landscape[6-9].

2.2 Platform Trials: an efficient strategy to accelerate drug development and scientific discovery

Traditionally, clinical trials are designed by a single sponsor to answer only one question: does a single investigational product work? These trials include a pre-specified number of treatment arms (generally limited to two or three arms) and have a finite duration based on the time required to answer the trial question. Moreover, each trial requires an expensive, ad hoc trial infrastructure that is dismantled at the end of the trial.

In contrast, platform trials are designed to investigate multiple investigational products in parallel and sequentially with the capability to adapt over time. Platform trials do not have a pre-specified end date: the platform remains open long-term and is available to evaluate new investigational products as they become available[16]. The trial infrastructure is built at the beginning and is shared across different treatments, leading to operational efficiencies. Investigational products are compared to a shared placebo group. Sharing of placebo participants is possible because all investigational products are evaluated using a common Master Protocol[17]. The Master Protocol describes the framework of the study, including the trial population, inclusion and exclusion criteria, regimen assignment and randomization schemes, a schedule of assessments, primary, secondary and exploratory outcomes, statistical methodology, and planned analyses that are common for all investigational products to be tested.

Platform trials are an ideal setting to advance the scientific understanding of the disease and novel endpoints with a coordinated strategy. Thanks to its coordinated structure and common data and sample acquisition processes, the platform trial can serve as a natural history registry and bio-repository from placebo participants[17]. With the testing of multiple investigational products using a common protocol and uniform data and sample acquisition processes, the platform is designed to answer multiple scientific questions and to serve as a source of data that can be used to enhance the design of other research projects. Further, accumulated learnings about endpoint behavior can be leveraged to adapt the Master Protocol so that future investigational products will be studied using more efficient biomarkers, outcome measures, and analyses[17, 18].

2.3 Investigational Product Profile

In this trial, multiple investigational products for ALS will be tested simultaneously or sequentially. These investigational products will be provided by different partners, either pharmaceutical companies or academic groups.

This trial is designed as a perpetual platform trial. This means that there is a single Master Protocol dictating the conduct of the trial. The additional details that govern the testing of each investigational product will be summarized in separate RSAs.

During the trial, a regimen may be discontinued early due to futility or safety concerns. Additionally, a regimen may be added to the trial based on the ability of the trial to accommodate their incorporation.

Selection of interventions will be done by the Therapy Evaluation Committee (TEC) based on evidence supporting the intended mechanism of action, target engagement, previous pre-clinical data and Phase I or other clinical data (including safety data available for each compound), and compatibility with the Master Protocol.

Each RSA that is part of the HEALEY ALS Platform Trial will describe:

1. Specific additional inclusion or exclusion criteria for that investigational product, if any. The RSA will include a summary of the product characteristics, including mechanism of action and pharmacology background, drug profile including a summary of available preclinical and clinical data including pharmacokinetic (PK)/pharmacodynamic, target of action, biomarker data, drug-drug interaction, safety data, prohibited medications (as applicable) and benefit-risk assessment. For a more detailed description of the investigational product profile, the RSA will refer to the current Investigator's Brochure.

2. Specific biomarker assessments not performed as part of the Master Protocol, if any, which are specific to the intervention and proposed mechanism of action.

3. Specific safety assessments not performed as part of the Master Protocol, if any, which are specific to known toxicology or potential safety concerns associated with the intervention.

4. Specific information on study methodology, such as the primary endpoint, maximum sample size, number of arms (e.g., to accommodate multiple dosages), and details for interim analyses for early futility and early success (if applicable).

3 Objectives and Endpoints

OBJECTIVES	ENDPOINTS	JUSTIFICATION FOR ENDPOINTS
Primary		
To evaluate the efficacy of multiple investigational products on ALS disease progression.	Change in disease severity as measured by the ALS Functional Rating Scale-Revised (ALSFRS-R) total score and survival	*The ALSFRS-R measures function in daily activities and is an established scale for monitoring disease progression in ALS.*
Secondary		
To test the effect of multiple investigational products on selected secondary measures of disease progression.	• Change in respiratory function as assessed by slow vital capacity (SVC).<br>• Change in muscle strength as measured isometrically using hand-held dynamometry (HHD) and grip strength.<br>• Survival.	*Decline in respiratory function is a direct result of the known pathophysiology of the ALS and demonstration of a treatment benefit on respiratory endpoints may also provide evidence of effectiveness.*<br><br>*Loss of strength is a hallmark of disease progression in ALS and meaningful differences in muscle strength should be supportive of an effect on measures of function in activities of daily living.*
Safety		
	• Treatment-emergent adverse and serious adverse events.	*Toxicities associated with investigational products may*

OBJECTIVES	ENDPOINTS	JUSTIFICATION FOR ENDPOINTS
To evaluate the safety of multiple investigational products for people with ALS.	• Changes in laboratory values and treatment-emergent and clinically significant laboratory abnormalities.<br>• Changes in ECG parameters and treatment-emergent and clinically significant ECG abnormalities.<br>• Treatment-emergent suicidal ideation and suicidal behavior.	*manifest as clinical signs and symptoms, new diagnoses, laboratory or cardiac changes and abnormalities, or suicidality.*
Exploratory		
To test the effect of multiple investigational products on selected biomarkers and endpoints.	• Changes in biofluid biomarkers.<br>• Changes in patient reported outcomes.	*These endpoints have been chosen to provide greater understanding of ALS and may provide opportunities for identification of surrogate endpoints that are reasonably likely to predict clinical benefit and that might serve as a basis for accelerated approval in future trials.*

4 STUDY DESIGN

4.1 Overall Study Design and Plan

This is a perpetual, multicenter, multi-regimen, randomized, placebo-controlled, adaptive platform clinical trial evaluating the safety and efficacy of multiple investigational products simultaneously or sequentially in ALS.

There will be multiple interventional regimens, each consisting of the research participants receiving either the active treatment or its matching placebo. Research participants, investigators and site staff will not be blinded to the regimen assignment, but they will be blinded to active product or matching placebo assignment and this blind will be maintained throughout the ATE period of the study. Regimens may start at different time points during the trial.

This Master Protocol describes the framework for the trial design population and the minimum inclusion and exclusion criteria for all regimens. Each RSA will contain intervention-specific information and will define any additional trial elements and regimen-specific eligibility criteria that may exist.

The number of research participants, the duration of treatment for each intervention, and the follow-up period will all be described in the RSA. The primary endpoint will be assessed for all interventions every 4 weeks, either on-site or via phone.

Interim analyses for regimens will begin when at least one regimen has 40 randomized participants within that regimen who have had the opportunity to complete at least 24 weeks of follow-up. Interim analyses will continue to occur simultaneously for all actively enrolling regimens every 12 weeks. A regimen is eligible to stop early for futility (all regimens) or for success (for applicable regimens only) at any interim analysis once there are 40 randomized participants within that regimen who have had the opportunity to complete at least 24 weeks of follow-up. The default design for a regimen will include stopping early only for futility.

At each interim analysis, a set of possible changes or adaptions that an intervention is eligible for will be checked.

- An intervention can be found to have failed to favorably modify the progression of disease and be deemed futile or unsafe. Upon meeting the criterion for futility, enrollment in that regimen would be discontinued and end of study procedures would be initiated. Research participants in that regimen may be re-screened for entry into the Master Protocol after the mandatory wash-out period, if eligible.

- Accrual and maximum exposure of enrolled participants may be reached for an intervention. In this case, this is the end of that regimen and all research participants in that regimen would have completed follow-up through week 24 of the placebo-controlled period. Research participants will continue in the ATE period or may be re-screened for entry into the Master Protocol after the mandatory wash-out period, if eligible.

- If a regimen includes more than one active arm to allow for multiple dosing levels or other variations in treatment, then rules specifying the allocation to these arms can be included. This will be detailed in the RSA.

5 STUDY POPULATION

5.1 Number of Research Participants

Research participants that meet the Master Protocol inclusion and exclusion criteria listed below will be further screened and randomly assigned to a regimen.

The sample size enrolled in each regimen will be determined based on the specifics of the intervention, anticipated effect size, and number of treatment arms evaluated within that regimen.

5.2 Master Protocol Inclusion and Exclusion Criteria

5.2.1 Master Protocol Inclusion Criteria

These are the inclusion criteria that research participants must meet to be eligible to enter the Master Protocol and are common for all regimens:

1. Sporadic or familial ALS diagnosed as clinically possible, probable, lab-supported probable, or definite ALS defined by revised El Escorial criteria (Appendix I)**.**
2. Age 18 years or older.
3. Capable of providing informed consent and complying with study procedures, in the SI's opinion.
4. Time since onset of weakness due to ALS ≤ 36 months at the time of the Master Protocol Screening Visit.
5. Vital Capacity ≥ 50% of predicted capacity at the time of the Master Protocol Screening Visit measured by Slow Vital Capacity (SVC), or, if required due to pandemic-related restrictions, Forced Vital Capacity (FVC) measured in person.
6. Participants must either not take riluzole or be on a stable dose of riluzole for ≥ 30 days prior to the Master Protocol Screening Visit.
7. Participants must either not take edaravone or have completed at least one cycle (typically 14 days) of edaravone prior to the Master Protocol Screening Visit.
8. Participants must have the ability to swallow pills and liquids at the time of the Master Protocol Screening Visit and, in the SI's opinion, have the ability to swallow for the duration of the study.
9. Geographically accessible to the site.
10. Participants must either not take Relyvrio/ Albrioza or have started Relyvrio/ Albrioza ≥ 30 days prior to the Master Protocol Screening Visit.

5.2.2 *Master Protocol Exclusion Criteria*

These following exclusion criteria are common for all regimens:

1. Clinically significant unstable medical condition (other than ALS) that would pose a risk to the participant, according to SI's judgment (e.g., cardiovascular instability, systemic infection, untreated thyroid dysfunction), or clinically significant laboratory abnormality or EKG changes.

   Clinically significant abnormal liver or kidney function is exclusionary. The following values [alanine aminotransferase (ALT) or aspartate aminotransferase (AST) > 3 times the upper limit of normal (ULN) or estimated Glomerular Filtration Rate (eGFR) < 30 $mL/min/1.73m^2$] are exclusionary regardless of clinical symptoms.

2. Presence of unstable psychiatric disease, cognitive impairment, dementia or substance abuse that would impair ability of the participant to provide informed consent, in the SI's opinion.
3. Active cancer or history of cancer, except for the following: basal cell carcinoma or successfully treated squamous cell carcinoma of the skin, cervical carcinoma in situ, prostatic carcinoma in situ, or other malignancies curatively treated and with no evidence of disease recurrence for at least 3 years.
4. Use of investigational treatments for ALS (off-label use or active participation in a clinical trial) within 5 half-lives (if known) or 30 days (whichever is longer) prior to the Master Protocol Screening Visit. (*Please refer to the Manual of Procedures (MOP) for current list of experimental therapies*)
5. Exposure at any time to any gene therapies under investigation for the treatment of ALS (off-label use or investigational).
6. If female, breastfeeding, known to be pregnant, planning to become pregnant during the study, or of child-bearing potential and unwilling to use effective contraception, for the duration of the trial and for 3 months, or as specified in each RSA, after discontinuing study treatment.
7. If male of reproductive capacity, unwilling to use effective contraception for the duration of the trial and for 3 months, or as specified in each RSA, after discontinuing study treatment.
8. Anything that would place the participant at increased risk or preclude the participant's full compliance with or completion of the study, in the SI's opinion.
9. If a participant is being re-screened, the disqualifying condition has not been resolved, or the mandatory wash-out duration has not occurred.

If justified based on regimen's specific investigational product, additional inclusion or exclusion criteria (e.g., contraindications, laboratory parameters) will be listed in the RSA.

Riluzole. Participants taking concomitant riluzole at study entry must be on a stable dose for 30 days prior to the Master Protocol Screening Visit and must intend to continue taking the same stable dosage throughout the study.

Edaravone. Participants taking concomitant edaravone at study entry must have completed at least one cycle of treatment (typically 14 days) prior to the Master Protocol Screening Visit and must intend to continue taking the same stable dosage throughout the study.

Relyvrio/ Albrioza. Participants taking concomitant Relyvrio/ Albrioza at study entry must have started ≥ 30 days prior to the Master Protocol Screening Visit

If the SI determines that riluzole, edaravone or Relyvrio/ Albrioza should be discontinued or dose-adjusted for medical reasons, this will not be considered a protocol deviation. If extenuating circumstances cause doses to be missed for reasons not intended or medically indicated (scheduling, drug availability, etc.), this will not be considered a protocol deviation. If riluzole, edaravone or Relyvrio/ Albrioza is stopped for any non-medical reason (participant preference), this will be recorded as a minor protocol deviation. If initiation of riluzole, edaravone or Relyvrio/ Albrioza therapy begins at any point during participation, this will be recorded as a minor protocol deviation

Date of ALS Symptom Onset. For the purposes of this study, the date of symptom onset will be defined as the date the participant first had symptoms of muscle weakness. To be eligible for this study, the date of symptom onset must be no greater than 36 months prior to the Master Protocol Screening Visit date.

6 PARTICIPANT SELECTION AND ENROLLMENT

6.1 Identifying Participants

This study will be conducted at selected ALS centers in the US. Sites participating in this Master Protocol must be members of the Northeast ALS Consortium (NEALS). NEALS is a non-profit group of research institutions, each of which is a NEALS Member, who collaboratively conduct clinical research in ALS and other motor neuron diseases. Research participants will be recruited from these centers, after approval by the sIRB.

Information about all research participants enrolled into the trial will be recorded in the Electronic Data Capture (EDC) system. This information will include reasons for screen failure for participants not assigned to a regimen, as well as a log of all participants randomized within a regimen, irrespective of whether they have been treated with the intervention or not.

6.2 Consenting Participants

Written informed consent will be obtained from participants before any study procedures or assessments, not performed as clinic visit standard of care (e.g., vital signs, weight), are done and after the study objectives and endpoints, methods, anticipated benefits, and potential hazards are explained. The willingness of the participant to participate in the study will be documented in writing on a consent form approved by the sIRB, which will be signed and dated by the participant. The SI will keep the original consent forms and a copy will be given to the participant. The participant will also be informed that they may refuse entry into the trial and are free to withdraw from the trial at any time without prejudice to future treatment.

Informed consent will be obtained by the site investigator or other licensed staff who are trained on the study and authorized to obtain consent. The SI, or other licensed staff, will be involved in explaining the study and will play a role during the consent process, answering any questions in conjunction with the coordinator and ensuring that the process is carried out correctly. All participants will be offered the opportunity to discuss participation with a licensed physician Investigator and the participant’s decision to accept or decline this opportunity will be documented in the research file. Site study staff performing consent must provide participants adequate information to allow for an informed decision about participation, facilitate the potential participant's understanding of the information, and provide ample time and opportunity to inquire about details of the trial. The SI, nor the site study staff, should coerce or unduly influence a potential participant to participate or to continue to participate in the study. Site study staff must continuously provide information as the clinical investigation progresses or as the participant or situation requires.

Informed consent will be conducted for the Master Protocol and subsequently for the applicable RSA to which that participant was assigned. Those participants who are not found eligible for the Master Protocol will not be consented for any RSA.

Participants may be required to reconsent to the Master Protocol if new procedures or information is added in the future. Should a participant need to reconsent, this should occur during the participant's next in-person visit. If the participant's next in-person visit is conducted remotely, reconsent may also be completed remotely using the following procedures:

1. The site staff provides a copy of the informed consent form to the participant.
2. The participant reads through the consent form but does not sign.
3. The Site Investigator, or other study staff member approved and delegated to obtain informed consent, contacts the participant and reviews the informed consent form with the participant over the phone or via telemedicine.
4. The participant signs the informed consent form and provides the original signed consent form back to the site.

Once received at the site, the individual who consented the participant via phone or telemedicine signs the informed consent form.

6.3 Ineligible Participants

All sites will be required to collect demographic information including age, gender, race, and ethnicity, and reasons for ineligibility for the Master Protocol for participants that have signed the Master Protocol consent form and are deemed ineligible for the Master Protocol.
Once assigned to a regimen, participant eligibility will be confirmed using regimen-specific inclusion and exclusion criteria, if any, during the Regimen-Specific Screening Visit. Reasons for ineligibility for the regimen must be documented.

6.4 Randomization

6.4.1 Randomization Procedures

Randomization will be conducted using an interactive response technology (IRT) system. The randomization for the Master Protocol consists of a tiered randomization as described below and depicted in the Study Workflow.

1. Randomization equally to a regimen among all available regimens to which the participant has not previously been randomized.
2. Randomization to placebo or an active intervention in the assigned regimen, and randomization to the set of active sub-arms, if applicable.

In the first stage, after a participant is determined eligible according to the Master Protocol inclusion and exclusion criteria, a research participant will be randomized equally among all available regimens to which the participant has not previously been randomized. In the second stage, if a participant has been determined eligible by meeting all inclusion and no exclusion criteria specific to their assigned regimen, the research participant will be randomized 3:1 to the active intervention or the placebo for the regimen. If the regimen has multiple arms, a participant will also be randomized according to the allocation scheme for that regimen. The allocation scheme for regimens with multiple arms will be detailed in the respective RSA.

6.4.2 Treatment Allocation

Treatment group within a regimen will be assigned via the IRT system after a participant has been determined eligible for any inclusion and exclusion criteria specific to their assigned regimen.

6.4.3 Blinding

In this trial, investigational products may have a different mode (e.g., oral, subcutaneous) or frequency of administration and may be introduced at different time points. Each intervention arm will have its own concurrent, randomized placebo control arm.

Research participants, SIs, and everyone involved in the conduct of a given trial regimen will not be blinded to regimen assignments but will be blinded to the randomized treatment assignments within a regimen, and this blind will be maintained through the ATE period of the study, as outlined in the HEALEY ALS Platform Trial Bliniding and Unblinding Plan. In the event of a medical emergency that necessitates the unblinding of the Medical Monitor (MM) or a SI to safely provide care for a participant, emergency unblinding of that single participant may be undertaken as outlined below.

6.4.4 Emergency Unblinding Plan

Emergency unblinding for a research participant will only be undertaken when it is essential to treat the participant safely. It must only be used in an emergency when the identity of the treatment arm must be known to the SI to provide appropriate medical treatment or otherwise ensure the safety of research participants or others exposed to investigational products. In most

cases, investigational product discontinuation and knowledge of the possible treatment assignments will be sufficient to treat a study participant who presents with an emergency condition. However, if unblinding is necessary, the blind may be broken only for that participant.

6.5 Discontinuation of Treatment and Terminations

6.5.1 Discontinuation of Investigational Product

If the SI or designee is concerned about the use of a prohibited medication or other safety issues (including lack of safety data collected), then the investigational product may need to be discontinued.

If the investigational product for an RSA is administered orally, and a participant loses the ability to swallow the investigational product, the possibility of alternative modes of administration (e.g., via G-tube) will be detailed in the RSA.

A research participant may choose to discontinue the investigational product at any time for any reason. However, the SI or designee will encourage the research participant to follow the study protocol under the intent-to-treat principle (ITT). These research participants will be encouraged to follow the study visits, off treatment, up to the week 24 visit and through the ATE period of the study, following the Schedule of Activities. At a minimum, collection of the ALSFRS-R, AEs, Concomitant Medications, CSSRS, and other outcome measures should be encouraged. Loss to follow-up should be prevented whenever possible. For participants followed under the ITT principle, an in-person Early Termination Visit will be completed after they discontinue follow up under the ITT principle. A Follow-Up Safety Call at the time study drug is discontinued may not be required, as long as the participant is followed under the ITT principle for a minimum of 28 days.

Upon discontinuation of investigational product, the research participant should return any unused investigational product.

For all research participants, the reason for permanent discontinuation of investigational product must be recorded in the Case Report Form (CRF). These data will be included in the trial database and reported.

6.5.2 Termination of Individual Research Participants

A SI or designee may terminate a research participant if a medical condition or other situation occurs such that continued participation would not be in the best interest of the participant.

A research participant may withdraw consent for study participation. No justification is required for the decision.

Upon termination, the research participant should return any unused investigational product.

6.5.3 Termination of the Master Protocol or a Regimen by the Investigational New Drug (IND) Holder/ Sponsor

The IND-holder/Sponsor reserves the right to terminate the overall Master Protocol or any individual regimen at any time or during an interim analysis.

If there are regimen-specific criteria for terminating a regimen, they will be detailed in the RSA.

7 INVESTIGATIONAL PRODUCT AND PLACEBO

7.1 Investigational Product

Multiple investigational products (i.e., interventions, or active agents, from different regimen partners) will be tested in this Platform Trial.

Each investigational product will have an RSA in which the complete description of the tested product can be found. Each intervention may have multiple arms, such as different dosages or frequencies or routes of administration.

Each active agent will have a matching placebo. All regimens will be compliant with the Master Protocol, which outlines the majority of all clinical, biomarker, and safety assessments, making a shared placebo group both desirable and feasible.

7.1.1 Investigational Product Manufacturer

Details identifying the investigational product manufacturer will be included in the corresponding RSA.

7.1.2 Labeling and Packaging

Investigational product for each regimen will be provided by the regimen partner and will be described in the corresponding RSA. Packaging and labeling will follow Good Manufacturing Practices (GMP) regulations.

Samples of labels will be kept with the Trial Master File (TMF).

The participants will be instructed to return all remaining investigational product including empty package material, if applicable, to the study site. Drug accountability will be performed as described in each RSA.

Details for packaging, labeling, and re-supply will be described in the corresponding RSA.

7.1.3 Acquisition and Storage

Investigational product for all regimens will be received at the study site by designated study staff, handled and stored safely and properly at the site pharmacy or other designated location, and kept in a secure location to which only the trial pharmacist and designated pharmacy staff, SI, and clinical staff have access. Upon receipt, the investigational product will be stored according to the instructions specified on the labels. Storage conditions will be adequately

monitored and temperature in the area in which the investigational product is stored will be controlled, monitored and recorded, at a minimum daily.

In accordance with local regulatory requirements, the SI, designated site staff, or head of the medical institution (where applicable) at each site must document the amount of investigational product dispensed and/or administered to study participants, the amount received from the Central Pharmacy, and the amount destroyed upon completion of the study. An investigator is responsible for ensuring product accountability records are maintained throughout the course of the study. The research pharmacist or designated study staff will be responsible for maintaining an accurate record of the shipment and dispensing of investigational product in a drug accountability log.

Additional investigational product-specific details will be provided in the RSA.

7.1.4 Destruction of Investigational Product

Details on how the investigational product will be destroyed, who is responsible for the destruction, and how long documentation will be retained at sites, will be provided in the RSA or defer to site specific policies.

7.1.5 Investigator's Brochure

An Investigator's Brochure will be provided for each regimen as a separate document to this protocol.

7.2 Dosage Changes

Any changes in dosage, along with the circumstances surrounding those for such, will be described in the RSA.

7.3 Participant Compliance

Compliance for each regimen will be defined in the RSA. In cases of non-compliance, the participant will be reminded of the importance of taking the investigational product per protocol.

7.4 Overdose

Details on the actions to take in the event of an overdose will be included in the RSA.

7.5 Other Medications

Throughout the study, participants may be prescribed concomitant medications deemed necessary to provide adequate care. Participants should not receive other investigational products

during the study. This includes marketed agents at experimental dosages that are being tested for the treatment of ALS. All concomitant medications and significant non-drug therapies, including supplements, received by a participant should be recorded on the appropriate source document and electronic Case Report Form (eCRF).

7.5.1 Prohibited Medications

Details on medications that may not be taken during the trial will be included in the RSA.

8 STUDY SCHEDULE

This section describes trial procedures that are common to all regimens tested in the HEALEY ALS Platform Trial as detailed in the Master Protocol. Each regimen will follow these procedures and may add additional procedures as described in the corresponding RSA.

No study procedures should be performed prior to the signing of the Master Protocol ICF. All participants will sign the Master Protocol ICF prior to undergoing any study tests or procedures.

Study Visit Recommendations:

1. Visit windows are consecutive calendar days and the target visit dates are calculated from the Baseline Visit – Day 0 (first day of investigational product administration).
2. Assessment of vital capacity should be performed at the start of a visit so as not to fatigue the participant with other testing.
3. The remaining clinical assessments should be administered in the same sequence as listed in the sections below and at approximately the same time of the day.
    a. Any additional assessments that are included in an RSA for a given visit should be completed once the Master Protocol SOA for that visit is complete, unless otherwise specified in the RSA.
4. Outcomes for individual participants should be measured by the same evaluator throughout their participation in a regimen. Evaluators must be qualified, trained, and must fulfill certification criteria defined by the Sponsor. Training documentation must be filed in the Investigator Site File (ISF).
5. In-person visits may be conducted in-clinic, or by phone or telemedicine, or in-home.

If needed to protect the safety of the participant due to a pandemic, disability or other medical reason, designated visits in the Schedule of Activities (i.e. Week 4, Week 8, and Week 16) may be conducted via telemedicine (or phone if telemedicine is not available) with remote services instead of in-person. If a planned in-clinic visit is conducted via telemedicine (or phone if telemedicine is not available) with remote services, only selected procedures will be performed. Instructions on how to document missed procedures are included in the study Manual of Procedures (MOP). The following procedures should be completed in the suggested order and as per the Schedule of Activities: ALSFRS-R,AE Review, Columbia-Suicide Severity Rating Scale, Concomitant Medications, and IP Accountability/Compliance; each RSA may describe additional activities. Procedures for collecting IP Accountability/Compliance remotely are described in the study MOPs. Additionally, selected vital signs and laboratory evaluations may be performed by a home health agency as described in the study MOP. Vital signs include: systolic and diastolic pressure, respiratory rate, heart rate, and temperature. Laboratory evaluation includes hematology (CBC with differential), coagulation parameters, complete

chemistry panel and thyroid function, and urinalysis. Pregnancy testing is only repeated as applicable if there is a concern for pregnancy.

Vital Capacity: the default and preferred method for assessing Vital Capacity within the Master Protocol Screening period for eligibility is SVC performed by NEALS certified evaluators using a study-approved portable spirometer. If needed to protect the safety of the participants and/or site staff due to pandemic-related restrictions, Vital Capacity can be measured by a Pulmonary Function Laboratory evaluator to assess eligibility. At all subsequent visits to Master Protocol Screening, if SVC cannot be obtained due to to a pandemic, disability or other medical reason, this outcome measure should be skipped, recorded as such in the applicable source documentation, and a protocol deviation reported.

Clinical Safety Labs: the default and preferred method for collecting clinical safety labs is by using the study specific central laboratory as described in the study MOP. If needed to protect the safety of the participant due to a pandemic, disability or other medical reason, clinical safety labs can be collected using one of the following methods:

- Home health agency as described in the study MOP, or
- Local site laboratory resources, or
- Independent labs offering such services as described in this protocol and/or local to the participant (e.g. primary care physician)

8.1 Master Protocol Screening Visit

The following procedures will be performed in-person, after Master Protocol informed consent is obtained, to determine the participant's eligibility for the Master Protocol and are listed in preferred order to be completed:

Assess Master Protocol inclusion and exclusion criteria
Obtain ALS and medical history
Obtain demographics
Perform Vital Capacity
Perform physical examination
Perform neurological examination
Measure vital signs including height and weight
Administer ALSFRS-R questionnaire
Perform 12-lead electrocardiogram (ECG)
Collect blood samples for clinical screening assessments (including coagulation parameters) and, for women of child bearing potential (WOCBP), for pregnancy test
Collect urine sample for urinalysis
Review and document concomitant medications

Schedule the Regimen Specific Screening Visit if the participant passes screening for the Master Protocol

8.1.1 Regimen Assignment and Within-regimen Randomiation

If the participant meets all Master Protocol eligibility criteria, the participant will be randomly assigned to a regimen and will undergo informed consent for that regimen at their scheduled Regimen-Specific Screening Visit. If eligible, the participant will then be randomized in a 3:1 ratio to active treatment or matching placebo for that regimen. If the intervention has multiple arms (for example, multiple dosages), a research participant will also be randomized according to the allocation scheme specific for that regimen.

8.1.2 Screen Failures and Re-Assignment

Any participant who signs the Master Protocol consent form will be considered enrolled in the Platform Trial. If a participant fails the Master Protocol screening, *at a minimum* the following information should be captured and entered into the EDC system:

- Demographics (including the participant's NeuroSTAMP)
- Reason for screen failure
- Any eligibility criteria that were assessed prior to when the research participant failed to meet an inclusion criterion or met an exclusion criterion.

If a research participant fails to meet a time-dependent Master Protocol eligibility criterion (e.g., duration of stable medication prior to enrollment, interval following last use of an investigational treatment, or other criteria as applicable), then that participant will be considered to have screen failed, and may be re-screened for Master Protocol eligibility immediately after resolution of the disqualifying condition. Safety labs required for the site investigator to determine eligibility, can be repeated during the Master Protocol screening period, if necessary. If a participant is re-screened for Master Protocol eligibility, they must complete a full Master Protocol Screening Visit as outlined in section 8.1.

If a research participant meets Master Protocol eligibility, is assigned to a specific regimen, but does not meet additional regimen-specific inclusion and exclusion criteria for that regimen, then the research participant may be re-assigned immediately to a different regimen.

If a research participant meets Master Protocol eligibility, is assigned to a regimen, and is randomized within that regimen, but their participation in that regimen is discontinued, that participant may have the opportunity to be re-screened for the Master Protocol. The research participant must provide written informed re-consent to the Master Protocol and must still meet the Master Protocol inclusion and exclusion eligibility criteria in order to be re-assigned into a different regimen. Re-screening for the Master Protocol and potential re-assignment into a different regimen, if available, may occur due to the following situations:

- A regimen is stopped due to futility or success
  - The research participant may be re-screened after 30 days or 5 half-lives (if known), whichever is longer
- A research participant completes their Week 24 Visit in the placebo-controlled period and terminates their participation in the study
  - The research participant may be re-screened after 30 days or 5 half-lives (if known), whichever is longer
- A research participant discontinues participation in a regimen for personal reasons (e.g., inconvenience of the intervention, mode of administration, required assessments) or due to an adverse event, or is discontinued by the SI
  - The research participant may not be re-screened until after the date that their 24-Week Visit would have occurred, had they completed participation in that regimen.

8.2 Regimen Specific Screening Visit

This visit will take place in-person after random assignment to a regimen. This visit may be combined with the Baseline Visit, please refer to the RSA for applicability. The following procedures will be performed and are listed in preferred order to be completed:

Obtain written regimen-specific informed consent from participant
Assess regimen-specific inclusion and exclusion criteria
Concomitant medication review
Assess and document AEs

8.3 Baseline Visit

This visit will take place in-person after the regimen-specific Screening Visit. This visit may be combined with the Regimen Specific Screening Visit, please refer to the RSA for applicability. The following procedures will be performed and are listed in preferred order to be completed:

Randomize within the regimen
Collect vital signs
Perform SVC
Perform muscle strength testing
Administer ALSFRS-R questionnaire
Administer Patient Reported Outcomes
Collect blood samples for Clinical Safety Labs, biomarkers, and optional DNA sequencing
Collect urine sample for biomarkers
Perform LP for CSF collection (as described in the RSA)
Review and document concomitant medications
Assess and document AEs

Administer the C-SSRS Baseline questionnaire
Administer first dose of investigational product for the regimen, per the instructions in the RSA.
Dispense investigational product to participant, per instructions in the RSA

8.4 Week 2 Telephone Visit

This visit will take place 14 ± 3 days after the Baseline Visit via telephone. The following procedures will be performed and documented and are listed in preferred order to be completed:

Assess and document AEs
Drug Compliance Check-In
Remind participant to bring in investigational product to the Week 4 Visit

8.5 Week 4 Visit

This visit will take place in-person 28 ± 7 days after the Baseline Visit. The following procedures will be performed and are listed in the preferred order to be completed:

Collect vital signs
Administer ALSFRS-R questionnaire
Collect blood samples for Clinical Safety Labs and, for WOCBP, for pregnancy test if applicable
Review and document concomitant medications
Assess and document AEs
Administer the C-SSRS Since Last Visit questionnaire
Collect blood for optional DNA sequencing (if not done at Baseline)
Perform investigational product accountability and compliance
Schedule next Study Visit
Remind participant to bring in investigational product to the Week 8 Visit

8.6 Week 8 Visit

This visit will take place in-person 56 ± 7 days after the Baseline Visit. The following procedures will be performed and are listed in the preferred order to be completed:

Collect vital signs including weight
Perform SVC
Perform muscle strength testing
Administer ALSFRS-R questionnaire
Ask participant to complete Patient Reported Outcomes
Collect blood samples for Clinical Safety Labs and, for WOCBP, for pregnancy test if applicable
Collect urine sample biomarker analyses

Collect blood sample for biomarker analyses
Collect blood for optional DNA sequencing (if not done previously)
Review and document concomitant medications
Assess and document AEs
Administer the C-SSRS Since Last Visit questionnaire
Perform investigational product accountability and compliance

8.7 Week 12 Telephone Visit

This visit will take place 84 ± 3 days after the Baseline Visit via telephone. The following procedures will be performed and are listed in the preferred order to be completed:

Administer ALSFRS-R questionnaire
Review and document concomitant medications
Assess and document AEs
Drug Compliance Check-In
Schedule next Study Visit
Remind participant to bring in investigational product to the Week 16 Visit

8.8 Week 16 Visit

This visit will take place in-person 112 ± 7 days after the Baseline Visit. The following procedures will be performed and are listed in the preferred order to be completed:

Collect vital signs including weight
Perform SVC
Perform muscle strength testing
Administer ALSFRS-R questionnaire
Ask participant to complete Patient Reported Outcomes
Collect blood samples for Clinical Safety Labs (including coagulation parameters) and, for WOCBP, for pregnancy test if applicable
Collect urine sample biomarker analyses
Collect blood sample for biomarker analyses
Collect blood for optional DNA sequencing (if not done previously)
Review and document concomitant medications
Assess and document AEs
Administer the C-SSRS Since Last Visit questionnaire
Perform LP for CSF collection (as described in the RSA)
Perform investigational product accountability and compliance

8.9 Week 20 Telephone Visit

This visit will take place 140 ± 3 days after the Baseline Visit via telephone. The following procedures will be performed and are listed in preferred order to be completed:

Administer ALSFRS-R questionnaire
Review and document concomitant medications
Assess and document AEs
Drug Compliance Check-In
Schedule next Study Visit

8.10 Week 24 Visit

This visit will take place in-person 168 ± 7 days after the Baseline Visit. The following procedures will be performed and are listed in preferred order to be completed:

Collect vital signs including weight
Perform SVC
Perform muscle strength testing
Administer ALSFRS-R questionnaire
Ask participant to complete Patient Reported Outcomes
Perform 12-lead electrocardiogram (ECG)
Collect blood samples for Clinical Safety Labs and, for WOCBP, for pregnancy test if applicable
Collect urine sample biomarker analyses
Collect blood sample for biomarker analyses
Collect blood for optional DNA sequencing (if not done previously)
Review and document concomitant medications,
Assess and document AEs
Administer the C-SSRS Since Last Visit questionnaire
Perform investigational product accountability and compliance
Ask participant to complete the Exit Questionnaire

8.11 Follow-Up Safety Call

Participants will have a Follow-Up Safety after their last dose of study drug. Additional details, including the timing of this call, can be found in the RSA. The following procedure will be performed at a minimum:

- Assess and document AEs
- Review and document concomitant medications

8.12 Early Termination Visit & Follow-up Safety Call for Early Terminations

Participants who terminate their participation in the study early will be asked to be seen for an in-person Early Termination Visit and complete a Follow-Up Safety Call.

The outcome measures that are required to be collected for each regimen, and the timing of and activities for the Early Termination Visit and Follow-Up Safety Call, will be detailed in the RSA.

8.13 Protocol Deviations

A protocol deviation is any noncompliance with the sIRB approved clinical trial protocol. The noncompliance may be either on the part of the participant, the SI, or the study site staff. Patient reported outcomes (e.g. ALSAQ-40 and CNS-BFS) not performed due a participant language barrier, and procedures that were attempted but failed will not be reported as protocol deviations. A minor protocol deviation will be considered a departure from the protocol of relatively minor degree (such as study visit a day or two out of window due to participant being sick). A major deviation will be considered any significant deviation from the protocol that the Sponsor determines negatively affect: (1) the rights or welfare of the participant; (2), risk benefit assessment; or (3) the integrity of the data (ability to draw valid conclusions from the study data). As a result of deviations, corrective and preventative actions are to be developed by the site with review by the CC and Study Monitors and implemented promptly.

All deviations from the protocol will be recorded in the participant's source documentation. Protocol deviations must be entered in the Protocol Deviations Log in the EDC system. Deviations will be reported to the sIRB per their guidelines.

8.14 Missed Visits and Procedures

Missed visits and any procedures not performed (not attempted) for reasons other than illness, injury or progressive disability (i.e., participant is physically unable to perform test) will be reported as protocol deviations.

Procedures or visits not performed due to illness, injury or disability, including procedures that were attempted but failed (e.g., blood samples unable to be drawn after multiple attempts, or weight unable to be obtained due to participant immobility) will not be reported as protocol deviations.

Investigational product compliance that is outside the limits set in the RSA will be reported as a protocol deviation (see RSA for further details).

Details and specific instructions regarding protocol deviations, including any exceptions to this standard procedure, are found in the study Manual of Procedures.

Missed assessments due to pandemic-related restrictions must be documented as such in the source documentation and in the EDC.

8.15 Recording Deaths and Vital Status Determination

Information on whether a participant has died may be obtained from the participant's family, from clinic notes, or from a publicly available data source like the Centers for Disease Control and Prevention (CDC) National Death Index or the Social Security Death Index.

Vital status, defined as a determination of date of death or PAV status or date last known alive and date last known to be PAV-free, will be determined by site study staff for each randomized participant throughout the placebo-controlled portion of their follow-up (generally the Week 24 visit, as indicated in the SOA). At approximately the time of the last patient last visit (LPLV) of the placebo-controlled period of a given regimen, a second vital status check will be completed by site study staff for each randomized participant. When prompted by the Coordination Center, sites will contact all randomized participants to assess vital status.

We may also ascertain vital status at later time points. An outside vendor will be used to ascertain death or date of last known alive for all randomized participants by using publicly available data sources. When prompted by the Coordination Center, sites will provide demographic information (e.g. participant name, date of birth, last known address) to the vendor using a secure method.

9 CLINICAL ASSESSMENTS AND OUTCOME MEASURES

9.1 Clinical Variables

Assessments will be performed at designated time-points throughout the study for clinical evaluation. In addition to the assessments evaluated below, participants will provide information on their demographics, past medical history, including ALS, as well as concomitant medication usage.

Details of any additional specific clinical assessments required for a regimen will be reported in the corresponding RSA.

9.1.1 Vital Signs and Anthropometrics

Vital signs include weight in kg or lbs, systolic and diastolic blood pressure in mmHg, pulse rate (radial artery)/minute, respiratory rate/minute, and temperature in °C or °F. **Height in cm or in will be measured and recorded at the Master Protocol Screening Visit (Visit 1) only.** This one-time height measurement must be used throughout the duration of the study.

9.1.2 Clinical Safety Laboratory Tests

The following laboratory tests will be performed for safety at the Master Protocol Screening to determine eligibility and at the following visits: Screening/Baseline as described in the RSA, Week 4, Week 8, Week 16, and Week 24/ET

- Hematology with differential panel: complete blood count with differential (hematocrit, hemoglobin, platelet count, RBC indices, Total RBC, Total WBC, and WBC & differential)
- Blood chemistry panel/Liver function tests (LFTs): alanine aminotransferase (ALT (SGPT)), aspartate aminotransferase (AST (SGOT)), albumin, alkaline phosphatase, bicarbonate, blood urea nitrogen, calcium, chloride, creatinine (estimated Glomerular Filtration Rate [eGFR] will be calculated using the MDRD equation and creatinine clearance will be calculated using the Cockcroft-Gault equation), glucose, magnesium, phosphate, potassium, sodium, thyroid-stimulating hormone (TSH), total bilirubin and total protein
- Urinalysis: albumin, bilirubin, blood, clarity, color, glucose, ketones, nitrate, pH, protein, specific gravity, urobilinogen and WBC screen
- Serum human chorionic gonadotrophin (hCG) for WOCBP (collected only at Master Protocol Screening Visit, and as necessary throughout course of study)

Additionally, coagulation parameters (Prothrombin time, International normalized ratio, Activated partial thromboplastin time) will be collected for all participants at the Master Protocol Screening and Week 16 Visits.

All participants will have clinical safety laboratory tests at the designated visits outlined in the schedule of activities. All laboratory samples will be analyzed at a central laboratory, unless needed to protect the safety of the participant due to a pandemic, disability or other medical reason, they are collected by a home health agency, local site laboratory or independent laboratory. The SI may order additional local testing or a retest at the central laboratory, if needed, to further assess an AE, or if there is any suspicion that a participant may be pregnant, or if a result could not be obtained throughout the course of the study. If additional testing is required to obtain results, the participant may be asked to return to repeat blood collection for such missing results.

9.1.3 12-Lead ECG

A standard 12-lead ECG will be performed for safety at the screening visit to determine eligibility and for safety as outlined in the schedule of activities. Tracings will be reviewed by a central ECG reader and a copy of the tracings will be kept on site as part of the source documents.

9.1.4 Physical Examination

A physical examination will be performed and recorded. The following systems will be examined: head/neck, eyes, ears, nose and throat, cardiovascular, lungs, abdomen, musculoskeletal, central nervous system, extremities, and skin.

9.1.5 Neurological Examination

A neurological examination will be performed and recorded. Examination will include assessment of mental status, motor and sensory function, reflexes, and coordination/cerebellar function.

9.1.6 Columbia-Suicide Severity Rating Scale

The US FDA recommends the use of a suicidality assessment instrument that maps to the Columbia Classification Algorithm for Suicide Assessment (C-CASA). The C-CASA was developed to assist the FDA in coding suicidality data accumulated during the conduct of clinical trials of antidepressant drugs. One such assessment instrument is the C-SSRS. The C-SSRS involves a series of probing questions to inquire about possible suicidal thinking and behavior.

At the Baseline Visit, the C-SSRS Baseline version will be administered. This version is used to assess suicidality over the participant's lifetime.

At all other visits, the Since Last Visit version of the C-SSRS will be administered. This version of the scale assesses suicidality since the participant's last visit.

If there is a positive response to question 4 or 5 on the severity of ideation subscale or any positive response on the suicidal behavior subscale of suicide attempt or suicidal ideation by the participant during the administration of the C-SSRS during the treatment period, the appropriately qualified clinician will be notified during the study visit to determine the appropriate actions required to ensure the participant's safety. The site must ensure that the participant is seen by a licensed physician (or other qualified individual as required by local institutional policy) before leaving the study site. The SI will determine whether the participant should remain on study drug. Reference to the Clinical Triage Guidelines Using the C-SSRS can be found here https://cssrs.columbia.edu/wp-content/uploads/C-SSRStriageexampleguidelines.pdf.

It is recommended that a medically licensed physician, nurse, nurse practitioner, or physician assistant to assess the C-SSRS. All evaluators must be certified to perform the C-SSRS; specific certification requirements are outlined in the study operations manual. Certification is required prior to performing the C-SSRS.

9.2 Endpoints

Details of any additional specific endpoints required for a regimen will be described in the corresponding RSA.

9.2.1 ALS Functional Rating Scale - Revised

The ALSFRS-R is a quickly administered (5 minutes) ordinal rating scale used to determine participants' assessment of their capability and independence in 12 functional activities. Each functional activity is rated 0-4 for a total score that ranges from 0 to 48. Higher scores indicate better function. Initial validity in ALS patients was established by documenting that, change in ALSFRS-R scores correlated with change in strength over time, was closely associated with quality of life measures, and predicted survival. The test-retest reliability is greater than 0.88 for all test items. The advantages of the ALSFRS-R are that all 12 functional activities are relevant to ALS, it is a sensitive and reliable tool for assessing activities of daily living function in those with ALS, and it is quickly administered. With appropriate training the ALSFRS-R can be administered with high inter-rater reliability and test-retest reliability. The ALSFRS-R can be administered by phone with good inter-rater and test-retest reliability. The equivalency of phone versus in-person testing, and the equivalency of study participant versus caregiver responses have also been established. Additionally, the ALSFRS-R can also be obtained using a web-based

interface with good concordance with in-person assessment. All ALSFRS-R evaluators must be NEALS certified.

9.2.2 Slow Vital Capacity

The vital capacity (VC) will be determined using the upright slow VC (SVC) method. All evaluators performing SVC, must be NEALS certified. The SVC will be measured using the study-approved portable spirometer, and assessments will be performed using a face mask. A printout from the spirometer of all VC trials will be retained. Three VC trials are required for each testing session, however up to 5 trials may be performed if the variability between the highest and second highest VC is 10% or greater for the first 3 trials. Only the 3 best trials are recorded on the CRF. The highest VC recorded is utilized for eligibility. At least 3 measurable VC trials must be completed to score VC for all visits after screening. Predicted VC values and percent-predicted VC values will be calculated using the Quanjer Global Lung Initiative equations.

9.2.3 Measures of Muscle Strength

Hand Held Dynamometry: HHD will be used as a quantitative measure of muscle strength for this study. Six proximal muscle groups will be examined bilaterally in both upper and lower extremities (shoulder flexion, elbow flexion, elbow extension, hip flexion, knee flexion, and knee extension), all of which have been validated against maximum voluntary isometric contraction (MVIC) testing[19]. In addition, wrist extension, abductor pollicis brevis, abductor digiti minimi, first dorsal interosseous contraction and ankle dorsiflexion will be measured bilaterally; these muscles are often affected in ALS.

Bilateral Hand Grip: Bilateral hand grip will be measured using a Jamar hand dynamometer to test the maximum isometric strength of the hand and forearm muscles, measured in pounds.

9.2.4 Training and Validation

All evaluators must be NEALS trained and certified to perform the ALSFRS-R, SVC, and HHD assessments; specific certification requirements are outlined in the study MOP. Certification and training will be provided by the Barrow Outcome Center. Certification occurs via a formal evaluation of reliability and accuracy of performance of these measures. Repeat certification will be required on an annual basis for ALSFRS-R and every two years for SVC and HHD outcome measures. It is strongly preferred that a single evaluator performs all measures throughout the study, as much as possible.

9.2.5 Patient Reported Outcomes

Patient reported outcomes will be assessed in this trial and will be defined in the RSA.

9.3 Biofluid Collection

All samples will be labeled with a code. The code will not include any identifiable information but will be linked to a specific participant, visit, and sample by data entry of the code into the EDC. There is no scheduled date on which the samples will be destroyed. Samples may be stored for research until they are used, damaged, decayed or otherwise unfit for analysis. Participants have the option of declining participation in this portion of the study at any time by withdrawing their consent to have their sample used. However, it will not be possible to destroy samples that may have already been used.

Specific instructions regarding the collection, processing, storage, and shipment of these samples will be provided in the study Laboratory Manual.

Details of any additional specific biofluid samples required for a regimen will be described in the corresponding RSA.

9.3.1 DNA Collection

All participants consenting to DNA collection will provide a blood sample for deoxyribonucleic acid (DNA) extraction for genome sequencing during the Baseline Visit. The DNA sample can be collected after the Baseline Visit if a baseline sample is not obtained for any reason or the sample is not usable.

9.3.2 Blood Biomarker Sample Collection

All participants will provide blood samples for biomarker assessments.

9.3.3 Urine Collection

Urine samples will be collected at Baseline, Week 8, Week 16 and Week 24 study visits. Up to 10mL of urine may be collected.

9.3.4 Lumbar Puncture

The lumber puncture (LP) procedure will be performed to collect CSF, as described in each RSA.

Study staff should document the time of study drug dose administered.
Coagulation parameters (Prothrombin time, International normalized ratio, Activated partial thromboplastin time) will be collected for all participants at the Master Protocol Screening and Week 16 Visits.
The SI will discuss all potential LP risks to the subjects including:

- Local pain at injection site
- Reaction to anesthetic agents

- o Bleeding at needle entrance site
- o Infection at needle entrance site
- o Post-LP low-pressure headache

Extensive experience with research LP in Alzheimer's disease reveals a very low incidence of complication, including the incidence of post-LP headache (Zetterberg et al., 2010). Fewer than 2.6% of patients in a memory disorder clinic developed post-LP headache, and only a single patient in a cohort of over 1,000 had a headache lasting more than 5 days (Zetterberg et al., 2010). No other local or generalized complications occurred.

The procedure must be performed by the SI or another licensed practitioner with experience and training in performing LPs,. When possible, LPs should be performed with an atraumatic Sprotte needle to reduce the risk of post-LP headache.

10 SAFETY AND ADVERSE EVENTS

The AE definitions and reporting procedures provided in this protocol comply with all applicable United States Food and Drug Administration (FDA) regulations and ICH guidelines. The SI will carefully monitor each participant throughout the study for possible adverse events. All AEs will be documented on CRFs designed specifically for this purpose. It is also important to report all AEs, especially those that result in permanent discontinuation of the investigational product being studied, whether serious or non-serious.

10.1 Definitions of AEs, Suspected Adverse Drug Reactions & SAEs

10.1.1 Adverse Event and Suspected Adverse Drug Reactions

An AE is any unfavorable and unintended sign (including a clinically significant abnormal laboratory finding, for example), symptom, or disease temporally associated with a study, use of a drug product or device whether or not considered related to the drug product or device.

Adverse drug reactions (ADR) are all noxious and unintended responses to a medicinal product related to any dose. The phrase "responses to a medicinal product" means that a causal relationship between a medicinal product and an adverse event is at least a reasonable possibility, i.e., the relationship cannot be ruled out. Therefore, a subset of AEs can be classified as suspected ADRs, if there is a causal relationship to the medicinal product.

Examples of adverse events include: new conditions, worsening of pre-existing conditions, clinically significant abnormal physical examination signs (e.g., skin rash, peripheral edema, etc.), or clinically significant abnormal test results (e.g., lab values or vital signs), with the exception of outcome measure results, which are not being recorded as adverse events in this trial (they are being collected, but analyzed separately). Stable chronic conditions (e.g., diabetes, arthritis) that are present prior to the start of the study and do not worsen during the trial are NOT considered adverse events. Chronic conditions that occur more frequently (for intermittent conditions) or with greater severity, would be considered as worsened and therefore would be recorded as adverse events.

Adverse events are generally detected in two ways:

1. Clinical: symptoms reported by the participant or signs detected on examination.
2. Ancillary Tests: abnormalities of vital signs, laboratory tests, and other diagnostic procedures (other than the outcome measures, the results of which are not being captured as AEs).

For the purposes of this study, symptoms of progression/worsening of ALS, including 'normal' progression, will be recorded as adverse events.

The following measures of disease progression will not be recorded as adverse events even if they worsen (they are being recorded and analyzed separately): SVC results, ALSFRS-R results, and muscle strength results.

If discernible at the time of completing the AE log, a specific disease or syndrome rather than individual associated signs and symptoms should be identified by the SI and recorded on the AE log. However, if an observed or reported sign, symptom, or clinically significant laboratory anomaly is not considered by the SI to be a component of a specific disease or syndrome, then it should be recorded as a separate AE on the AE log. Clinically significant laboratory abnormalities, such as those that require intervention, are those that are identified as such by the SI.

Participants will be monitored for adverse events from the time they sign consent for the Master Protocol until completion of their participation as defined in the RSA.

An unexpected adverse event is any adverse event, the specificity or severity of which is not consistent with the current regimen applicable Investigator's Brochure. An unexpected, suspected adverse drug reaction is any unexpected adverse event that for which, in the opinion of the SI or Sponsor, there is a reasonable possibility that the investigational product caused the event.

10.1.2 Serious Adverse Events

A SAE is defined as an adverse event that meets any of the following criteria:

1. Results in death.
2. Is life threatening: that is, poses an immediate risk of death as the event occurred.
    a. This serious criterion applies if the study participant, in the view of the SI or Sponsor, is at immediate risk of death from the AE <u>as it occurs</u>. It does not apply if an AE that might hypothetically have caused death if it were more severe.
3. Requires inpatient hospitalization or prolongation of existing hospitalization.
    a. Hospitalization for an elective procedure (including elective PEG tube/g-tube/feeding tube placement) or a routinely scheduled treatment is not an SAE by this criterion because an elective or scheduled "procedure" or a "treatment" is not an untoward medical occurrence.
4. Results in persistent or significant disability or incapacity.
    a. This criterion applies if the "disability" caused by the reported AE results in a substantial disruption of the participant's ability to carry out normal life functions.
5. Results in congenital anomaly or birth defect in the offspring of the participant (whether the participant is male or female).

6. Necessitates medical or surgical intervention to preclude permanent impairment of a body function or permanent damage to a body structure.
7. Important medical events that may not result in death, are not life-threatening, or do not require hospitalization may also be considered SAEs when, based upon appropriate medical judgment, they may jeopardize the participant and may require medical or surgical intervention to prevent one of the outcomes listed in this definition. Examples of such medical events include blood dyscrasias or convulsions that do not result in inpatient hospitalization, or the development of drug dependency or drug abuse.

An inpatient hospital admission in the absence of a precipitating, treatment-emergent, clinical adverse event may meet criteria for "seriousness" but is not an adverse experience, and will therefore, not be considered an SAE. An example of this would include a social admission (participant admitted for other reasons than medical, e.g., lives far from the hospital, has no place to sleep).

A serious, suspected adverse drug reaction is an SAE that, in the opinion of the SI or Sponsor, there is a reasonable possibility that the investigational product caused the event.

The SI is responsible for classifying adverse events as serious or non-serious.

10.2 Assessment and Recording of Adverse Events

The SI will carefully monitor each participant throughout the study for possible AEs. All AEs will be documented on source and CRFs designed specifically for this purpose. All AEs will be collected and reported in the EDC system and compiled into reports for monthly reviewing by the MM for the Master Protocol. The Master Protocol MM shall promptly review all information relevant to the safety of the investigational product, including all SAEs. Special attention will be paid to those that result in permanent discontinuation of the investigational product(s) being studied, whether serious or non-serious.

10.2.1 Assessment of Adverse Events

At each visit (including telephone interviews), the participant will be asked if they have had any problems or symptoms since their last visit in order to determine the occurrence of adverse events. If the participant reports an adverse event, site staff will probe further to determine:
1. Type of event
2. Date of onset and resolution (duration)
3. Severity (mild, moderate, severe)
4. Seriousness (does the event meet the above definition for an SAE)
5. Causality, relation to investigational product and disease

6. Action taken regarding investigational product
7. Outcome

Severity of Event
The following guidelines will be used to describe severity.

- **Mild** – Events require minimal or no treatment and do not interfere with the participant's daily activities.
- **Moderate** – Events result in a low level of inconvenience or concern with the therapeutic measures. Moderate events may cause some interference with functioning.
- **Severe** – Events interrupt a participant's usual daily activity and may require systemic drug therapy or other treatment. Severe events are usually potentially life-threatening or incapacitating. Of note, the term "severe" does not necessarily equate to "serious".

10.2.2 Relatedness of Adverse Event to Investigational Product

The relationship of the AE to the investigational product should be specified by the SI based on temporal relationship and his/her clinical judgment, using the following definitions:

- **Related** – The AE is known to occur with the study intervention, there is a reasonable possibility that the study intervention caused the AE, or there is a temporal relationship between the study intervention and event. Reasonable possibility means that there is evidence to suggest a causal relationship between the study intervention and the AE.
- **Not Related** – There is not a reasonable possibility that the administration of the study intervention caused the event, there is no temporal relationship between the study intervention and event onset, or an alternate etiology has been established.

10.2.3 Recording of Adverse Events

All clinical Adverse Events and Key Study Events (e.g., Mortality, Pregnancy, PAV, and Tracheostomy) are recorded in the participant's records. The site should enter the AE and Key Study Event information into the EDC system as soon as possible after learning of the event or receiving an update on an existing event.

Entries on the AE Log (and into the EDC system) will include the following: description of the event, severity, seriousness, date of onset, date of resolution, relationship to investigational product, action taken, and primary outcome of event.

10.3 Adverse Events and Serious Adverse Events - Reportable Events

The following are considered reportable events and must be reported to the CC within 24 hours of the site being notified of the event: all events that meet the above criteria for SAEs, or any

unexpected AE that is deemed related to the study drug, which would have implications for the conduct of the study (e.g., requiring a significant and usually safety-related change to the protocol such as revising inclusion/exclusion criteria or including a new monitoring requirement, informed consent or investigator's brochure)

The timelines for reporting dosage changes are described in each RSA as applicable.

The MM also reviews AE reports, compiled by Data Management, as described in the safety monitoring plan. The MM will review blinded study data on enrollment, abnormal laboratory results and protocol deviations. These reports will collectively be known as the Medical Monitor Report.

The MM communicates with the IND Holder / Sponsor, the DSMB, and the CC as needed for reporting of SAEs to the FDA within the required timeframe per FDA investigational new drug application (IND) regulations.

All AEs that meet the criteria for a serious, unexpected, suspected adverse drug reaction (SUSAR), for which there is a reasonable possibility that the investigational product caused the event, in the opinion of the IND Holder / Sponsor, will be submitted to FDA in an expedited fashion.

Death, respiratory failure and hospitalization for routine procedures (i.e., g-tube placement) will not be reported individually in an expedited manner because they are anticipated to occur in the study population at some frequency independent of drug expose. The DSMB will review aggregate analysis of these events and the sponsor will submit an IND safety report if the aggregate analysis indicates these events occur more frequently in any of the active drug treatment groups, as compared to the concurrent placebo group.

11 STATISTICAL CONSIDERATIONS

The statistical considerations specified here describe the overall structure and statistical design of the platform trial and propose recommended statistical details for evaluating individual regimens. Additional details regarding the master protocol recommended design are specified in Appendix I (ALS Master Protocol Recommended Design and Simulation Report) to the Master Protocol Statistical Analysis Plan (M-SAP). Regimen-specific deviations from the Master Protocol recommended statistical design are allowed and full statistical details are provided in separate regimen specific statistical analysis plans (R-SAPs) for each regimen. In the case of any deviation between this protocol and Appendix I of the M-SAP, Appendix I of the M-SAP takes precedence. In case of any deviation with an R-SAP, the order of precedence is specified in the R-SAP.

Interim analyses will be performed by a distinct team of unblinded statisticians. All other analyses will be performed either on blinded data or after database lock for a given regimen.

11.1 Statistical Design Overview

The primary aim of the trial is to compare the effectiveness of novel/emerging therapies to placebo for participants with ALS. The master protocol will include multiple regimens where a regimen is defined as both an active treatment and the corresponding randomized placebo.

The recommended primary effecicay endpoint is the change from baseline through week 24 in disease severity as measured by the ALS Functional Rating Scale-Revised (ALSFRS-R) total score and survival. The primary analysis is a Bayesian shared parameter analysis of ALSFRS-R and survival that compares each regimen-specific active treatment to a shared placebo group that is common across all regimens, as specified in Appendix I of the M-SAP, Section 2.2 (ALS Master Protocol Recommended Design and Simulation Report Index). The primary survival endpoint is the composite event of death or a death equivalent of progression to PAV. PAV is defined as use of noninvasive or invasive mechanical ventilation for more than 22 hours per day for more than seven consecutive days. The date of PAV initiation is the first day of the consecutive days.

Participants in the master protocol will be randomized in two stages; first to one of the actively enrolling regimens after the participant is determined eligible based on Master Protocol criteria; and then 3:1 to active treatment or placebo within the regimen after the participant is determined eligible based on any regimen-specific eligibility criteria. The master protocol recommended design is 160 participants randomized within each regimen.

New regimens are launched and added in parallel to existing regimens already being investigated in the platform. Regimens exit the platform either after completing all planned accrual and follow-up or by meeting criteria for success (if applicable) or futility at pre-defined interim analyses. The recommended early stopping criteria for success (if applicable) and futility are defined in Appendix I of the M-SAP. Early stopping criteria are non-binding.

Early stopping criteria will be evaluated for active regimens that have sufficient data, defined as having at least 40 randomized participants within that regimen who have had the opportunity to complete at least 24 weeks of follow-up. Interim analyses will occur globally across the platform every 12 weeks. If a regimen is not stopped early, then the regimen-specific final analysis will take place when all its randomized participants have had the opportunity to complete 24-week-placebo-controlled follow-up.

11.2 Analysis Plan

11.2.1 Primary Analysis Population and Shared Control

The primary analysis dataset uses all available longitudinal data (ALSFRS-R total score assessments at 0, 4, 8, 12, 16, 20 and 24 weeks,and assessments during the placebo-controlled follow up period that substitute for missed scheduled visits, including early termination visits) as well as survival within 24 weeks for participants within the primary analysis population.

The primary analysis population for each analysis regimen is based on the intent to treat (ITT) principle and includes all participants randomized to the active treatment arm within the analysis regimen, all participants concurrently randomized to the control arm within the analysis regimen, and control participants from all contributing regimens. The shared control group includes all participants concurrently randomized to the control arm within the analysis regimen, participants randomized to the control arm across other actively enrolling regimens (concurrent shared controls) and participants randomized to the control arm in previously completed regimens (non-concurrent shared controls). Concurrent shared controls are defined as participants randomized to placebo control who were randomized within a 6-month (defined as 180 days) window of the first and last randomization within the analysis regimen.

The Master Protocol allows for additional and more restrictive eligibility criteria specified in a regimen specific appendix (RSA). These additional eligibility criteria will be limited and will be allowed only to ensure participant safety or based on mechanism of action. Major additional regimen-specific eligibility criteria are those that have the potential to lead to significant differences in the patient population with respect to the rate of disease progression. If these criteria can be assessed for all participants entering the platform, then they may be used to subset the shared control to include only those participants that meet these additional regimen-specific

eligibility criteria in the primary analysis. If a subset of the shared control will be used in the primary analysis due to major additional regimen-specific eligibility criteria, this will be pre-specified within each RSA.

11.2.2 Primary Efficacy Analysis

Unless otherwise specified in the RSA or R-SAP, the primary analysis for each analysis regimen is a Bayesian shared parameter model of function and survival. The functional component is a repeated measures ALSFRS-R model that measures the slowing in the mean rate of progression for ALSFRS-R in active treatment participants relative to control participants and uses all available longitudinal data for all participants within the primary analysis population. The survival component is an exponential proportional hazards model. The functional and survival components are connected through a shared common treatment effect (slowing in rate of progression of the disease). The shared treatment effect between the functional and survival components allows the participants who were loss to follow-up due to mortality to inform treatment effect estimates beyond their censored ALSFRS-R longitudinal data. The degree to which treatment effects on mortality inform the shared treatment effect parameter depends on the mortality rate within the study.

The ALSFRS-R repeated measures model is based on a linear rate of progression in ALSFRS-R for placebo participants and a proportional slowing in the rate of progression for active treatment participants at each time point. The treatment effect is quantified by the disease rate ratio (DRR), which represents the ration of the rate of decline of disease progression (rate of ALSFRS-R and mortality rate) of active treatement participant relative to placebo participants (i.e. a proportional slowing). The model incorporates participant-level random effects in the baseline value of the ALSFRS-R (intercept) and in the rate of progression (slope); covariate effects to account for covariate-explainable differences in rates of progression based on participant-specific baseline covariates; and regimen-specific random effects in the rate of progression (slope). Regimen-specific random effects are used to account for potential differences in the shared control group not explained by the included covariates. Full specification of the primary efficacy analysis is described in Appendix I of the M-SAP.

11.2.3 Handling of Missing Data

The Bayesian repeated-measures model of ALSFRS-R naturally accommodates differential length of follow-up and utilizes all available longitudinal data for each participant. The model also accommodates missing data due to death (via included mortality component), as well as data missing at random (i.e. due to reasons unrelated to disease progression). No imputation is planned for the primary efficacy analysis. However, planned sensitivity analyses will investigate the potential effect of missing data on inference and estimates from the primary analysis.

11.2.4 Handling of Re-Screened and Repeat Participants

Upon completion of a regimen, a research participant may be re-screened into the Master Protocol and assigned to another regimen after 30 days or 5 half-lives (if known), whichever is longer. Those participants who have been re-screened and meet eligibility criteria for a subsequent randomization will be analyzed as new participants with new baseline visits and sets of observations for each regimen to which they were assigned. The baseline visit for each regimen is defined as the visit after assignment to and randomization within that regimen. Within the primary analysis, sets of observations from a repeat participant across multiple regimens will be treated as independent conditional on baseline covariates within each regimen. After the required washout period and adjusting for progression during previous regimens in the primary analysis (pre-baseline ALSFRS-R slope), it is not expected that future ALSFRS-R progression will be associated with active treatment or placebo received in the previous regimen.

11.2.5 Inclusion of Multiple Doses

If an RSA includes multiple doses under investigation, the recommended primary analysis will be a pooled analysis of all active doses compared to the shared control. Additionally, secondary analyses will be specified within the R-SAP to investigate each active dose separately.

11.3 Interim Analyses and Trial Adaptations

Multiple interim analyses will allow each regimen to stop randomization early for success (if applicable) or futility, with interims occurring simultaneously for all actively enrolling regimens with sufficient data. Interims will begin when at least one regimen within the master protocol has 40 randomized participants (30 active and 10 control) with the opportunity to complete at least 24 weeks of follow-up. Subsequent interims will occur every 12 weeks. If a regimen is not stopped early for success (if applicable) or futility at an interim analysis, the regimen-specific final analysis of the placebo-controlled period will take place after all participants randomized to that regimen have had the opportunity to complete 24-weeks of follow-up. The default design for a regimen will include stopping early only for futility.

11.3.1 Regimen Success

The default design for a regimen will not include early stopping for success. If early stopping for success is included in a regimen, success will be declared based on the posterior probability that the active treatment is superior to the placebo group. The posterior probability for declaring success is specified in Appendix I of the M-SAP and determined based on clinical trial simulations to achieve an overall one-sided Type I error of 2.5%.

11.3.2 Regimen Futility

Futility will be declared for a regimen at an interim analysis if the posterior probability that active treatment slows disease progression relative to placebo by at least 10% is less than 5%. Early stopping for futility in non-binding.

11.4 Secondary, Exploratory, and Sensitivity Analyses

11.4.1 Secondary Efficacy Analyses

As a secondary analysis, a joint-rank test of function and survival will be performed. The analysis will rank each pair of participants based first on survival time during the 24-week placebo-controlled period and second, if they both survived or one was censored prior to the observed survival endpoint of the other, on the change in ALSFRS-R total score from baseline to the last jointly observed visit up to 24-weeks[20-22]. The summed ranks will be analyzed by linear regression to adjust for important covariates.

11.4.2 Secondary Endpoints

Secondary endpoints and analyses will be detailed within each RSA and R-SAP.

11.4.3 Exploratory Analyses

Exploratory endpoints and their corresponding analyses will be detailed within each RSA and R-SAP. Such analyses may evaluate alternative functional endpoints and biofluid biomarkers for monitoring disease progression.

11.4.4 Sensitivity Analyses

Additional sensitivity analyses of the primary efficacy analysis will be conducted for various definitions of the analysis population (e.g. depending on definition of shared controls), and to evaluate the potential effect of modeling assumptions and missing data. Further details are provided in Appendix I of the M-SAP.

11.5 Safety Analyses

Safety data, including deaths and death-equivalents, treatment-emergent adverse events (TEAE), and changes in vital signs, ECGs, and clinical laboratory test results, will be summarized for each treatment group among all participants who initiate treatment. The frequency and type of any observed suicidal ideation or suicidal behavior will be described. Descriptive statistics of continuous measures will be provided for the observed data and for the change from baseline at each measured time point. Time-to-death or death equivalent will be estimated by Kaplan-Meier

product-limit estimates. Adverse events will be summarized by treatment group as counts of events and as the proportion of participants experiencing any given type of TEAE as classified by MedDRA system organ class, high level term, and preferred term and classified according to severity, relatedness, and outcome. Laboratory test results will be classified by Common Terminology Criteria for Adverse Events (CTCAE) grad and as below the lower limit of normal, within normal limits and above the upper limit of normal. Shift tables will be used to summarize changes from baseline to each visit by treatment group. Clinically significant out-of-range laboratory tests are recorded as adverse events and will be documented in the TEAE summaries. Additional safety assessments may be specified in the R-SAP.

11.6 Sample Size Justification

Clinical trial simulation is used to quantify operating characteristics for each regimen. In particular, virtual participant outcomes are created under different assumptions for key design parameters including placebo rates of progression for ALSFRS-R, measurement error for ALSFRS-R, mortality rates, treatment effects on ALSFRS-R, treatment effects on mortality, dropout rates and accrual rates. For each set of simulation assumptions (i.e. a scenario), many trials are simulated and virtually executed, including all interim analyses for early success and early futility. Trial operating characteristics are summarized across all simulated trials for each scenario. Complete details and results are provided in Appendix I of the M-SAP.

Simulations outlined in Appendix I of the M-SAP, provide example operating characteristics of the recommended design for the first 3 regimens enrolling in the platform across a range of simulation assumptions. Under the null scenario there was an overall one-sided Type I error rate of 2.4% per regimen. The average duration of the regimen was 14 months and there was a 28% chance that the regimen would meet the criterion for early stopping for futility.

The Master Protocol recommended design has 61%, 77%, and 88% power to detect a slowing in the rate of ALSFRS-R progression of 25%, 30% and 35% respectively when function and survival have a common treatment effect. When there is no benefit for survival and a 30% slowing in ALSFRS-R progression the power drops to 72%. When there is a negative effect on survival and a 30% slowing in ALSFRS-R progression, the power drops further to 68%. There is less than a 1% chance in these scenarios that the regimen would meet the criterion for early stopping for futility.

12 DATA COLLECTION, MANAGEMENT, MONITORING, AND REPORTING

The EDC system will be developed to facilitate the collection, management, monitoring, and reporting of study data for the Master Protocol.

12.1 Quality Assurance

Protocol procedures are reviewed with the SI and associated personnel prior to the study to ensure the accuracy and reliability of data. Each SI must adhere to the protocol detailed in this document and agree that any changes to the protocol must be approved by the CC prior to implementing the change unless it is to address immediate participant safety.

12.2 Role of Data Management

Data Management (DM) is the development, execution and supervision of plans, policies, programs, and practices that control, protect, deliver, and enhance the value of data and information assets. The Neurological Clinical Research Institute (NCRI) will serve as the Data Coordination Center (DCC) for the Master Protocol and is responsible for developing, testing, and managing the clinical data systems and management activities. All data will be managed in compliance with NCRI policies and applicable regulatory requirements.

12.2.1 Data Collection

Site personnel will collect data onto paper source documents as appropriate and into electronic source documents (e.g., voice sample data, EMR data, etc). Values from paper source documents will be transcribed into the corresponding eCRFs in the EDC system by site personnel; electronically captured data will be transmitted to their respective vendors for consolidation and reconciliation with the study EDC system. Clinical sites will be monitored to ensure compliance with the study protocol, data management requirements, and GCP.

12.2.2 Data Entry and Edit Checks

Site personnel are instructed to enter collected data into the EDC system shortly after a visit. Please note: SAEs must be entered into the EDC system and reported to the CC within 24 hours of site awareness of the SAE. Data collection is the responsibility of the staff at the site under the supervision of the SI as specified in the delegation log for that site. During the study, the SI must maintain complete and accurate documentation for the study.

To ensure accuracy and completeness of the data set, logic and range checks as well as in-form rules will be built into the EDC system, and electronic queries will be created to track potential data issues. The sites will only have access to the queries concerning their own participants.

12.2.3 Data Lock Process

The EDC system will have the ability to lock each trial regimen independently to prevent any modification of data once a trial regimen is closed. Once this option is activated, every user will have read-only access to the data until all access is revoked following NCRI DM procedures. The specific database lock procedures are detailed in the DM Standard Operating Procedures (SOP).

12.3 Role of Study Monitors

All aspects of the trial, including the individual regimens, will be monitored by qualified individuals designated by the Sponsor. Monitoring will be conducted according to GCP and applicable government regulations. The SIs will agree to allow monitors access to the clinical supplies, dispensing and storage areas, and to the clinical files of the study participants, and, if requested, agree to assist the monitors. Monitoring visits will be conducted according to a pre-defined Monitoring Plan.

12.3.1 Clinical Monitoring

Study Monitors will visit each study site during the course of the study to review source documentation, ICF, and the clinical facilities to ensure the study is conducted in accordance with the study protocol and compliance with ICH/GCP and regulatory guidelines. Investigators are responsible for allowing access to all source documents and medical records related to the subject's participation in the study. Monitoring visits will occur at defined intervals per the Study Monitoring Plan. Study monitor(s) will identify study non-compliance and if appropriate, the study monitor(s) will assist the site with developing a corrective and preventative action plan. All significant noncompliance will be communicated to the Sponsor. Regimen specific monitoring activities will be specified in the appendices of the Study Monitoring Plan.

12.3.2 Monitoring Report

The Study Monitors will provide monitoring reports to the Sponsor and, if requested, and when required, will provide reports of protocol compliance to the Sponsor and other applicable parties as detailed in the Study Monitoring Plan.

12.4 Data and Safety Monitoring Board

An independent DSMB will be assembled for the trial. A DSMB Charter will detail the processes of this group. The DSMB will receive blinded and unblinded summary reports of the frequency of all clinical adverse events and safety laboratory tests for each trial regimens at planned

periodic meetings throughout the study as specified in the DSMB Charter. The DSMB will receive separate reports per trial regimen to review independently. Placebo data from all trial regimens will be compiled into a single report for review. Meetings will be held in-person or via teleconference.

Summaries of serious adverse events and enrollment per trial regimen will be provided to the DSMB by the Study Biostatisticians as specified in the DSMB Charter. AE(s) of Special Interest events occurring within 24 hours of dosing for any given treatment arm, and any severe unexpected serious adverse events for a treatment arm are considered events of interest and will be reported in real-time (within 1 business day of CC awareness) to the DSMB. The DSMB can ask to receive the SAE reports more frequently. As necessary, the DSMB can review the frequencies of clinical and laboratory abnormalities. Recommendations for modification or termination of the trial based on safety data will be made by the DSMB to the Sponsor. The DSMB will review safety data throughout the trial and may stop enrollment into a treatment arm for safety if they determine that there is a significant difference in the rate of a particular adverse event that would indicate a risk that is greater than the possible benefit of the investigational product. A notable increase in the frequency of any adverse event should be examined by the DSMB although it may not lead to a recommendation by the DSMB.

Prior to each DSMB meeting, the CC will provide an update to the DSMB on enrollment, data quality (missing data), and protocol adherence for each treatment arm. The CC will be responsible for communication with the DSMB.

Complete information can be found in the DSMB Charter.

12.5 Neurological Global Unique Identifier

A participant Neurological Global Unique Identifier (NeuroGUID), or its derivitives', will be used as the identifier for participant's samples participating in this study. The NeuroGUID is an 11-character string that is generated using encryption technology and algorithms licensed by the NCRI from the NIH in 2013.

The NeuroGUID is generated on a secure website that utilizes 128-bit Secure Socket Layer by using an irreversible encryption algorithm that accepts ten identifying data elements, (e.g., last name at birth, first name at birth, sex at birth, day, month and year of birth, city and country of birth, etc.), and creates a series of coded strings ("hashes") that are encrypted and sent to a secure server. The server produces a unique randomly generated alphanumeric string or NeuroGUID. No identifying information is stored in the system; it is simply used to guarantee the uniqueness of a NeuroGUID. If the same information is entered again, the same NeuroGUID will be returned.

12.6 Data Handling and Record Keeping

The SI is responsible for ensuring the accuracy, completeness, legibility, and timeliness of the data reported. All source documents should be completed in a neat, legible manner to ensure accurate interpretation of data. Dark ink is required to ensure clarity of reproduced copies. When making changes or corrections, cross out the original entry with a single line, and initial and date the change. Do not erase, overwrite, or use correction fluid or tape on the original.

Source document templates (SDTs) will be provided for use and maintained for recording data for each participant enrolled in the study. Data reported in the eCRF derived from source documents should be consistent with the source documents and discrepancies should be explained.

12.6.1 Confidentiality

Study participant medical information obtained by this study is confidential, and disclosure to third parties other than those noted below is prohibited. Upon the participant's permission, medical information may be given to his or her personal physician or other appropriate medical personnel responsible for his or her welfare. All local and federal guidelines and regulations regarding maintaining study participant confidentiality of data will be adhered to.

Data generated by this study must be available for inspection by representatives of the US FDA, the Office for Human Research Protections (OHRP), the sponsor, all pertinent national and local health and regulatory authorities, the CC or their representative, Study Monitoring personnel, and the sIRB.

12.6.2 Retention of Records

US FDA regulations (21 CFR 312.62[c]) require that records and documents pertaining to the conduct of this study and the distribution of investigational drugs, including CRFs (if applicable), consent forms, laboratory test results, and medical inventory records, must be retained by the SI for two years after marketing application approval. If no application is filed, these records must be kept for two years after the investigation is discontinued and the US FDA and the applicable national and local health authorities are notified. The CC or their representative will notify the SIs of these events. The SIs should retain all study documents and records until they are notified in writing by the Sponsor or their representative.

No records may be disposed of without the written approval of the Sponsor. Written notification should be provided to the Sponsor prior to transferring any records to another party or moving them to another location.

12.7 Reporting, Publications, and Notification of Results

12.7.1 Publication Policy

The HEALEY ALS Platform Trial Executive Committee will set the policy for publication of results from the HEALEY ALS Platform Trial and all RSAs. No data or results generated from the HEALEY ALS Platform Trial or its RSAs may be published without written agreement from the HEALEY ALS Platform Trial Executive Committee.

The responsibilities of the HEALEY ALS Platform Trial Executive Committee are outlined in the HEALEY ALS Platform Trial Governance Plan.

12.7.2 Data and Sample Sharing

Data and sample sharing policies will be specified in the legal agreements governing the trial.

12.7.3 Trial Registration

The Master Protocol and each RSA will be registered on www.ClinicalTrials.gov. Results will be posted to ClinicalTrials.gov according to applicable regulations.

13 APPENDICES

Appendix I: El Escorial World Federation of Neurology Criteria for the Diagnosis of ALS

Information obtained from the web site: ***www.wfnals.org***.

The diagnosis of Amyotrophic Lateral Sclerosis [ALS] requires:
A - The presence of:
(A:1) evidence of lower motor neuron (LMN) degeneration by clinical, electrophysiology cal or neuropathologic examination,
(A:2) evidence of upper motor neuron (UMN) degeneration by clinical examination, and
(A:3) progressive spread of symptoms or signs within a region or to other regions, as determined by history or examination, together with
B - The absence of:
(B:1) electrophysiological and pathological evidence of other disease processes that might
explain the signs of LMN and/or UMN degeneration, and
(B:2) neuroimaging evidence of other disease processes that might explain the observed clinical and electrophysiological signs.

CLINICAL STUDIES IN THE DIAGNOSIS OF ALS
A careful history, physical and neurological examination must search for clinical evidence of UMN and LMN signs in four regions [brainstem, cervical, thoracic, or lumbosacral spinal cord] (see Table 1) of the central nervous system [CNS]. Ancillary tests should be reasonably applied, as clinically indicated, to exclude other disease processes. These should include electrodiagnostic, neurophysiological, neuroimaging and clinical laboratory studies. Clinical evidence of LMN and UMN degeneration is required for the diagnosis of ALS. The clinical diagnosis of ALS, without pathological confirmation, may be categorized into various levels of certainty by clinical assessment alone depending on the presence of UMN and LMN signs together in the same topographical anatomic region in either the brainstem [bulbar cranial motor neurons], cervical, thoracic, or lumbosacral spinal cord [anterior horn motor neurons]. The terms Clinical Definite ALS and Clinically Probable ALS are used to describe these categories of clinical diagnostic certainty on clinical criteria alone:

A. Clinically Definite ALS is defined on clinical evidence alone by the presence of UMN, as well as LMN signs, in three regions.

B. Clinically Probable ALS is defined on clinical evidence alone by UMN and LMN signs in at least two regions with some UMN signs necessarily rostral to (above) the LMN signs.

C. Clinically Probable ALS - Laboratory-supported is defined when clinical signs of UMN and LMN dysfunction are in only one region, or when UMN signs alone are present in one region, and LMN signs defined by EMG criteria are present in at least two limbs, with proper application of neuroimaging and clinical laboratory protocols to exclude other causes.

D. Clinically Possible ALS is defined when clinical signs of UMN and LMN dysfunction are found together in only one region or UMN signs are found alone in two or more regions; or LMN signs are found rostral to UMN signs and the diagnosis of Clinically Probable - Laboratory-supported ALS cannot be proven by evidence on clinical grounds in conjunction with electrodiagnostic, neurophysiologic, neuroimaging or clinical laboratory studies. Other diagnoses must have been excluded to accept a diagnosis of Clinically Possible ALS.

Table 1

	Brainstem	Cervical	Thoracic	Lumbosacral
Lower motor neuron signs weakness, atrophy, fasciculations	jaw, face, palate, tongue, larynx	neck, arm, hand, diaphragm	back, abdomen	back, abdomen, leg, foot
Upper motor neuron signs pathologic spread of reflexes, clonus, etc.	clonic jaw<br>gag reflex<br>exaggerated snout reflex<br>pseudobulbar features<br>forced yawning<br>pathologic DTRs<br>spastic tone	clonic DTRs<br>Hoffman reflex<br>pathologic DTRs<br>spastic tone<br><br>preserved reflex in weak wasted limb	loss of superficial abdominal reflexes<br>pathologic DTRs<br>spastic tone	clonic DTRs - extensor plantar response<br>pathologic DTRs<br>spastic tone<br><br>preserved reflex in weak wasted limb

Appendix II: ALS Functional Rating Scale – Revised (ALSFRS-R)

ALSFRS-R

QUESTIONS: **SCORE:**

1. Speech
   - 4 = Normal speech processes
   - 3 = Detectable speech disturbances
   - 2 = Intelligible with repeating
   - 1 = Speech combined with nonvocal communication
   - 0 = Loss of useful speech

2. Salivation
   - 4 = Normal
   - 3 = Slight but definite excess of saliva in mouth; may have nighttime drooling
   - 2 = Moderately excessive saliva; may have minimal drooling
   - 1 = Marked excess of saliva with some drooling
   - 0 = Marked drooling; requires constant tissue or handkerchief

3. Swallowing
   - 4 = Normal eating habits
   - 3 = Early eating problems – occasional choking
   - 2 = Dietary consistency changes
   - 1 = Needs supplemental tube feeding
   - 0 = NPO (exclusively parenteral or enteral feeding)

4. Handwriting
   - 4 = Normal
   - 3 = Slow or sloppy; all words are legible
   - 2 = Not all words are legible
   - 1 = No words are legible but can still grip a pen
   - 0 = Unable to grip pen

5a. Cutting Food and Handling Utensils (patients without gastrostomy)
   - 4 = Normal
   - 3 = Somewhat slow and clumsy, but no help needed

2 = Can cut most foods, although clumsy and slow; some help needed
1 = Food must be cut by someone, but can still feed slowly
0 = Needs to be fed

5b. Cutting Food and Handling Utensils (alternate scale for patients with gastrostomy) ☐
4 = Normal
3 = Clumsy, but able to perform all manipulations independently
2 = Some help needed with closures and fasteners
1 = Provides minimal assistance to caregivers
0 = Unable to perform any aspect of task

6. Dressing and Hygiene ☐
4 = Normal function
3 = Independent, can complete self-care with effort or decreased efficiency
2 = Intermittent assistance or substitute methods
1 = Needs attendant for self-care
0 = Total dependence

7. Turning in Bed and Adjusting Bed Clothes ☐
4 = Normal function
3 = Somewhat slow and clumsy, but no help needed
2 = Can turn alone, or adjust sheets, but with great difficulty
1 = Can initiate, but not turn or adjust sheets alone
0 = Helpless

8. Walking ☐
4 = Normal
3 = Early ambulation difficulties
2 = Walks with assistance
1 = Nonambulatory functional movement only
0 = No purposeful leg movement

9. Climbing Stairs ☐
4 = Normal
3 = Slow
2 = Mild unsteadiness or fatigue
1 = Needs assistance

0 = Cannot do

R-1. Dyspnea ☐

4 = None
3 = Occurs when walking
2 = Occurs with one or more of the following: eating, bathing, dressing
1 = Occurs at rest, difficulty breathing when either sitting or lying
0 = Significant difficulty, considering using mechanical respiratory support

R-2 Orthopnea ☐

4 = None
3 = Some difficulty sleeping at night due to shortness of breath, does not routinely use more than two pillows
2 = Needs extra pillow in order to sleep (more than two)
1 = Can only sleep sitting up
0 = Unable to sleep without mechanical assistance

R-3 Respiratory Insufficiency ☐

4 = None
3 = Intermittent use of BiPAP
2 = Continuous use of BiPAP during the night
1 = Continuous use of BiPAP during the night and day
0 = Invasive mechanical ventilation by intubation or tracheostomy

Evaluator's Initials: _____ _____ ______

Appendix III: Columbia-Suicide Severity Rating Scale (C-SSRS) Baseline Version

Information obtained from: http://www.cssrs.columbia.edu/

SUICIDAL IDEATION	
Ask questions 1 and 2. If both are negative, proceed to "Suicidal Behavior" section. If the answer to question 2 is "yes", ask questions 3, 4 and 5. If the answer to question 1 and/or 2 is "yes", complete "Intensity of Ideation" section below.	Lifetime: Time He/She Felt Most Suicidal
**1. Wish to be Dead**<br>Subject endorses thoughts about a wish to be dead or not alive anymore, or wish to fall asleep and not wake up.<br>***Have you wished you were dead or wished you could go to sleep and not wake up?***<br>If yes, describe:	**Yes No**<br>☐ ☐
**2. Non-Specific Active Suicidal Thoughts**<br>General, non-specific thoughts of wanting to end one's life/commit suicide (e.g., *"I've thought about killing myself"*) without thoughts of ways to kill oneself/associated methods, intent, or plan.<br>***Have you actually had any thoughts of killing yourself?***<br>If yes, describe:	**Yes No**<br>☐ ☐
**3. Active Suicidal Ideation with Any Methods (Not Plan) without Intent to Act**<br>Subject endorses thoughts of suicide and has thought of at least one method during the assessment period. This is different than a specific plan with time, place or method details worked out (e.g., thought of method to kill self but not a specific plan). Includes person who would say, *"I thought about taking an overdose but I never made a specific plan as to when, where or how I would actually do it…and I would never go through with it."*<br>***Have you been thinking about how you might do this?***<br>If yes, describe:	**Yes No**<br>☐ ☐
**4. Active Suicidal Ideation with Some Intent to Act, without Specific Plan**<br>Active suicidal thoughts of killing oneself and subject reports having some intent to act on such thoughts, as opposed to *"I have the thoughts but I definitely will not do anything about them."*<br>***Have you had these thoughts and had some intention of acting on them?***<br>If yes, describe:	**Yes No**<br>☐ ☐
**5. Active Suicidal Ideation with Specific Plan and Intent**<br>Thoughts of killing oneself with details of plan fully or partially worked out and subject has some intent to carry it out.<br>***Have you started to work out or worked out the details of how to kill yourself? Do you intend to carry out this plan?***<br>If yes, describe:	**Yes No**<br>☐ ☐
INTENSITY OF IDEATION	
The following features should be rated with respect to the most severe type of ideation (i.e., 1-5 from above, with 1 being the least severe and 5 being the most severe). Ask about time he/she was feeling the most suicidal.<br>_____ ____<br>***Most Severe Ideation:***<br>***Type # (1-5) Description of Ideation***	Most Severe
**Frequency**<br>***How many times have you had these thoughts?***<br>(1) Less than once a week (2) Once a week (3) 2-5 times in week (4) Daily or almost daily (5) Many times each day	____
**Duration**<br>***When you have the thoughts, how long do they last?***<br>(1) Fleeting - few seconds or minutes (4) 4-8 hours/most of day<br>(2) Less than 1 hour/some of the time (5) More than 8 hours/persistent or continuous<br>(3) 1-4 hours/a lot of time	____

**Controllability**<br>***Could/can you stop thinking about killing yourself or wanting to die if you want to?***<br>(1) Easily able to control thoughts (4) Can control thoughts with a lot of difficulty<br>(2) Can control thoughts with little difficulty (5) Unable to control thoughts<br>(3) Can control thoughts with some difficulty (0) Does not attempt to control thoughts	____
**Deterrents**<br>***Are there things - anyone or anything (e.g., family, religion, pain of death) - that stopped you from wanting to die or acting on thoughts of committing suicide?***<br>(1) Deterrents definitely stopped you from attempting suicide (4) Deterrents most likely did not stop you<br>(2) Deterrents probably stopped you (5) Deterrents definitely did not stop you<br>(3) Uncertain that deterrents stopped you (0) Does not apply	____
**Reasons for Ideation**<br>***What sort of reasons did you have for thinking about wanting to die or killing yourself? Was it to end the pain or stop the way you were feeling (in other words you couldn't go on living with this pain or how you were feeling) or was it to get attention, revenge or a reaction from others? Or both?***<br>(1) Completely to get attention, revenge or a reaction from others (4) Mostly to end or stop the pain (you couldn't go on<br>(2) Mostly to get attention, revenge or a reaction from others living with the pain or how you were feeling)<br>(3) Equally to get attention, revenge or a reaction from others (5) Completely to end or stop the pain (you couldn't go on<br>and to end/stop the pain. living with the pain or how you were feeling)<br>(0) Does not apply	____

SUICIDAL BEHAVIOR<br>*(Check all that apply, so long as these are separate events; must ask about all types)*	**Lifetime**
**Actual Attempt:**<br>A potentially self-injurious act committed with at least some wish to die, *as a result of act.* Behavior was in part thought of as method to kill oneself. Intent does not have to be 100%. If there is ***any*** intent/desire to die associated with the act, then it can be considered an actual suicide attempt. ***There does not have to be any injury or harm***, just the potential for injury or harm. If person pulls trigger while gun is in mouth but gun is broken so no injury results, this is considered an attempt.<br>Inferring Intent: Even if an individual denies intent/wish to die, it may be inferred clinically from the behavior or circumstances. For example, a highly lethal act that is clearly not an accident so no other intent but suicide can be inferred (e.g., gunshot to head, jumping from window of a high floor/story). Also, if someone denies intent to die, but they thought that what they did could be lethal, intent may be inferred.<br>***Have you made a suicide attempt?***<br>***Have you done anything to harm yourself?***<br>***Have you done anything dangerous where you could have died?***<br>***What did you do?***<br>***Did you______ as a way to end your life?***<br>***Did you want to die (even a little) when you_____?***<br>***Were you trying to end your life when you _____?***<br>***Or did you think it was possible you could have died from_____?***<br>***Or did you do it purely for other reasons / without ANY intention of killing yourself (like to relieve stress, feel better, get sympathy, or get something else to happen)?*** *(*Self-Injurious Behavior without suicidal intent)<br>If yes, describe:<br>**Has subject engaged in Non-Suicidal Self-Injurious Behavior?**	**Yes No**<br>☐ ☐<br><br>Total # of Attempts<br>______<br><br>**Yes No**<br>☐ ☐

<table>
<tr><td colspan="3">Interrupted Attempt:
When the person is interrupted (by an outside circumstance) from starting the potentially self-injurious act (if not for that, actual attempt would have occurred).
Overdose: Person has pills in hand but is stopped from ingesting. Once they ingest any pills, this becomes an attempt rather than an interrupted attempt. Shooting: Person has gun pointed toward self, gun is taken away by someone else, or is somehow prevented from pulling trigger. Once they pull the trigger, even if the gun fails to fire, it is an attempt. Jumping: Person is poised to jump, is grabbed and taken down from ledge. Hanging: Person has noose around neck but has not yet started to hang - is stopped from doing so.
Has there been a time when you started to do something to end your life but someone or something stopped you before you actually did anything?
If yes, describe:</td><td>Yes No
☐ ☐

Total # of interrupted
______</td></tr>
<tr><td colspan="3">Aborted Attempt:
When person begins to take steps toward making a suicide attempt, but stops themselves before they actually have engaged in any self-destructive behavior. Examples are similar to interrupted attempts, except that the individual stops him/herself, instead of being stopped by something else.
Has there been a time when you started to do something to try to end your life but you stopped yourself before you actually did anything?
If yes, describe:</td><td>Yes No
☐ ☐

Total # of aborted
______</td></tr>
<tr><td colspan="3">Preparatory Acts or Behavior:
Acts or preparation towards imminently making a suicide attempt. This can include anything beyond a verbalization or thought, such as assembling a specific method (e.g., buying pills, purchasing a gun) or preparing for one's death by suicide (e.g., giving things away, writing a suicide note).
Have you taken any steps towards making a suicide attempt or preparing to kill yourself (such as collecting pills, getting a gun, giving valuables away or writing a suicide note)?
If yes, describe:</td><td>Yes No
☐ ☐</td></tr>
<tr><td colspan="3">Suicidal Behavior:
Suicidal behavior was present during the assessment period?</td><td>Yes No
☐ ☐</td></tr>
<tr><td>Answer for Actual Attempts Only</td><td>Most Recent Attempt Date:</td><td>Most Lethal Attempt Date:</td><td>Initial/First Attempt Date:</td></tr>
<tr><td>Actual Lethality/Medical Damage:
0. No physical damage or very minor physical damage (e.g., surface scratches).
1. Minor physical damage (e.g., lethargic speech; first-degree burns; mild bleeding; sprains).
2. Moderate physical damage; medical attention needed (e.g., conscious but sleepy, somewhat responsive; second-degree burns; bleeding of major vessel).
3. Moderately severe physical damage; medical hospitalization and likely intensive care required (e.g., comatose with reflexes intact; third-degree burns less than 20% of body; extensive blood loss but can recover; major fractures).
4. Severe physical damage; medical hospitalization with intensive care required (e.g., comatose without reflexes; third-degree burns over 20% of body; extensive blood loss with unstable vital signs; major damage to a vital area).
5. Death</td><td>Enter Code
______</td><td>Enter Code
______</td><td>Enter Code
______</td></tr>
<tr><td>Potential Lethality: Only Answer if Actual Lethality=0
Likely lethality of actual attempt if no medical damage (the following examples, while having no actual medical damage, had potential for very serious lethality: put gun in mouth and pulled the trigger but gun fails to fire so no medical damage; laying on train tracks with oncoming train but pulled away before run over).
0 = Behavior not likely to result in injury
1 = Behavior likely to result in injury but not likely to cause death
2 = Behavior likely to result in death despite available medical care</td><td>Enter Code
______</td><td>Enter Code
______</td><td>Enter Code
______</td></tr>
</table>

Appendix IV: Columbia-Suicide Severity Rating Scale (C-SSRS) Since Last Visit Version

Information obtained from: http://www.cssrs.columbia.edu/

SUICIDAL IDEATION	
Ask questions 1 and 2. If both are negative, proceed to "Suicidal Behavior" section. If the answer to question 2 is "yes", ask questions 3, 4 and 5. If the answer to question 1 and/or 2 is "yes", complete "Intensity of Ideation" section below.	Since Last Visit
**1. Wish to be Dead**<br>Subject endorses thoughts about a wish to be dead or not alive anymore, or wish to fall asleep and not wake up.<br>***Have you wished you were dead or wished you could go to sleep and not wake up?***<br>If yes, describe:	**Yes No**<br>□ □
**2. Non-Specific Active Suicidal Thoughts**<br>General, non-specific thoughts of wanting to end one's life/commit suicide (e.g., *"I've thought about killing myself"*) without thoughts of ways to kill oneself/associated methods, intent, or plan during the assessment period.<br>***Have you actually had any thoughts of killing yourself?***<br>If yes, describe:	**Yes No**<br>□ □
**3. Active Suicidal Ideation with Any Methods (Not Plan) without Intent to Act**<br>Subject endorses thoughts of suicide and has thought of at least one method during the assessment period. This is different than a specific plan with time, place or method details worked out (e.g., thought of method to kill self but not a specific plan). Includes person who would say, *"I thought about taking an overdose but I never made a specific plan as to when, where or how I would actually do it…and I would never go through with it."*<br>***Have you been thinking about how you might do this?***<br>If yes, describe:	**Yes No**<br>□ □
**4. Active Suicidal Ideation with Some Intent to Act, without Specific Plan**<br>Active suicidal thoughts of killing oneself and subject reports having some intent to act on such thoughts, as opposed to *"I have the thoughts but I definitely will not do anything about them."*<br>***Have you had these thoughts and had some intention of acting on them?***<br>If yes, describe:	**Yes No**<br>□ □
**5. Active Suicidal Ideation with Specific Plan and Intent**<br>Thoughts of killing oneself with details of plan fully or partially worked out and subject has some intent to carry it out.<br>***Have you started to work out or worked out the details of how to kill yourself? Do you intend to carry out this plan?***<br>If yes, describe:	**Yes No**<br>□ □
INTENSITY OF IDEATION	
The following features should be rated with respect to the most severe type of ideation (i.e., 1-5 from above, with 1 being the least severe and 5 being the most severe).<br>***Most Severe Ideation: _____ ____***<br>***Type # (1-5) Description of Ideation***	Most Severe
**Frequency**<br>***How many times have you had these thoughts?***<br>(1) Less than once a week (2) Once a week (3) 2-5 times in week (4) Daily or almost daily (5) Many times each day	____
**Duration**<br>***When you have the thoughts, how long do they last?***<br>(1) Fleeting - few seconds or minutes (4) 4-8 hours/most of day<br>(2) Less than 1 hour/some of the time (5) More than 8 hours/persistent or continuous<br>(3) 1-4 hours/a lot of time	____
**Controllability**<br>***Could/can you stop thinking about killing yourself or wanting to die if you want to?***<br>(1) Easily able to control thoughts (4) Can control thoughts with a lot of difficulty<br>(2) Can control thoughts with little difficulty (5) Unable to control thoughts<br>(3) Can control thoughts with some difficulty (0) Does not attempt to control thoughts	____

**Deterrents**<br>***Are there things - anyone or anything (e.g., family, religion, pain of death) - that stopped you from wanting to die or acting on thoughts of committing suicide?***<br>(1) Deterrents definitely stopped you from attempting suicide (4) Deterrents most likely did not stop you<br>(2) Deterrents probably stopped you (5) Deterrents definitely did not stop you<br>(3) Uncertain that deterrents stopped you (0) Does not apply	____
**Reasons for Ideation**<br>***What sort of reasons did you have for thinking about wanting to die or killing yourself? Was it to end the pain or stop the way you were feeling (in other words you couldn't go on living with this pain or how you were feeling) or was it to get attention, revenge or a reaction from others? Or both?***<br>(1) Completely to get attention, revenge or a reaction from others (4) Mostly to end or stop the pain (you couldn't go on<br>(2) Mostly to get attention, revenge or a reaction from others living with the pain or how you were feeling)<br>(3) Equally to get attention, revenge or a reaction from others (5) Completely to end or stop the pain (you couldn't go on<br>and to end/stop the pain living with the pain or how you were feeling)<br>(0) Does not apply	____

SUICIDAL BEHAVIOR<br>*(Check all that apply, so long as these are separate events; must ask about all types)*	Since Last Visit
**Actual Attempt:**<br>A potentially self-injurious act committed with at least some wish to die, *as a result of act.* Behavior was in part thought of as method to kill oneself. Intent does not have to be 100%. If there is ***any*** intent/desire to die associated with the act, then it can be considered an actual suicide attempt. ***There does not have to be any injury or harm***, just the potential for injury or harm. If person pulls trigger while gun is in mouth but gun is broken so no injury results, this is considered an attempt.<br>Inferring Intent: Even if an individual denies intent/wish to die, it may be inferred clinically from the behavior or circumstances. For example, a highly lethal act that is clearly not an accident so no other intent but suicide can be inferred (e.g., gunshot to head, jumping from window of a high floor/story). Also, if someone denies intent to die, but they thought that what they did could be lethal, intent may be inferred.<br>***Have you made a suicide attempt?***<br>***Have you done anything to harm yourself?***<br>***Have you done anything dangerous where you could have died?***<br>***What did you do?***<br>***Did you______ as a way to end your life?***<br>***Did you want to die (even a little) when you_____?***<br>***Were you trying to end your life when you _____?***<br>***Or did you think it was possible you could have died from_____?***<br>***Or did you do it purely for other reasons / without ANY intention of killing yourself (like to relieve stress, feel better, get sympathy, or get something else to happen)?*** (Self-Injurious Behavior without suicidal intent)<br>If yes, describe:<br>**Has subject engaged in Non-Suicidal Self-Injurious Behavior?**	**Yes No**<br>☐ ☐<br><br>Total # of Attempts<br>______<br><br>**Yes No**<br>☐ ☐
**Interrupted Attempt:**<br>When the person is interrupted (by an outside circumstance) from starting the potentially self-injurious act *(if not for that, actual attempt would have occurred).*<br>Overdose: Person has pills in hand but is stopped from ingesting. Once they ingest any pills, this becomes an attempt rather than an interrupted attempt. Shooting: Person has gun pointed toward self, gun is taken away by someone else, or is somehow prevented from pulling trigger. Once they pull the trigger, even if the gun fails to fire, it is an attempt. Jumping: Person is poised to jump, is grabbed and taken down from ledge. Hanging: Person has noose around neck but has not yet started to hang - is stopped from doing so.<br>***Has there been a time when you started to do something to end your life but someone or something stopped you before you actually did anything?***<br>If yes, describe:	**Yes No**<br>☐ ☐<br><br>Total # of interrupted<br>______

**Aborted Attempt:**<br>When person begins to take steps toward making a suicide attempt, but stops themselves before they actually have engaged in any self-destructive behavior. Examples are similar to interrupted attempts, except that the individual stops him/herself, instead of being stopped by something else.<br>***Has there been a time when you started to do something to try to end your life but you stopped yourself before you actually did anything?***<br>If yes, describe:	**Yes No**<br>☐ ☐<br>Total # of aborted<br>______
**Preparatory Acts or Behavior:**<br>Acts or preparation towards imminently making a suicide attempt. This can include anything beyond a verbalization or thought, such as assembling a specific method (e.g., buying pills, purchasing a gun) or preparing for one's death by suicide (e.g., giving things away, writing a suicide note).<br>***Have you taken any steps towards making a suicide attempt or preparing to kill yourself (such as collecting pills, getting a gun, giving valuables away or writing a suicide note)?***<br>If yes, describe:	**Yes No**<br>☐ ☐
**Suicidal Behavior:**<br>Suicidal behavior was present during the assessment period?	**Yes No**<br>☐ ☐
**Suicide:**	**Yes No**<br>☐ ☐
Answer for Actual Attempts Only	Most Lethal Attempt Date:
**Actual Lethality/Medical Damage:**<br>0. No physical damage or very minor physical damage (e.g., surface scratches).<br>1. Minor physical damage (e.g., lethargic speech; first-degree burns; mild bleeding; sprains).<br>2. Moderate physical damage; medical attention needed (e.g., conscious but sleepy, somewhat responsive; second-degree burns; bleeding of major vessel).<br>3. Moderately severe physical damage; *medical* hospitalization and likely intensive care required (e.g., comatose with reflexes intact; third-degree burns less than 20% of body; extensive blood loss but can recover; major fractures).<br>4. Severe physical damage; *medical* hospitalization with intensive care required (e.g., comatose without reflexes; third-degree burns over 20% of body; extensive blood loss with unstable vital signs; major damage to a vital area).<br>5. Death	*Enter Code*<br>______
**Potential Lethality: Only Answer if Actual Lethality=0**<br>Likely lethality of actual attempt if no medical damage (the following examples, while having no actual medical damage, had potential for very serious lethality: put gun in mouth and pulled the trigger but gun fails to fire so no medical damage; laying on train tracks with oncoming train but pulled away before run over).<br>0 = Behavior not likely to result in injury<br>1 = Behavior likely to result in injury but not likely to cause death<br>2 = Behavior likely to result in death despite available medical care	*Enter Code*<br>______

REFERENCES

1. Brooks BR. Natural history of ALS: symptoms, strength, pulmonary function, and disability. Neurology 1996;47:S71-81; discussion S81-72.
2. Qureshi M, Schoenfeld DA, Paliwal Y, Shui A, Cudkowicz ME. The natural history of ALS is changing: improved survival. Amyotroph Lateral Scler 2009;10:324-331.
3. Chio A, Logroscino G, Traynor BJ, et al. Global epidemiology of amyotrophic lateral sclerosis: a systematic review of the published literature. Neuroepidemiology 2013;41:118-130.
4. Miller RG, Mitchell JD, Moore DH. Riluzole for amyotrophic lateral sclerosis (ALS)/motor neuron disease (MND). Cochrane Database Syst Rev 2012;3:CD001447.
5. Gordon PH, Meininger V. How can we improve clinical trials in amyotrophic lateral sclerosis? Nat Rev Neurol 2011;7:650-654.
6. Bruijn L, Cudkowicz M, Group ALSCTW. Opportunities for improving therapy development in ALS. Amyotroph Lateral Scler Frontotemporal Degener 2014;15:169-173.
7. Nicholson KA, Cudkowicz ME, Berry JD. Clinical Trial Designs in Amyotrophic Lateral Sclerosis: Does One Design Fit All? Neurotherapeutics 2015;12:376-383.
8. Gladman M, Cudkowicz M, Zinman L. Enhancing clinical trials in neurodegenerative disorders: lessons from amyotrophic lateral sclerosis. Curr Opin Neurol 2012;25:735-742.
9. van den Berg LH, Sorenson E, Gronseth G, et al. Revised Airlie House consensus guidelines for design and implementation of ALS clinical trials. Neurology 2019;92:e1610-e1623.
10. Atassi N, Yerramilli-Rao P, Szymonifka J, et al. Analysis of start-up, retention, and adherence in ALS clinical trials. Neurology 2013;81:1350-1355.
11. Bedlack RS, Cudkowicz ME. Clinical trials in progressive neurological diseases. Recruitment, enrollment, retention and compliance. Front Neurol Neurosci 2009;25:144-151.
12. Bedlack RS, Pastula DM, Welsh E, Pulley D, Cudkowicz ME. Scrutinizing enrollment in ALS clinical trials: room for improvement? Amyotroph Lateral Scler 2008;9:257-265.
13. Kaufmann P, O'Rourke PP. Central institutional review board review for an academic trial network. Acad Med 2015;90:321-323.
14. Paganoni S, Cudkowicz M, Berry JD. Outcome measures in amyotrophic lateral sclerosis clinical trials. Clin Investig (Lond) 2014;4:605-618.
15. Benatar M, Boylan K, Jeromin A, et al. ALS biomarkers for therapy development: State of the field and future directions. Muscle Nerve 2016;53:169-182.
16. Saville BR, Berry SM. Efficiencies of platform clinical trials: A vision of the future. Clin Trials 2016;13:358-366.
17. Woodcock J, LaVange LM. Master Protocols to Study Multiple Therapies, Multiple Diseases, or Both. N Engl J Med 2017;377:62-70.
18. Bateman RJ, Benzinger TL, Berry S, et al. The DIAN-TU Next Generation Alzheimer's prevention trial: Adaptive design and disease progression model. Alzheimers Dement 2017;13:8-19.
19. Beck M, Giess R, Wurffel W, Magnus T, Ochs G, Toyka KV. Comparison of maximal voluntary isometric contraction and Drachman's hand-held dynamometry in evaluating patients with amyotrophic lateral sclerosis. Muscle Nerve 1999;22:1265-1270.

20. Finkelstein DM, Schoenfeld DA. Combining mortality and longitudinal measures in clinical trials. Stat Med 1999;18:1341-1354.
21. Healy BC, Schoenfeld D. Comparison of analysis approaches for phase III clinical trials in amyotrophic lateral sclerosis. Muscle Nerve 2012;46:506-511.
22. Berry JD, Miller R, Moore DH, Cudkowicz ME, van den Berg LH, Kerr DA, Dong Y, Ingersoll EW, Archibald D. The Combined Assessment of Function and Survival (CAFS): a new endpoint for ALS clinical trials. Amyotroph Lateral Scler Frontotemporal Degener 2013;14:162-168.
23. Geronimo A., Simmons Z. Evaluation of remote pulmonary function testing in motor neuron disease. Amyotrophic Lateral Sclerosis and Frontotemporal Degeneration, 2019; 20: 348–355).

MASTER STATISTICAL ANALYSIS PLAN (M-SAP)

**Title**	Platform Trial for the Treatment of Amyotrophic Lateral Sclerosis (ALS): A perpetual multi-center, multi-regimen, clinical trial evaluating the safety and efficacy of investigational products for the treatment of ALS
**Regulatory Sponsor**	Merit E. Cudkowicz, MD
**Current Protocol Version**	5.0
**Current Protocol Date**	15 Dec 2022
**Statistical Analysis Plan Version**	3.0
**Statistical Analysis Plan Date**	06 Feb 2023

SAP APPROVAL SIGNATURES

Merit E. Cudkowicz, MD, MSc Date
Principal Investigator and Regulatory Sponsor

Sabrina Paganoni, MD, PhD Date
Co-Principal Investigator

Ben Saville, PhD Date
Senior Statistical Scientist, Berry Consultants

Eric A. Macklin, PhD Date
Study Biostatistician

SAP REVISION HISTORY

Version	Date	Description of Changes
1.0	24 Jun 2020	Initial version
2.0	01 Aug 2022	Revision of Section 5.2 SVC to specify that a minimum of 2 completed SVC maneuvers are required to calculate an estimate of maximum SVC for a given participant visit. A single maximum vital capacity reported by a pulmonary function lab and obtained by either a slow or forced maneuver is also acceptable.
3.0	06 Feb 2023	Revision of Section 1.0 Governing Documents to reference Master Protocol version 5.0.<br>Revision of Section 2.3 Study Population to update eligibility criteria.<br>Revision of Section 2.5 Regimen Allocation to specify additional stratification by use vs. non-use of sodium phenylbutyrate/taurursodiol at the time of screening for the Master Protocol.<br>Revision of Section 2.6 Treatment Allocation to specify additional stratification by use vs. non-use of sodium phenylbutyrate/taurursodiol at the time of screening for the Master Protocol.<br>Revision of Section 2.9 Master Protocol Schedule of Assessments to align with Master Protocol version 5.0.<br>Revision of Section 3.6 Missing Data to specify that mean imputation of missing baseline covariates occurs after transformation, where applicable.<br>Revision of Section 4 Study Endpoints to align with Master Protocol version 5.0.<br>Revision of Section 5.1 ALSFRS-R to specify details of the calculation of pre-baseline slope.<br>Revision of Section 5.2 SVC to specify details of the calculation of normal values.<br>Revision of Section 5.3 HHD and Grip Strength to define HHD0[2] and to specify that HHD0 and HHD0[2] are composite endpoints with death or death-equivalent.<br>Removal of former Section 5.4 Voice Characteristics to align with Master Protocol version 5.0.<br>Revision of Section 5.4 Biofluid Biomarkers to specify the assay techniques used to quantify serum creatinine and serum and CSF NfL

Version	Date	Description of Changes
		and to specify that levels of serum and CSF NfL will be log-transformed in all analyses.<br>Revision of Section 5.5 Patient-reported Outcomes to align with Master Protocol version 5.0.<br>Revision of Section 5.6 Survival to add an additional survival endpoint of death alone, to specify that the date of PAV initiation, where applicable, will be imputed as the fifteenth day of a month if not specified more precisely, and to specify that time at risk begins at each participant's Baseline Visit and ends at the Week 24 Visit if completed or at the earlier of the end of the Week 24 Visit window or date last known alive if a Week 24 visit is not completed.<br>Addition of Section 5.7 King's ALS Clinical Staging System to specify determination of King's stage.<br>Addition of Section 5.8 Clinical Safety Laboratory Tests to specify clinical safety laboratory tests and derivation of measures of drug-induced liver injury.<br>Revision of Section 6.1 Analysis Sets to define the Full Analysis Set, to add the Efficacy Common Mode of Administration (ECM) analysis set, and to specify the time point at which data is excluded from the Efficacy Per-protocol analysis set.<br>Revision of Section 6.2 Baseline Characteristics to include additional baseline characteristics.<br>Revision of Section 6.3 Primary Efficacy Analysis and Supportive Analyses to specify the estimand.<br>Revision of Section 6.4 Interim Analysis to specify that the default design for a regimen will include stopping early only for futility.<br>Revision of Section 6.5 Hierarchical Testing to fully specify the secondary endpoints.<br>Revision of Section 6.5.2 Repeated-measures Model to add a fixed term for treatment group (removing the shared-baseline assumption at the recommendation of the FDA), to add fixed terms for baseline use of sodium phenylbutyrate/taurursodiol, baseline serum NfL level, and their interactions with visit, and to specify the estimand.<br>Revision of Section 6.5.3 Random-slopes Model to add a fixed term for treatment group (removing the shared-baseline assumption at the recommendation of the FDA), to add fixed terms for baseline serum NfL level, baseline use of sodium phenylbutyrate/taurursodiol, and their interactions with visit, and to specify the estimand.

Version	Date	Description of Changes
		Revision of Section 6.5.4 Survival to specify the analysis sets, to include baseline age, baseline serum NfL level, and baseline use of sodium phenylbutyrate/taurursodiol as additional covariates in adjusted models.<br><br>Revision of Section 6.5.5 CAFS to provide additional detail on the calculation of the rank-sums and to specify analyses of additional survival endpoints, functional endpoints, analysis methods.<br><br>Revision of Section 6.5.6 HHD0 and HHD0[2] to specify that analysis requires methods for interval-censored data and to add baseline serum NfL level and baseline use of sodium phenylbutyrate/taurursodiol as additional covariates,<br><br>Revision of Section 6.5.7 Placebo Multiple Imputation to specify analysis primary and secondary functional endpoints, to add baseline serum NfL level and baseline use of sodium phenylbutyrate/taurursodiol as additional covariates, and to revise specification of template SAS code,<br><br>Revision of Section 6.5.9 Subgroup Analyses to add subgroups, specify how rare subgroup levels should be handled, and specify the estimates to be obtained.<br><br>Revision of Section 6.5.10 Comparison of Controls across Regimens to add baseline serum NfL level and baseline use of sodium phenylbutyrate/taurursodiol as additional covariates.<br><br>Revision of Section 6.6.1 Treatment-emergent Adverse Events to define treatment-emergence, to include summaries of fatal TEAEs, to include summary of TEAEs observed in at least 5% of participants, to specify summaries of TEAEs by subgroups, to indicate that treatment-dependent differences in TEAE incidence rates rather than rate ratios will be estimated, and to remove testing of treatment-dependent differences in the distribution of TEAE characteristics.<br><br>Revision of Section 6.6.2 Safety Labs to specify that clinical safety laboratory abnormalities will be summarized by CTCAE toxicity grade and to include an aggregate summary of abnormalities observed over all post-baseline visits in shift tables.<br><br>Revision of Section 6.6.4 Vital Signs and Weight to clarify the analysis sets and analysis methods used to analyze change in weight.<br><br>Revision of Section 6.7.1 Participant Disposition to specify more completely the summaries of participant disposition.<br><br>Revision of Section 6.7.2 Study Drug Compliance Exposure and Tolerance to restrict summaries to the ERO and SRO analysis sets and

Version	Date	Description of Changes
		to change measures of exposure from counts of doses to durations of exposure.<br>Addition of Section 6.7.4 Medical History to specify summaries of medical history data.<br>Addition of Section 6.7.5 Blindedness to specify summaries and analyses of blindedness assessments.<br>Addition of Section 6.7.6 Protocol Deviations to specify summaries of protocol deviations.<br>Revision of Section 8 References to add new references and remove references that are no longer cited.<br>Addition of Appendix 1 ALS Master Protocol Recommended Statistical Analysis, Design and Simulation Report; and a Summary of Changes from the previous version, dated May 14, 2021.

Table of Contents

1. Governing Documents 8
2. Study Design 8
   2.1 Overview 8
   2.2 Study Objectives 8
   2.3 Study Population 9
   2.4 Participant Flow 9
   2.5 Regimen Allocation 9
   2.6 Treatment Allocation 10
   2.7 Treatment Administration 10
   2.8 Allocation Concealment 10
   2.9 Master Protocol Schedule of Assessments 10
3. General Considerations for Data Analysis 12
   3.1 Statistical Software 12
   3.2 Summary Statistics 12
   3.3 Precision 12
   3.4 Transformations 12
   3.5 Multiplicity Adjustments 12
   3.6 Missing Data 12
4. Study Endpoints 13
   4.1 Efficacy Endpoints 13
   4.2 Exploratory Efficacy Endpoints 13
   4.3 Safety Endpoints 13
5. Measurement Definitions 13
   5.1 ALSFRS-R 13
   5.2 SVC 14
   5.3 HHD and Grip Strength 14
   5.4 Biofluid Biomarkers 15
   5.5 Patient-reported Outcomes 15
   5.6 Survival 15
   5.7 King's ALS Clinical Staging System 15
   5.8 Clinical Safety Laboratory Tests 16
6. Statistical Methodology 17
   6.1 Analysis Sets 17
   6.2 Baseline Characterization 18
   6.3 Primary Efficacy Analysis and Supportive Analyses 18
   6.4 Interim Analysis 19
   6.5 Secondary Efficacy Analyses 19
      6.5.1 Hierarchical Testing 19
      6.5.2 Repeated-measures Model 19
      6.5.3 Random-slopes Model 21
      6.5.4 Survival 23
      6.5.5 CAFS 23
      6.5.6 HHD0 and HHD0[2] 24
      6.5.7 Placebo Multiple Imputation 24
      6.5.8 Additional Sensitivity Analyses of Primary and Key Secondary Outcomes 25
      6.5.9 Subgroup Analyses 25
      6.5.10 Comparison of Controls across Regimens 26
   6.6 Safety Analyses 27
      6.6.1 Treatment-emergent Adverse Events 27
      6.6.2 Safety Labs 28
      6.6.3 ECG Results 28
      6.6.4 Vital Signs and Weight 28
      6.6.5 Suicidality 28
   6.7 Other Analyses 28

6.7.1 Participant Disposition 28
6.7.2 Study Drug Compliance and Tolerance 29
6.7.3 Concomitant Medication Use 29
6.7.4 Medical History 29
6.7.5 Blindedness 30
6.7.6 Protocol Deviations 30
7. Validation 30
7.1 Primary Efficacy Analysis 30
7.2 Secondary, Exploratory, and Safety Analyses 30
8. References 30
9. Appendix I 32

1. Governing Documents

This Master Statistical Analysis Plan (M-SAP) defines the default outcome measures, analysis samples, and planned analyses for the placebo-controlled portion of regimens tested in the HEALEY ALS Platform Trial for Regimen-specific Appendices (RSAs) initiated after implementation of version 5.0 of the HEALEY ALS Platform Trial Master Protocol in February 2023. RSAs initiated on earlier versions of the HEALEY ALS Platform Trial Master Protocol will refer to version 2.0 of the M-SAP dated 06 Mar 2020. Each RSA will have a regimen-specific SAP (R-SAP) that specifies any deviations or further details that supersede the specifications given here. The M-SAP and each R-SAP supplement the Master Protocol, the "ALS Master Protocol Recommended Statistical Analysis, Design and Simulation Report" (MPRDR, Appendix 1 to this M-SAP), and the applicable RSA. Please refer to the Master Protocol and applicable RSA for details on the rationale for the study design, eligibility criteria, conduct of the trial, clinical assessments and schedule of assessments, definitions and reporting of adverse events, data management conventions, and regulatory oversight and compliance procedures. The MPRDR and any regimen-specific deviations described in the applicable RSA and R-SAP are authoritative in defining the primary and interim analyses. In case of discrepancies between either SAP and the Master Protocol and applicable RSA concerning other matters of analysis, the M-SAP and applicable R-SAP are authoritative. In case of discrepancies between the M-SAP and R-SAP, the R-SAP is authoritative. On all other matters, the Master Protocol and applicable RSA are authoritative.

2. Study Design

2.1 Overview

The HEALEY ALS Platform Trial is a perpetual multi-center, multi-regimen clinical trial evaluating the safety and efficacy of investigational products for the treatment of ALS. Investigational products will be evaluated according to details specified in distinct RSAs. Each RSA will describe the nature of the intervention and its mechanism of action, including the mode and frequency of administration, number of doses, the specific target population (to be selected within the pre-defined subsets of the Master Protocol), additional enrollment criteria (if any), sample size, and other specific intervention-related information and assessments (safety or other assessments that may be in addition to those outlined in the Master Protocol).

2.2 Study Objectives

Primary Efficacy Objective:

- To evaluate the efficacy of multiple investigational products as compared to placebo on ALS disease progression.

Secondary Efficacy Objectives:

- To evaluate the effect of multiple investigational products on selected secondary measures of disease progression.

Safety Objectives:

- To evaluate the safety of multiple investigational products for people with ALS.

Exploratory Efficacy Objectives:

- To evaluate the effect of multiple investigational products on selected biomarkers and endpoints.

Each regimen will test the efficacy of a distinct treatment.

2.3 Study Population

Individuals eligible for trial participation are men or women at least 18 years old who meet the El Escorial criteria of possible, laboratory-supported probable, probable, or definite for a diagnosis of sporadic or familial ALS, report onset of weakness within 36 months of screening, possess a slow vital capacity (SVC) at least 50% of that expected based on age, sex, and height, are treatment naïve or on a stable dose of riluzole, edaravone, or both, are naïve to or have started treatment with sodium phenylbutyrate/taurursodiol at least 30 days prior to Master Protocol Screening, lack unstable clinical or psychiatric conditions, and were not recently exposed to other investigational treatments or prior gene therapy. Detailed inclusion and exclusion criteria are specified in the Master Protocol and applicable RSA.

Participants will be recruited from approximately 60 centers located throughout the US that are part of the Northeast ALS (NEALS) Consortium.

2.4 Participant Flow

Participants that consent to the Master Protocol will be screened for Master Protocol-level inclusion and exclusion criteria. Those determined eligible will be randomly assigned to an applicable regimen. Participants that consent to their assigned regimen will be screened for any regimen-specific inclusion and exclusion criteria. Those determined eligible will be randomized to either active or placebo treatment for their assigned regimen.

This perpetual platform trial will continue enrolling research participants for as long as there are regimens that are enrolling. Enrollment into a specific regimen will continue until the target number of randomized participants in a regimen has been reached unless stopped earlier due to a determination of regimen success or futility.

Each regimen will compare double-blind active and placebo treatment groups for a maximum of up to 28 weeks or as otherwise specified in the RSA. Participants will complete a baseline visit prior to initiating study drug. After the baseline visit, participants will be seen in clinic at 4, 8, 16, and 24 weeks and will complete telephone visits at 2, 12, and 20 weeks and 28 days after last dose of study drug or as otherwise specified in the RSA.

An active treatment extension (ATE) will be available to participants who complete the double-blind phase as described in their respective RSAs. Participants may also opt to re-screen for the Master Platform and enroll in a second regimen, if eligible.

Detailed descriptions of study procedures and timing are specified in the Master Protocol and applicable RSA.

2.5 Regimen Allocation

Participants determined eligible for Master Protocol-level inclusion and exclusion criteria will be randomized with equal probability to all applicable regimens. Applicable regimens are those for

which the participant's site is active and for which the participant is not known to be ineligible, e.g., due to prior screen failure. Randomizations are stratified for all combinations of use vs. non-use of riluzole, edaravone, and sodium phenylbutyrate/taurursodiol at the time of screening for the Master Protocol. Details of regimen assignment are described in the Platform Trial Regimen Assignment Plan.

2.6 Treatment Allocation

Prior to the baseline visit, eligible participants for a given regimen will be randomly allocated in a 3:1 ratio to active or placebo treatment. Active participants may be further divided at random to more than one dose group, if applicable. Randomizations follow a permuted-block schedule, stratified for all combinations of use vs. non-use of riluzole, edaravone, and sodium phenylbutyrate/taurursodiol at the time of screening for the Master Protocol. The randomization schedule will be prepared by computer program by an unblinded study statistician who will not contribute to decisions concerning the course, conduct, or analysis of the HEALEY ALS Platform Trial.

2.7 Treatment Administration

Details of treatment administration are described in the applicable RSA.

2.8 Allocation Concealment

Allocation to regimen is not concealed. Allocation to treatment groups within a regimen is concealed to participants and investigators. The regimen randomization schedule is known only to the unblinded study statistician who generated the schedule, any unblinded staff responsible for managing the interactive response technology (IRT) used for randomization, all staff at the central pharmacy responsible for distribution of study drug, and the unblinded Independent Statistical Analysis Committee (ISAC) responsible for interim analyses. An independent Data and Safety Monitoring Board (DSMB) will receive unblinded data as described in the DSMB Charter. Concealment of the true regimen treatment assignment of specific participants is achieved by using anonymous study drug kit identifiers linked at time of dispensation to participants. Member of the HEALEY ALS Platform Trial Executive Committee, members of the Regimen Steering Committee, site investigators and other site staff including research pharmacists, clinical coordination and data management staff, the medical monitor, and all participants are blinded to regimen treatment assignments of participants. Additional details concerning blinding are described in Data Sharing Plan and the Clintrex/CMSU Firewall Plan.

2.9 Master Protocol Schedule of Assessments

Activity	Master Protocol Screening[1]	Regimen Specific Screening[1]	Baseline	Week 2	Week 4[13]	Week 8[13]	Week 12	Week 16[13]	Week 20	Week 24	Follow-Up Safety Call
	Clinic	Clinic	Clinic	Phone	Clinic	Clinic	Phone	Clinic	Phone	Clinic	Phone
	-42 to -1 Days	Refer to RSA	Day 0	Day 14 ±3	Day 28 ±7	Day 56 ±7	Day 84 ±3	Day 112 ±7	Day 140 ±3	Day 168 ±7	Refer to RSA
Written Informed Consent[2]	X	X									
Inclusion/Exclusion Review	X	X[3]									
Regimen Specific Screening procedure(s)		X									

Activity	Master Protocol Screening[1]	Regimen Specific Screening[1]	Baseline	Week 2	Week 4[13]	Week 8[13]	Week 12	Week 16[13]	Week 20	Week 24	Follow-Up Safety Call
	Clinic	Clinic	Clinic	Phone	Clinic	Clinic	Phone	Clinic	Phone	Clinic	Phone
	-42 to -1 Days	Refer to RSA	Day 0	Day 14 ±3	Day 28 ±7	Day 56 ±7	Day 84 ±3	Day 112 ±7	Day 140 ±3	Day 168 ±7	Refer to RSA
ALS & Medical History	X										
Demographics	X										
Physical Examination	X										
Neurological Exam	X										
Vital Signs[4]	X		X		X	X		X		X	
Slow Vital Capacity	X[12]		X			X		X		X	
Muscle Strength Assessment			X			X		X		X	
ALSFRS-R	X		X		X	X	X	X	X	X	
Patient Reported Outcomes[10]			X			X		X		X	
12-Lead ECG	X									X	
Clinical Safety Labs[5]	X		X		X	X		X		X	
Concomitant Medication Review	X	X	X		X	X	X	X	X	X	X
Adverse Event Review[6]	X	X	X	X	X	X	X	X	X	X	X
Columbia-Suicide Severity Rating Scale			X		X	X		X		X	
Biomarker Blood Collection			X			X		X		X	
Biomarker Urine Collection			X			X		X		X	
DNA Collection[7] (optional)			X								
CSF Collection[11]			X					X			
Assignment to a regimen	X										
Randomization within a regimen		X									
Administer/Dispense Study Drug			X[8]			X		X			
Study Drug Accountability/Compliance				X[14]	X	X	X[14]	X	X[14]	X	
Exit Questionnaire										X	
Vital Status Determination										X[9]	

[1] Master Protocol Screening procedures must be completed within 42 days to 1 day prior to the Baseline Visit. Refer to RSA for details about RSA Specific Screening procedures, if any, should be performed.
[2] During the Master Protocol Screening Visit, participants will be consented via the Master Protocol informed consent form (ICF). After a participant is assigned to a regimen, participants will be consented a second time via the regimen-specific ICF.
[3] At the Regimen Specific Screening Visit, participants will have regimen-specific inclusion and exclusion criteria assessed, if applicable.
[4] Vital signs include weight, systolic and diastolic pressure, respiratory rate, heart rate and temperature. Height in cm measured at Master Protocol Screening Visit only.
[5] Clinical safety labs include hematology (CBC with differential), complete chemistry panel, liver function tests, thyroid function and urinalysis. Coagulation panel will occur at the Master Protocol Screening and Week 16 Visits or as in the RSA. Serum pregnancy testing will occur in women of child-bearing potential at the Master Protocol Screening Visit and as necessary during the study. Pregnancy testing is only repeated as applicable if there is a concern for pregnancy.
[6] Adverse events that occur after signing Master Protocol consent form will be recorded.
[7] The DNA sample can be collected after the Baseline Visit if a baseline sample is not obtained or the sample is not usable.
[8] Administer first dose of study drug only after Baseline Visit procedures are completed
[9] Vital status, defined as a determination of date of death or death equivalent or date last known alive, will be determined for each randomized participant at the end of the placebo-controlled period of their follow-up (generally the Week 24 Visit, as indicated). If at that time the participant is alive, his or her vital status should be determined again at the time of the last participant's last visit (LPLV) of the placebo-controlled period of a given regimen. We may also ascertain vital status at later time points by using publicly available data sources as described in section 8.15.b
[10] Each RSA will detail which PRO is collected at each Visit. Specific PRO collection may differ from this Schedule of Activities.
[11] CSF collection may differ from the Schedule of Activities and will be performed as described in each RSA.
[12] If required due to pandemic-related or other restrictions, Forced Vital Capacity (FVC) performed by a Pulmonary Function Laboratory evaluator may be used for eligibility (Master Protocol Screening ONLY).
[13] Visit may be conducted via phone or telemedicine with remote services instead of in-person if needed to protect the safety of the participant due to a pandemic, disability or other medical reason.

[14] Drug accountability will not be done at phone visits. A drug compliance check in will be conducted during phone visits to ensure participant is taking drug per dose regimen and to note any report of missed doses.

3. General Considerations for Data Analysis

3.1 Statistical Software

Statistical analyses will be performed using SAS (SAS Institute, NC, USA), R (R Foundation for Statistical Computing, Vienna, Austria), or JAGS (Plummer, SourceForge).

3.2 Summary Statistics

Data will be summarized with respect to disposition, demographics, pre-treatment characteristics, efficacy endpoints, safety endpoints, and tolerability. Summary statistics for continuous variables will include the number of observations, the mean, median, standard deviation, inter-quartile range, and range. Summaries of categorical data will include counts, denominators, and percentages.

3.3 Precision

Results will generally be reported to 3 significant figures. Percentages will generally be reported to 0.1 percentage points. P-values will generally be reported to two digits when greater than or equal to 0.095, to three digits when greater than or equal to 0.00095 and less than 0.095, and as <0.001 for all smaller values.

3.4 Transformations

Data that are strictly positive, continuous, and strongly right skewed will typically be log-transformed prior to any inferential testing. Skewness greater than 3 will be used as a guide in determining which variables to transform. Which variables warrant transformation and how they will be transformed will be determined prior to regimen data lock. Original, untransformed values will be used for all summaries. Any data transformations used in the primary and interim analyses are described in the MPRDR (Appendix 1 to this M-SAP).

3.5 Multiplicity Adjustments

A single primary analysis is planned. The default criterion for significance at the final analysis is adjusted to ensure an overall one-sided type 1 error rate less than 0.025 in the absence of early stopping for futility. Secondary efficacy endpoints will be tested in a pre-specified order using a closed testing procedure to maintain an overall two-tailed family-wise error rate less than 0.05 under the condition that the primary and all secondary analyses are null. Nominal comparison-wise p-values will also be reported for all analyses unless otherwise specified.

3.6 Missing Data

If baseline values of a given measure are missing, the last observed pre-treatment value will be used. Missing baseline covariates will be imputed using the mean of the respective covariate. For any baseline covariate that is transformed for analysis, means will be imputed after transformation. Off-schedule results, e.g., those collected as part of an Early Termination Visit, will be used in place of the closest missing scheduled visit (while preserving visit sequence) for analyses that depend on visit-specific data. Other results will not be carried forward.

The planned mixed model analyses, where applied, yield estimates that are unbiased conditional on the observed values under a missing at random assumption. Secondary analyses of efficacy endpoints that accommodate missing values are described below (see Sections 6.5.5 and 6.5.6).

4. Study Endpoints

4.1 Efficacy Endpoints

The primary efficacy endpoint is change from baseline through Week 24 in disease severity as measured by the ALS Functional Rating Scale-Revised (ALSFRS-R) total score and survival.

Secondary efficacy endpoints include change from baseline through Week 24 in muscle strength as measured isometrically using hand-held dynamometry (HHD) and grip strength, respiratory function as assessed by slow vital capacity (SVC), and survival.

4.2 Exploratory Efficacy Endpoints

The following categories of exploratory endpoints will be evaluated:

- Biofluid biomarkers
- Patient-reported outcomes

4.3 Safety Endpoints

The following safety endpoints will be evaluated:

- Treatment-emergent adverse and serious adverse events.
- Changes in laboratory values and treatment-emergent and clinically significant laboratory abnormalities.
- Changes in ECG parameters and treatment-emergent and clinically significant ECG abnormalities.
- Treatment-emergent suicidal ideation and suicidal behavior.

Reported proportions will use as their denominator all participants in the applicable Safety sample (see Section 6.1 below).

5. Measurement Definitions

5.1 ALSFRS-R

The ALSFRS-R (Cedarbaum et al. 1999) is a 12-item instrument completed by clinician interview for assessing participants' function in four domains: bulbar, fine motor, gross motor, and respiratory. Each item is scored from 0 to 4 with higher scores indicating greater function. The ALSFRS-R total score is the sum of all items (range 0 to 48). Domain scores are the sum of their respective items (range 0 to 12): bulbar function = Q1-Q3, fine motor function = Q4-Q6, gross motor function = Q7-Q9, and respiratory function = QR1-QR3 (Q10-Q12).

The average rate of disease progression prior to the Baseline Visit as measured by ALSFRS-R (pre-baseline slope of ALSFRS-R or delta-FRS) will be calculated as 48 minus the ALSFRS-R

total score measured at the Baseline Visit, that difference divided by the number of months from onset of symptom weakness due to ALS to the date of the Baseline Visit. The number of months will be calculated as the difference in days from onset of symptomatic weakness to the Baseline Visit multiplied by 12 / 365.25. The date of onset of symptomatic weakness will be imputed as the fifteenth day of a month if not specified more precisely.

5.2 SVC

Slow vital capacity (SVC) is the maximum volume of air that can be slowly exhaled after slow, maximal inhalation. Trained technicians coach participants through 3 to 5 maneuvers using a spirometer. A minimum of 2 completed SVC maneuvers are required to calculate an estimate of maximum SVC for a given participant visit. A single maximum vital capacity reported by a pulmonary function lab and obtained by either a slow or forced maneuver is also acceptable. The maximum volume expired is converted to percent of predicted normal using normal values for forced vital capacity (FVC). FVC normal values are calculated based on sex, age, height, and ethnicity using equations published by the Global Lung Function Initiative (GLI, Quanjer et al. 2012). Higher values indicate greater respiratory function.

Age will be calculated as number of days from date of birth to the date of a given SVC assessment divided by 365.25. The following correspondence between self-identified race and race defined by GLI classification will be used:

Self-identified Race	GLI-defined Race
American Indian or Alaska Native	Mixed/Other
Asian	South East Asian
Black or African American	African American
Native Hawaiian or Other Pacific Islander	Mixed/Other
White	Caucasian
Unknown	Caucasian
Not reported	Caucasian
More than one race indicated	Mixed/Other

5.3 HHD and Grip Strength

Manual muscle testing by hand-held dynamometry (HHD) assesses isometric muscle strength by recording force generated against a hand-held dynamometer placed against a given limb by a trained evaluator. Each muscle is tested with a standard beginning position, usually midway between maximum flexion or extension of the muscle being studied, and with the dynamometer placed at a defined position. Trained evaluators coach participants through 3 to 5 maximal exertions for each muscle and record the maximum force observed in pounds. Evaluators note if participants are not able to overcome the force exerted for a given muscle. Conversely, a value of 0 is recorded for a given muscle if the participant cannot assume the testing position due to weakness or can attain the position but cannot exert measurable force.

The following 11 muscles are tested bilaterally by HHD: shoulder flexion, elbow flexion, elbow extension, wrist extension, abductor pollicis brevis contraction, abductor digiti minimi contraction, first dorsal interosseous contraction, hip flexion, knee flexion, knee extension, and ankle dorsiflexion. Grip strength is tested bilaterally by Jamar hand dynamometer. Between HHD and hand grip, 24 muscles or muscle groups are tested. The force recorded for each muscle

is converted to a percentage change from baseline among muscles with non-zero strength at baseline. Individual percentages are averaged together to yield upper extremity, lower extremity, and global average percentages. Higher values indicate greater strength retention.

HHD data will also be summarized as time to loss of strength. HHD0 and HHD0[2] will be defined as the times from the Baseline Visit to the first and second post-baseline occurrence of a muscle with a strength recording of 0, respectively, among those muscles that were non-zero at baseline. All 11 muscles assessed by HHD as well as grip strength will be considered. Both HHD0 and HHD0[2] are composite endpoints with death or death-equivalent, censored at the Week 24 Visit if completed or at the earlier of the end of the Week 24 Visit window or date last known alive if a Week 24 visit is not completed. Larger HHD0 and HHD0[2] times indicate longer preservation of strength.

5.4 Biofluid Biomarkers

Blood and urine biomarkers will be assayed. Biomarkers will include serum creatinine and serum and cerebrospinal fluid (CSF) neurofilament light chain (NfL). Serum creatinine will be assayed by the kinetic Jaffe method (test 001370, Labcorp, Burlington, NC). NfL will be assayed by single-molecule array (Simoa; Quanterix, Billerica, MA). Levels of serum and CSF NfL that are reported to be below the limit of quantitation will be imputed at the limit of quantitation. Levels of serum and CSF NfL will be log-transformed in all analyses. Other biomarkers may be analyzed.

5.5 Patient-reported Outcomes

Patient-reported outcomes may be included as regimen-specific assessments. The description and scoring of each instrument will be specified in the R-SAP.

5.6 Survival

The primary survival endpoint will be the composite of death or death equivalent. A secondary survival endpoint of death alone independent of any death equivalent (including a death equivalent that occurs prior to baseline) is also defined. Death equivalent will be defined as use of permanent assisted ventilation (PAV), where PAV is defined as more than 22 hours per day of noninvasive or invasive mechanical ventilation for more than seven consecutive days. The date of PAV initiation is the first day of the consecutive days. The date of PAV initiation, where applicable, will be imputed as the fifteenth day of a month if not specified more precisely. Any participant on PAV at baseline will be censored at baseline for the composite survival endpoint.

Time at risk for both the composite survival endpoint of death or death equivalent and the survival endpoint of death alone will be measured from each participant's Baseline Visit and will be censored at the Week 24 Visit if completed or at the earlier of the end of the Week 24 Visit window or date last known alive if a Week 24 visit is not completed.

Additional secondary analyses of survival may consider different durations of follow-up.

5.7 King's ALS Clinical Staging System

The King's ALS Clinical Staging System (Roche et al. 2012) is a 4-level ordinal scale with the first three levels indicating the number (1, 2, or 3) of distinct central nervous system regions

(bulbar, upper limb, and lower limb) with neuromuscular dysfunction and levels 4a and 4b indicating nutritional or respiratory failure secondary to ALS, respectively.

Participants will be classified to King's stage 1, 2, 3, 4a, or 4b based on scores from ALSFRS-R assessments according to a published derivation (Balendra et al. 2014). Bulbar involvement is defined as a score less than 4 on any of the ALSFRS-R questions in the bulbar domain (questions 1, 2, and 3). Upper limb involvement is defined as a score less than 4 on either of the ALSFRS-R questions related to hand function (questions 4 and 5A). Lower limb involvement is defined as a score less than 4 on the ALSFRS-R question about walking (question 8). Nutritional failure is defined as responding that the participant uses gastrostomy for greater than 50% of their nutrition. Respiratory failure is defined as a score of 0 on the ALSFRS-R question addressing dyspnea (question 10 or R-1) or a score less than 4 on the ALSFRS-R question about use of mechanical ventilation (question 12 or R-3). Participants without evidence by ALSFRS-R scores of involvement of any of the three central nervous system regions will be scored as King's stage 1 due to their confirmed diagnosis with ALS. Participant may meet criteria for both King's stage 4a and 4b.

5.8 Clinical Safety Laboratory Tests

Clinical safety labs include hematology, blood chemistry panel, liver function tests, thyroid function, urinalysis, and pregnancy testing in women of childbearing potential as specified in Section 9.1.2 Clinical Safety Laboratory Tests of the Master Protocol:

- Hematology with differential panel: complete blood count with differential (hematocrit, hemoglobin, platelet count, RBC indices, Total RBC, Total WBC, and WBC & differential)
- Blood chemistry panel/Liver function tests (LFTs): alanine aminotransferase (ALT (SGPT)), aspartate aminotransferase (AST (SGOT)), albumin, alkaline phosphatase, bicarbonate, blood urea nitrogen, calcium, chloride, creatinine (estimated Glomerular Filtration Rate [eGFR] will be calculated using the MDRD equation and creatinine clearance will be calculated using the Cockcroft-Gault equation), glucose, magnesium, phosphate, potassium, sodium, thyroid-stimulating hormone (TSH), total bilirubin and total protein
- Urinalysis: albumin, bilirubin, blood, clarity, color, glucose, ketones, nitrate, pH, protein, specific gravity, urobilinogen and WBC screen
- Serum human chorionic gonadotrophin (hCG) for WOCBP (collected only at Master Protocol Screening Visit, and as necessary throughout course of study)
- Coagulation parameters: Prothrombin time, International normalized ratio, Activated partial thromboplastin time

Clinical safety labs will also include derived measures of potential drug-induced liver injury (DILI), including those that potentially meet the Hy's law criteria, as distinct safety lab outcomes.

Three potential DILI criteria will be defined:

- ALT or AST >3x ULN with TBL >1.5x ULN

- AST or ALT >3x ULN with TBL >2x ULN
- AST or ALT >3x ULN with TBL >2x ULN and ALP <2x ULN (potential Hy's Law cases)

where ULN is upper level of normal and all levels are measured on the same day.

6. Statistical Methodology

6.1 Analysis Sets

The following analysis sets will be used for testing efficacy, safety, and tolerability endpoints of a specific focal regimen:

- Efficacy Full Analysis Set (FAS): Participants who were randomized within the focal regimen plus placebo participants from specified regimens who had an opportunity to complete placebo-controlled follow-up, classified according to their randomized treatment assignment. Observations made after premature permanent discontinuation of study drug are included in this sample, should such participants remain on study. Observations completed after regimen data lock will be excluded. Participants determined to not meet ALS diagnostic criteria are excluded.
- Efficacy Concurrent-controls (ECC) Set: The subset of participants in the FAS analysis set who were randomized within 180 days prior to the first randomization for the applicable regimen and within 180 days after the last randomization for the applicable regimen, exclusive.
- Efficacy Regimen-only (ERO) Set: The subset of participants in the FAS analysis set who were randomized within a given regimen.
- Efficacy Common Mode of Administration (ECM) Set: The subset of participants in the FAS analysis set who are in regimens in which study drug is administered by the same route as the focal regimen (including oral, subcutaneous injection, intravenous infusion).
- Efficacy Per-protocol (EPP) Set: The subset of participants in the FAS analysis set who initiated study treatment and who meet other requirements for protocol compliance as specified in the R-SAP, classified according to the treatment they actually received. This sample may include placebo participants from no other, some other, or all other regimens and may be restricted to those who would have been eligible for the applicable regimen as specified in the R-SAP. If a participant's data are truncated for inclusion in the EPP analysis set due to a time-dependent event (e.g., non-adherence to protocol-specified dosing, initiation of a prohibited medication, initiation of a medication approved for treatment of ALS), clinical events observed after the censoring event may be included in the EPP analysis set as specified in the R-SAP. Data from placebo participants shared from other regimens will not be truncated due to non-adherence to protocol-specified dosing.
- Safety Full (STF) Set: Participants who initiated treatment within the focal regimen plus placebo participants from specified regimens who are not known to be ineligible for the focal regimen and who initiated treatment in their respective regimen, classified according to the treatment they actually received. Observations made after premature permanent discontinuation of study drug are included in this sample, should such participants remain on study. Observations completed after regimen data lock are excluded.

- Safety Narrow (STN) Set: The subset of participants in the STF analysis set who were exposed to interventions with the same mode of administration.
- Safety Regimen-only (SRO) Set: The subset of participants in the STF analysis set who were randomized within a given regimen.

The meaning of regimen data lock and the criteria for inclusion of data for placebo participants assigned to other regimens at the time of regimen data lock will be defined in the HEALEY ALS Platform Trial Data Management Plan and the HEALEY ALS Platform Trial Data Sharing Plan.

6.2 Baseline Characterization

Each analysis sample will be summarized overall and by treatment group for the following characteristics: randomization site; age, sex, race, ethnicity, El Escorial diagnosis, months since ALS symptom onset, delay between symptom onset and ALS diagnosis, site of ALS symptom onset, use at baseline of riluzole, edaravone, or sodium phenylbutyrate/taurursodiol and combinations of use and non-use of these medications, ALSFRS-R total score, delta-FRS, SVC, King's stage, weight, body mass index (BMI), serum creatinine concentration, and serum NfL concentration.

6.3 Primary Efficacy Analysis and Supportive Analyses

Unless otherwise specified in the RSA, the primary analysis for each regimen is a Bayesian shared-parameter model of function and survival. Details of the model, including documentation of operating characteristics under a range of scenarios, are provided in the MPRDR (Appendix 1 to this M-SAP). Any regimen-specific deviations are described in the applicable RSA and R-SAP.

The estimand of the primary analysis is the relative rate of disease progression (the "disease rate ratio" or DRR) of active treatment relative to placebo in the FAS population under an assumption that active treatment slows mean time to death or death equivalent by the same proportion as treatment slows the mean rate of functional progression as measured by change in ALSFRS-R total score over time. The estimand is defined by the following attributes:

- Treatment: active treatment vs. placebo.
- Population: FAS population as defined in Section 6.1.
- Variables: time to death or death equivalent and rate of change in ALSFRS-R total score from baseline to the Week 24 Visit.
- Intercurrent event 1: treatment discontinuation due to death or death equivalent: no ALSFRS-R data from participants who reach the death or death equivalent endpoint are included in the analysis, handled via mortality component in model, composite variable strategy approach.
- Intercurrent event 2: treatment discontinuation not due to death or death equivalent: handled via treatment policy approach, all data will be used including data collected during the placebo-controlled period after treatment discontinuation regardless of concomitant medication, for those participants who have not been censored due to mortality. Missing data post-treatment will not be imputed, handled via missing at random assumption.
- Population-level summary: mean ratio of hazard or progression rate of active treatment relative to placebo.

6.4 Interim Analysis

Unless otherwise specified in the RSA, regimens will be considered for early stopping for success or futility at regular intervals. The default design for a regimen will include stopping early only for futility. Details of the interim analysis schedule, the early stopping rules, and documentation of operating characteristics under a range of scenarios, are provided in the MPRDR (Appendix 1 to this M-SAP). Any regimen-specific deviations are described in the applicable RSA and R-SAP.

6.5 Secondary Efficacy Analyses

6.5.1 Hierarchical Testing

Primary inference for secondary efficacy endpoints will be based on analysis of the FAS analysis set using a repeated-measures linear mixed model for functional endpoints (see Section 6.5.2 below) and by Kaplan-Meier product-limit estimates and log-rank test for survival (see Section 6.5.5 below). The default sequence for testing is the following:

1. Change from baseline to Week 24 in upper limb muscle strength as assessed isometrically using HHD and grip strength and measured as average percent change from baseline among maneuvers with non-zero strength at baseline in the FAS analysis set,
2. Change from baseline to Week 24 in respiratory function as assessed by slow vital capacity (SVC) and measured as percent predicted using GLI normal values in the FAS analysis set,
3. Change from baseline to Week 24 in lower limb muscle strength as assessed isometrically using HHD and measured as average percent change from baseline among maneuvers with non-zero strength at baseline in the FAS analysis set, and
4. Survival defined as freedom from death and permanent assisted ventilation (PAV) from baseline to Week 24 in the FAS analysis set.

RSAs may specify a different sequence of secondary endpoints to evaluate using a closed testing procedure or another method for controlling family-wise type 1 error rate.

If the primary analysis indicates a significant slowing in disease progression from the Bayesian shared-parameter model of function and survival, then each secondary efficacy endpoint would be declared significant in the specified sequence using a criterion of two-tailed $p < 0.05$. After the first failure to declare significance, no endpoints lower in the hierarchy can be significant. Nominal comparison-wise p-values for secondary efficacy endpoints will also be reported.

6.5.2 Repeated-measures Model

Treatment groups of a given regimen will also be compared for progression of continuous efficacy and selected safety endpoints (ALSFRS-R total score, ALSFRS-R domain scores, HHD upper extremity, lower extremity, and global average percentages, SVC percent-predicted, serum creatinine, serum NfL, and weight) using a repeated-measures linear mixed model in the FAS, ECC, ERO, ECM, and EPP samples. The model will include fixed terms for discrete visit, treatment group, treatment group × visit interaction, time since symptom onset and time since symptom onset × visit interaction, delta-FRS and delta-FRS × visit interaction, baseline log-transformed serum NfL level and baseline log-transformed serum NfL level × visit interaction, baseline riluzole use and baseline riluzole × visit interaction, baseline edaravone use and baseline

edaravone × visit interaction, and baseline sodium phenylbutyrate/taurursodiol use and baseline sodium phenylbutyrate/taurursodiol × visit interaction. Fixed terms other than treatment group and visit will be centered by subtracting the sample mean of a given covariate for a given analysis set. The model will assume unstructured covariance among participant-specific repeated measures. In analysis sets in which placebo participants are drawn from more than three regimens, the model will include random regimen-specific intercepts. In analysis samples with three or fewer regimens, the model will include fixed effects for regimen.

The following equations describe the model with regimen random effects:

$$\begin{gathered} Y_{ij} = a_{k(i)} + \gamma_1 t_i + \gamma_{2,j} v_j + \boldsymbol{\gamma}'_{\mathbf{3}} \boldsymbol{z_i} + \gamma_{4,j}\, t_i v_j + \boldsymbol{\gamma}'_{\mathbf{5},\boldsymbol{j}} \boldsymbol{z}_i\, v_j + \epsilon_{ij} \\ a_k \sim N(0, \sigma_r^2), \boldsymbol{\epsilon_{i\cdot}} \sim N(\mathbf{0}, \boldsymbol{R}), \mathrm{Cov}\big(b_{k(i)}, \epsilon_{ij}\big) = 0 \end{gathered} \qquad \text{(eqn. 1)}$$

where Y_{ij} is a given efficacy endpoint measured for participant i at visit j, $a_{k(i)}$ is a random intercept for regimen k to which participant i was assigned, v_j is an indicator variable for visit j, $\boldsymbol{z}_i$ is the vector of covariates (centered time since onset, centered delta-FRS, centered baseline log-transformed serum NfL level, centered baseline riluzole use, centered baseline edaravone use, and centered baseline sodium phenylbutyrate/taurursodiol use) for participant i, t_i is an indicator variable for treatment t to which participant i was assigned, γ_1, $\gamma_{2,j}$, $\boldsymbol{\gamma_3}$, $\gamma_{4,j}$, and $\boldsymbol{\gamma_{5,j}}$ are estimated parameters and vectors of parameters for the fixed effects, and ϵ_{ij} is the residual for participant i at visit j. The regimen-specific random effects are normally distributed with mean 0 and variance σ^2_r. The vector of residuals for a given participant are normally distributed with mean **0** and an unstructured covariance matrix **R**. The regimen-specific random effect for a given participant and residuals for that participant are uncorrelated.

The following SAS code specifies the model:

```
proc mixed data=xxx method=reml;
  class regimen id trtrnd visit;
  model Value = trtrnd|visit
                sx2bl|visit dFRS|visit nfl|visit rlz|visit edv|visit
                amx|visit / solution cl;
  random intercept / subject=regimen type=vc;
  repeated visit / subject=id type=un;
```

where `id` is a participant study identifier, `trtrnd` is the randomly assigned treatment group, `visit` is the visit identifier, `Value` is value of the efficacy endpoint being tested for a given participant at a given visit, `sx2bl` is months since ALS symptom onset, `dFRS` is pre-baseline slope, `nfl` is baseline log-transformed serum NfL level, `rlz` is an indicator of riluzole use at baseline, `edv` is an indicator of edaravone use at baseline, and `amx` is an indicator of sodium phenylbutyrate/taurursodiol use at baseline.

The primary estimate will be the treatment-dependent difference in change from baseline to the Week 24 Visit. The estimate and its 95% Wald confidence bounds will be obtained by a linear contrast of adjusted means. The following SAS code specifies the linear contrast for a regimen with one active treatment assuming an endpoint measured every 8 weeks and that the sort order for treatment group has the active group last and visits are sorted chronologically:

```
  estimate "3|Act vs Plb|dWk 24" trtrnd*visit 1 0 0 -1  -1 0 0 1 / cl;
```

The estimand estimated by the primary linear contrast of the repeated-measures linear mixed model is the mean difference in 24-week change from baseline of a given continuous efficacy endpoint in the active treatment group relative to the placebo group in the FAS population. The estimand is defined by the following attributes:

- Treatment: active treatment vs. placebo.
- Population: FAS population as defined in Section 6.1.
- Variables: absolute change in endpoint from baseline to the Week 24 Visit.
- Intercurrent event: treatment discontinuation: handled via treatment policy approach, all data will be used including data collected during the placebo-controlled period after treatment discontinuation. Missing data post-treatment, including data missing due to death, will not be imputed, handled via missing at random assumption.
- Population-level summary: difference in conditional means of active treatment relative to placebo.

A significant positive difference in 24-week change from baseline in the direction of greater improvement or less worsening among participants randomized to active treatment would support inference of benefit from active treatment for the efficacy endpoint being tested. Inference from this analysis is supportive of inference from the Bayesian shared-parameter, repeated-measures model for the primary endpoint.

6.5.3 Random-slopes Model

Treatment groups of a given regimen will be compared for mean rate of progression of continuous efficacy and selected safety endpoints (ALSFRS-R total score, ALSFRS-R domain scores, HHD upper extremity, lower extremity, and global average percentages, SVC percent-predicted, serum creatinine, serum NfL, and weight) using a random-slopes linear mixed model in the FAS, ECC, ERO, ECM, and EPP samples. The model will include fixed terms for month since the Baseline Visit, treatment group, treatment group × month interaction, time since ALS symptom onset and time since ALS symptom onset × month interaction, delta-FRS and delta-FRS × month interaction, baseline riluzole use and baseline riluzole use × month interaction, baseline edaravone use and baseline edaravone use × month interaction, baseline sodium phenylbutyrate/taurursodiol use and baseline sodium phenylbutyrate/taurursodiol × month interaction, and baseline log-transformed serum NfL level and baseline log-transformed serum NfL level × month interaction. Study months will be calculated as the difference in days from the Baseline Visit to the date of assessment of a given endpoint multiplied by 12 / 365.25. Fixed terms other than treatment group and month will be centered by subtracting the sample mean of a given covariate for a given analysis set. The model will include random participant-specific intercepts and slopes with unstructured covariance. In analysis samples in which placebo participants are drawn from more than three regimens, the model will include random regimen-specific intercepts and slopes with unstructured covariance. In analysis samples with three or fewer regimens, the model will include fixed effects for regimen and regimen x month interaction.

The following equations describe the model with regimen random effects:

$$\begin{gathered} Y_{ij} = \gamma_1 + a^0_{k(i)} + b^0_i + \gamma_2 t_i + \boldsymbol{\gamma}'_3 \boldsymbol{z_i} \\ + \left(\gamma_4 + a^1_{k(i)} + b^1_i + \gamma_5 t_i + \boldsymbol{\gamma}'_6 \boldsymbol{z_i}\right) m_{ij} + \epsilon_{ij} \\ \{a^0_k, a^1_k\} \sim N(\mathbf{0}, \boldsymbol{\Sigma_r}), \{b^0_k, b^1_k\} \sim N\left(\mathbf{0}, \boldsymbol{\Sigma_p}\right), \epsilon_{ij} \sim N(0, \sigma^2_\epsilon) \\ \mathrm{Cov}(\boldsymbol{a}_k, \boldsymbol{b}_k) = \mathbf{0}, \mathrm{Cov}(\boldsymbol{a}_k, \boldsymbol{\epsilon}_{i\cdot}) = \mathbf{0}, \text{and } \mathrm{Cov}(\boldsymbol{b}_k, \boldsymbol{\epsilon}_{i\cdot}) = \mathbf{0} \end{gathered} \qquad \text{(eqn. 2)}$$

where Y_{ij} is a given efficacy endpoint measured for participant i at visit j, $a^0{}_{k(i)}$ and $a^1{}_{k(i)}$ are random intercept and slope for regimen k to which participant i was assigned, $b^0{}_i$ and $b^1{}_i$ are random intercept and slope for participant i, $\boldsymbol{z_i}$ is the vector of covariates (centered time since onset, centered delta-FRS, centered baseline riluzole use, centered baseline edaravone use, centered for sodium phenylbutyrate/taurursodiol use, and baseline log-transformed serum NfL level) for participant i, m_{ij} is the time from baseline to observation j for participant i in months calculated as days x 12 / 365.25, t_i is an indicator variable for treatment t to which participant i was assigned, γ_1, γ_2, $\boldsymbol{\gamma_3}$, γ_4, γ_5, and $\boldsymbol{\gamma_6}$ are estimated parameters and vectors of parameters for the fixed effects, and ϵ_{ij} is the residual for observation j for participant i. The regimen-specific random effects are normally distributed with mean **0** and unstructured covariance matrix $\boldsymbol{\Sigma_r}$. The participant-specific random effects are normally distributed with mean **0** and unstructured covariance matrix $\boldsymbol{\Sigma_p}$. The residuals for a given participant are normally distributed with mean 0 and variance $\sigma^2{}_\epsilon$. The regimen-specific random effects, participant-specific random effects, and residuals are uncorrelated.

The following SAS code specifies the model:

```
proc mixed data=xxx method=reml;
  class regimen id trtrnd;
  model Value = month trtrnd*month
                sx2bl|month dFRS|month nfl|month rlz|month edv|month
                amx|month / solution;
  random intercept month / subject=regimen type=un;
  random intercept month / subject=id type=un;
```

where `month` is time in months from the Baseline Visit (assuming 12 months in an average of 365.25 days per year) and other fields are the same as identified above in Section 6.5.2.

The primary estimate will be the treatment-dependent difference in slopes. The estimate and its 95% Wald confidence bounds will be obtained by a linear contrast of adjusted means. The following SAS code specifies the linear contrast for a regimen with one active treatment assuming that the sort order for treatment group has the active group last:

```
  estimate "3|Act vs Plb|Slope (/mn)" month 0 trtrnd*month -1 1 / cl;
```

The estimand estimated by the primary linear contrast of the repeated-measures linear mixed model is the mean difference in 24-week change from baseline of a given continuous efficacy endpoint in the active treatment group relative to the placebo group in the FAS population. The estimand is defined by the following attributes:

- Treatment: active treatment vs. placebo.
- Population: FAS population as defined in Section 6.1.
- Variables: absolute change in endpoint from baseline to the Week 24 Visit.

- Intercurrent event: treatment discontinuation: handled via treatment policy approach, all data will be used including data collected during the placebo-controlled period after treatment discontinuation. Missing data post-treatment, including data missing due to death, will not be imputed, handled via missing at random assumption.
- Population-level summary: difference in conditional means of active treatment relative to placebo.

A significant positive difference in slopes in the direction of greater improvement or less worsening among participants randomized to active treatment would support inference of benefit from active treatment for the efficacy endpoint being tested. Inference from this analysis is supportive of inference from the Bayesian shared-parameter, repeated-measures model for the primary endpoint.

6.5.4 Survival

The survival endpoints will be analyzed in the FAS, ECC, ERO, ECM, EPP, STF, STN, and SRO analysis sets. The proportion of participants who reach each survival endpoint and the time from baseline to that endpoint will be summarized by treatment group in all analysis samples. If more than 10% of a given sample reaches a survival endpoint, then the estimated time to a 10% event rate will be estimated by Kaplan-Meier product-limit estimates with complimentary log-log confidence bounds by treatment group. Time to each survival endpoint will be compared between treatment groups by log-rank test. If at least one occurrence of a given survival endpoint is observed in each treatment group, then the treatment-dependent hazard ratio with profile likelihood confidence bounds will be estimated by unadjusted Cox proportional hazards regression and by Cox proportional hazards regression adjusting for age at baseline, time since ALS symptom onset, delta-FRS, baseline log-transformed serum NfL level, use of riluzole at baseline, use of edaravone at baseline, and use of sodium phenylbutyrate/taurursodiol at baseline with a random gamma-distributed regimen-specific frailty term. If convergence is not obtained or the analysis sample includes 3 or fewer regimens, then the frailty term will be dropped.

6.5.5 CAFS

To account for potentially informative loss to follow-up due to death, treatment groups of a given regimen will be compared by the Combined Assessment of Function and Survival (CAFS, Berry et al. 2013) in the FAS, ECC, ERO, ECM, and EPP samples. CAFS is a joint-rank test (Finkelstein and Schoenfeld 1999) of a composite endpoint combining function and survival. The test consists of calculating a rank-sum score for each individual relative to pair-wise comparisons with all other participants. Participants are ranked according to time to the survival endpoint when that is observed for both members of a pair or when one is censored after the observed event time for the other. Pairs that cannot be ranked by time to the survival endpoint are ranked by absolute change from baseline in the functional endpoint at the maximum follow-up time at which both participants have an observation. Pair-wise rankings are scored -1 for the member of the pair with shorter time to the survival endpoint or greater decline in function and 1 for the other member or 0 for both members when time to the survival endpoint is equal or not evaluable and change in function is equal, e.g., when one participant is lost to follow-up after baseline. Pair-wise ranking scores are summed by participant to calculate a rank-sum. Inference of treatment-dependent difference in the hierarchical composite of death and disease progression is based on a Wilcoxon rank-sum test of the participant-specific rank-sum scores or on a regression of the rank-sum scores adjusting for covariates.

All combinations of the following elements will be tested:

1. Survival endpoint: (a) death or death-equivalent, and (b) death alone.

2. Function endpoint: (a) ALSFRS-R total score, (b) HHD upper extremity percentage, (c) SVC percent-predicted, and (d) HHD lower extremity percentage.

3. Analysis: (a) Wilcoxon rank-sum test by t-distribution approximation, and (b) adjusted mixed model analysis controlling for time since ALS symptom onset, delta-FRS, baseline log-transformed serum NfL level, use of riluzole at baseline, use of edaravone at baseline, and use of sodium phenylbutyrate/taurursodiol at baseline and including a random effect of regimen in all analyses that include at least three regimens or a fixed effect of regimen otherwise.

6.5.6 HHD0 and HHD0[2]

The time to first and second loss of measurable strength among muscles assessed by HHD and grip strength will be compared between treatments by interval-censored nonparametric maximum likelihood methods and interval-censored Cox proportional hazards regression in the FAS, ECC, ERO, ECM, and EPP samples. The model will adjust for time since ALS symptom onset, delta-FRS, baseline log-transformed serum NfL level, use of riluzole at baseline, use of edaravone at baseline, and use of sodium phenylbutyrate/taurursodiol at baseline.

The first HHD0 and HHD0[2] analyses will consider all 11 muscles and grip strength with non-zero strength at baseline (up to 24 bilateral strength assessments in total). A second pair of HHD0 and HHD0[2] analyses will restrict to the four bilateral muscle groups or grip strength that reach zero most frequently in a specific analysis sample (up to 8 bilateral strength assessments in total).

6.5.7 Placebo Multiple Imputation

To account for possible treatment-dependent loss to follow-up whether due to death, intolerance, or other causes, the effect of treatment on ALSFSR-R total score, HHD upper extremity percentage, SVC percent-predicted, and HHD lower extremity percentage will be estimated in the repeated-measures and random-slopes models using placebo multiple imputation for missing data (Ayele et al. 2014) in the FAS and ERO samples. Fifty imputed datasets will be generated for a given endpoint. Missing values for each visit will be imputed from those predicted by linear regression from earlier visits using data only from placebo participants, adjusting for time since symptom onset, delta-FRS, baseline log-transformed serum NfL level, baseline riluzole use, baseline edaravone use, and baseline sodium phenylbutyrate/taurursodiol use.

The following SAS code specifies the imputation for an endpoint measured every 8 weeks:

```
proc mi data=work.outcomes seed=xxx nimpute=50 minmaxiter=1000
        minimum=. . . . . . . x x x x maximum=. . . . . . . y y y y ;
  class trtrnd rlz edv amx;
  fcs reg(Wk08 = sx2bl dFRS nfl rlz edv amx Wk00);
  fcs reg(Wk16 = sx2bl dFRS nfl rlz edv amx Wk00 Wk08);
  fcs reg(Wk24 = sx2bl dFRS nfl rlz edv amx Wk00 Wk08 Wk16);
  mnar model( Wk08 Wk16 Wk24 / modelobs=(trtrnd="0"));
  var sx2bl dFRS nfl rlz edv amx Wk00 Wk08 Wk16 Wk24;
run;
```

where `Wk00`, `Wk08`, `Wk16`, and `Wk24` are the values of a given efficacy endpoint at the Baseline, Week 8, Week 16, and Week 24 Visits, respectively, `trtrnd` has a value of zero (0) for participants randomized to placebo, and `x` and `y` take appropriate values to specify the range of a given outcome measure (i.e., 0 and 48 for ALSFRS-R total score; 0 and . [missing value indicator] for HHD upper and lower extremity percentages and SVC).

6.5.8 Additional Sensitivity Analyses of Primary and Key Secondary Outcomes

To evaluate the influence of extreme values on treatment estimates from the random-slopes model, a robust linear mixed model that down-weights extreme values (Koller 2013) will be used as a sensitivity analysis of primary and key secondary outcomes using the FAS analysis set. The same fixed and random effects as specified for the primary random-slopes model in Section 6.5.3 will be used. A smoothed Huber ρ-function will be used with a tuning parameter that yields 95% asymptotic efficiency for normally distributed data.

As an alternative to CAFS for controlling the influence of potentially information loss to follow-up due to death or other causes, sensitivity of estimated treatment effects to potentially informative missingness will be analyzed using a composite endpoint of time to death and 24-week change in ALSFRS-R total score using the FAS analysis set with imputation across a range of weights spanning relative benefit or harm from the active treatment by exponential tilting (Wang et al. 2017).

6.5.9 Subgroup Analyses

Differences in treatment efficacy will be estimated in the following subgroups by including subgroup, subgroup × time, and subgroup × treatment × time terms to random-slope models for primary and secondary efficacy endpoints using the FAS analysis set .

- Age (less than 65 years vs. 65 years or older),
- Sex (female vs. male),
- Race (white vs. any minority race with greater than 5% prevalence in the sample),
- Ethnicity (Hispanic or Latino vs. non-Hispanic or Latino),
- Baseline use of riluzole (yes vs. no),
- Baseline use of edaravone (yes vs. no),
- Baseline use of sodium phenylbutyrate/taurursodiol (yes vs. no),
- BMI (less than 25 kg/m^2, 25 to less than 30 kg/m^2, 30 kg/m^2 or more),
- Chronic kidney disease (CKD) stage (stage 1 or better [eGFR 90 mL/min/1.73m^2 or more], stage 2 [eGFR 60 to 89 mL/min/1.73m^2], stage 3 or worse [eGFR less than 60 mL/min/1.73m^2]),
- delta-FRS (fast vs. slow progression with median split),
- Time since onset of weakness (less than 18 months vs. 18 months or longer),
- El Escorial and time since onset of weakness (El Escorial definite and less than 18 months vs. not both),

- Baseline symptom severity (all ALSFRS-R questions scored 2 or greater vs. any question scored 0 or 1),
- Early disease state (all ALSFRS-R questions scored 2 or greater, SVC 80%-predicted or greater, and time since symptom onset less than 24 months vs. not meeting all three criteria),
- Site of symptom onset (bulbar vs. non-bulbar),
- Baseline plasma NfL concentration (high vs. low by median split), and
- Site (individual sites with at least 5 participants per treatment group and all participants from sites with fewer than 5 participants per treatment group pooled).

For each subgroup, unknown, not reported, and missing will be considered as one group. All individuals not included in a specified subgroup will be combined into a mixed, "other" group. The "other" group will be included in analyses if its prevalence is greater than 5%; otherwise, the "other" group will be excluded.

For each subgroup, the following estimates will be obtained:

- the treatment-group specific estimate within each subgroup level,
- the treatment difference within each subgroup level,
- the difference of differences between each pair of subgroup levels, and
- for subgroups with more than two levels, the F-test for the subgroup × treatment × time term will be estimated.

6.5.10 Comparison of Controls across Regimens

The magnitude of differences in regimen-specific rates of progression for primary and secondary efficacy endpoints will be compared by random-slopes models for functional endpoints and by Kaplan-Meier product-limit estimates, log-rank test, and Cox proportional hazards regression for survival using the placebo participants in the FAS analysis set . The random-slopes models will include fixed effects of regimen and regimen x time interaction, adjusting for time since onset, delta-FRS, baseline log-transformed serum NfL level, baseline riluzole use, baseline edaravone use, and baseline sodium phenylbutyrate/taurursodiol and their interactions with time. Fixed terms other than regimen and month will be centered by subtracting the sample mean of a given covariate. The models will include participant-specific intercepts and slopes with unstructured covariance. Adjusted regimen-specific slope estimates, difference of the focal regimen control group vs. each other regimen and vs. the average of all other regimens, and an overall F-test of regimen-specific differences in rates of disease progression will be estimated. Regimen-specific slopes will be plotted vs. calendar year of median randomization. Cox models will include main effects for regimen, time since onset, delta-FRS, baseline log-transformed serum NfL level, baseline riluzole use, baseline edaravone use and baseline sodium phenylbutyrate/taurursodiol use. Adjusted regimen-specific hazard ratios of the focal regimen control group vs. each other regimen and vs. the average of all other regimens, and an overall F-test of regimen-specific differences in hazard of mortality will be estimated.

6.6 Safety Analyses

6.6.1 Treatment-emergent Adverse Events

TEAEs are defined as those adverse events with onset dates in the interval from treatment initiation to either the Final Safety Visit if completed, the date of death if the participant dies, the date of last study contact if the participant early terminates or is lost to follow-up, or the date of first dose of study drug during participation in the ATE, if so exposed. Adverse events with onset on the day of placebo-controlled treatment initiation and adverse events with incompletely specified onset date where the ambiguous date spans the day of double-blind treatment initiation will be assumed to be treatment emergent except for those known to precede first exposure to study drug.

The incidence of TEAEs will be summarized by the number of events of a given classification experienced by participants in each treatment group and by the number and proportion of participants experiencing such an event in each treatment group in all safety samples. The number of events and proportion of participants experiencing TEAEs will be summarized without regard to MedDRA term for any TEAE, any serious TEAE, any severe TEAE, any fatal TEAE, any related TEAE, and any TEAE leading to discontinuation of study drug. TEAEs will be summarized by MedDRA system organ class, high level term, and preferred term for all TEAEs. TEAEs, serious TEAEs, and non-serious TEAEs observed in at least 5% of participants in a given treatment group will be summarized by MedDRA system organ class and preferred term and by MedDRA preferred term alone. TEAEs will be summarized by MedDRA system organ class and preferred term for serious TEAEs, severe TEAEs, fatal TEAEs, related TEAEs, and TEAEs leading to discontinuation of study drug. TEAEs and serious TEAEs will be stratified by the following subgroups: age, sex, race, ethnicity, baseline riluzole use, baseline edaravone use, baseline sodium phenylbutyrate/taurursodiol use, weight, BMI, and CKD stage. Subgroup classifications will be the same as described in Section 6.5.9 Subgroup Analyses except that the "other" group will be retained regardless of prevalence.

For overall summaries of TEAE not classified by MedDRA term and for summaries of TEAEs, serious TEAEs, and non-serious TEAEs observed in at least 5% of participants in a given treatment group, treatment-specific TEAE incidence rates in units of number per 100 participant years and treatment-dependent differences will be estimated by Poisson regression with log link and offsets equal to the log of weeks from initiation to discontinuation of placebo-controlled treatment. Comparison-wise 95% confidence intervals will be calculated with variance estimates obtained by the delta method.

The incidence of TEAEs will be summarized without regard to MedDRA term in all safety samples for the following characteristics: (a) seriousness, (b) severity, (c) relationship to study drug, (d) action taken with study drug, (e) use of a concomitant medication, and (f) outcome. For each level of a given TEAE characteristic, summaries will include the number of events of a given classification and the number and proportion of participants for which that level of a characteristic was the worst they experienced. Any unknown characteristic of a TEAE will not be classified as worst. The level for participants who experienced no TEAE will be coded as the zero-level of each characteristic.

6.6.2 Safety Labs

The absolute level and the absolute change from baseline for each safety laboratory assay will be summarized as means, standard deviations, medians, and ranges at each visit by treatment group in all safety samples. The proportion of participants with abnormal levels classified to a toxicity grade based on quantitative grading using National Cancer Institute (NCI) Common Terminology Criteria for Adverse Events (CTCAE) version 5.0, the proportion with abnormal and clinically significant safety lab levels, and the proportion with possible DILI by each definition will be summarized by treatment group by visit and for any post-baseline visit in all safety samples. The proportion of participants with abnormal levels by CTCAE grade, the proportion with lab levels that are below the lower limit of normal, within normal limits, or above the upper limit of normal will be presented as shift tables vs. the status of each participant at baseline for each visit and for any post-baseline visit by treatment group in all safety samples.

6.6.3 ECG Results

The absolute level and the absolute change from baseline for each ECG parameter will be summarized as means, standard deviations, medians, and ranges at each visit by treatment group in all safety samples. The proportion of participants with abnormal and clinically significant ECG findings or parameter values of special concern will be summarized by finding for each treatment group by visit and at any post-baseline visit in all safety samples. The parameter values of special concern are the following: QTcF > 480 ms, QTcF > 500 ms, increase from baseline in QTcF > 60 ms, increase from baseline in PR > 25% to a value above 200 ms, increase from baseline in HR > 25% to a value below 50 bpm, increase from baseline in HR > 25% to a value above 100 bpm, and increase from baseline in QRS > 25% to a value above 110 ms.

6.6.4 Vital Signs and Weight

The absolute level and the absolute change from baseline for vital signs and weight will be summarized as means, standard deviations, medians, and ranges at each visit by treatment group in all safety samples. Changes in weight will also be analyzed as an efficacy endpoint by repeated-measures and random-slopes mixed models in the FAS, ECC, ERO, ECM, and EPP samples.

6.6.5 Suicidality

The proportion of participants who report any post-baseline suicidal ideation or any post-baseline suicidal behavior on the C-SSRS will be summarized by treatment group in all safety samples. Suicidal behaviors will include: actual attempts, non-suicidal self-injurious behavior, interrupted attempts, aborted attempts, preparatory acts or behaviors, and completed suicide. The most severe ideation, maximal frequency, maximal duration, minimal controllability, minimal deterrents, maximal reasons for ideation, maximal actual lethality or medical damage, and maximal potential lethality will be summarized by treatment group in all safety samples.

6.7 Other Analyses

6.7.1 Participant Disposition

All participants consented to the Master Protocol between the time of the first and last consent of a participant assigned to a regimen included in the FAS analysis set will be summarized as a single set for the following events: consented to the Master Protocol, failed screening for the Master Protocol, other reasons not assigned to a regimen (including timing out of the screening

window, death, withdrawal of consent, early termination, and administrative termination), and assigned to a regimen. The number of participants ever assigned and last assigned to the focal regimen, to a non-focal contributing regimen, and to a non-contributing regimen will be separately indicated. Reasons for Master Protocol screen failure will be summarized.

All participants in the above sample assigned to the focal regimen or a non-focal contributing regimen will be summarized as two sets (last assigned to the focal regimen vs. last assigned to a non-focal contributing regimen) for the following events: consented to a regimen, failed screening for a regimen, other reasons not randomized within a regimen (including timing out of the screening window, death, withdrawal of consent, early termination, and administrative termination), and randomized within a regimen. If a given individual is screened multiple times prior to randomization within a regimen, then the final screening experience of that individual will be summarized. Reasons for screen failure will be summarized separately for all participants screened for the focal regimen whether that was their final screening experience or not.

All participants in each analysis set will be summarized as two sets (randomization to active study drug vs. randomization to placebo) for the following events: initiated regimen-specific study drug, prematurely terminated study participation due to death, withdrawal of consent, early termination, loss to follow-up, or administrative termination, completed 24-week follow up, and completed a safety follow-up visit vs. continued into the ATE. Reasons for withdrawal from the study will be presented.

6.7.2 Study Drug Compliance and Tolerance

The number of days of exposure to study drug will be summarized by treatment group in the ERO and SRO analysis sets. The proportion of participants on study drug at each clinic visit will be summarized by treatment group. Study drug exposure will be calculated In two ways:

- as the number of days from dose initiation to the final safety assessment during the placebo-controlled period, inclusive, and
- as the number of days from dose initiation to drug withdrawal, inclusive, less any interval during which use of study drug was interrupted (individual missed doses will not be subtracted unless noted in the dosage management log).

The proportion of participants judged tolerant of study drug, both those who are free of intolerance due to an AE and those free of intolerance for any reason, will be summarized by treatment group.

6.7.3 Concomitant Medication Use

Concomitant medications taken during the study period will be coded using the World Health Organization (WHO) Drug Dictionary Enhanced and Default Anatomical, Therapeutic, and Chemical (ATC) class. The percentage of participants in all safety samples taking each WHO drug classified medication and each ATC class of medications will be summarized by treatment group for medications taken at baseline and those initiated after first dose of study drug.

6.7.4 Medical History

Medical histories will be summarized by MedDRA system organ class, high level term, and preferred term by treatment group for all medical conditions recorded and for medical conditions identified as ongoing at baseline in the FAS, ERO, STF, and SRO analysis sets.

6.7.5 Blindedness

The proportions of participants and site investigators who report on the Exit Questionnaire a guess of active vs. placebo treatment assignment, each level of surety of that guess, and each of five pre-specified reasons for making a treatment assignment will be summarized by treatment group in the FAS, ERO, and EPP analysis sets. Treatment-dependent differences in the proportion guessing active treatment assignment will be tested among all respondents and among those stating they are at least somewhat sure of their guess by Fisher's exact test, the difference in proportion guessing active treatment assignment will be estimated with confidence bounds, and the distribution of surety of guesses will be compared by exact Cochran–Armitage trend test.

6.7.6 Protocol Deviations

The number of major and minor protocol deviations will be summarized by type of deviation and treatment group in all analysis sets. Listings of all protocol deviations will be produced.

7. Validation

7.1 Primary Efficacy Analysis

The primary efficacy analysis will be validated according to a validation plan administered by Berry Consultants.

7.2 Secondary, Exploratory, and Safety Analyses

Key secondary analyses of the primary efficacy endpoint and primary analyses of secondary efficacy endpoints and summaries and analyses of serious TEAEs will be replicated from Study Data Tabulation Model (SDTM) source data by two analysts or statisticians working independently. Exact matches are required to validate results for the following integer results: treatment- and visit-specific counts of efficacy endpoints, counts of events, number of participants affected, and number of participants at risk for safety endpoints. Relative matches within 0.1% of the common mean are required to validate results for the following floating point results: estimates, standard errors, confidence bounds, and p-values.

All other analysis code and results will be reviewed by a statistician other than the initial analyst.

8. References

Ayele BT, Lipkovich I, Molenberghs G, Mallinckrodt CH. A multiple-imputation-based approach to sensitivity analyses and effectiveness assessments in longitudinal clinical trials. J Biopharm Stat. 2014;24(2):211-28.

Balendra R, Jones A, Jivraj N, Knights C, Ellis CM, Burman R, Turner MR, Leigh PN, Shaw CE, Al-Chalabi A. Estimating clinical stage of amyotrophic lateral sclerosis from the ALS Functional Rating Scale. Amyotroph Lateral Scler Frontotemporal Degener. 2014 Jun;15(3-4):279-84.

Berry JD, Miller R, Moore DH, Cudkowicz ME, van den Berg LH, Kerr DA, Dong Y, Ingersoll EW, Archibald D. The Combined Assessment of Function and Survival (CAFS): a new endpoint for ALS clinical trials. Amyotroph Lateral Scler Frontotemporal Degener. 2013 Apr;14(3):162-8.

Cedarbaum JM, Stambler N, Malta E, Fuller C, Hilt D, Thurmond B, Nakanishi A. The ALSFRS-R: a revised ALS functional rating scale that incorporates assessments of respiratory function. BDNF ALS Study Group (Phase III). J Neurol Sci. 1999 Oct 31;169(1-2):13-21.

Finkelstein DM, Schoenfeld DA. Combining mortality and longitudinal measures in clinical trials. Stat Med. 1999 Jun 15;18(11):1341-54.

Koller M. Robust Estimation of Linear Mixed Models. Diss., ETH Zurich, Nr. 20997, 2013, URL https://www.research-collection.ethz.ch/bitstream/handle/20.500.11850/65478/eth-6670-02.pdf (accessed 17 Jun 2020).

Quanjer PH, Stanojevic S, Cole TJ, Baur X, Hall GL, Culver BH, Enright PL, Hankinson JL, Ip MS, Zheng J, Stocks J; ERS Global Lung Function Initiative. Multi-ethnic reference values for spirometry for the 3-95-yr age range: the global lung function 2012 equations. Eur Respir J. 2012 Dec;40(6):1324-43. SAS Macro at https://www.ers-education.org/guidelines/global-lung-function-initiative/spirometry-tools/sas-macro.aspx (accessed 30 Oct 2019).

Roche JC, Rojas-Garcia R, Scott KM, Scotton W, Ellis CE, Burman R, Wijesekera L, Turner MR, Leigh PN, Shaw CE, Al-Chalabi A. A proposed staging system for amyotrophic lateral sclerosis. Brain. 2012 Mar;135(Pt 3):847-52.

Wang C, Scharfstein DO, Colantuoni E, Girard TD, Yan Y. Inference in randomized trials with death and missingness. Biometrics. 2017 Jun;73(2):431-440.

9. Appendix I

ALS Master Protocol
Recommended Statistical Analysis, Design and Simulation Report

February 6, 2023

Table of Contents

Summary of Changes 32
1.0 Introduction **33**
2.0 Primary Efficacy Analysis **34**
2.1 Primary Analysis Population and Shared Control 34
2.2 Bayesian Shared Parameter Model of Function and Mortality 35
2.2.1 Treatment Effect ALSFRS-R and Mortality 37
2.2.2 Random Intercept and Slope Effects for ALSFRS-R 37
2.2.3 Regimen-Specific Random Slope Effects ALSFRS-R 38
2.2.5 Baseline Covariate Effects ALSFRS-R 39
2.2.6 Residual Errors ALSFRS-R 40
2.2.7 Baseline Mortality Hazard Rate 40
2.3 Posterior Summaries 40
3.0 Success Thresholds, Interim Analyses and Trial Adaptations **40**
3.1 Regimen Success 41
3.2 Regimen Futility 41
4.0 Goodness of Fit Diagnostics and Sensitivity Analyses of Primary Efficacy Analysis **41**
4.1 Goodness-of-fit Diagnostics 41
4.2 Key Supportive Joint Rank Analysis of ALSFRS-R and Mortality 42
4.3 Sensitivity of Analysis Population 42
4.4 Sensitivity Analysis for Handling Missing Data 43
4.5 Shared Parameter Model Sensitivity Analyses 43
4.6 Sensitivity of Survival Endpoint 44
5.0 Clinical Trial Simulations **45**
5.1 Virtual Patient ALSFRS-R Simulation 45
5.2 Base Simulation Scenarios and Sensitivity Scenarios 46
6.0 Example Trials and Operating Characteristics **48**
6.1 Operating Characteristics Base Scenarios 48
6.2 Operating Characteristics and Example Trials Integration of Mortality and Function 50
6.3 Operating Characteristics and Example Trials Differences Across Regimens 56
6.4 Operating Characteristics Additional Sensitivity Scenarios 61
7.0 References **62**

Summary of Changes

Changes since document dated May 14, 2021.

Section 1.0: Updated general descriptions of trial design, primary analysis, planned sample size, and default early stopping conditions at interim analyses.

Section 2.1: Updated the analysis sample definition to exclude participants determined to not meet ALS diagnostic criteria. Clarified the definition of the survival endpoint. Defined "ongoing participants" and specified that data from ongoing participants would not be used in the primary analysis.
Section 2.2: Clarified reference to the survival endpoint.
Section 2.2.5: Added baseline log-transformed serum NfL level and baseline sodium phenylbutyrate/taurursodiol use as additional covariates. Specified that all covariates would be standardized by subtracting their means. Specified that all missing baseline covariates would be imputed by their mean. Specified that means would be calculated after transformation for any transformed baseline covariates.
Section 3.1: Removed reference to the first three regimens specifically.
Section 4.1: Added a table summarizing the number and percent of completed or expected ALSFRS-R assessments that do not contribute to the functional component of the primary analysis.
Section 4.5: Added details regarding the piecewise-linear sensitivity analysis.
Section 4.6: Added a sensitivity analysis using death alone as the survival endpoint.

5.0 Introduction

The ALS Master Protocol is a perpetual, multicenter, multi-regimen, randomized, placebo-controlled, adaptive platform trial. The primary objective of the trial is to evaluate the efficacy of multiple investigational products, as compared to placebo, on ALS disease progression. The master protocol will include multiple regimens where a regimen is defined as both an active treatment and the corresponding randomized placebo. Participants in the master protocol will be randomized in two stages; first to one of the actively enrolling regimens; and then 3:1 to active treatment or placebo within the regimen.

This document describes the master protocol recommended primary endpoint, primary analysis, and interim analyses. This will be the default statistical design and primary analysis for all regimens investigated in this master protocol. The recommended design is intended to support inference of substantial evidence of effectiveness when the data so warrant.

Each regimen will have a regimen-specific appendix (RSA). Deviations from the master protocol recommended statistical design are permitted. The exact statistical design along with complete operating characteristics for each regimen will be specified in the RSA.

The master protocol recommended primary efficacy endpoint is change from baseline through week 24 in disease severity as measured by the ALS Functional Rating Scale-Revised (ALSFRS-R) total score and survival. The primary analysis is a Bayesian shared parameter analysis of ALSFRS-R and survival. The primary analysis compares each regimen-specific active treatment to a shared control group.

The sample size enrolled in each regimen will be determined based on the specifics of the intervention, anticipated effect size, and number of treatment arms evaluated within that regimen. There are frequent interim analyses in order to potentially stop a regimen early for success or for futility. The default design for a regimen will include stopping early only for futility. Interim analyses will be conducted at the same time for all regimens within the master protocol, every 12 weeks of trial time. A regimen is eligible for these interim analyses once a minimum specified amount of data has been observed for that regimen.

2.0 Primary Efficacy Analysis

The following describes the Bayesian shared parameter primary analysis method of function and survival for each regimen, the primary analysis population, prior distributions for all model parameters, and specific posterior summaries of interest that will be used in the design. We denote the regimen being analyzed as the "analysis regimen" throughout.

2.1 Primary Analysis Population and Shared Control

The primary analysis population for each analysis regimen is guided by the intent to treat (ITT) principle and includes all participants randomized to the active treatment arm within the analysis regimen, all participants concurrently randomized to the control arm within the analysis regimen, and control participants from other regimens from a shared control, excluding any participant determined to not meet ALS diagnostic criteria.

The primary analysis dataset uses longitudinal data on disease progression (ALSFRS-R measurements at 0, 4, 8, 12, 16, 20 and 24 weeks, at early termination visits, and assessments during double-blind follow-up that substitute for missed scheduled visits) for those who have not been censored due to reaching the survival endpoint (a composite of death or death-equivalent) as well as occurrence of the survival endpoint during planned double-blind follow-up on participants within the primary analysis population. Follow-up for primary analysis of survival among participants who do not die or reach a death equivalent prior to the Week 24 Visit will be censored at the Week 24 Visit date, if completed, or at the earlier of the end of the Week 24 Visit window or date last known alive if a Week 24 visit is not completed. Off-schedule results, e.g., those collected as part of an Early Termination Visit, will be used in place of the closest missing scheduled visit (while preserving visit sequence) for analyses that depend on visit-specific data. Off-schedule results that do not substitute for a missed schedule visit will not be used.

The shared control group will include participants randomized to the control arm across other actively enrolling regimens (concurrent shared controls) and previously completed regimens (non-concurrent shared controls) within the master protocol as specified in the RSA. Concurrent shared controls are defined as participants on a control arm who were randomized within a 6-month window of the start and stop of randomization of the analysis regimen. For the purposes of analysis, 6 months is defined as 180 days. For example, if the analysis regimen enrolls from Month 12 – Month 24 in the platform, the

concurrent shared controls will include all available data from all participants randomized between Month 6 and Month 30 within the platform across all regimens.

For interim analyses, all available data at the time of the analysis will be used including data on participants who have not had the opportunity to complete 24 weeks of follow-up (denoted ongoing). The phrase "opportunity to complete 24 weeks" is meant to describe participants who have at least 24 weeks of time elapse since their baseline visit.

For final primary analyses, data from ongoing participants will not be used, but participants from open regimens who have had the opportunity to complete 24 weeks of follow-up may be used. To minimize risk of potential unblinding of data for regimens outside of the analysis regimen that have not yet had last patient last visit (open regimens) if shared control data from open regimens are used within the primary analysis, no participant-level data will be shared and no summaries of the data will be provided from which individual participants could be identified until all regimens that are included in the shared control for the analysis regimen have been closed (last patient last visit) and locked. For this reason, regimens may choose to use shared control data only from regimens that have been closed (last patient last visit) to avoid delay in release of participant-level data. The shared control population that will be included in the final primary analysis for each regimen will be prespecified in each regimen-specific appendix.

Finally, the master protocol allows for additional and more restrictive inclusion/exclusion criteria within a regimen specific appendix (RSA). However, these additional inclusion/exclusion criteria will be limited and will be allowed based only on restrictions due to safety or mechanism of action. Major additional RSA inclusion/exclusion criteria that can be assessed for all participants entering the platform will be used to subset the shared control to include only shared control participants that meet these major additional RSA inclusion/exclusion criteria. Major additional RSA inclusion/exclusion criteria that will be used to subset the shared control will be limited to those criteria that have the potential to lead to significant differences in the patient population in terms of the rate of disease progression within the primary analysis population and will be prespecified within each RSA. The first three regimens enrolling within the platform did not include any major additional inclusion/exclusion criteria.

2.2 Bayesian Shared Parameter Model of Function and Mortality

The primary analysis for each analysis regimen is a Bayesian shared parameter model of function and survival with the following components:

- **Function**: Bayesian repeated measures model of ALSFRS-R at baseline through 24 weeks for all participants who have not experienced mortality (per definition of the composite survival endpoint). Participants who have experienced mortality will not contribute to the functional component of the analysis. The Bayesian repeated measures model of ALSFRS-R measures the

slowing in the rate of progression for ALSFRS-R in treated participants relative to control for survivors.
- **Mortality**: Exponential proportional hazards model of time to the composite survival endpoint of death or death-equivalent through 24 weeks that measures the mortality hazard ratio in treated participants relative to control.
- **Shared Treatment Effect**: A shared treatment effect parameter (slowing in time of the progression of the disease) is common between the function and mortality components and provides an overall estimate of the treatment effect that is averaged across the two endpoints. This shared parameter corresponds to a slowing in the rate of ALSFRS-R progression as well as a slowing in the time to mortality.

The ALSFRS-R repeated measures component of survivors is based on a linear rate of progression in ALSFRS-R for placebo participants and a proportional slowing in the rate of progression for treated participants at each time point. The model incorporates participant-level random effects in the baseline value of the ALSFRS-R (intercept) and in the rate of progression (slope); covariate effects to account for covariate-explainable differences in rates of progression based on participant-specific baseline covariates; regimen-specific differences in baseline values, rates of progression and measurement error, and a time-trend effect to accommodate non-concurrently randomized controls. Assuming there are N_{func} total participants who have survived, let the ALSFRS-R at timepoint *j* for participant *I* be labelled Y_{ij}, for *j* = the number of weeks at the visit with expected values around 0, 4, 8, 12, 16, 20, and 24 weeks and $i = 1, \ldots, N_{func}$. We note that the value for *j* will be the actual week that the visit took place rounded to the nearest day. The Bayesian repeated measures model for ALSFRS-R is

$$Y_{ij} = (\alpha_{r(i),t(i)} + \gamma_i) - \beta_i * \exp\left(\theta_{t(i)} + \delta X_i + \rho_{d(i)} + \eta_{r(i)}\right) * \left(\frac{j * 12}{52}\right) + \epsilon_{ij};$$
$$\epsilon_{ij} \sim N\left(0, \sigma^2_{r(i),t(i)}\right).$$

The mortality component is an exponential proportional hazards model that assumes a constant baseline hazard rate for all control participants. Given the low expected rate of mortality within this study (5%) and to avoid over-parameterization, we do not include covariate, regimen-specific, or time-trend effects in the mortality component. Assuming there are N_{mort} total participants, the hazard rate of mortality per month for participants $i = 1, \ldots, N_{mort}$ is denoted λ_i and is

$$\lambda_i = \lambda_0 \exp\left(\theta_{t(i)}\right); i = 1, \ldots, N_{mort}.$$

The shared parameter model makes use of the following additional notation:
- Treatment arm: $t(i)$ denotes the treatment arm, *T,* to which participant *i* is randomized where $T = 0{,}1$ corresponds to placebo and active arm, respectively.
- Regimen: $r(i)$ denotes the regimen, *R,* to which participant *i* is randomized where $R = 1, \ldots N_r$ corresponds to the analysis regimen (R=1) and all other N_r, enrolling regimens respectively.

- Time of Randomization: $d(i)$ denotes the time of randomization, d, for participant *i*. The time of randomization is expressed as an ordinal discrete outcome that where $d = 0$ is the current time period for concurrent controls (defined as in Section 2.1 with those participants randomized within 6 months (180 days) from the start or finish of randomization within the analysis regimen) and $d = 1, \ldots, D$ are the number of additional 6-month intervals preceding the start of the current time period.
- Baseline Covariates: X_i is a vector of dimension C of baseline covariates for participant *i* that have been standardized.

Each parameter in the above model and the prior distribution assumed for each parameter is described below in Sections 2.2.1 – 2.2.7. The majority of the prior distributions are chosen to be non-informative (large amount of variability and uncertainty) to allow the posterior distributions to reflect what is observed in the data.

2.2.1 Treatment Effect ALSFRS-R and Mortality

The disease rate ratio (DRR) under treatment T is the parameter $\exp(\theta_T)$. For a placebo patient, the DRR is set equal to 1 ($\theta_0 = 0$). Thus, the DRR parameter for active treatment represents the relative change to the rate of decline of ALSFRS-R and the rate of mortality of treated participant relative to a placebo participant. The estimated DRR can also be interpreted as the average rate of decline in function and mortality, with the weights across function and mortality depending on the proportion of patients with observed mortality. Appropriately, more weight is given to mortality relative to function as the proportion of observed mortality increases. If the DRR is less than 1 then the rate of disease progression on function and mortality for actively treated participants is slower than that for placebo. The DRR value represents the proportional slowing in time (or increase) of the disease. A DRR = 0.75 corresponds to a 25% slowing in the rate of functional progression and time to mortality and a value of 0.25 represents a 75% slowing in the rate of functional progression and time to mortality.

The prior distribution for the DRR for the regimen-specific active treatment, *T=1,* is uniform between 0 and 2; placing equal weight on a DRR<1 and DRR>1:

$$\exp(\theta_1) \sim Unif(0,2).$$

2.2.2 Random Intercept and Slope Effects for ALSFRS-R

Participant-specific random intercept effects, $\gamma_i, i = 1, \ldots, N$, represent the increase or decrease in the participant-specific expected ALSFRS-R at the time of randomization compared to the overall mean for the treatment arm and regimen. Participant-specific

random slope effects, $I, i = 1, \dots, N$, represent the participant-specific rate of decline per month in ALSFRS-R expected under placebo.

The overall mean estimate per treatment arm and regimen is assumed to have a non-informative prior normal distribution with a mean of 38 (mean value observed in historical trials within the PRO-ACT database) and SD of 10:

$$\alpha_{R,T} \sim N(38, 10^2); T = 0,1; R = 1, \dots N_R$$

Participant-level random intercept effects are modelled using a hierarchical normal distribution with a population mean of 0 and population standard deviation, σ_γ, with a uniform hyper-prior:

$$\gamma_i \sim N\left(0, \sigma_\gamma^2\right); i = 1, \dots N;$$
$$\sigma_\gamma \sim Unif(0,10).$$

The uniform hyper-prior on the standard deviation is non-informative with a range from 0 to 10 (twice observed SD of the participant-level intercepts in historical trials within the PRO-ACT database).

Participant-level random slope effects are modelled using a hierarchical normal distribution with overall population mean, μ_β, with a normal hyper-prior; and an arm-specific population standard deviation, $\sigma_{\beta,T}; T = 0,1$, with a uniform hyper-prior:

$$\beta_i \sim N\left(\mu_\beta, \sigma_{\beta,t(i)}^2\right); i = 1, \dots N;$$
$$\mu_\beta \sim N(1,1^2);$$
$$\sigma_{\beta,T} \sim Unif(0,2); T = 0,1.$$

The mean and variance of the participant-level random effects are estimated from the data. This is done by placing hyperprior distributions on the relevant parameters in the random effects distribution and using the observed data to estimate the hyper-parameters. Non-informative distributions are used for these hyper-priors to ensure that the data determine the corresponding hyper-prior estimates and the estimated random effect distributions. In particular, the normal hyper-prior on the mean of the participant-level random slope effects is non-informative with a mean of 1 (mean value observed in historical trials within the PRO-ACT database) with a standard deviation of 1. The uniform hyper-prior on the standard deviation is non-informative with a range from 0 to 2 (over twice observed SD of the participant-level slopes in historical trials within the PRO-ACT database).

2.2.3 Regimen-Specific Random Slope Effects ALSFRS-R

The increase or decrease in the rate of progression for placebo participants for all regimens compared to that of the analysis regimen is the parameter $\exp(\eta_r)$. For the analysis regimen (*r=1*), the effect is assumed to be 1 ($\eta_1 = 0$).

The log of the regimen-specific random effects, η_r, will be modelled using a hierarchical model to allow for dynamic borrowing within the shared control across different regimens:

$$\eta_r \sim N(0, \sigma_\eta^2); r = 2, \ldots N_r;$$
$$1/\sigma_\eta^2 \sim Gamma(1, .05^2).$$

The gamma (shape and rate parameterization) hyper-prior is informed based on estimated between-trial variability in rates of progression for ALSFRS-R from historical trials.

2.2.4 Time-Trend Effects ALSFRS-R

The log of the time-trend effects will be modelled using a simple normal dynamic linear model where the distribution of each time-trend effect is normal with a mean equal to the effect at the previous time period and a standard deviation that has a uniform hyper-prior:

$$\rho_0 = 0;$$
$$\rho_d \sim N(\rho_{d-1}, \sigma_\rho^2); d = 1, \ldots D;$$
$$\sigma_\rho \sim Unif(0,2).$$

2.2.5 Baseline Covariate Effects ALSFRS-R

Covariates included in the primary efficacy analysis are: time since onset, pre-baseline slope of ALSFRS-R, baseline log-transformed serum NfL level, baseline riluzole use, baseline edaravone use, and baseline sodium phenylbutyrate/taurursodiol use as indicated in participants' concomitant medication logs. All covariates will be standardized by subtracting their means using all participants in the regimen-specific analysis data set (regimen-specific treatment and control as well as additional shared control). Missing baseline covariates for a participant will be imputed using the mean value across all participants in the applicable analysis dataset (regimen-specific treatment and control as well as additional shared control for the primary analysis). For any baseline covariate that is transformed for analysis, means will be calculated after transformation. Covariate effects are modelled as $\exp(\delta_c), c = 1, \ldots, C$, accounting for covariate-explainable variability in the rate of ALSFRS-R progression due to participant-specific baseline covariates.

Prior distributions for the log of the covariate effects are independent and identically distributed as:

$$\delta_c \sim N(0, 1^2); c = 1, \ldots C.$$

2.2.6 Residual Errors ALSFRS-R

The residual errors for the individual observations, $\epsilon_{ij}, i = 1, \ldots, N$ and $j = 0, \ldots, 24$ are modelled as independent normal distributions with a mean of 0 and with an treatment arm and regimen-specific standard deviation of $\sigma_{R,T}; T = 0,1; R = 1, \ldots, N_R$.

The prior distribution for the standard deviation of the residual error term in each arm are independent and identically distributed as:

$$\sigma_{R,T} \sim Unif(0,4); T = 0,1; R = 1, \ldots, N_r.$$

The uniform prior distribution is non-informative, ranging from 0 to 4 (over twice that observed in historical trials within the PRO-ACT database).

2.2.7 Baseline Mortality Hazard Rate

The baseline mortality hazard rate per month common across all placebo participants is λ_0. The prior distribution for the placebo mortality hazard rate is distributed as Gamma (shape and rate parametrization):

$$\lambda_0 \sim Gamma(.01,1);$$

with a mean of 0.01 and a standard deviation of 0.1. This prior distribution is centered on the value observed within the PRO-ACT database.

2.3 Posterior Summaries

The Bayesian posterior distribution of all model parameters is calculated using Markov chain Monte Carlo (MCMC). We will draw at least 500,000 samples from the posterior distribution with a thinning of 10. The exact number of samples from the MCMC will be determined based on convergence diagnostics to ensure an adequate effective sample size of the posterior distribution.

Summaries of the Bayesian posterior distribution for the DRR under the regimen-specific active treatment, $\exp(\theta_1)$, will be provided including the mean and 95% credible intervals; the probability that the treatment is superior to placebo, $Pr\,(\exp(\theta_1) < 1)$; and the probability the treatment slows the rate of progression by at least 10%, $Pr\,(\exp(\theta_1) < .9)$.

3.0 Success Thresholds, Interim Analyses and Trial Adaptations

Interim analyses will occur simultaneously for all actively enrolling regimens. Interims will begin after at least one regimen within the master protocol has 40 randomized participants with the opportunity to complete at least 24 weeks of follow-up. Subsequent interims will occur approximately every 12 weeks. At an interim analysis, a regimen can either stop

early for success or for futility. A regimen is eligible to stop early for success or for futility once there are 40 randomized participants within that regimen with the opportunity to complete at least 24 weeks of follow-up. If a regimen has not stopped early for success or futility at an interim analysis, the regimen-specific final analysis will take place after all participants randomized to that regimen (typically 120 active and 40 control) have had the opportunity to complete 24 weeks of follow-up.

3.1 Regimen Success

In the default design, early success stopping at interim analyses will not be conducted. Success will be declared for a regimen at the final analysis if the posterior probability that the treatment, *T=1*, is superior to placebo group is greater than .979:

$$Pr\,(\exp(\theta_1) < 1) > .979.$$

The threshold for the final analysis of .979 was chosen to control type I error at 2.5% across all Null simulation scenarios (except for when the analysis regimen has a slower rate of progression on ALSFRS-R compared to all other regimens) without futility stopping.

3.2 Regimen Futility

Futility will be declared for a regimen at an interim analysis if the posterior probability that the treatment, *T=1*, slows disease progression by at least 10% is less than 5%:

5.0

$Pr\,(\exp(\theta_1) < .9) < \ .05$.0 Goodness of Fit Diagnostics and Sensitivity Analyses of Primary Efficacy Analysis

5.0 The following goodness-of-fit diagnostics, supportive analyses, and sensitivity analyses will be performed for the primary efficacy analysis and will be conducted on the primary analysis population unless otherwise specified.*1 Goodness-of-fit Diagnostics*

The following goodness-of-fit diagnostics will be performed to assess the goodness-of-fit of the proposed Bayesian shared parameter analysis model:

- Residual plots for ALSFRS-R: residuals vs. fitted values of ALSFRS-R at each 4-week visit and for each treatment group.

- Mean and SD in change from baseline of ALSFRS-R at each 4-week visit for each treatment group compared to model fitted expected values from primary analysis model.
- Kaplan-Meier curve for each treatment group compared to fitted exponential curves from primary analysis model.

5.0 A table summarizing the number and percent of completed or expected ALSFRS-R assessments that do not contribute to the functional component of the primary analysis separated by visit and categorized by a) missing due to death or death equivalent, and b) missing for other reasons.*2 Key Supportive Joint Rank Analysis of ALSFRS-R and Mortality*

5.0 As a key supportive analysis, a joint rank test of survival and function will be performed. The analysis will rank all participants based first on survival time and second, if they survived, the change in ALSFRS-R from 0 to 24-weeks as described in Berry et al. 2013 (Berry, 2013). Given that participants may have different lengths of follow-up, comparisons will be made between any two participants based on the shortest duration of follow-up between the two. For example, if participant 1 has not experienced mortality and has ALSFRS-R values through 12 weeks and participant 2 has not experienced mortality and has ALSFRS-R values through 24 weeks, the comparison of the change from baseline at 12 weeks in ALSFRS-R will be made. Additionally, if participant 1 has ALSFRS-R values through 36 weeks and then experienced mortality at 36 weeks and participant 2 has ALSFRS-R values only through 24 weeks, a comparison is made based on ALSFRS-R values through 24 weeks. A linear regression analysis will be performed on the ranks to adjust for important covariates described in Section 2.2.5 using a fixed effect for each covariate as well as a treatment effect. This secondary analysis will be performed on the primary analysis population as well as the sensitivity analysis populations described in Section 4.3.*3 Sensitivity of Analysis Population*

As sensitivity analyses, the Bayesian primary analysis model will be applied to the following analysis populations:

- Concurrent Shared Control Population: All participants randomized to the active treatment arm or placebo arm within the analysis regimen and all participants randomized to the placebo arm across other regimens within the master protocol who have been randomized within a 6-month window of when the analysis regimen has been enrolling.
- Common Mode of Administration Population: All participants randomized to the active treatment arm or placebo arm within the analysis regimen and all participants randomized to the placebo arm across other regimens within the master protocol that have the same mode of administration.

5.0 Regimen Only Population: All participants randomized to the active treatment arm or placebo arm within the analysis regimen only. Because the population only involves one regimen, certain aspects of the analysis model will require modification (e.g. no regimen-specific effects).*4 Sensitivity Analysis for Handling Missing Data*

The Bayesian repeated measures component of ALSFRS-R naturally accommodates differential length of follow-up and utilizes all available longitudinal data for each participant who has not been censored due to mortality. Additionally, we provide the following sensitivity analyses to missing (not due to mortality) ALSFRS-R data within the Bayesian repeated measures component of ALSFRS-R.

- Impute all missing ALSFRS-R based on last observation carried forward.
- Impute all missing ALSFRS-R as 0 for both treatment and control arms.
- Impute all missing ALSFRS-R as the worst value observed within the combined treatment and control arms for both treatment and control arms.

5.0 Impute all missing ALSFRS-R as the worst value observed within the respective treatment/control arms.*5 Shared Parameter Model Sensitivity Analyses*

The following analyses will be conducted to test the sensitivity of the results to the primary efficacy model assumptions.

- The functional component of the primary analysis model, the Bayesian repeated measures model of ALSFRS-R, will be updated to allow for different rates of progression within each 4-week interval (k) to assess the sensitivity of the results to the linearity assumption in the primary efficacy analysis. Let $Y_{i,j,k}$ denote the ALSFRS-R at visit *j* for participant *i*. The ALSFRS-R component is then defined as:

$$Y_{i,j} = (\alpha_{r(i),t(i)} + \gamma_i) - \sum_{k=1:6} \mu_{\beta,k} * \exp\left(\beta_i + \ \theta_{t(i)} + \delta X_i + \rho_{d(i)} + \eta_{r(i)}\right) * \left(\frac{E_{i,j,k} * 12}{52}\right) + \epsilon_{i,j};$$

$$\epsilon_{ij} \sim N\left(0, \sigma^2_{r(i),t(i)}\right).$$

Where $E_{i,j,k}$ is the number of weeks of exposure for subjecI *i* in visit *j* within each 4-week interval *k*=1,2,…,6; $\mu_{\beta,k}$ is the overall slope for time interval *k*; and β_i is a common subject-level effect to the slope over time periods. The following prior distributions are placed on the overall mean slope per time period and the subject-level effect to the slope:

$$\mu_{\beta,k} \sim N(1,1^2); k = 1:6;$$
$$\beta_i \sim N\left(0, \sigma^2_{\beta,t(i)}\right); i = 1, \dots N;$$
$$\sigma_{\beta,T} \sim Unif(0,2); T = 0,1.$$

Prior distributions for all other parameters remain the same as the primary analysis.

- The shared parameter primary analysis model will be updated to allow for different treatment effects across time (4-week intervals) for the functional component and a different treatment effect between function and mortality.
- The shared parameter primary analysis model will be updated to allow for different treatment effects between function and mortality.
- The shared parameter primary analysis model will be updated to explore a range of degrees of fixed borrowing of controls across regimens. In particular, the variance of the regimen-specific random slope effects (Section 2.2.3) will be fixed across a set of values ranging from 0.001^2 (full borrowing) to 10^2 (no borrowing):

$$\eta_r \sim N(0, \sigma_\eta^2); r = 2, \ldots N_r;$$
$$\sigma_\eta^2 = .001^2,\ .01^2, .05^2, .1^2, .5^2, 1^2, 5^2, 10^2.$$

5.0 Summaries of the estimated treatment effect and estimated differences across regimens within each sensitivity analysis will be provided to assess the robustness of the primary analysis results to the degree of borrowing within the shared control. *.6 Sensitivity of Survival Endpoint*

As a sensitivity analysis, the Bayesian primary analysis model will be applied to the primary analysis population with the composite survival endpoint of death or death-equivalent replaced by a survival endpoint of death alone.

5.0 Clinical Trial Simulations

Clinical trial simulation is used to provided simulated example trials and to quantify operating characteristics for each regimen. In particular, virtual patient outcomes are created under different assumptions for key design parameters including placebo rates of progression for ALSFRS-R, measurement error for ALSFRS-R, mortality rates, treatment effects on ALSFRS-R, treatment effects on mortality, dropout rates and accrual rates. For each set of simulation assumptions (i.e., a scenario), many trials are simulated and virtually executed, including all interim analyses for early success and early futility. Trial operating characteristics are summarized across all simulated trials for each scenario.

5.1 Virtual Patient ALSFRS-R Simulation

Participant-level ALSFRS-R data at each timepoint is simulated using placebo data from the Pooled Resource Open-Access ALS Clinical Trials Database (PRO-ACT) with a bootstrap approach. In particular, a simulation database is created from the PRO-ACT database using each placebo participant with at least 3 ALSFRS-R measurements over the course of at least 24 weeks. For each of these participants, an intercept, slope and residual standard-error is calculated from a participant-specific linear regression of the measured ALSFRS-R within the PRO-ACT database as a function of the month of each measured visit. These linear regression values as well as baseline covariates are recorded for each participant within the simulation database.

Within the current simulations, a subset of the PRO-ACT simulation database is sampled based on those participants that have a time since onset less than 3 years and have a slow vital capacity or forced vital capacity (SVC / FVC) greater than 50% (N=2175). Within this subset, the average subject-specific slope (ALSFRS-R decline per month) is -1.03 with a SD of 0.76. The average residual standard error is 1.77.

To create a dataset of participant-level ALSFRS-R scores at each 4-week visit for each simulated clinical trial from the PRO-ACT simulation database, the following steps are taken:

- Choose a participant, *i,* from the PRO-ACT simulation database and record values of the participant-specific intercept (γ_i), slope (β_i), residual standard error (σ_i^2), and baseline covariates.
- Simulate participant-specific ALSFRS-R at 0, 4, 8, 12, 16, 20 and 24 weeks (j) from:
$$Y_{i,j} \sim N(\gamma_i + (\beta_i + \theta_{t(i)}) * \frac{j*12}{52}, \sigma_i^2);$$
assuming $\theta_0 = 0$ for placebo and θ_1 is found such that $(\hat{\beta} + \theta_1)/\hat{\beta}$ = 1- assumed % slowing of treatment; where $\hat{\beta}$ is the average slope across all subjects.

The treatment effect within the simulations is introduced as an additive effect to avoid introducing differences in the variance of the participant-specific rates of progression between the treatment and control arms. This is to provide a conservative estimate of

power as the multiplicative effect of treatment on the rate of progression decreases the variance of participant-level slopes in the treatment arm as the treatment becomes more effective.

5.2 Base Simulation Scenarios and Sensitivity Scenarios

All trial simulations assume there are 3 regimens within the platform; all are enrolling within the same population and randomizing concurrently (as defined in Section 2.1). Treatment effect assumptions will vary across the first regimen and operating characteristics will be reported for this regimen. The other two regimens will be assumed to be Null (no benefit on ALSFRS-R or mortality).

The trial simulations are performed under a base scenario (set of simulation assumptions) as well as numerous sensitivity scenarios. In particular, a simulation scenario is defined by 6 different parameters (Table 5.2.1). This set of 6 parameters characterizes various nuisance parameters, but also key assumptions on the endpoints such as their variability and the behavior of the control arm and treatment effect. We define a “base case” set of values for these 6 parameters as well as two sensitivity analysis values. The table below lists each of these 6 parameters along with the base case value and the two sensitivity analysis values.

Table 5.2.2 describes treatment effect assumptions. We simulate the base case of parameter values from Table 5.2.1 under all treatment effect scenarios in Table 5.2.2 and consider this as the primary set of operating characteristics for this trial (6 scenarios). Then, for each of 6 parameters that define a scenario, we vary one sensitivity analysis value at a time from the base case assumptions. For the first 5 parameter values we simulate under the null treatment effect and the common treatment effect with 30% slowing and a HR of .7 (10 sensitivity analyses across 2 treatment effect assumptions). For parameter 6 (mortality rate) we simulate under 4 treatment effect scenarios (Null, Common Effect with HR=.7 and Slowing=30%, No Mortality Benefit, and Worse Mortality). All sensitivity scenarios will be simulated with and without futility stopping.

Table 5.2.1: Simulation Scenario Parameter Assumptions

Parameter	Base Case	Sensitivity Analysis Values	
		a	b
1. Accrual rate (pts/mo)	40	20	60
2. Non-mortality dropout rate per month	2%	4%	6%
3. Timing of Start of Enrollment of Other Regimens	1 Month apart	3 Months apart	6 Months apart
4. ALSFRS-R Slope Distribution All Regimens	Same as PRO-ACT	10% Slower Progression and less variable	10% Faster Progression and more variable
5. ALSFRS-R Measurement Error All Regimens	Same as PRO-ACT	10% less	10% more

6. ALSFRS-R Differences in Slope Distribution Across Regimens	None	10% Slower Progression of Analysis Regimen	10% Faster Progression of Analysis Regimen
7. ALSFRS-R Differences in Measurement Error Across Regimens	None	10% less for Analysis Regimen	10% more for Analysis Regimen
8. 24-Week Mortality Rate	5%	10%	20%

Table 5.2.2: Treatment Effect Assumptions Mortality and Function

**Scenario**	**ALSFRS-R Slowing (1-DRR)**	**Mortality HR**
**Null**	0%	1.0
**Common Mortality and Function Benefit**	25%	.75
	30%	.70
	35%	.65
**No Mortality Benefit**	30%	1.0
**Worse Mortality**	30%	1.3

6.0 Example Trials and Operating Characteristics

For each scenario and treatment effect we simulate 10000 clinical trials. We present example simulated trials as well as overall average operating characteristics across all simulated trials. For each simulation scenario and treatment effect, we report the following regimen-specific operating characteristics for the first regimen:

- Mean duration (months)
- Mean sample size randomized to the regimen
- Probability of early success, total probability of success, and cumulative probability of stopping for success as a function of trial duration.
- Probability of early futility and cumulative probability of stopping for futility as a function of trial duration.
- Average estimate of the treatment effect and estimate of the treatment effect as a function of total trial duration.

6.1 Operating Characteristics Base Scenarios

Table 6.1.1 shows the overall operating characteristics for a single regimen under the master protocol recommended design for the Base simulation scenarios with futility stopping. Additionally, Table 6.1.2 summarizes the observed treatment effect estimates in each simulated trial as a function of the trial duration and outcome.

Under the null scenario there is an overall one-sided Type I error rate of 2.4% for the regimen. The average duration of the regimen is 14 months. There is a 28.0% chance that the regimen will be stopped early for futility.

The master protocol recommended design has 61%, 77%, and 88% power to detect a slowing in the rate of disease progression of 25%, 30% and 35% respectively when mortality and function have a common treatment effect. When there is no benefit in mortality and a 30% slowing in ALSFRS-R progression the power drops from 77% to 72%. When there is a negative effect on mortality and a 30% slowing in ALSFRS-R progression, the power drops further to 68%. Finally, there is less than 1% chance in these scenarios that the regimen will be stopped early for futility.

Given the expected accrual rate, interim analyses will begin approximately 7 months into the trial. Therefore, the final analysis for a regimen is expected to occur around 16 months after that regimen is initiated. Table 6.1.2 summarizes the observed treatment effect estimates that would lead to early stopping for futility or final success. If a regimen stops at the first interim analysis for futility, the average treatment effect estimate (DRR) is 1.35. The smallest observed DRR stopping for futility at this look across all simulated trials was 1.13, corresponding to a 13-35% increase in the rate of disease progression for treatment relative to placebo. At later analyses, 10-13 months from the start of the regimen, the average treatment effect estimate (DRR) observed among regimens that stopped for futility was 1.16-1.21 and the smallest observed DRR across all simulated trials observed to stop

for futility at these looks was 1.05-1.08, corresponding to a 5-8% increase in the rate of disease progression for treatment relative to placebo. Regimens never stopped early for futility if observed treatment effect was less than 1 (signaling a slowing in the rate of progression) and only stopped for futility if there was at least a 5% estimated increase in the rate of progression under treatment.

Table 6.1.1: Base Scenarios Operating Characteristics with Early stopping for Futility

Scenario (% Slowing ALSFRS-R)	HR Mort.	Mean Duration (Months)	Prob. Success	Prob. Early Futility	Mean Estimated DRR
**0%**	**1**	14	0.024	0.28	1.05
**25%**	**.75**	15	0.61	0.01	0.75
**30%**	**.7**	15	0.77	0.00	0.69
**35%**	**.65**	15	0.88	0.00	0.64
**30%**	**1**	15	0.72	0.00	0.71
**30%**	**1.3**	15	0.68	0.01	0.72

Table 6.1.2: Base Scenarios Observed Treatment Effect (DRR = 1 - % Slowing) Estimate per Trial Duration and Outcome

Interim /Final	Approx. Trial Duration (Months)	Early Futility		Success	
		Mean	Min	Mean	Max
**1**	**7**	1.35	1.13	-	-
**2**	**10**	1.21	1.08	-	-
**3**	**13**	1.16	1.05	-	-
**Final**	**16**	-	-	0.65	0.82

6.2 Operating Characteristics and Example Trials Integration of Mortality and Function

This section provides sensitivity of the overall operating characteristics to the assumptions of an overall 5% 6-month mortality rate as well as the assumption that mortality and function have a common treatment effect. All operating characteristics in this section assume no futility stopping. Additionally, we show results from two example trials to demonstrate how the proposed Bayesian shared parameter primary analysis accounts for mortality.

Table 6.2.1 provides Type I error, power and DRR estimates for the base scenario (5% 6-month mortality) as well as scenarios when the mortality rate is increased (10-20% 6-month mortality). Power and average DRR estimates are provided for treatment effect scenarios where the true DRR for mortality and function is the same and equal to .70 and when the true DRR for mortality and function is different (null or negative effect on mortality).

Under the null scenario when the true DRR equals 1.0 for function and true HR=1.0 for mortality, the type I error and the estimated DRR are not sensitive to the assumption of the percentage of 6-month mortality.

Under scenarios when the treatment effect for mortality is null or worse (true HR=1.0 or HR=1.3) and there is a 30% slowing in ALSFRS-R (true DRR=0.70), the power and estimated DRR decrease as the rate of mortality increases. This is due to the overall treatment effect being more heavily weighted by the mortality effect. The average estimated DRR equals 0.69 when the true DRR and HR parameters equal 0.70. Under a 20% 6-month mortality rate and a true DRR equal to 0.70, the average estimated DRR equals 0.75 and 0.81 when the true mortality HR equals 1.0 and 1.3, respectively, thereby decreasing the estimated functional treatment effect by 6-12% (absolute) compared to the scenario with both true DRR and true HR equal 0.70.

Table 6.2.1: Base and Sensitivity Scenarios on Combining Mortality and Function; No Early Futility Stopping

Scenario	DRR ALSFRS-R= 1.0 DRR Mort. = 1.0		DRR ALSFRS-R = .70 DRR Mort. = .70		DRR ALSFRS-R = .70 DRR Mort. = 1.0		DRR ALSFRS-R = .70 DRR Mort. = 1.3	
	Type I Error	Mean Est. DRR	Power	Mean Est. DRR	Power	Mean Est. DRR	Power	Mean Est. DRR
Base (5% Mort. Rate)	0.024	1.01	0.77	0.69	0.72	0.71	0.68	0.72
10% Mort. Rate	0.023	1.01	0.77	0.69	0.67	0.72	0.57	0.75
20% Mort. Rate	0.024	1.01	0.77	0.69	0.56	0.75	0.35	0.81

Additionally, to demonstrate that our model properly accounts for mortality and to show the sensitivity of the model to treatment-dependent differences in mortality, two simulated trials have been explored (Tables 6.2.2 and 6.2.3 and Figures 6.2.1-6.2.2). Within each simulated trial, we fix the ALSFRS-R data for each participant as well as the mortality data of participants in the placebo arm. We then let the mortality data on the active treatment arm vary to explore how the key supportive joint rank analysis (CAFS) and the proposed Bayesian shared-parameter primary analysis respond to an increasing number of deaths on the active treatment arm. The point at which inference about benefit from treatment switches from significant to non-significant, the tipping point, reveals how sensitive a given analysis is to an excess of deaths in the active arm. An analysis with a smaller tipping point is more sensitive and would be less likely to support a claim of benefit from the active treatment when the active treatment slows functional progression does not also reduce mortality rates.

The base simulations assume a substantial slowing in rate of functional decline of 30%. Within these example trials, as more participants on the active treatment arm experience mortality, the estimated disease rate ratio (DRR), the common treatment effect estimate between mortality and function, increases towards 1 (no effect of treatment) and the posterior probability that the treatment slows the rate of progression (DRR < 1) decreases. This is similar to the change seen in the CAFS joint rank analysis: as mortality in the active treatment arm increases, its p-value becomes less significative (see details below). The two example trials differ in the overall mortality rate (Example 1 has a higher rate than Example 2). Comparison of the two example trials shows that the sensitivity of the results to the mortality hazard ratio depends on the overall mortality rate (the mortality hazard ratio is smaller at the tipping point for Example 1 than for Example 2). These examples demonstrate that the proposed Bayesian shared-parameter model is more sensitive to discrepancies in the effect of treatment on mortality vs. the effect of treatment on function relative to the CAFS joint rank analysis.

For each example, we tabulate the following parameters across a range of mortality rates in the active treatment arm (Tables 6.2.2 and 6.2.3): the number of deaths on active treatment and shared placebo, the posterior DRR parameter estimates from the shared-parameter analysis vs. the sensitivity analysis that allows different DRR treatment effects between function and mortality, CAFS one-sided p-value, and posterior probability that the DRR is less than 1 from the shared parameter model. For Example 1, we present several graphs from specific representative trial simulations (the highlighted rows from Table 6.2.2; Plots 6.2.1-6.2.2). The top left plot shows estimates of change from baseline in ALSFRS-R through 24-weeks. The results include fitted values for the active treatment and the shared placebo from the Bayesian shared-parameter model (solid lines), fitted values for the active treatment and the shared placebo from the sensitivity analysis with different treatment effects on mortality and function (dotted lines), and raw mean values of the change from baseline at each visit (circles). The top right plot is a residual plot of fitted ALSFRS-R value (x-axis) at each visit vs. the residual ALSFRS-R value (y-axis) from the Bayesian shared-parameter model. The color of the circle represents active treatment vs. shared placebo. The size of the circle represents visit time (24-week visits are larger circles than 4-week visits). The bottom left plot shows Kaplan-Meier survival curves for mortality for the active

treatment and the shared placebo as well as the fitted exponential curves from the Bayesian shared-parameter model. The bottom right plot shows the DRR parameter estimates from the shared-parameter analysis vs. the sensitivity analysis that allows different DRR treatment effects between function and mortality.

Within Example Trial 1 (Table 6.2.2 and Figures 6.2.1-6.2.2), there are 18 deaths on the placebo arm (15%). Results are shown for 11-29 total deaths on the treatment arm with mortality hazard ratios from the mortality component alone ranging from 0.68 to 1.58. Simulations representative of rows highlighted in Table 6.2.2 are presented in Figures 6.2.1-6.2.2. The treatment effect estimated from the functional component alone for those participants who did not experience mortality range from 0.71-0.73. As the number of deaths increases on the active treatment arm, the posterior estimate for the shared treatment effect between mortality and function from the Bayesian shared-parameter model increases (0.73 to 0.88) and the posterior probability that the DRR is less than one decreases (0.994 to 0.853). Similarly, the CAFS one-sided p-value increases (0.002 to 0.233). For all mortality hazard ratios less than or equal to one, both CAFS and the Bayesian shared-parameter model indicate a significant treatment benefit. For the Bayesian shared-parameter model, the tipping point at which results become non-significant occurs between mortality hazard ratios of 1.04 and 1.10 (17 to 18 deaths in the active treatment arm). Under the CAFS analysis, the tipping point at which results become non-significant occurs between mortality hazard ratios of 1.15 to 1.27 (19 to 21 deaths on the active treatment arm). Under the simulated conditions, the Bayesian shared-parameter model was more sensitive to a discrepancy between the effect of treatment on function and mortality than the CAFS analysis.

Within Example Trial 2 (Table 6.2.3), there are 11 deaths on the placebo arm (9%). Results are shown for 6-19 total deaths on the treatment arm with mortality hazard ratios from the mortality component alone ranging from 0.66 to 1.57. The treatment effect estimated from the functional component alone for those participants who did not experience mortality ranges from 0.72-0.75. As the number of deaths increases on the active treatment arm, the posterior estimate for the shared treatment effect between mortality and function from the Bayesian shared-parameter model increases (0.71 to 0.85) and the posterior probability that the DRR is less than one decreases (0.998 to 0.917). Similarly, the CAFS one-sided p-value increases (0.001 to 0.085). For all mortality hazard ratios less than or equal to 1.13 both CAFS and the Bayesian shared-parameter model indicate a significant treatment benefit (CAFS p-value less than .025 and Bayesian posterior greater than 0.979). For the Bayesian shared-parameter model, the tipping point at which results become non-significant occurs between mortality hazard ratios of 1.13 to 1.21 (11 to 12 deaths on the active treatment arm). Under the CAFS analysis, the tipping point at which results become non-significant occurs between mortality hazard ratios of 1.27 to 1.33 (13 to 14 deaths on the active treatment arm). As in Example Trial 1, the Bayesian shared-parameter model was more sensitive to a discrepancy between the effect of treatment on function and mortality than the CAFS analysis. We also note that with the lower mortality rate, both the Bayesian shared-parameter analysis and the CAFS analysis were less sensitive to a discrepancy between the effect of treatment on function and mortality.

Table 6.2.2: Example Trial 1 demonstrating integration of mortality and function in primary analysis. Highlighted rows correspond to Figures 6.2.1 and 6.2.2						
Deaths PBO	Deaths TRT	Posterior Mean Estimate			CAFS P-Value	Posterior Probability of DRR <1
		Mortality DRR (HR)	Function DRR	Common DRR		
18	11	0.68	0.73	0.73	0.002*	0.994*
18	13	0.81	0.72	0.73	0.003*	0.991*
18	14	0.87	0.73	0.74	0.005*	0.988*
18	17	1.04	0.73	0.76	0.013*	0.982*
18	18	1.10	0.73	0.77	0.015*	0.975
18	19	1.15	0.73	0.78	0.022*	0.973
18	21	1.27	0.73	0.79	0.028	0.972
18	23	1.36	0.71	0.80	0.035	0.956
18	24	1.41	0.71	0.81	0.056	0.950
18	26	1.48	0.71	0.82	0.091	0.948
18	27	1.52	0.71	0.84	0.135	0.908
18	29	1.58	0.73	0.88	0.233	0.853

Table 6.2.3: Example Trial 2 demonstrating integration of mortality and function in primary analysis						
Deaths PBO	Deaths TRT	Posterior Mean Estimate			CAFS P-Value	Posterior Probability of DRR <1
		Mortality DRR (HR)	Function DRR	Common DRR		
11	6	0.66	0.72	0.71	0.001*	0.998*
11	7	0.77	0.72	0.73	0.003*	0.993*
11	8	0.87	0.74	0.74	0.004*	0.992*
11	9	0.96	0.74	0.75	0.006*	0.988*
11	10	1.06	0.73	0.75	0.009*	0.982*
11	11	1.13	0.74	0.78	0.013*	0.979*
11	12	1.21	0.74	0.78	0.014*	0.976
11	13	1.27	0.73	0.78	0.017*	0.975
11	14	1.33	0.74	0.80	0.028	0.961
11	16	1.45	0.73	0.80	0.036	0.959
11	17	1.49	0.74	0.82	0.058	0.939
11	19	1.57	0.75	0.85	0.085	0.917

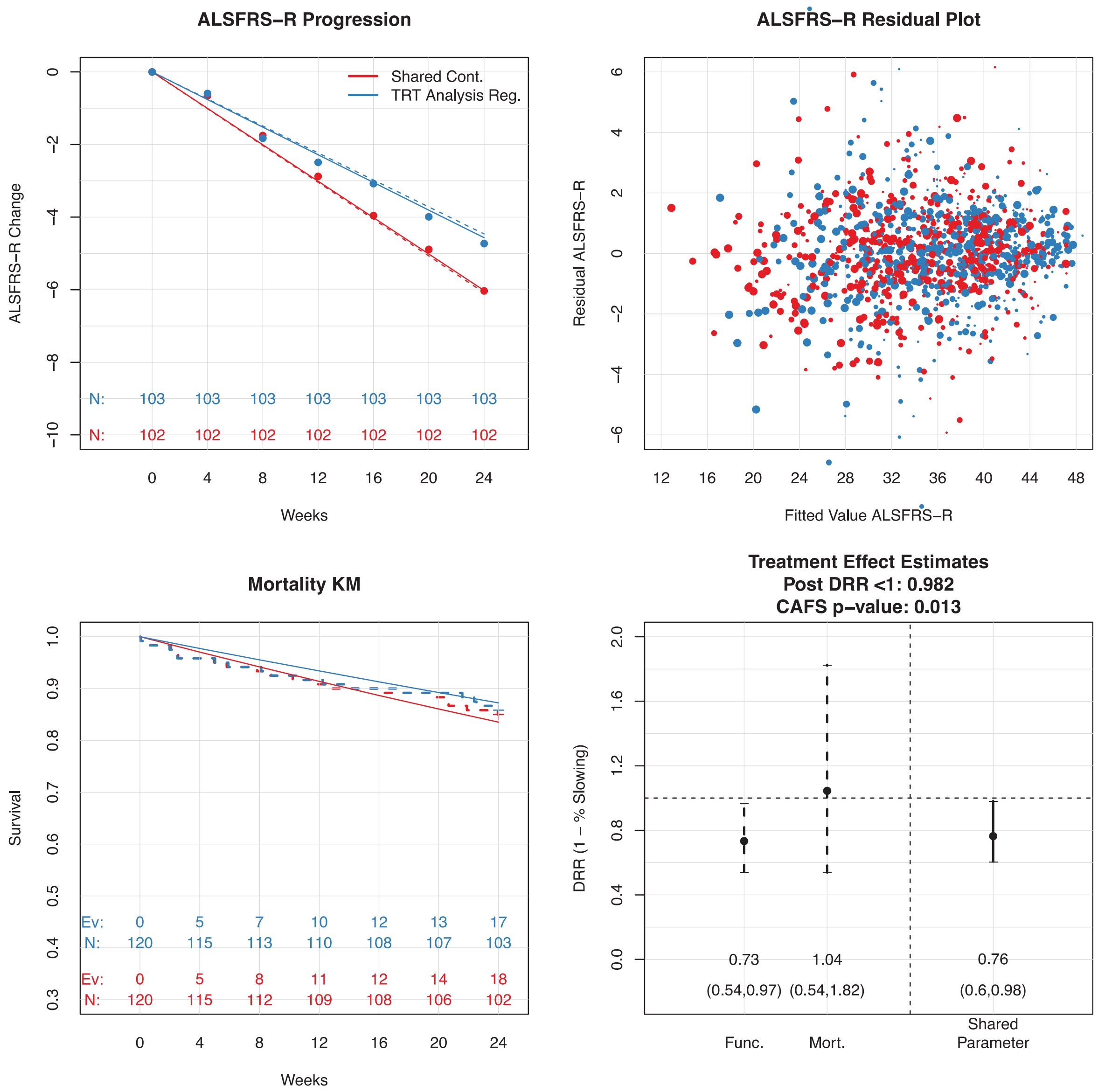

Figure 6.2.1: Example Trial 1 with 18 Deaths on Placebo and 17 Deaths on Treatment demonstrating integration of mortality and function in primary analysis.

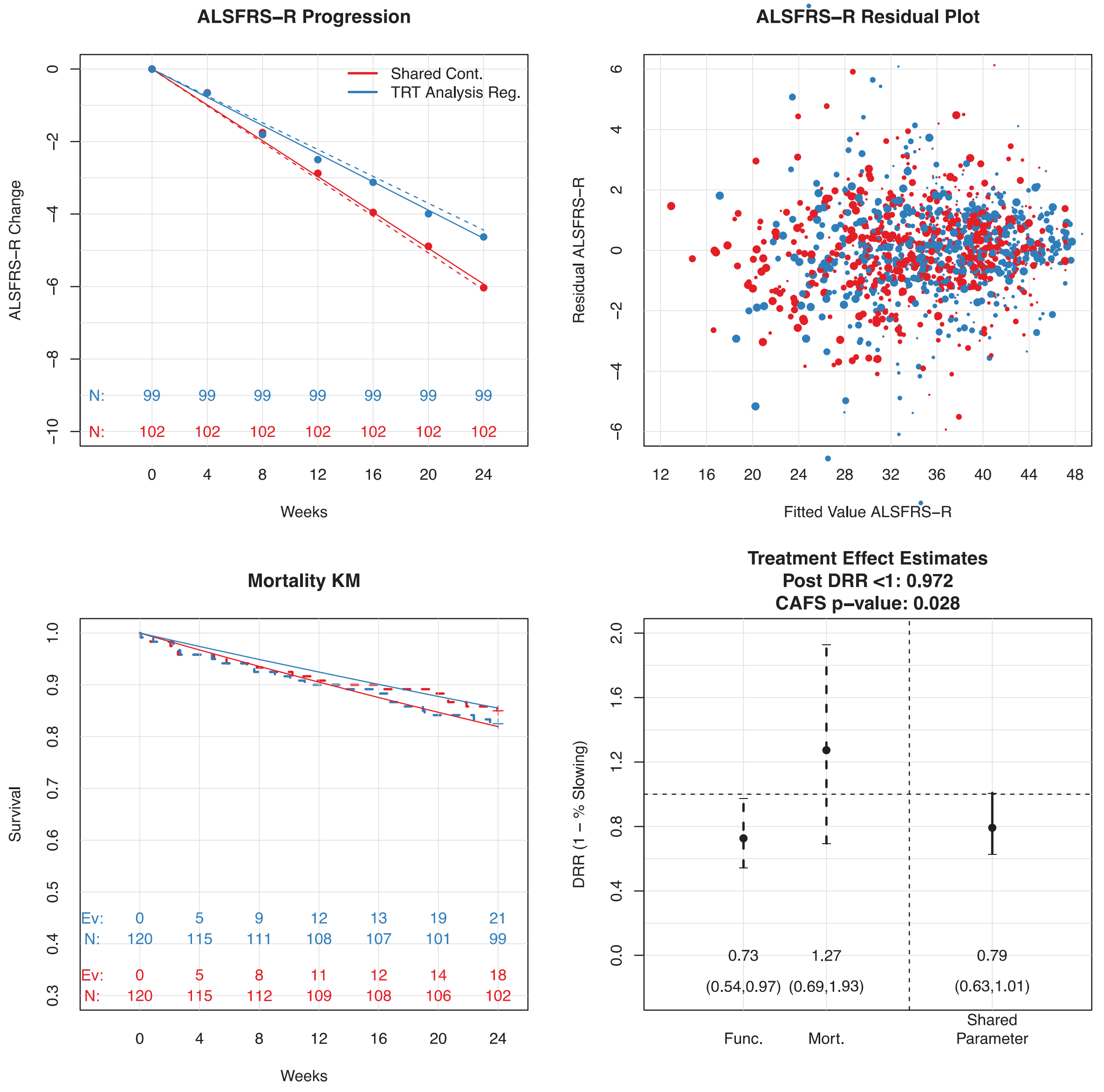

Figure 6.2.2: Example Trial 1 with 18 Deaths on Placebo and 21 Deaths on Treatment demonstrating integration of mortality and function in primary analysis.

6.3 Operating Characteristics and Example Trials Differences Across Regimens

This section provides sensitivity of the overall operating characteristics to the assumption that regimens within the shared control have the same ALSFRS-R distributions. In particular, we look at the sensitivity of operating characteristics to differences across the regimens in the rate of progression of ALSFRS-R as well as differences in the residual error of ALSFRS-R. All operating characteristics in this section assume no futility stopping.

Table 6.3 provides Type I error, power and DRR estimates for the base scenario (all regimens have the same ALSFRS-R progression rate and residual error) as well as scenarios when the analysis regimen differs from all other regimens in terms of the ALSFRS-R progression rate and ALSFRS-R residual error. Power and DRR estimates are provided for treatment effect scenarios where the DRR for mortality and function is the same and equal to .70 and when the DRR for mortality and function is different (null or negative effect on mortality).

Table 6.3: Base and Sensitivity Scenarios on Differences Across Regimens; No Early Futility Stopping; Common Mortality and Function Effect

Scenario	Null DRR 1.0		Alternative DRR 0.70	
	Type I Error	Mean Est. DRR	Power	Mean Est. DRR
Base	0.024	1.01	0.77	0.69
ALSFRS-R Slower Progress Analysis Reg.	0.060	0.96	0.85	0.66
ALSFRS-R Faster Progress Analysis Reg.	0.010	1.05	0.67	0.72
ALSFRS-R Lower Resid. Error Analysis Reg.	0.024	1.01	0.79	0.69
ALSFRS-R Higher Resid. Error Analysis Reg.	0.023	1.01	0.75	0.69

Under the extreme assumption that participants in the analysis regimen progress at a 10% slower rate of progression on ALSFRS-R than those in all other regimens, there is an increased Type I error of 6% and a small positive bias in the treatment effect estimate of .96 (4% slowing in progression). Under the assumption that participants in the analysis regimen progress at a 10% faster rate of progression on ALSFRS-R than those in all other regimens, the Type I error decreases to 1% with a small negative bias in the treatment effect estimate equal to 1.05 (5% increase in progression). The sensitivity of these results is due to the borrowing across regimens for the shared control.

Operating characteristics are not sensitive to differences in the ALSFRS-R residual error for the analysis regimen compared to other regimens other than having an increase or decrease in power when ALSFRS-R under the analysis regimen has more or less residual error.

In addition to the primary analysis, sensitivity analyses will be provided with regards to the shared control (Section 4.2) to assess differences in rates of progression across regimens and how treatment effect estimates differ under the analysis regimen when the treated

group is compared to only the regimen-specific controls and compared to only the controls with the same mode of administration. We provide examples from simulated trials (Figures 6.3.1-6.3.3) to demonstrate how discrepancies between the primary analysis and proposed sensitivity analyses will address the concern of a potential type I error and bias due to sharing of controls that are heterogenous.

For each simulated example trial, the left plots show results from the primary shared-parameter model for the change from baseline in ALSFRS-R through 24 weeks using all shared controls (top) and for the sensitivity analysis using only regimen-specific controls (bottom). The results include fitted values for the active treatment and for each regimen-specific control (blue line for active treatment; red line for matching control from analysis regimen; pink for additional shared controls when included in the analysis) and raw mean values in the change from baseline at each visit (circles). The middle plots show mortality KM plots as well as exponential fitted curves for the primary analysis using all shared controls (top) vs. the sensitivity analysis with only regimen-specific controls (bottom). The right plots show the DRR parameter estimates, i.e., the posterior probability of slowing progression (DRR<1), from the primary analysis method using all shared controls (top) vs. the sensitivity analysis using only regimen-specific controls (bottom). Additionally, we note the one-sided p-value from the key supportive CAFS analysis using all shared controls (top right) compared to only regimen-specific controls (bottom right).

Figure 6.3.1 shows results from a null simulated trial (no treatment effect) where there are differences across regimens in the shared control. In particular, the matched regimen-specific control from the analysis regimen has a slower rate of progression on ALSFRS-R (red circles) than the controls from the other regimens in the shared control (pink circles). The raw mean change from baseline at 24 weeks in the matched regimen-specific controls is approximately -4.5 and the mean change from baseline at 24 weeks in the other shared controls is approximately -6.5 and -7.2. These differences across regimens within the shared control lead to a fitted ALSFRS-R slope for the matched regimen-specific control to be pulled down and estimated to be larger (faster progression) than what is observed in that regimen only (red line falls below red circles). This causes a positive treatment effect bias in the DRR (estimated to be 0.73) under the primary analysis method. The posterior probability that the DRR is less than 1 is 0.990 and the corresponding one-sided p-value from the CAFS is 0.0063. Both analysis methods using shared controls would lead to a type I error. In comparison, the sensitivity analysis using only matched regimen-specific controls leads to a fitted value for the regimen-only controls ALSFRS-R slope that estimates slower decline than what was estimated using all shared controls. This results in a smaller treatment effect estimate of 0.91 under the primary shared-parameter analysis. The posterior probability that the DRR is less than 1 is 0.683 and the corresponding one-sided p-value from the CAFS is 0.5525. The clear discrepancies between the primary analysis and the proposed sensitivity analysis using only regimen-specific controls in the Bayesian shared parameter analysis as well as the CAFS analysis demonstrates that the positive result in the primary analysis may be a type I error due to the sharing of heterogenous controls.

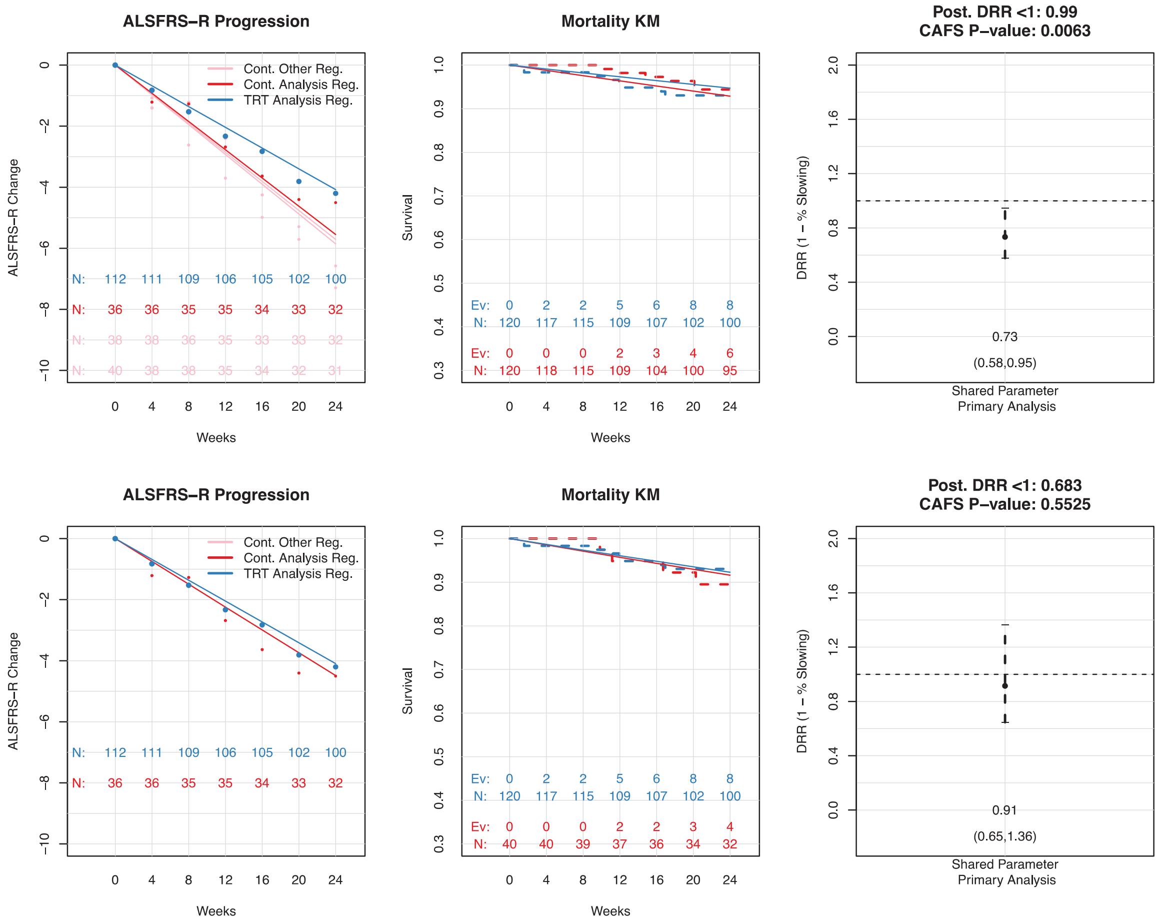

Figure 6.3.1: *Example Trial 3. Null Treatment Effect (DRR = 1.0). Differences across regimens in shared control. Top row shows results from the primary analysis using all shared controls. Bottom row shows results from sensitivity analyses using only matched regimen-specific controls.*

Figure 6.3.2 shows results from a null simulated trial (no treatment effect) where there are more substantial differences across regimens in the shared control than in the previous example. The raw mean change from baseline at 24 weeks in the matched regimen-specific controls is approximately -4.0 and the mean change from baseline at 24 weeks in the other shared controls is approximately -7.0 for both. Because the Bayesian primary analysis allows for dynamic sharing of controls (more sharing if they look similar vs. less sharing if they look different) these substantial differences across regimens allows the model to learn the controls are likely very different across regimens and leads to a fitted ALSFRS-R slope for the matched regimen-specific control that is less influenced and pulled down by the controls of the other two regimens than in the previous example (red line falls slightly below red circles). This causes a slight positive treatment effect bias in the DRR (estimated to be 0.90) under the primary analysis method. The posterior probability that the DRR is less than 1 is 0.714 and the Bayesian shared parameter analysis leads to a null result. However, because the CAFS analysis uses all shared controls and the amount of sharing is not dynamic, the results of this analysis are more sensitive to the increased rate of progression in the other two control groups and the corresponding one-sided p-value from the CAFS is 0.0026. Leading to a Type I error in the CAFS analysis but not the Bayesian

shared parameter analysis. When matched regimen-specific controls are only used, both the Bayesian shared parameter analysis and the CAFS analysis lead to a null result, (treatment effect estimate of 1.0, posterior probability DRR<1 of 0.51 and CAFS p-value of -.3675).

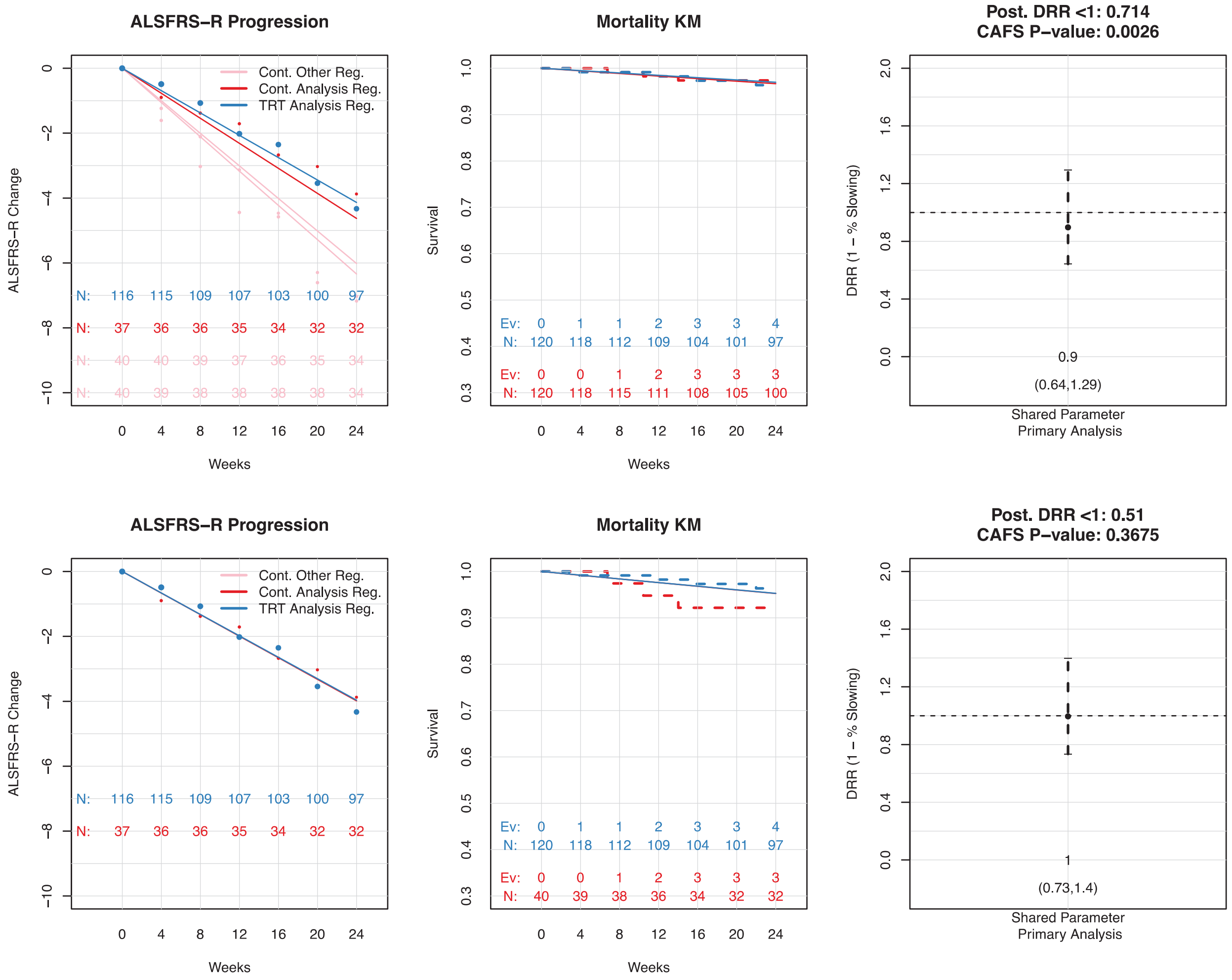

Figure 6.3.2: *Example Trial 4. Null Treatment Effect (DRR = 1.00). Differences across regimens in shared control. Top row shows results from the primary analysis using all shared controls. Bottom row shows results from sensitivity analyses using only matched regimen-specific controls.*

Figure 6.3.3 shows results from a positive simulated trial (30% slowing in disease progression) where there are no simulated differences across regimens in the shared control. Within this example, the matched regimen-specific control from the analysis regimen has a similar rate of progression on ALSFRS-R (red circles) as the controls from the other regimens in the shared control (pink circles). In particular, the raw mean change from baseline at 24 weeks in the matched regimen-specific controls is approximately -5.9 and the mean change from baseline at 24 weeks in the other shared controls is approximately -5.9 and -7.2. The similarities across regimens within the shared control leads to a fitted ALSFRS-R slope for the matched regimen-specific control that provides a

good fit to the observed data (red line falls within red circles). The treatment effect is estimated to be 0.67, the posterior probability that the DRR is less than 1 is 0.998, and the corresponding one-sided p-value from the CAFS is 0.0088. The sensitivity analysis using only matched regimen-specific controls leads to a similar treatment effect estimate of 0.73, but with more uncertainty. The posterior probability that the DRR is less than 1 is 0.931 and the corresponding one-sided p-value from the CAFS is 0.1102. The consistency in results between the primary analysis using all shared controls as well as the sensitivity analysis using only matched regimen-specific controls provides confidence that the positive primary analysis results are not biased by the shared controls.

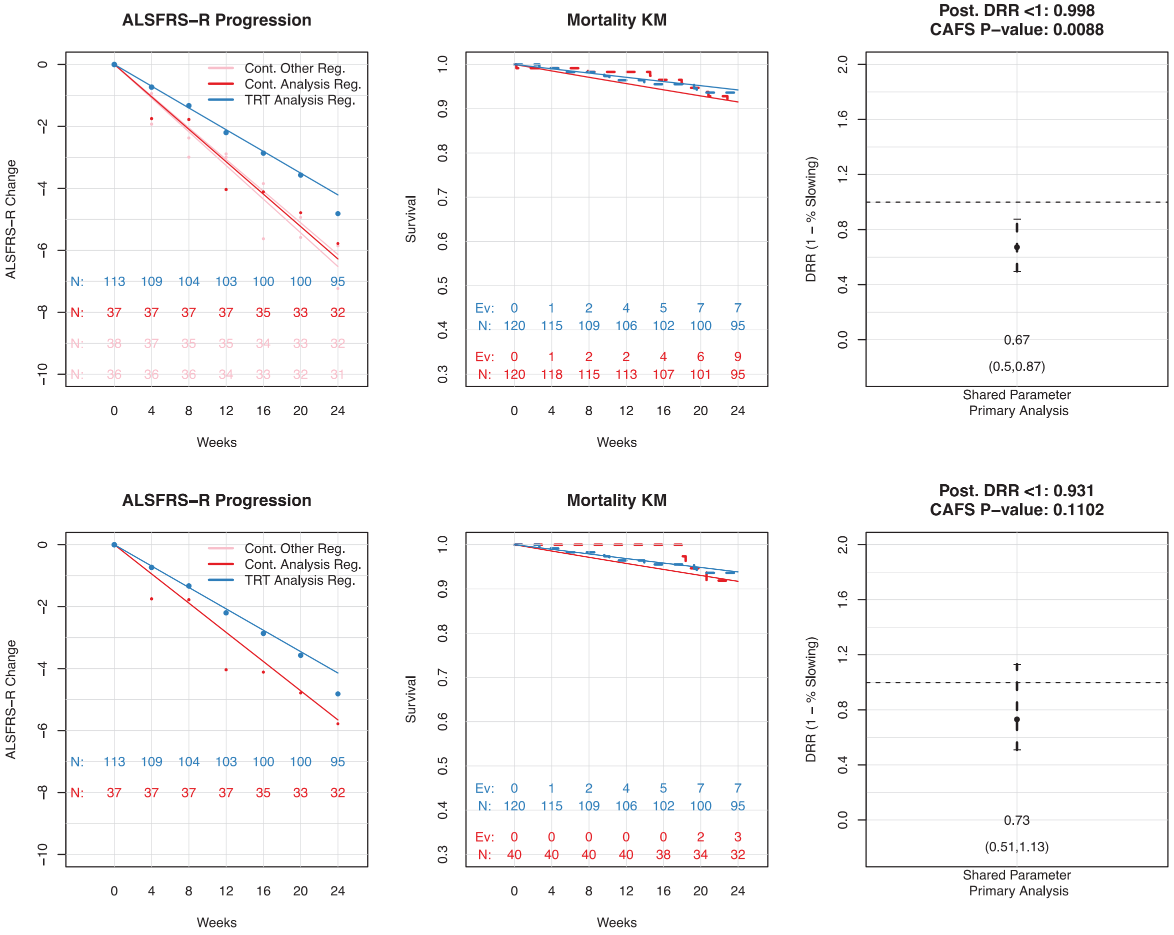

Figure 6.3.3: *Example Trial 5. Positive Treatment Effect (DRR = 0.70). No differences across regimens in shared control. Top row shows results from the primary analysis using all shared controls. Bottom row shows results from sensitivity analyses using only matched regimen-specific controls.*

6.4 Operating Characteristics Additional Sensitivity Scenarios

This section provides additional sensitivity results of the overall operating characteristics to the assumptions of accrual rates, dropout rates, timing of enrolling additional regimens, and distributional assumptions of ALSFRS-R common to all regimens. All operating characteristics in this section assume no futility stopping.

Table 6.4 shows the overall power (under a DRR of .70 for Mortality and Function) and Type I error for the base scenarios as well as all additional sensitivity scenarios described in Section 5.2 without futility stopping.

Under all Null scenarios, Type I error is controlled at 2.5% or less without futility stopping. Type I error decreases as there is less shared controls in the final analysis (faster accrual and regimens enrolling further apart).

Under all scenarios where the treatment effect is common between ALSFRS-R slowing and mortality (DRR is 0.70) the power of the trial ranges between 56-79%. Power is the lowest when the regimens start six months apart since the first regimen will have less shared control. Mean DRR estimates range from 0.69 – 0.71 (29-31% slowing).

Table 6.4: Base and Sensitivity Scenarios with No Early Futility Stopping; Common Mortality and Function Effect

Scenario	Null DRR 1.0		Alternative DRR 0.70	
	Type I Error	Mean Est. DRR	Power	Mean Est. DRR
Base	0.024	1.01	0.77	0.69
Slower Accrual	0.025	1.01	0.77	0.69
Faster Accrual	0.020	1.01	0.76	0.69
4% Dropout	0.024	1.01	0.74	0.69
6% Dropout	0.024	1.01	0.71	0.69
Reg. Start 3 Months Apart	0.023	1.02	0.67	0.70
Reg. Start 6 Months Apart	0.021	1.03	0.56	0.71
ALSFRS-R Slower Progress All Reg.	0.023	1.01	0.75	0.69
ALSFRS-R Faster Progress All Reg.	0.023	1.01	0.78	0.69
ALSFRS-R Lower Resid. Error All Reg.	0.023	1.01	0.79	0.69
ALSFRS-R Higher Resid. Error All Reg.	0.023	1.01	0.75	0.69

7.0 References

Berry, J. D., et al., 2013. The Combined Assessment of Function and Survival (CAFS): a new endpoint for ALS clinical trials. *Amyotrophic Lateral Sclerosis and Frontotemporal Degeneration,* Volume 14.3, pp. 162-168.
