# Supplementary material for: Verdiperstat in Amyotrophic Lateral Sclerosis: Results From the Randomized HEALEY ALS Platform Trial
Source: JAMA Neurol. 2025 Feb 17;82(4):333–43. doi: 10.1001/jamaneurol.2024.5249 (PMC11833655; doi:10.1001/jamaneurol.2024.5249)
Supplement: Supplement 2. — Revised Trial Protocol. [file jamaneurol-e245249-s002.pdf]

REGIMEN-SPECIFIC APPENDIX [B]

FOR VERDIPERSTAT (BHV-3241)

Regimen-Specific Appendix Date: 02 December 2021

Version Number: 6.0

TABLE OF CONTENTS

SIGNATURE PAGE 5
LIST OF ABBREVIATIONS 6
REGIMEN-SPECIFIC APPENDIX (RSA) SUMMARY 9
SCHEDULE OF ACTIVITIES – 24-WEEK PLACEBO-CONTROLLED PHASE 11
SCHEDULE OF ACTIVITIES – REGIMEN-SPECIFIC OPEN-LABEL EXTENSION PHASE (OPTIONAL) 15
1 INTRODUCTION REGIMEN B: VERDIPERSTAT (BHV-3241) 17
1.1 Verdiperstat Background Information 17
1.1.1 Primary Pharmacodynamics 17
1.1.2 Secondary Pharmacodynamics 18
1.1.3 Safety Pharmacology 18
1.2 Verdiperstat Rationale 19
1.2.1 Dosage Selection and Justification 20
2 STUDY OBJECTIVES AND ENDPOINTS 21
3 RSA DESIGN 22
3.1 Scientific Rationale for RSA Design 22
3.3 End of Regimen Definition 23
4 RSA ENROLLMENT 24
4.1 Number of Study Participants 24
4.2 Additional RSA Inclusion and Exclusion Criteria 24
4.2.1 RSA Inclusion Criteria 24
4.2.2 RSA Exclusion Criteria 24
4.3 Treatment Assignment Procedures 24
5 INVESTIGATIONAL PRODUCT 25
5.1 Investigational Product Manufacturer 25
5.2 Labeling, Packaging, and Resupply 25
5.2.1 Labeling 25
5.2.2 Acquisition and Preparation 25

5.2.3 *Drug Returns and Destruction* 26

5.3 Study Medication/Intervention, Administration and Duration, and Titration 26

5.3.1 *Administration and Duration* 26

5.3.2 *Dosage Modification* 27

5.4 Drug Holiday 28

5.5 Participant Compliance 28

5.6 Justification for Dosage 28

5.7 Overdose 28

5.8 Drug-Drug interactions and Prohibited Medications 29

5.8.1 *Effect of other drugs on verdiperstat metabolism* 29

5.8.2 *Effect of verdiperstat on the metabolism of other drugs:* 29

5.9 Verdiperstat Known Potential Risks and Benefits 30

5.9.1 *Known Potential Risks* 30

5.9.2 *Known Potential Benefits* 33

6 REGIMEN SCHEDULE 34

6.1 Placebo-Controlled Period 35

6.1.1 *Verdiperstat Regimen Specific Screening Visit* 35

6.1.2 *Baseline Visit* 35

6.1.3 *Week 2 Telephone Visit* 36

6.1.4 *Week 4 Visit* 36

6.1.5 *Week 8 Visit* 36

6.1.6 *Week 12 Telephone Visit* 37

6.1.7 *Week 16 Visit* 37

6.1.8 *Week 20 Telephone Visit* 37

6.1.9 *Week 24 Visit or Early Termination Visit* 38

6.1.10 *Follow-Up Safety Call* 39

6.1.11 *Process for Early Terminations* 39

6.1.12 *Criteria for Participant Termination* 39

6.2 Open Label Extension (OLE) 40

6.2.1 *Week 2 Telephone Visit - OLE* 41

6.2.2 *Week 4 Visit – OLE* 41

6.2.3 *Week 8 Visit - OLE* 41

6.2.4 *Week 12 Telephone Visit – OLE* 41

6.2.5 *Week 16 Visit – OLE* 41

6.2.6 Week 20 Telephone Visit – OLE ... 42
6.2.7 Week 24 Telephone Visit – OLE ... 42
6.2.8 Week 28 Visit and Q12 Weeks – OLE ... 42
7 OUTCOME MEASURES AND ASSESSMENTS ... 43
7.1 Voice Analysis ... 43
7.2 ALSAQ-40 ... 43
7.3 Center for Neurologic Study Bulbar Function Scale ... 43
8 BIOFLUID COLLECTION ... 44
8.2 Pharmacokinetic Assessments ... 44
8.3 Pharmacodynamic Biomarker Assessments ... 44
9 SAFETY AND ADVERSE EVENTS ... 45
9.1 Adverse Events of Special Interest – Thyroid Function ... 45
10 REGIMEN-SPECIFIC STATISTICAL CONSIDERATIONS ... 46
10.1 Deviations from the Default Master Protocol Trial Design ... 46
10.2 Regimen Specific Operating Characteristics ... 46
10.3 Sharing of Controls from Other Regimens ... 50
11 REFERENCES ... 51
Appendix I: The Bulbar Function Scale (CNS-BFS) ... 53
Appendix II: The ALSAQ-40 ... 56

List of Figures

Figure 1. Regimen Specific Appendix Schematic ... 22

SIGNATURE PAGE

I have read the Regimen-Specific Appendix (RSA) entitled, Verdiperstat (BHV-3241) in Participants with Amyotrophic Lateral Sclerosis (ALS) dated July 15, 2021 (Version 5.0.) and agree to abide by all described RSA procedures. I agree to comply with the International Conference on Harmonisation Tripartite Guideline on Good Clinical Practice, applicable FDA regulations and guidelines identified in 21 CFR Parts 11, 50, 54, and 312, central Institutional Review Board (IRB) guidelines and policies, and the Health Insurance Portability and Accountability Act (HIPAA).

By signing the RSA, I agree to keep all information provided in strict confidence and to request the same from my staff. Study documents will be stored appropriately to ensure their confidentiality. I will not disclose such information to others without authorization, except to the extent necessary to conduct the study.

Site Name: ____

Site Investigator: ____

Signed: ______________________ Date: ____________

LIST OF ABBREVIATIONS

AE	Adverse Event
ALP	Alkaline Phosphatase
ALS	Amyotrophic Lateral Sclerosis
ALSFRS-R	ALS Functional Rating Scale-Revised
ALSAQ-40	Amyotrophic Lateral Sclerosis Assessment Questionnaire
Anti-TPO	Anti-Thyroid Peroxidase
AUC	Area Under the Curve
BHV-3241	Verdiperstat
BID	Twice daily
°C	Degrees Celsius
CBC	Complete blood count
C_{max}	Maximum Plasma Concentration
CNS	Central Nervous System
CRF	Case Report Form
CSF	Cerebrospinal fluid
C-SSRS	Columbia-Suicide Severity Rating Scale
CYP	Cytochrome P450
DNA	Deoxyribonucleic Acid
ECG	Electrocardiogram
eCRF	Electronic Case Report Form
ER	Extended Release
°F	Degrees Fahrenheit
FVC	Forced Vital Capacity
GRAS	Generally recognized as safe
HDPE	High-density polyethylene
HHD	Hand Held Dynamometry
HIPAA	Health Insurance Portability and Accountability Act
HR	Hazard Ratio

IB	Investigator Brochure
ICF/IC	Informed Consent Form
ICH	International Council on Harmonization
IP	Investigational Product
IRB	Institutional Review Board
IWRS	Interactive Web-Based Response System
LPLV	Last participant last visit
MAD	Modification of Diet in Renal Disease
mg	Milligram
MPO	Myeloperoxidase
MSA	Multiple System Atrophy
NADPH	Nicotinamide adenine dinucleotide phosphate
NfL	Neurofilament Light Chain
NOEL	No-Observed-Effect-Level
NOX	NADPH oxidase
NRF2	Nuclear factor erythroid 2–related factor 2
OLE	Open-Label Extension
PD	Pharmacodynamic
PET	Positron emission tomography
P-gp	P-glycoprotein
PK	Pharmacokinetic
PMA	Phorbol-12-myristate-13-acetate
PP	Polypropylene
QD	Once Daily
QTcF	Corrected QT Interval by Fridericia
ROS	Reactive oxygen species
RNS	Reactive nitrogen species
RSA	Regimen-specific appendix
SAD	Single Ascending Dose
SAE	Serious Adverse Event

SI	Site Investigator
SOA	Schedule of Activities
SOD	Superoxide dismutase
SVC	Slow Vital Capacity
T3	Triiodothyronine
T4	Thyroxine
TSPO	Translocator Protein
TPO	Thyroid Peroxidase
TSH	Thyroid Stimulating Hormone
TSPO	Translocator Protein
USB	Universal Serial Bus
USP	US Pharmacopoeia
VC	Vital Capacity
WOCBP	Women of Childbearing Potential

REGIMEN-SPECIFIC APPENDIX (RSA) SUMMARY

Regimen-Specific Appendix [B]

For verdiperstat (also known as BHV-3241).

Rationale and RSA Design

The proposed study is based on cumulative preclinical and clinical studies that implicate myeloperoxidase (MPO) activity in the onset and progression of neurodegenerative diseases and suggest treatment with verdiperstat at a dosage of 600 mg twice daily (BID) has the potential to slow neurodegeneration in Amyotrophic Lateral Sclerosis (ALS).

Allocation to Treatment Regimens

Participants must first be screened under the Master Protocol before they are randomized to an RSA. As soon as pre-defined criteria for futility for the RSA are met, or the target number of randomized participants for the RSA has been reached, enrollment will stop in the RSA.

Number of Planned Participants and Treatment Groups

The number of planned participants for this regimen is approximately 160.

There are 2 treatment groups for this regimen, active and placebo. Participants will be randomized in a 3:1 ratio to active treatment or placebo (i.e., 120 active: 40 placebo).

Planned Number of Sites

Research participants will be enrolled from approximately 60 centers in the US.

Treatment Duration

The maximum duration of the placebo-controlled portion is 24 weeks.

Follow-up Duration

At the conclusion of the 24-week placebo-controlled period of the study, all participants will either schedule a 28-day follow up phone call and end their participation in the regimen or have the option to receive verdiperstat in the open-label extension (OLE) phase of the study.

The OLE portion of the study will continue until verdiperstat is approved and available in the United States, or Biohaven Pharmaceuticals terminates development of verdiperstat for ALS. At the completion of the OLE, a Follow-up Safety Call should be conducted approximately 28 days after the last dose of study drug.

For participants who early terminate from the placebo-controlled phase, an in-person Early Termination Visit and a Follow-up Safety Call should be conducted. At the Early Termination Visit the same procedures as described for the Week 24 visit should be conducted. At the Follow-up Safety Call, information on clinical status should be collected.

For participants who early terminate from the OLE, an in-person Early Termination Visit and a Follow-up Safety Call should be conducted. At the Early Termination Visit the same procedures as described for the Final visit should be conducted. At the Follow-up Safety Call, information on clinical status should be collected.

Total Planned Trial Duration

For participants completing only the placebo-controlled Treatment Period of the study, the planned amount of time for participation in the trial is 34 weeks, or about 8 months. This duration assumes a 6-week screening window, a 24-week placebo-controlled treatment period, and a 28-day safety follow-up period for those participants who do not enter the OLE. Participants will complete approximately 10 study visits during the placebo-controlled period of the study.

SCHEDULE OF ACTIVITIES – 24-WEEK PLACEBO-CONTROLLED PHASE

As per the Schedule of Activities (SOA) below, visits must occur every 4 weeks and will be alternatively clinic-, phone-, or telemedicine-based, as applicable. There is a maximum 24-week duration of placebo-controlled treatment for a Regimen.

**Activity**	**Master Protocol or Regimen-Specific**	**Master Protocol Screening[1]**	**Regimen Specific Screening[1]**	**Baseline**	**Week 2[2]**	**Week 4[18,19]**	**Week 8[18,19]**	**Week 12**	**Week 16[18,19]**	**Week 20**	**Week 24 or Early Term. Visit[3, 18]**	**Follow-up Safety Call[3,4]**
		Clinic	Clinic	Clinic	Phone	Clinic	Clinic	Phone	Clinic	Phone	Clinic	Phone
		-42 to -1 Days	-41 to 0 Days	Day 0	Day 14 ±3	Day 28 ±7	Day 56 ±7	Day 84 ±3	Day 112 ±7	Day 140 ±3	Day 168 ±7	28±7 days after last dose
Written Informed Consent – Placebo-controlled Period[5]	**Master**	X	X									
Written Informed Consent - OLE	**Regimen**								X			
Inclusion/Exclusion Review	**Master**	X	X[6]									
ALS & Medical History	**Master**	X										
Demographics	**Master**	X										
Physical Examination	**Master**	X										
Neurological Exam	**Master**	X										
Vital Signs[7]	**Master**	X		X		X	X		X		X	
Slow Vital Capacity	**Master**	X[20]		X			X		X		X	
Home Spirometry	**Regimen**	X[20]		X			X		X		X	
Muscle Strength Assessment	**Master**			X			X		X		X	
ALSFRS-R	**Master**	X		X		X	X	X	X	X	X	
ALSAQ-40	**Regimen**			X							X	
CNS bulbar Function Scale	**Regimen**			X			X		X		X	
12-Lead ECG	**Regimen**	X					X				X	

Activity	Master Protocol or Regimen-Specific	Master Protocol Screening[1]	Regimen Specific Screening[1]	Baseline	Week 2[2]	Week 4[18,19]	Week 8[18,19]	Week 12	Week 16[18,19]	Week 20	Week 24 or Early Term. Visit[3, 18]	Follow-up Safety Call[3,4]
		Clinic	Clinic	Clinic	Phone	Clinic	Clinic	Phone	Clinic	Phone	Clinic	Phone
		-42 to -1 Days	-41 to 0 Days	Day 0	Day 14 ±3	Day 28 ±7	Day 56 ±7	Day 84 ±3	Day 112 ±7	Day 140 ±3	Day 168 ±7	28±7 days after last dose
Clinical Safety Labs[8]	Master	X		X		X	X		X		X	
Verdiperstat (BHV-3241specific PD biomarkers[10]	Regimen			X		X	X		X		X	
PK Blood Collection[10]	Regimen			X		X	X		X		X	
Biomarker Blood Collection	Master			X			X		X		X	
Biomarker Urine Collection	Master			X			X		X		X	
DNA Collection[11](optional)	Master			X								
CSF Collection (optional)	Master			X					X[17]			
Concomitant Medication Review	Master	X	X	X		X	X	X	X	X	X	
Concomitant Medication Review	Regimen				X							
Adverse Event Review[9]	Master	X	X	X	X	X	X	X	X	X	X	X
Columbia-Suicide Severity Rating Scale	Master			X		X	X		X		X	
Install Smartphone App[21]	Regimen			X								
Voice Recording[12]	Regimen			X		X	X		X		X	
Uninstall Smartphone App	Master										X	
Assignment to the Regimen	Master	X										
Randomization within the Regimen	Master		X									
Administer/Dispense Investigational product	Regimen			X[13]		X	X		X		X[14]	

Activity	Master Protocol or Regimen-Specific	Master Protocol Screening[1]	Regimen Specific Screening[1]	Baseline	Week 2[2]	Week 4[18,19]	Week 8[18,19]	Week 12	Week 16[18,19]	Week 20	Week 24 or Early Term. Visit[3, 18]	Follow-up Safety Call[3,4]
		Clinic	Clinic	Clinic	Phone	Clinic	Clinic	Phone	Clinic	Phone	Clinic	Phone
		-42 to -1 Days	-41 to 0 Days	Day 0	Day 14 ±3	Day 28 ±7	Day 56 ±7	Day 84 ±3	Day 112 ±7	Day 140 ±3	Day 168 ±7	28±7 days after last dose
Study Drug Accountability/Compliance	**Master**				X[2,22]	X	X	X[22]	X	X[22]	X	
Dose Escalation	**Regimen**			X[15]	X[15]							
Exit Questionnaire	**Master**										X	
Vital Status Determination	**Master**										X[16]	

[1] Master Protocol Screening procedures must be completed within 42 days to 1 day prior to the Baseline Visit. The Regimen-Specific Screening Visit and Baseline Visit should be combined if possible.

[2] At the end of Week 2, an assessment of compliance and tolerance to this dose titration schedule will be conducted. The assessment will be conducted by phone. If tolerability issues are experienced with 300 mg QD or 300 mg BID dosing, the titration schedule may be modified (see Section 5.3.2).

[3] Participants will only have a Follow-Up Safety Call at this time if they *do not* continue into the OLE or if they discontinue prior to Week 24. Participants who continue into OLE will have a Follow-Up Safety Call after their last dose of study drug during the OLE phase.

[4] Participants who continue into the OLE and then early terminate will be asked to complete an Early Termination Visit and Follow-Up Safety Call as described in the body of this RSA.

[5] During the Master Protocol Screening Visit, participants will be consented via the Master Protocol informed consent form (ICF). After a participant is randomized to a regimen, participants will be consented a second time via the RSA ICF.

[6] At the Regimen Specific Screening Visit, participants will have regimen-specific inclusion and exclusion criteria assessed, if applicable.

[7] Vital signs include weight, systolic and diastolic pressure, respiratory rate, heart rate and temperature. Height is measured at Master Protocol Screening Visit only.

[8] Clinical safety labs include hematology (CBC with differential), complete chemistry panel, thyroid function (TSH) and urinalysis. Serum pregnancy testing will occur in women of child-bearing potential at the Master Protocol Screening Visit and as necessary during the study. Pregnancy testing is only repeated as applicable if there is a concern for pregnancy.

[9] Adverse events that occur after signing the master protocol consent form will be recorded.

[10] . For each sample, the time of the last verdiperstat or matching placebo dose prior to sample collection, time of the last meal prior to sampling and time of the PD/PK sample collection should be reported on the CRF.

[11] The DNA sample can be collected after baseline if a baseline sample is not obtained or the sample is not usable.
[12] In addition to study visits outlined in the SOA, participants may be asked to complete twice weekly voice recordings at home. During weeks when a participant is doing a voice recording in-clinic, he or she would only do one other voice recording at home that week.
[13] Administer first dose of investigational product (IP) only after Baseline Visit procedures are completed. Participants should take the first dose of IP while in the office/clinic on the day of the Baseline visit and stay at the clinic for approximately 30 minutes post-dose for observation.
[14] Investigational product will only be dispensed at this visit if the participant continues in the OLE.
[15] From start to end of Week 1, participants will ingest either 300 mg QD of verdiperstat or matching placebo QD. From start to end of Week 2, participants will ingest either 300 mg BID of verdiperstat or matching placebo BID. Starting with Week 3 and continuing to Week 24, participants will ingest either 600 mg BID of verdiperstat or matching placebo BID.
[16] Vital status, defined as a determination of date of death or death equivalent or date last known alive, will be determined for each randomized participant at the end of the placebo-controlled portion of their follow-up (generally the Week 24 Visit, as indicated). If at that time the participant is alive, his or her vital status should be determined again at the time of the last participant's last visit (LPLV) of the placebo-controlled portion of a given regimen. We may also ascertain vital status at later time points by using publicly available data sources as described in section 8.15 of the Master Protocol.
[17] If the CSF collection cannot happen at the Week 16 Visit for logistical reasons such as scheduling, it can happen at the Week 24 Visit.
[18] Participants should be instructed to skip the morning dose of study drug on the day of the study visit. Study drug should not be taken until after study visit procedures are complete.
[19] Visit may be conducted via phone or telemedicine with remote services instead of in-person if this is needed to protect the safety of the participant due to a pandemic or other reason.
[20] If required due to pandemic-related restrictions, Forced Vital Capacity (FVC) performed by a Pulmonary Function Laboratory evaluator or with a study-approved home spirometer, or sustained phonation using a study approved method may be used for eligibility (Master Protocol Screening ONLY).
[21] Two smartphone apps should be installed on the participant's phone, one to collect the voice recordings and one to collect home spirometry.
[22] Drug accountability will not be done at phone visits. A drug compliance check in must be held during phone visits to ensure participant is taking drug per dose regimen and to note any report of missed doses.

SCHEDULE OF ACTIVITIES – REGIMEN-SPECIFIC OPEN-LABEL EXTENSION PHASE (OPTIONAL)

Activity	Open Label Extension (Optional)									
	Week 24 Visit	Week 2[1]	Week 4[11]	Week 8[11]	Week 12	Week 16[10,11]	Week 20	Week 24	Week 28[10,11] and Q12 weeks	Follow-up Safety Call[3,4]
	Clinic	Phone	Clinic	Clinic	Phone	Clinic	Phone	Phone	Clinic	Phone
	Day 0	Day 14 +3	Day 28 +7	Day 56 ±7	Day 84 +3	112 +7 days	140+3 days	168+3 days	Q12 weeks +14 days	28±7 days after last dose
Vital Signs[5]	X		X	X		X			X	
Slow Vital Capacity	X		X	X		X			X	
Home Spirometry			X	X		X			X	
Muscle Strength Assessment	X									
ALSFRS-R	X		X	X	X	X	X	X	X	
ALSAQ-40	X								X[14]	
CNS bulbar Function Scale	X			X		X			X	
12-Lead ECG	X		X						X[13]	
Clinical Safety Labs[6]	X		X	X		X			X	
Biomarker Blood Collection	X					X			X[12]	
Verdiperstat (BHV-3241specific PD biomarkers[7]	X					X			X[12]	
PK Blood Collection[7]	X					X			X[12]	
Biomarker Blood Collection	X					X			X[12]	
Biomarker Urine Collection	X					X			X[12]	
Concomitant Medication Review	X	X	X	X	X	X	X	X	X	
Adverse Event Review	X	X	X	X	X	X	X	X	X	X
Columbia-Suicide Severity Rating Scale	X		X	X		X			X	
Administer/Dispense Investigational product	X[8]		X	X		X			X[15]	
Drug Accountability/Compliance	X	X[16]	X	X	X[16]	X	X[16]	X[16]	X	
Dose Escalation		X[9]								

[1] At the end of Week 2, an assessment of compliance and tolerance to this dose titration schedule will be conducted. The assessment will be conducted by phone. If tolerability issues are experienced with 300 mg QD or 300 mg BID dosing, the titration schedule may be modified (see Section 5.3.2).
[2] The last visit for the open-label extension will be at Week 52. Participants who withdraw consent or terminate early (prior to Week 52) from the study will be asked to be seen for an in-person Early Termination Visit and should have the same procedures as the Week 52 visit.
[3] Participants who continue into the OLE and then early terminate will be asked to complete an Early Termination Visit and Follow-Up Safety Call as described in the body of this RSA.
[4] Participants will have a Follow-up Safety Call approximately 28 days after the last dose of study drug. At this call, information on clinical status should be collected.
[5] Vital signs include weight, systolic and diastolic pressure, respiratory rate, heart rate and temperature.
[6] Clinical safety labs include hematology (CBC with differential), complete chemistry panel, liver function tests, thyroid function (TSH) and urinalysis. Serum pregnancy testing will occur in women of child-bearing potential at the Master Protocol Screening Visit and as necessary during the study. Pregnancy testing is only repeated as applicable if there is a concern for pregnancy.
[7]

[8] Administer first dose of investigational product (IP) only after Week 24 procedures are completed. Participants should take the first dose of IP while in the office/clinic on the day of the Week 24 visit and stay at the clinic for approximately 30 minutes post-dose for observation.
[9] From start to end of Week 1, participants will ingest 300 mg QD of verdiperstat QD. From start to end of Week 2, participants will ingest 300 mg BID of verdiperstat BID. Starting with Week 3 and continuing to Week 52, participants will ingest 600 mg BID of verdiperstat BID.
[10] Participants should be instructed to skip the morning dose of study drug on the day of the study visit – at OLE Weeks 16, 28 and 52. Study drug should not be taken until after study visit procedures are complete.
[11] Visit may be conducted via phone or telemedicine with remote services instead of in-person if this is needed to protect the safety of the participant due to a pandemic.
[12]
[13] 12-Lead ECG is performed at OLE Weeks 4 and 52 only
[14] The ALSAQ-40 is performed at OLE Weeks 28 and 52 only
[15] Investigational product is not administered at the participant's final in-clinic visit at the completion of the OLE.
[16] Drug accountability will not be performed at phone visits. A drug compliance check-in must be held during phone visits to ensure participant is taking drug per dose regimen and to note any report of missed doses.

1 INTRODUCTION REGIMEN B: VERDIPERSTAT (BHV-3241)

1.1 Verdiperstat Background Information

Biohaven Pharmaceuticals, Inc [Biohaven] is developing a new drug, verdiperstat (also known as BHV-3241), for the treatment of neurodegenerative diseases, including amyotrophic lateral sclerosis (ALS).

Verdiperstat is a first-in-class, potent, selective, brain-permeable, irreversible myeloperoxidase (MPO) enzyme. MPO is one of the most abundant enzymes in activated myeloid cells, including microglia [1]. It is a lysosomal enzyme that plays essential roles in immune surveillance and host defense. In disease, innate immune system activation leads to MPO-induced pathological oxidative stress and further inflammation that contribute to cellular injury [1]. Increasing evidence suggests MPO is involved several neurodegenerative diseases [1],[2].

The proposed study is based on cumulative nonclinical, clinical, and neuroimaging studies that implicate MPO activity in the onset and progression of neurodegenerative diseases and suggest treatment with verdiperstat has the potential to slow neurodegeneration. The high unmet need for an effective treatment, together with the available data, provide a compelling rationale for the development of verdiperstat as a treatment for ALS.

Summaries of relevant findings from nonclinical and clinical studies conducted with verdiperstat are provided. Please refer to the Investigator Brochure [3] for additional information.

1.2.1 Dosage Selection and Justification

The dosage of verdiperstat selected for evaluation in this study is 600 mg BID. This dosage was selected based on cumulative experience, including nonclinical toxicology and safety/tolerability, pharmacokinetic, pharmacodynamic, and preliminary efficacy data from phase 1 and phase 2 studies.

Nonclinical toxicology: The program for verdiperstat is comprehensive and supports oral administration in the clinic for chronic treatment. The following studies were included in the toxicology program: single and repeat-dose toxicity in rats and dogs, genotoxicity, reproductive toxicity, phototoxicity, and safety pharmacology. The verdiperstat 600 mg BID dosage is anticipated to produce pharmacokinetic exposures below limits set based on nonclinical toxicology data.

Clinical studies: As of January 2021, approximately 490subjects have received verdiperstat in completed and ongoing studies. In the phase 1 studies (5 completed studies) in healthy subjects, treatment with multiple dosages of up to 900 mg BID was generally safe and well tolerated. In the completed phase 2 studies in participants with Parkinson's disease and Multiple System Atrophy (MSA), treatment at dosages of up to 600 mg BID for 8-12 weeks was generally safe and well tolerated. In the phase 2 studies, the 600 mg BID dosage decreased MPO activity in plasma, providing evidence of peripheral target engagement; reduced TSPO binding on brain PET imaging, providing evidence of central target engagement and proof of mechanism (decreased microglial activation/neuroinflammation); and demonstrated favorable, dose-dependent trends on clinical efficacy measures at 12 weeks in subjects with MSA.

A phase 3 study in patients with MSA is ongoing and currently is still blinded. In this study, approximately 336 subjects have received verdiperstat or placebo. Subjects are randomized 1:1

to receive verdiperstat or placebo, 600 mg BID, for 48 weeks with the option to continue in the study for an additional 48 weeks of open-label treatment with verdiperstat. Please refer to the Investigator's Brochure [3] for additional information.

2 STUDY OBJECTIVES AND ENDPOINTS

Primary Efficacy Objective:
To evaluate the efficacy of verdiperstat as compared to placebo on ALS disease progression.

Secondary Efficacy Objective:

- To test the effect of verdiperstat on selected secondary measures of disease progression, including survival.

Safety Objective:

- To evaluate the safety of verdiperstat for ALS.

Exploratory Efficacy Objective:

- To test the effect of verdiperstat on selected biomarkers and endpoints.
- To explore verdiperstat pharmacokinetics (PK) and pharmacodynamic (PD) effects.

Primary Efficacy Endpoint:
Change in disease severity as measured by the ALS Functional Rating Scale-Revised (ALSFRS-R) total score using a Bayesian repeated measures model that accounts for loss to follow-up due to mortality.

Secondary Efficacy Endpoints:

- Change in respiratory function as assessed by slow vital capacity (SVC).
- Change in muscle strength as measured isometrically using hand-held dynamometry (HHD) and grip strength.
- Survival.

Safety Endpoints:

- Treatment-emergent adverse and serious adverse events.
- Changes in laboratory values and treatment-emergent and clinically significant laboratory abnormalities.
- Changes in ECG parameters and treatment-emergent and clinically significant ECG abnormalities.
- Treatment-emergent suicidal ideation and suicidal behavior.

Exploratory Efficacy Endpoints:

- Changes in quantitative voice characteristics.
- Changes in biofluid biomarkers of neurodegeneration.

- Changes in patient reported outcomes.
- Changes in verdiperstat PK and PD biomarkers.
- Change in respiratory function as assessed by home spirometry.

3 RSA DESIGN

This study is a multicenter, randomized, double-blind, 2-arm placebo-controlled parallel-group study designed to evaluate the efficacy and safety of verdiperstat in a population of participants with ALS. The study is planned to consist of a Screening phase lasting a maximum of approximately 6 weeks (42 days) and a randomized double-blind treatment phase of approximately 24 weeks (see Figure 1). It is anticipated that the Randomization phase will include a dose titration period of approximately 2 weeks followed by a full dosage period of approximately 22 weeks. Participants completing 24 weeks of treatment with verdiperstat or placebo will be eligible to continue to an Open-label Extension Phase (OLE) which will include an additional 52 weeks of dosing. Approximately 160 participants in total are planned to be randomized in a 3:1 ratio to receive either verdiperstat 600 mg BID, or matching placebo BID. The assessments for each visit are outlined in the Schedule of Activities (SoA).

Figure 1. Regimen Specific Appendix Schematic

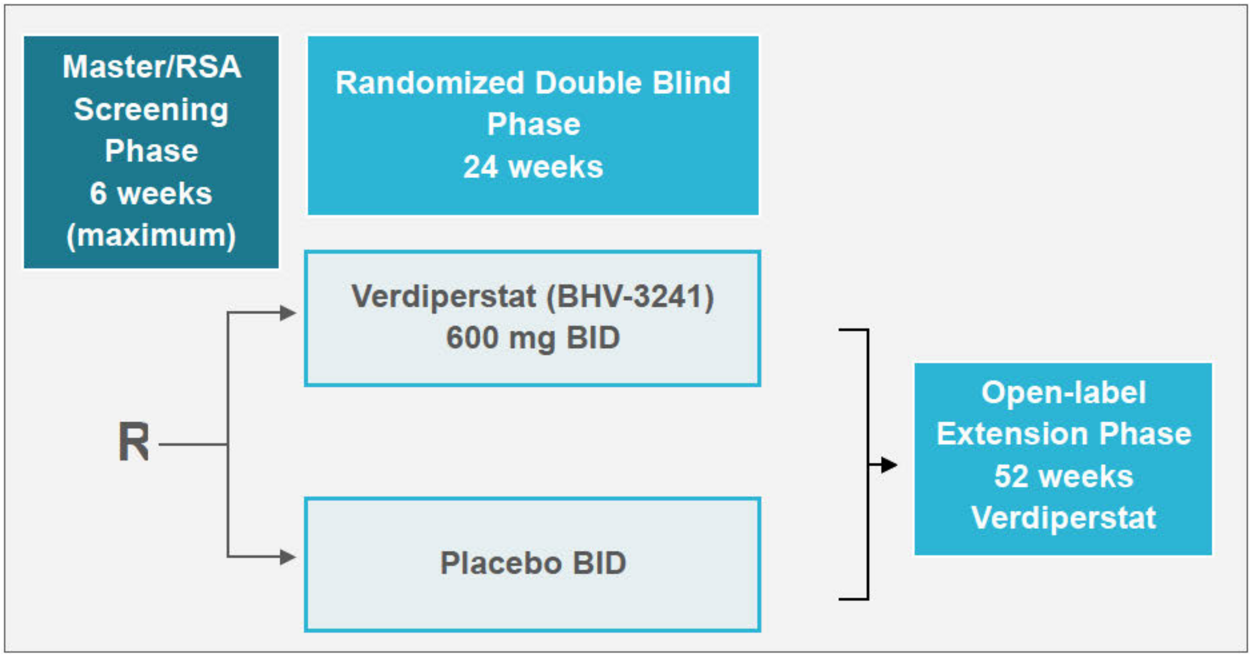

3.1 Scientific Rationale for RSA Design

This RSA is designed to correspond with the design of the Master Protocol and the goals of that study.

3.2 End of Partipation Definition

A participant is considered to have ended his or her participation in the placebo-controlled period of the Regimen if they:

- Complete planned placebo-controlled period visits, as described in the SOA, including participants on or off study drug
- Early terminate from the study and complete the Early Termination Visit and Follow-Up Phone call as described in Section 6.1.11
- Withdraw consent to continue participation in the study, or are lost to follow-up

If a participant initiates open-label study drug in the OLE period, he or she is considered to have completed his or her participation in the OLE period of the Regimen if they choose to discontinue participation or if all planned OLE period visits, including the last visit or the last scheduled procedure shown in the SOA, have been completed.

3.3 End of Regimen Definition

The end of the placebo-controlled period in a Regimen occurs when all randomized participants have completed their participation in the placebo-controlled period as defined in section 3.2.

The end of the OLE period in a Regimen occurs when all participants who initiated open-label study drug in the OLE period have completed their participation in the OLE period as defined in Section 3.2.

4 RSA ENROLLMENT

4.1 Number of Study Participants

Approximately 160 participants will be randomized into this Regimen.

4.2 Additional RSA Inclusion and Exclusion Criteria

To be randomized to an RSA, participants must meet the Master Protocol eligibility criteria. In addition, participants meeting all the following inclusion and exclusion criteria will be allowed to enroll in this Regimen.

4.2.1 RSA Inclusion Criteria

There are no additional RSA Inclusion Criteria from those described in the Master Protocol.

4.2.2 RSA Exclusion Criteria

- Participants who are taking strong inhibitors of CYP1A2 (i.e., ciprofloxacin, enoxacin, fluvoxamine, zafirlukast) for chronic/long-term use defined as more than two weeks.

- Participants who are taking strong inhibitors of CYP3A4 (i.e., conivaptan, itraconazole, ketoconazole, posaconazole, troleandomycin, voriconazole, clarithromycin, diltiazem, idelalisib, nefazodone, and certain antiviral agents [cobicistat, danoprevir, ritonavir, elvitegravir, indinavir, lopinavir, paritaprevir, ombitasavir, dasabuvir, saquinavir, tipranavir, nelfinavir]) for chronic/long-term use defined as more than two weeks. Note: Topical antifungal use is not exclusionary. Participants should not consume large quantities of grapefruit juice (more than 8oz per day) on a regular basis.

There are RSA-specific requirements that apply to Master Protocol exclusion criteria #6. For further details of contraceptive requirements for this RSA, please refer to Section 5.8.2.

*Guidance regarding CYP1A2 and CYP3A4 has been updated in Protocol Section 5.8 Drug-Drug Interactions and Prohibited Medications, however this exclusion criteria has **not** been modified as all participants have been enrolled.*

4.3 Treatment Assignment Procedures

Each participant who meets all eligibility criteria for the RSA will be randomized to receive either verdiperstat or matching placebo for approximately 24 weeks of treatment. Participants who complete the 24-week blinded Treatment Period will be eligible to continue treatment with open-label verdiperstat for an additional 52 weeks.

5 INVESTIGATIONAL PRODUCT

The Investigational Product (IP) should be stored in a secure area according to local regulations. It is the responsibility of the Site Investigator (SI) to ensure that the IP is only dispensed to study participants. The IP must be dispensed only from official study sites by authorized personnel according to local regulations.

In this protocol, the IP is verdiperstat 300 mg extended release (ER) tablets and matching placebo tablets.

5.1 Investigational Product Manufacturer

Biohaven Pharmaceuticals, Inc. is the manufacturer of verdiperstat.

5.2 Labeling, Packaging, and Resupply

5.2.1 Labeling

Container(s) of investigational product will bear a label containing (at a minimum) the name of the study drug, lot and/or batch number and appropriate storage conditions (15°-25°C [59°-77°F] [see USP controlled room temperature] in a tightly closed container, protected from light).

The drug product is presented as verdiperstat Extended Release (ER) Tablets that are reddish-beige, film coated and oval (300 mg strength). The tablets each consist of 300 mg of verdiperstat drug substance as free base, and generally recognized as safe (GRAS) excipients for oral dosage forms including hydroxypropyl methyl cellulose, microcrystalline cellulose, hydroxypropyl cellulose, sodium stearyl fumarate and orange color. Matching placebo tablets contain microcrystalline cellulose and sodium stearyl fumarate and are film-coated with OPADRY® (mixture of hypromellose, polyethylene glycol and titanium dioxide) and iron oxide to give reddish-beige color. The tablets are packed in high-density polyethylene (HDPE) bottles. The bottles are induction sealed and closed with child resistant polypropylene (PP) screw caps.

All investigational products should be kept in a secure area with limited access under appropriate storage conditions: 15°-25°C (59°-77°F) (see USP controlled room temperature) in a tightly closed container, protected from light.

Excursions outside this storage temperature range should be reported as per the Pharmacy Manual Instructions.

5.2.2 Acquisition and Preparation

Initially, after informed consent is obtained at the Screening Visit, the SI or designee will enter the participant into the study and obtain a participant number assignment. After completion of Screening evaluations, all eligible participants will be randomized, in a 3:1 ratio to receive either verdiperstat (600 mg BID) or matching placebo, using an EDC.

5.2.3 *Drug Returns and Destruction*

At each in person visit the steps outlined in Manual of Procedures must be followed for study drug accountability and compliance, as well as study drug return and destruction.

Prior to study drug destruction, all used and unused IP requires a second accountability verification to be completed by a different study team member, and both verifications should be documented on the study destruction logs. No study drug may be destroyed on-site until written approval is provided by the study monitoring team. Sites should follow their local drug destruction policies.

5.3 Study Medication/Intervention, Administration and Duration, and Titration

All participants will be randomized to receive verdiperstat 600 mg BID or matching placebo BID. Participants should take the study drug twice a day; dosing in the morning and evening approximately 12 hours apart.

The tablets should be swallowed whole with a drink of water (or other liquid) or swallowed with substances of other consistencies as appropriate (e.g., apple sauce). The tablets should not be split, chewed, or crushed.

Study medication can be taken without regard to meals.

5.3.1 *Administration and Duration*

<u>Day 1/first dose:</u>

Participants should be administered the Day 1/first dose of study medication (300 mg QD or matching placebo QD) while in the office/clinic on the day of the Regimen-Specific Baseline visit after all visit assessments are complete. Participants should stay at the office/clinic for monitoring for approximately 30 minutes post-dose.

<u>Dose titration period:</u>

From the beginning to the end of Week 1 participants should ingest either 300 mg QD of verdiperstat or matching placebo QD. From the beginning to the end of Week 2, participants should ingest either 300 mg BID of verdiperstat or matching placebo BID.

At the end of Week 2, an assessment of compliance and tolerance to this dose titration schedule will be conducted. The assessment will be conducted by phone. If tolerability issues are experienced with 300 mg QD or 300 mg BID dosing, the titration schedule may be modified (see 5.3.2).

Full dose period:

Starting with the beginning of Week 3 and throughout the remainder of the study, participants should ingest either 600 mg BID of verdiperstat or matching placebo BID.

At the Weeks 4, 8, 16, and 24 study visits, the morning dose of study medication should be held on the day of the study visit and administered in the clinic/office during the study visit, to collect one pre-dose (trough) PK and PD blood sample (see Section 8).

5.3.2 Dosage Modification

Participants will be instructed on the dosing regimen. If tolerability issues are experienced during the dosage titration period with the 300 mg QD and/or 300 mg BID doses (or matching placebo), the titration schedule may be extended (e.g., up titration to the next dosage level delayed by an additional week). Potential tolerability issues may include sudden, clinically significant changes from baseline in symptoms of presyncope, syncope, orthostatic hypotension, or falls that are not otherwise explained. The SI must consult with the Medical Monitor if he or she believes that a change to the dosage titration schedule is warranted; and, the SI must document any such changes to the dosage titration schedule.

During the full dose period of the study, it is anticipated that all participants will receive either verdiperstat 600 mg BID or matching placebo BID. If participants have difficulty tolerating verdiperstat 600 mg BID or matching placebo BID dosing, the SI may permit the participants to switch to verdiperstat 300 mg BID or matching placebo BID dosing (and document this change in the participant's records). Potential tolerability issues may include those listed above as well as clinically significant laboratory abnormalities (i.e., thyroid and renal function) that are not otherwise explained. Down titration to verdiperstat 300 mg BID or matching placebo BID will only be allowed to address tolerability issues and only with Medical Monitor approval. If a participant is down titrated to the 300 mg BID dose, they may be allowed to retry the 600 mg BID dose (re-challenge), if deemed appropriate by the SI and Medical Monitor. Only two re-challenges are allowed. Any such changes must be documented. If a switch to verdiperstat 300 mg BID or matching placebo BID dosing does not result in acceptable tolerability, then dosing should be discontinued.

Dosage modification should be considered if a participant experiences one of the following:

a. Clinically significant changes in frequency and/or severity from baseline in symptoms of presyncope, syncope, orthostatic hypotension, and/or falls that are not explained by disease progression or intercurrent illness and not able to be adequately treated with symptomatic pharmacological and non-pharmacological treatments, based on the judgement of the SI.

b. Development of Grade 3 or Grade 4 hypothyroidism. Re-challenge may be attempted, if warranted, when improved to less than or equal to Grade 2 (inclusive of treatment with thyroxine replacement therapy, see Section 9.1).

c. Development of a greater than or equal to Grade 3 toxicity or an intolerable side effect attributed to verdiperstat based on the judgement of the SI. Re-challenge may be attempted, if warranted, when symptoms improve to less than or equal to Grade 1.

5.4 Drug Holiday

Other dosage reductions or holidays may be determined by the SI who should consult with the Medical Monitor. If a participant experiences an AE for which the SI believes a dose reduction or holiday is warranted, then the SI may temporarily suspend dosing (dose holiday) until such time as he/she feels it is safe for the participant to return to the assigned dose. Any such modifications to the dosage regimen should be noted in the eCRF. If greater than seven days, then a discussion with the Medical Monitor must take place and is required. If a participant is off study drug for more than 14 consecutive days, the participant should re-escalate using the titration paradigm described in section 5.3.1.

5.5 Participant Compliance

Responsible study personnel will dispense the study drug. Participants will be requested to return any unused IP including empty packaging and used bottles at each study visit. Treatment compliance will be assessed at in clinic study visits through bottle counts and will be documented and summarized by a drug-dispensing log for each participant. During phone visits, drug compliance check-in will be held to ensure participant is taking drug per dose regimen and to note any report of missed doses. Participants will be counseled on the importance of taking the study drug as directed at all study visits. If poor compliance continues, (i.e., multiple missed doses resulting in less than 80% overall compliance), discontinuation of the participant from the trial should be considered and discussed with the Medical Monitor.

If the participant loses the ability to swallow while enrolled in Regimen B, the participant will no longer be able to take the study drug as directed. If this occurs the participant will discontinue study drug and will be asked to complete the 6-month study under ITT.

5.6 Justification for Dosage

The dosage of verdiperstat proposed for evaluation in this ALS platform trial regimen is 600 mg BID. This dosage selection was based on cumulative clinical and nonclinical experience with verdiperstat, including nonclinical toxicology, safety and efficacy data in multiple system atrophy and Parkinson’s disease patients. Please refer to the Investigator’s Brochure [3] for additional information.

5.7 Overdose

An overdose is defined as the accidental or intentional administration of any dose of the product that is considered both excessive and medically important. All occurrences of overdose as defined above (suspected or confirmed and irrespective of whether or not it involved

verdiperstat) must be communicated to the Healey Center and Biohaven Pharmaceutical or a specified designee within 24 hours of the SI becoming aware of the updated information and be fully documented as an SAE. An SAE is reported for overdose when the SI feels the overdose was excessive and medically important. Details of any signs or symptoms and their management should be recorded including details of any antidote(s) administered.

5.9 Verdiperstat Known Potential Risks and Benefits

ALS is an adult-onset, fatal neurodegenerative disease. No disease modifying treatment currently exists, only symptomatic and palliative approaches are available. Verdiperstat is an irreversible inhibitor of the MPO enzyme that promotes oxidative stress and neuroinflammation. The high unmet need for an effective treatment for ALS, together with the available preclinical and clinical data with verdiperstat, provide a compelling and favorable overall benefit-risk assessment for the development of verdiperstat at the 600 mg BID dosage as a treatment for ALS. The safety monitoring in the planned clinical study will minimize the potential risks to study participants.

5.9.1 Known Potential Risks

Preclinical and clinical studies have demonstrated an acceptable safety and tolerability profile for verdiperstat but do suggest specific potential risks. The present study includes general and specific safety procedures anticipated to minimize any potential risks.

General procedures will include frequent safety assessments by SIs, thorough evaluations and review of AEs and SAEs on an ongoing basis to monitor for any safety signals or trends by the

Sponsor and Medical Monitor, and Data Safety Monitoring Board review of the benefit-risk of the study for participants.

5.9.1.1 *Thyroid*

In preclinical studies, reversible histopathological changes in the thyroid gland and reversible thyroid hormone changes were observed. In clinical studies, verdiperstat has been associated with laboratory changes indicative of decreased thyroid function. Specifically, there have been increases over time in mean TSH levels and some decreases over time in mean free T4 and mean free T3 levels relative to placebo. Most participants did not have thyroid function test values outside the normal range, and the abnormalities that occurred were mild. Changes in thyroid function tests associated with verdiperstat returned toward baseline levels during the period of observation following discontinuation of verdiperstat.

In aggregate, clinically significant thyroid function abnormalities were rare or infrequent with verdiperstat. The present study will involve monitoring of thyroid function. Thyroid function test abnormalities indicating clinically significant thyroid hormone deficiency are readily treatable with thyroid hormone replacement (see Section 9.1).

5.9.1.2 *Renal*

Reversible renal findings were observed in the 1-month preclinical studies in female rats at high doses, and no renal changes were noted in the 6-month study. In clinical studies, verdiperstat has been associated with decreases in mean uric acid levels over time relative to placebo. A variable proportion of participants receiving verdiperstat have had plasma uric acid levels below the lower limit of normal. Decreases in uric acid levels associated with verdiperstat have tended to return toward baseline following the discontinuation of dosing. Indices of renal function have not shown any abnormalities associated with verdiperstat. The mechanism of the changes in uric acid levels is unclear but could include decreased uric acid production or decreased renal tubular reabsorption. Hypouricemia is thought to be a biochemically defined disorder with no known clinical significance.

In aggregate, clinically significant renal events associated with decreases in uric acid levels have not been observed with verdiperstat. The present study will continue to monitor for indices of renal function.

5.9.1.3 *Cardiovascular*

Increases in heart rate were observed in dogs during the preclinical studies. However, verdiperstat had no effect on blood pressure or on ECG parameters at any dose. Two studies evaluating cardiovascular response to orthostatic tilt in dogs were also performed; no incidences of orthostatic hypotension were observed following administration of verdiperstat.

The first SAD clinical study [15] was discontinued on the basis of AEs that included syncope associated with brief sinus pauses detected on cardiac telemetry. Two of the cases of syncope were associated with orthostatic testing. There was not a relationship between these events and

verdiperstat concentrations. There was no evidence of direct proarrhythmic or other cardiotoxic effects. It was considered that events of syncope may have represented an exaggerated physiological response to study procedures, involving syncope of neurocardiogenic origin. However, an effect of verdiperstat could not be ruled out. A second SAD study [16] was conducted with 2 modifications designed to reduce the risk of syncope: (1) the exclusion of participants with a history of recurrent presyncope and/or syncope in connection with orthostatic challenge, and (2) fractionated dosing. In this second SAD study, there were no episodes of presyncope or syncope, and no clinically relevant findings involving vital signs or ECG parameters. Subsequent clinical studies have used ER formulations and there have been no cases of syncope in participants receiving verdiperstat. There have been some AEs potentially related to syncope (e.g., dizziness, orthostatic hypotension). Overall, there have been no clinically relevant findings involving vital signs or ECGs, with the exception of some decreases in the mean RR interval observed in the verdiperstat 600-mg BID group in the safety/tolerability study conducted in participants with Parkinson's disease [17].

In the clinically concluded Phase 1 study (BHV3241-101), the effect of verdiperstat on ECG parameters was evaluated in 14 healthy volunteers. Preliminary analyses of continuous ECG recordings (Holter monitors) demonstrated that verdiperstat may have had an effect on heart rate, but no clinically relevant effects on the QTcF, PR, or QRS intervals.

In the ongoing, blinded Phase 3 study in subjects with MSA (BHV3241-301) and Phase 2/3 study in subjects with ALS, cardiovascular events have been reported. Independent Data Monitoring Committees are responsible for reviewing unblinded study data from each study on a regular basis in order to safeguard the interests of the enrolled study subjects and for monitoring the benefit-risk profile of the blinded clinical study.

In aggregate, the clinical data do not show clear evidence of significant cardiovascular abnormalities associated with administration of verdiperstat ER formulations. However, an effect of verdiperstat cannot be ruled out, and cardiovascular TEAEs and parameters, including vital signs and ECGs, will continue to be monitored in this study.

5.9.1.4 *Liver*

Minimally non-adverse, increased alanine aminotransferase and slight (adaptive) histopathological effects in the liver were observed in rats during the preclinical studies; these are likely related to enzyme induction (e.g., CYP2B1 induction). In the MAD study [18], several participants, including those receiving verdiperstat and placebo, had increases in hepatic transaminases.

In the ongoing, blinded Phase 3 study in subjects with MSA (BHV3241-301), preliminary data indicate that 4 subjects (1.2%) discontinued the study due to elevations in liver enzymes. Of these, 3 subjects had elevations 5x upper limit of normal. All 4 subjects were asymptomatic, and the elevations in liver enzymes were isolated without increased bilirubin levels. Elevated levels returned to normal in a few weeks.

In aggregate, the clinical data show a possible association of asymptomatic and reversible liver enzyme increases with verdiperstat. The present study will involve monitoring of liver function tests.

5.9.2 Known Potential Benefits

The rationale for the proposed study is based on cumulative preclinical and clinical studies that implicate MPO activity in the onset and progression of neurodegenerative diseases and suggest treatment with verdiperstat has the potential to reduce oxidative stress and neuroinflammation (microglial activation).

6 REGIMEN SCHEDULE

In addition to procedures in the Master Protocol, the following regimen specific procedures will be conducted during the study:

- Home Spirometry
  - Note: Home spirometry should be collected within the visit window but will occur while the participant is not in the clinic (at home or other remote location).
- ALSAQ-40
- CNS Bulbar Function Scale
- Blood samples for PK and PD analyses
- Smartphone installation and removal
- Voice recording

Modifications to Regimen Schedule

Designated visits in the Schedule of Activities (i.e. Week 4, Week 8, and Week 16) may be conducted via telemedicine (or phone if telemedicine is not available) with remote services instead of in-person if needed to protect the safety of the participant due to a pandemic or other reason. If a planned in-clinic visit is conducted via telemedicine (or phone if telemedicine is not available) with remote services, only selected procedures will be performed. Instructions on how to document missed procedures are included in the MOP.

In addition to the procedures in the Master Protocol that should be conducted during the phone or telemedicine and remote visits, the following regimen-specific procedures should be completed:

- Home Spirometry (Week 8 and 16 only)
- Voice Recording
- CNS Bulbar Function Scale (Week 8 and 16 only).

Details on collection of the CNS Bulbar Function Scale, dispensing IP during remote visits, and documenting subjects' willingness to participate in OLE are described in the MOP.

Blood samples for PK and PD analyses and the Week 8 ECG (Week 8 only) are **<u>not</u>** collected during the remote visits by the home health agency and this should be recorded as such in the applicable source documentation and EDC.

6.1 Placebo-Controlled Period

6.1.1 Verdiperstat Regimen Specific Screening Visit

This visit will take place in-person after the Master Protocol randomization to a regimen. There are no additional procedures specific for the verdiperstat RSA other than what is being done at the Master Protocol.

Participants may be required to reconsent to the regimen if new procedures or information is added in the future. Should a participant need to reconsent, this should occur during the participant's next in-person visit. If the participant's next in-clinic visit is conducted remotely, reconsent may also be completed remotely using the following procedures:

1. The site staff sends copy of the informed consent form to the participant.
2. The participant reads through the consent form but does not sign.
3. The Site Investigator, or other study staff member approved and delegated to obtain informed consent, contacts the participant and reviews the informed consent form with the participant.
4. The participant signs the informed consent form and returns the original signed consent form back to the site.
5. Once received at the site, the individual who consented the participant signs the informed consent form.

6.1.2 Baseline Visit

This visit will take place in-person after the Regimen-Specific Screening Visit. The following procedures will be performed for the regimen schedule:

- ALSAQ-40
- CNS Bulbar Function Scale
- Collection of plasma samples for verdiperstat PD biomarkers (MPO protein and activity) and a plasma PK sample
- Install Smartphone App
- Home Spirometry
- Voice Recording
- Dispense IP
- Remind participant to bring IP to the next visit

After all Baseline procedures are completed, administer a single 300 mg tablet of verdiperstat or placebo. Participants should stay at the clinic for approximately 30 minutes for observation.

Participants should be instructed:

- To ingest either 300 mg QD of BHV-3241 or matching placebo QD for the rest of Week 1.
- To ingest either 300 mg BID of BHV-3241 or matching placebo BID starting at the beginning of Week 2.
- Participants should take the study drug twice a day; dosing in the morning and evening approximately 12 hours apart.

6.1.3 Week 2 Telephone Visit

This visit (via telephone) will take place 14 ± 3 days after the baseline Visit. The following procedures will be performed for the regimen schedule:

- Collection of concomitant medication information.
- An assessment of compliance (i.e. whether participant has stopped taking study drug) and tolerance to this dose titration schedule.
  - If tolerability issues are experienced with 300 mg QD or 300 mg BID dosing, the titration schedule may be modified (see Section 5.3.2).
- Participants should be instructed:
  - Starting at the beginning of Week 3, to ingest either 600 mg BID of verdiperstat or matching placebo BID and continue taking this dosage through the remainder of the 24-week Treatment Period.

6.1.4 Week 4 Visit

Participants should be instructed to skip the morning dose of study drug on the day of the study visit. Study drug should not be taken until after study visit procedures are complete.

This visit will take place in-person 28 ± 7 days after the Baseline Visit. The following procedures will be performed for the regimen schedule:

- Collection of plasma samples for verdiperstat PD biomarkers (MPO protein and activity) and a plasma PK sample
- Voice Recording
- Dispense IP
- Remind participant to bring study drug to the next visit

6.1.5 Week 8 Visit

Participants should be instructed to skip the morning dose of study drug on the day of the study visit. Study drug should not be taken until after study visit procedures are complete.

This visit will take place in-person 56 ± 7 days after the Baseline Visit. The following procedures will be performed for the regimen schedule:

- Home Spirometry
- CNS Bulbar Function Scale
- 12-Lead ECG
- Collection of plasma samples for verdiperstat PD biomarkers (MPO protein and activity) and a plasma PK sample
- Voice recording
- Dispense IP
- Remind participant to bring IP to the next visit

6.1.6 Week 12 Telephone Visit

This visit will take place 84 ± 3 days after the Baseline Visit via telephone.

6.1.7 Week 16 Visit

Participants should be instructed to skip the morning dose of study drug on the day of the study visit. Study drug should not be taken until after study visit procedures are complete.

This visit will take place in-person 112 ± 7 days after the Baseline Visit. The following procedures will be performed:

- Document participant's willingness to participate in the OLE
    - If OLE consent is not obtained at Week 16, it may be obtained at Week 24.
- Home Spirometry
- CNS Bulbar Function Scale
- Collection of plasma samples for verdiperstat PD biomarkers (MPO protein and activity) and a plasma PK sample
- Voice Recording
- Dispense IP
- Remind participant to bring IP to the next visit

6.1.8 Week 20 Telephone Visit

This visit will take place 140 ± 3 days after the Baseline Visit via telephone.

6.1.9 Week 24 Visit or Early Termination Visit

Participants should be instructed to skip the morning dose of study drug on the day of the study visit. Study drug should not be taken until after study visit procedures are complete.

The Week 24 Visit will take place in-person 168 ± 7 days after the Baseline Visit. The following procedures will be performed at either the Week 24 Visit or the Early Termination Visit for the regimen schedule:

- Home Spirometry
- ALSAQ-40
- CNS Bulbar Function Scale
- Collection of plasma samples for verdiperstat PD biomarkers (MPO protein and activity) and a plasma PK sample
- Voice Recording
- Uninstall Smartphone App
- Dispense IP (only if continuing in OLE)
- Remind participant to bring IP to the next visit

For participants rolling into the OLE, all participants should be instructed to follow the dose escalation scheme.

- After all Week 24 procedures are completed, administer a single 300 mg tablet of verdiperstat. Participants should stay at the clinic for approximately 30 minutes for observation.
- Dispense study drug to last for the next 4 weeks.
- Participants should be instructed:
    - To ingest 300 mg QD of BHV-3241 for the rest of Week 1.
    - To ingest 300 mg BID of BHV-3241 starting at the beginning of Week 2. Participants should take the study drug twice a day; dosing in the morning and evening approximately 12 hours apart.
        - If tolerability issues are experienced with 300 mg QD or 300 mg BID dosing, the titration schedule may be modified (see Section 5.3.2).
    - To ingest 600 mg BID of BHV-3241 starting at the beginning of Week 3 and through the remainder of the 52-week OLE. Participants should take the study drug twice a day; dosing in the morning and evening approximately 12 hours apart.

For participants NOT rolling into the OLE:

A Follow-up Safety Call should be scheduled approximately 28 days after last dose of study drug to collect clinical status information.

6.1.10 Follow-Up Safety Call

Participants will have a Follow-Up Safety Call 28+7 days after their last dose of study drug. Only those participants NOT continuing on in the Open Label Extension will have the Follow-Up Safety Call following the end of their participation in the placebo-controlled portion of the trial. The following procedures will be performed:

- Assess and document AEs, including Key Study Events (*see section 10.3 of Master Protocol*)

6.1.11 Process for Early Terminations

Participants who early terminate from the study and do not complete the protocol per ITT will be asked to be seen for an in-person Early Termination Visit and complete a Follow-Up Safety Call. If a participant is not able to be seen in-person, safety assessments and others that can be conducted remotely should be performed.

The in-person Early Termination Visit should be scheduled as soon as possible after a participant early terminates. If the participant early terminates during the placebo-controlled portion of the Regimen, all assessments that are collected at the Week 24 in-clinic visit should be conducted. The Follow-Up Safety Call should be completed approximately 28±7 days after the last dose of study drug. If the participant terminates early during the OLE period, all assessments that are intended for collection at the OLE Week 24 in-clinic visit should be conducted. The Follow-Up Safety Call should be completed approximately 28±7 days after the last dose of IP.

If the Early Termination Visit occurs approximately 28±7 days after the last dose of study drug, the information from the Follow-Up Safety Call can be collected during the Early Termination Visit, and a separate Follow-Up Safety Call does not need to be completed. If the in-person Early Termination Visit does not occur within 28±7 days of the last dose of study drug, the Follow-Up Safety Call should occur approximately 28±7 days after the last dose of study drug and the Early Termination Visit will be completed after the Follow-Up Safety Call.

6.1.12 Criteria for Participant Termination

Participants MUST discontinue the IP (and non-investigational product at the discretion of the SI) for any of the following reasons:

- Withdrawal of informed consent (participant's decision to withdraw for any reason)
- Any clinical adverse event (AE), laboratory abnormality or intercurrent illness which, in the opinion of the SI or sponsor, indicates that continued participation in the study is not in the best interest of the participant

- Disease progression, which, in the opinion of the SI or sponsor, indicates that continued participation in the study is not in the best interest of the participant
- Pregnancy
- Termination of the study by the Sponsor
- Loss of ability to freely provide consent through imprisonment or involuntary incarceration for treatment of either a psychiatric or physical (e.g., infectious disease) illness

All participants who discontinue study treatment earlier than Week 24 should comply with protocol specified Early Termination procedures as appropriate, outlined in the Schedule of Activities.

An exception to the requirement for End of Treatment procedures is when a participant withdraws consent for all study procedures or loses the ability to consent freely (i.e., is imprisoned or involuntarily incarcerated for the treatment of either a psychiatric or physical illness).

6.2 Open Label Extension (OLE)

Participants who have completed the placebo-controlled portion of the trial on drug, will be eligible to continue in the Open Label Extension. The OLE of the study will continue until verdiperstat is approved and available in the United States, or Biohaven Pharmaceuticals terminates development of verdiperstat for ALS.

Modifications to OLE Schedule

Designated visits in the Schedule of Activities for the OLE (i.e. Week 4, Week 8, Week 16, Week 28, and Week 40) may be conducted via telemedicine (or phone if telemedicine is not available) with remote services instead of in-person if needed to protect the safety of the participant due to a pandemic or other reason. If a planned in-clinic visit is conducted via telemedicine (or phone if telemedicine is not available) with remote services, only selected procedures will be performed. Instructions on how to document missed procedures are included in the Manaul of Procedures.

In addition to the procedures in the Master Protocol that should be conducted during the phone or telemedicine and remote visits, the following regimen-specific procedures should be completed:

- Home Spirometry
- CNS Bulbar Function Scale (Week 8 and 16 only).

Blood samples for Week 16 PK and PD analysis and the Week 4 and 28 ECGs are **not** collected during the remote visits by the home health agency and this should be recorded as such in the applicable source documentation and EDC.

6.2.1 Week 2 Telephone Visit - OLE

This visit (via telephone) will take place 14 ± 3 days after the Week 24 visit. The procedures listed in the SoA should be performed including:

- Assess and document AEs and concomitant medications, including Key Study Events (see section 10.3 of Master Protocol)
- Assess compliance and tolerance to this dose titration schedule
  - If tolerability issues are experienced with 300 mg QD or 300 mg BID dosing, the titration schedule may be modified (see Section 5.3.2).
- Instruct participants on appropriate dosing
  - Starting at the beginning of Week 3, to ingest 600 mg BID of verdiperstat and continue taking this dose through the remainder of the 52-week OLE
- Remind participant to bring in IP to the next visit

6.2.2 Week 4 Visit – OLE

This visit will take place in-person 28 ± 10 days after the Week 24 Visit of the placebo-controlled portion of the trial. The procedures listed in the SoA should be performed and participants should be provided with 4 weeks of drug and instructions for dosing.

6.2.3 Week 8 Visit - OLE

This visit will take place in-person 56 ± 7 days after the Week 24 Visit of the placebo-controlled portion of the trial. The procedures listed in the SoA should be performed and participants should be provided with 8 weeks of drug and instructions for dosing.

6.2.4 Week 12 Telephone Visit – OLE

This visit will take place 84 ± 3 days after the Week 24 Visit of the placebo-controlled portion of the trial via telephone. The procedures listed in the SoA should be performed.

6.2.5 Week 16 Visit – OLE

Participants should be instructed to skip the morning dose of study drug on the day of the study visit. Study drug should not be taken until after study visit procedures are complete.

This visit will take place in-person 112 ± 7 days after the Week 24 Visit of the placebo-controlled portion of the trial. The procedures listed in the SoA should be performed and participants should be provided with 12 weeks of drug and instructions for dosing.

6.2.6 Week 20 Telephone Visit – OLE

This visit will take place in-person 140 ± 3 days after the Week 24 Visit of the placebo-controlled portion of the trial. The procedures listed in the SoA should be performed.

6.2.7 Week 24 Telephone Visit – OLE

This visit will take place 196 ± 3 days after the Week 24 Visit of the placebo-controlled portion of the trial via telephone. The procedures listed in the SoA should be performed.

6.2.8 Week 28 Visit and Q12 Weeks – OLE

Participants should be instructed to skip the morning dose of study drug on the day of the study visit. Study drug should not be taken until after study visit procedures are complete.

This visit will take place in-person 196 ± 14 days after the Week 24 Visit of the placebo-controlled portion of the trial. The procedures listed in the SoA should be performed and participants should be provided with 8 weeks of drug and instructions for dosing. Following the Week 28 OLE Visit, visit will occur every 12 weeks ± 14 days.

7 OUTCOME MEASURES AND ASSESSMENTS

For all assessments listed below, please refer to the Manual of Procedures for detailed instructions.

7.1 Voice Analysis

Voice samples will be collected twice per week, using an app installed on either an android or iOS based smartphone. The app characterizes ambient noise, then asks participants to perform a set of speaking tasks: reading sentences -- 5 fixed and 5 chosen at random from a large sentence bank-- repeating a consonant-vowel sequence, producing a sustained phonation, and counting on a single breath. Voice signals are uploaded to a HIPAA-compliant web server, where an AI-based analysis identifies relevant vocal attributes. Quality control (QC) of individual samples will occur by evaluation of voice records by trained personnel.

The voice analysis app is only available in English, therefore participants who do not speak English should not complete the voice recording. Caregivers cannot provide language assistance when the participant is completing the voice recording.

7.2 ALSAQ-40

The Amyotrophic Lateral Sclerosis Assessment Questionnaire-40 (ALSAQ-40) is a patient self-report health status patient-reported outcome. The ALSAQ-40 consists of forty questions that are specifically used to measure the subjective well-being of patients with ALS and motor neuron disease.

Participants will be handed the questionnaire and asked to write their answers themselves. Caregivers can also help, if needed.

7.3 Center for Neurologic Study Bulbar Function Scale

The Center for Neurologic Study Bulbar Function Scale (CNS-BFS) is a patient self-report scale that has been developed for use as an endpoint in clinical trials and as a clinical measure for evaluating and following ALS patients. The CNS-BFS consists of three domains (swallowing, speech, and salivation), which are assessed with a 21-question, self-report questionnaire.

Participants will be handed the questionnaire and asked to write their answers themselves. Caregivers can also help, if needed.

Instructions on administering the questionnaire during a phone or telemedicine visit will be included in the MOP.

7.4 Home Spirometry

Remote/home-based forced vital capacity will be measured with the MIR Spirobank Smart spirometer. Instructions for use will be provided to the participant. The participant will perform the vital capacity maneuver at home with real time video coaching (or phone coaching, if video is not available) by the evaluator. Three to five vital capacity maneuvers will be performed, consistent with the manner vital capacity is obtained in clinic.

9 SAFETY AND ADVERSE EVENTS

9.1 Adverse Events of Special Interest – Thyroid Function

Reversible changes in thyroid function tests were observed during the preclinical studies and clinical studies with verdiperstat. Based on prior trials, a small percentage of participants may be expected to develop mild (subclinical) hypothyroidism generally with serum TSH < 10 mIU/L. Laboratory monitoring of thyroid function will be performed and documented throughout this study. Site Investigators should review TSH levels and note the trend over time. The Central Laboratory will flag any TSH $\geq$ ULN, and T3 and free T4 analysis should be performed.

Any significant abnormal findings should be discussed with the study Medical Monitor and may be followed up as per local practice (e.g., investigations and consultation with endocrinologist). In cases of suspected hypothyroidism, repeat thyroid function tests should be measured and levothyroxine replacement therapy should be considered. Initiation of treatment is at the discretion of the SI.

Potential initiation of levothyroxine (T4) replacement therapy may be considered according to the following guidelines:

- For a serum TSH between 5 and 10, levothyroxine 0.05 mg daily (50 mcg) is a reasonable dose to prescribe.
- For a serum TSH between 10 and 20, levothyroxine 0.075 mg daily (75 mcg) is a reasonable dose to prescribe.
- For a serum TSH greater than 20, many physicians would feel more comfortable having an endocrinologist treat such participants.

If signs/symptoms of hypothyroidism (i.e. adverse event, initiation of levothyroxine) and/or significant changes in thyroid function develop, collection of follow up clinical chemistry samples and/or unscheduled study visits may be warranted. It is recommended to wait for 5 or 6 half-lives (5 – 6 weeks) to retest thyroid function. Serum T3, free T4 and TSH should be monitored in such participants, and the serum TSH assesses whether therapy is adequate.

If there are clinically significant issues with tolerability that are not able to be treated adequately with levothyroxine replacement therapy, the SI may consider modifications to the dosage regimen. Consultation with the Medical Monitor is required. Modifications may include reducing the dose of verdiperstat /matching placebo to 300 mg BID or taking a dose holiday. Any such modifications to the dosage regimen should be noted in the CRF.

10 REGIMEN-SPECIFIC STATISTICAL CONSIDERATIONS

10.1 Deviations from the Default Master Protocol Trial Design

The statistical design for this regimen will be in accordance with the default statistical design described in Appendix I of the master protocol with only one deviation. This regimen will not include interim analyses for early success. As such, unless this regimen is stopped early for futility, it will enroll to the maximum sample size. At the final analysis, the treatment will be considered success relative to the shared control group if the posterior probability of superiority is greater than 98.0%. This value was selected by simulation to control the overall one-sided Type I error rate across the null scenarios to less than 2.5%. Results are based on 5000 simulations per scenario.

10.2 Regimen Specific Operating Characteristics

Clinical trial simulation is used to quantify operating characteristics for this regimen given the difference from the default design. The simulation of virtual participants and the simulation scenarios are as described in Appendix I to the master protocol. We present here the operating characteristics for the regimen, according to the default design but with no opportunity to stop early for success. Futility is considered non-binding and as such, Table 10.2.1 shows null hypothesis scenarios and resulting Type I error with no futility stopping. We show null and alternative hypothesis scenarios inclusive of futility stopping in Tables 10.2.2 and 10.2.3. Table 10.2.2 shows operating characteristics under the base case assumptions and Table 10.2.3 shows operating characteristics for the various sensitivity scenarios.

This regimen will be one of the first three regimens to be enrolled in the platform and so there are no participants in the shared control from regimens already complete. The comparison to control will rely entirely upon concurrently randomized controls. Concurrently randomized controls will be defined according to Appendix I to the master protocol.

Table 10.2.1: Null Hypothesis Scenarios with No Early Futility Stopping

Sensitivity Scenario	Mean Duration (Months)	Mean N	Prob. Early Success	Prob. Total Success	Prob. Early Futility	Mean DRR
Null Treatment Effect (0% Slowing; Mort HR = 1.0)						
Base	15	160	0.000	0.020	0.000	1.007
Slower Accrual	27	160	0.000	0.023	0.000	1.007
Faster Accrual	11	160	0.000	0.023	0.000	1.008
Less Dropout	15	160	0.000	0.021	0.000	1.006
More Dropout	15	160	0.000	0.023	0.000	1.007
Reg. Start Same Time	18	160	0.000	0.025	0.000	1.005

Table 10.2.1: Null Hypothesis Scenarios with No Early Futility Stopping						
**Sensitivity Scenario**	**Mean Duration (Months)**	**Mean N**	**Prob. Early Success**	**Prob. Total Success**	**Prob. Early Futility**	**Mean DRR**
Reg. Start 3 Months Apart	11	160	0.000	0.025	0.000	1.017
ALSFRS-R Slower Less Var. Progress	15	160	0.000	0.022	0.000	1.006
ALSFRS-R Faster More Var. Progress	15	160	0.000	0.022	0.000	1.007
ALSFRS-R Lower Resid. Error	15	160	0.000	0.024	0.000	1.006
ALSFRS-R Higher Resid. Error	15	160	0.000	0.022	0.000	1.007
10% Mortality Rate	15	160	0.000	0.025	0.000	1.005
20% Mortality Rate	15	160	0.000	0.023	0.000	1.006

Table 10.2.2: Base Scenarios Operating Characteristics. Null and Alternative Hypotheses With Futility Stopping							
**Scenario (% Slowing ALSFRS-R)**	**HR Mort.**	**Mean Duration (Months)**	**Mean N**	**Prob. Early Success**	**Prob. Total Success**	**Prob. Early Futility**	**Mean DRR**
**0%**	**1**	14	155	0.000	0.020	0.290	1.049
**25%**	**.75**	15	160	0.000	0.615	0.008	0.747
**30%**	**.7**	15	160	0.000	0.776	0.002	0.694
**35%**	**.65**	15	160	0.000	0.888	0.001	0.642
**30%**	**1**	15	160	0.000	0.731	0.003	0.708
**30%**	**1.3**	15	160	0.000	0.683	0.004	0.722

Table 10.2.3: Base and Sensitivity Scenarios with Early Futility Stopping.						
**Sensitivity Scenario**	**Mean Duration (Months)**	**Mean N**	**Prob. Early Success**	**Prob. Total Success**	**Prob. Early Futility**	**Mean DRR**
Null Treatment Effect (0% Slowing; Mort HR = 1.0)						
Base	14	155	0.000	0.020	0.290	1.049
Slower Accrual	23	143	0.000	0.023	0.347	1.063
Faster Accrual	11	160	0.000	0.023	0.257	1.034
Less Dropout	14	155	0.000	0.021	0.280	1.047
More Dropout	14	155	0.000	0.023	0.271	1.047
Reg. Start Same Time	16	154	0.000	0.025	0.281	1.035
Reg. Start 3 Months Apart	10	160	0.000	0.025	0.219	1.043
ALSFRS-R Slower Less Var. Progress	14	155	0.000	0.022	0.283	1.049
ALSFRS-R Faster More Var. Progress	14	155	0.000	0.022	0.300	1.047
ALSFRS-R Lower Resid. Error	14	155	0.000	0.024	0.295	1.046
ALSFRS-R Higher Resid. Error	14	155	0.000	0.022	0.284	1.050
10% Mortality Rate	14	155	0.000	0.025	0.286	1.046
20% Mortality Rate	14	155	0.000	0.023	0.294	1.046
Alternative Common Treatment Effect (30% Slowing; Mort HR = .70)						
Base	15	160	0.000	0.776	0.002	0.694
Slower Accrual	27	160	0.000	0.771	0.005	0.695
Faster Accrual	11	160	0.000	0.762	0.002	0.694
Less Dropout	15	160	0.000	0.745	0.003	0.694
More Dropout	15	160	0.000	0.722	0.003	0.694
Reg. Start Same Time	18	160	0.000	0.782	0.002	0.691

Table 10.2.3: Base and Sensitivity Scenarios with Early Futility Stopping.						
**Sensitivity Scenario**	**Mean Duration (Months)**	**Mean N**	**Prob. Early Success**	**Prob. Total Success**	**Prob. Early Futility**	**Mean DRR**
Reg. Start 3 Months Apart	11	160	0.000	0.681	0.003	0.700
ALSFRS-R Slower Progress	15	160	0.000	0.754	0.003	0.693
ALSFRS-R Faster Progress	15	160	0.000	0.791	0.002	0.694
ALSFRS-R Lower Resid. Error	15	160	0.000	0.793	0.003	0.692
ALSFRS-R Higher Resid. Error	15	160	0.000	0.758	0.003	0.694
10% Mortality Rate	15	160	0.000	0.782	0.003	0.693
20% Mortality Rate	15	160	0.000	0.799	0.003	0.695
Alternative Treatment Effect No Mort. Benefit (30% Slowing; Mort HR = 1.0)						
Base (5% Mort. Rate)	15	160	0.000	0.731	0.003	0.708
10% Mort. Rate	15	160	0.000	0.695	0.004	0.721
20% Mort. Rate	15	160	0.000	0.602	0.007	0.749
Alternative Treatment Effect Worse Mort. (30% Slowing; Mort HR = 1.3)						
Base (5% Mort. Rate)	15	160	0.000	0.683	0.004	0.722
10% Mort. Rate	15	160	0.000	0.592	0.008	0.750
20% Mort. Rate	15	160	0.000	0.411	0.015	0.803

10.3 Sharing of Controls from Other Regimens

The primary analysis of this regimen will include sharing of all controls from the other regimens. This is justified by the minor differences in inclusion/exclusion criteria of the RSA, such that there are no expected systematic differences in the primary endpoint between the controls across regimens.

11 REFERENCES

1. Lefkowitz, D.L., and Lefkowitz, S.S. (2008). Microglia and myeloperoxidase: a deadly partnership in neurodegenerative disease. Free Radic Biol Med *45*, 726-731.
2. Pravalika, K., Sarmah, D., Kaur, H., Wanve, M., Saraf, J., Kalia, K., Borah, A., Yavagal, D.R., Dave, K.R., and Bhattacharya, P. (2018). Myeloperoxidase and Neurological Disorder: A Crosstalk. ACS Chem Neurosci *9*, 421-430.
3. Biohaven Pharmaeuticals Inc. Investigator Brochure Verdiperstat, Edition 3.0, March 2021.
4. Singh, A., Kukreti, R., Saso, L., and Kukreti, S. (2019). Oxidative Stress: A Key Modulator in Neurodegenerative Diseases. Molecules *24*.
5. Casas, A.I., Dao, V.T., Daiber, A., Maghzal, G.J., Di Lisa, F., Kaludercic, N., Leach, S., Cuadrado, A., Jaquet, V., Seredenina, T.*, et al.* (2015). Reactive Oxygen-Related Diseases: Therapeutic Targets and Emerging Clinical Indications. Antioxidants & redox signaling *23*, 1171-1185.
6. van der Vliet, A., Eiserich, J.P., Halliwell, B., and Cross, C.E. (1997). Formation of reactive nitrogen species during peroxidase-catalyzed oxidation of nitrite. A potential additional mechanism of nitric oxide-dependent toxicity. The Journal of biological chemistry *272*, 7617-7625.
7. Seredenina, T., Nayernia, Z., Sorce, S., Maghzal, G.J., Filippova, A., Ling, S.C., Basset, O., Plastre, O., Daali, Y., Rushing, E.J.*, et al.* (2016). Evaluation of NADPH oxidases as drug targets in a mouse model of familial amyotrophic lateral sclerosis. Free Radic Biol Med *97*, 95-108.
8. Beers, D.R., and Appel, S.H. (2019). Immune dysregulation in amyotrophic lateral sclerosis: mechanisms and emerging therapies. Lancet Neurol *18*, 211-220.
9. Hammond, T.R., Marsh, S.E., and Stevens, B. (2019). Immune Signaling in Neurodegeneration. Immunity *50*, 955-9.
10. Chew, S., and Atassi, N. (2019). Positron Emission Tomography Molecular Imaging Biomarkers for Amyotrophic Lateral Sclerosis. Front Neurol *10*, 135.
11. BHV3241-103. BHV3500-103. A Phase1, Open-Label, Drug Interaction Study to Evaluate the Effect of Fluvoxamine on the Pharmacokineticsof Single-Dose VerdiperstatAdministeredas anExtended Release Tablet in Healthy Adult Subjects: Biohaven Pharmaceuticals, Inc.; in reporting.
12. BHV3241-107. A Phase1, Open-Label,Drug Interaction Study to Evaluate the Effect of Itraconazoleon the Pharmacokineticsof Single-Dose Verdiperstat Administered as an Extended Release Tablet in Healthy Adult Subjects: Biohaven Pharmaceuticals, Inc.; in reporting.
13. BHV3241-106. A Phase 1, Open-Label, Drug Interaction Study to Evaluate the Effect of Multiple-Dose Verdiperstat Administeredas an Extended Release Tablet on the Pharmacokinetics of Midazolam, a Sensitive CYP3A4 Substrate, in Healthy Adult Subjects: Biohaven Pharmaceuticals, Inc.; in reporting.
14. Study 3210-1088-1800. AZD3241: In Vitro Assessment of the Induction Potential of AZD3241 in Primary Human Hepatocyte Cultures: AstraZeneca; 2009.
15. D0490C00001. A First Time in Man, Single-Centre, Placebo Controlled, Randomized, Double-blind (within dose panel) Study in Healthy Volunteers to Evaluate Safety,

Tolerability and Pharmacokinetics after Oral Single Ascending Doses of AZD3241: AstraZeneca; 2007.
16. D0490C00012. A Single-Centre, Placebo-Controlled, Randomised, Double-Blind Study to Evaluate the Safety, Tolerability and Pharmacokinetics of AZD3241 following Administration of Single Ascending (Part A) and Fractionated Ascending (Part B) Oral Doses to Young Healthy Volunteers: AstraZeneca; 2009.
17. D0490C00005. A Phase IIa, 12 Week, Multicentre, Double-Blind, Randomized, Placebo Controlled, Parallel-Group Study to Assess the Safety and Tolerability of Oral AZD3241 in Patients with Parkinson's Disease: AstraZeneca; 2014.
18. D0490C00002. A Phase I, Single Centre, Double-blind, Randomised, Placebo-controlled, Parallel-group Study to Assess the Safety, Tolerability and Pharmacokinetics of Extended Release Tablets of AZD3241 after Administration of Multiple Ascending Doses in Healthy Male and Female Volunteers including the Effect of Food: AstraZeneca; 2010.

Appendix I: The Bulbar Function Scale (CNS-BFS)

BULBAR FUNCTION SCALE (CNS-BFS)						
**SIALORRHEA**	**Does Not Apply (1)**	**Applies Rarely (2)**	**Applies Occasionally (3)**	**Applies Frequently (4)**	**Applies Most of the Time (5)**	
1. Excessive saliva is a concern to me.	❍	❍	❍	❍	❍	
2. I take medication to control drooling.	❍	❍	❍	❍	❍	
3. Saliva causes me to gag or choke.	❍	❍	❍	❍	❍	
4. Drooling causes me to be frustrated or embarrassed.	❍	❍	❍	❍	❍	
5. In the morning I notice saliva on my pillow.	❍	❍	❍	❍	❍	
6. My mouth needs to be dabbed to prevent drooling.	❍	❍	❍	❍	❍	
7. My secretions are not manageable.	❍	❍	❍	❍	❍	
				**TOTAL Sialorrhea Score: _____**		
**SPEECH**	**Does Not Apply (1)**	**Applies Rarely (2)**	**Applies Occasionally (3)**	**Applies Frequently (4)**	**Applies Most of the Time (5)**	**Unable to Communicate by Speaking (6)**
1. My speech is difficult to understand.	❍	❍	❍	❍	❍	❍

2. To be understood I repeat myself.	❍	❍	❍	❍	❍	❍
3. People who understand me tell other people what I said.	❍	❍	❍	❍	❍	❍
4. To communicate I write things down or use devices such as a computer.	❍	❍	❍	❍	❍	❍
5. I am talking less because it takes so much effort to speak.	❍	❍	❍	❍	❍	❍
6. My speech is slower than usual.	❍	❍	❍	❍	❍	❍
7. It is hard for people to hear me.	❍	❍	❍	❍	❍	❍
				**TOTAL Speech Score: _____**		

**SWALLOWING**	**Does Not Apply (1)**	**Applies Rarely (2)**	**Applies Occasionally (3)**	**Applies Frequently (4)**	**Applies Most of the Time (5)**	
☐ Feeding tube is in place						
1. Swallowing is a problem.	❍	❍	❍	❍	❍	
2. Cutting my food makes it easier to chew and swallow.	❍	❍	❍	❍	❍	
3. To get food down I have switched to a soft diet.	❍	❍	❍	❍	❍	
4. After swallowing I gag or choke.	❍	❍	❍	❍	❍	

5. It takes longer to eat.	❍	❍	❍	❍	❍	
6. My weight is dropping because I can't eat normally.	❍	❍	❍	❍	❍	
7. Food gets stuck in my throat.	❍	❍	❍	❍	❍	
				**TOTAL Swallowing Score: _____**		
				**OVERALL SCORE: _____**		

Appendix II: The ALSAQ-40

ALSAQ-40

Please complete this questionnaire as soon as possible. If you have any difficulties filling in this questionnaire by yourself, please have someone help you. However it is **your** responses that we are interested in.

The questionnaire consists of a number of statements about difficulties that you may have experienced **during the last 2 weeks.** There are no right or wrong answers: your first response is likely to be the most accurate for you. **Please check the box that best describes your own experiences or feelings.**

Please answer every question even though some may seem very similar to others, or may not seem relevant to you.

All the information you provide is **confidential.**

The following statements all refer to difficulties that you may have had **during the last 2 weeks**.<br>Please indicate, by checking the appropriate box, how often the following statements have been true for you.

ALSAQ-40 © Oxford University Innovation Limited, 2000. All rights reserved. Translated from English (UK) to English (USA) by Oxford Outcomes Ltd.

The following statements all refer to certain difficulties that you may have had during the last 2 weeks. Please indicate, by checking the appropriate box, how often the following statements have been true for you.

If you cannot walk at all
please check **Always/cannot walk at all.**

How often during the last 2 weeks have the following been true?

*Please check **one box** for each question.*

	Never	Rarely	Some-times	Often	Always or cannot walk at all
1. I have found it difficult to walk short distances, e.g. around the house.	☐	☐	☐	☐	☐
2. I have fallen over while walking.	☐	☐	☐	☐	☐
3. I have stumbled or tripped while walking.	☐	☐	☐	☐	☐
4. I have lost my balance while walking.	☐	☐	☐	☐	☐
5. I have had to concentrate while walking.	☐	☐	☐	☐	☐

*Please make sure that you have checked **one box for each question** before going on to the next page.*

ALSAQ-40 © Oxford University Innovation Limited, 2000. All rights reserved. Translated from English (UK) to English (USA) by Oxford Outcomes Ltd.

The following statements all refer to certain difficulties that you may have had <u>during the last 2 weeks.</u> Please indicate, by checking the appropriate box, how often the following statements have been true for you.

If you are not able to perform the activity at all please check **Always/cannot at all**

How often <u>during the last 2 weeks</u> have the following been true?

Please check ***one box*** *for each question*

	Never	Rarely	Some-times	Often	Always or cannot do at all
6. Walking had worn me out.	☐	☐	☐	☐	☐
7. I have had pains in my legs while walking.	☐	☐	☐	☐	☐
8. I have found it difficult to go up and down the stairs.	☐	☐	☐	☐	☐
9. I have found it difficult to stand up.	☐	☐	☐	☐	☐
10. I have found it difficult to move from sitting in a chair to standing upright.	☐	☐	☐	☐	☐

Please make sure that you have checked ***one box for each question*** *before going on to the next page.*

ALSAQ-40 © Oxford University Innovation Limited, 2000. All rights reserved. Translated from English (UK) to English (USA) by Oxford Outcomes Ltd.

The following statements all refer to certain difficulties that you may have had <u>during the last 2 weeks.</u> Please indicate, by checking the appropriate box, how often the following statements have been true for you.

If you cannot do the activity at all   *please check* **Always/cannot do at all.**

How often <u>during the last 2 weeks</u> have the following been true?

Please check ***one box*** *for each question*

	Never	Rarely	Some-times	Often	Always or cannot do at all
11. I have had difficulty using my arms and hands.	☐	☐	☐	☐	☐
12. I have found turning and moving in bed difficult.	☐	☐	☐	☐	☐
13. I have had difficulty picking things up.	☐	☐	☐	☐	☐
14. I have had difficulty holding books or newspapers, or turning pages.	☐	☐	☐	☐	☐
15. I have had difficulty writing clearly.	☐	☐	☐	☐	☐

Please make sure that you have checked ***one box for each question*** *before going on to the next page.*

ALSAQ-40 © Oxford University Innovation Limited, 2000. All rights reserved. Translated from English (UK) to English (USA) by Oxford Outcomes Ltd.

The following statements all refer to certain difficulties that you may have had <u>during the last 2 weeks.</u> Please indicate, by checking the appropriate box, how often the following statements have been true for you.

If you cannot do the activity at all please check **Always/cannot do at all.**

How often <u>during the last 2 weeks</u> have the following been true?

Please check ***one box*** *for each question*

	Never	Rarely	Some-times	Often	Always or cannot do at all
**16. I have found it difficult to do jobs around the house.**	☐	☐	☐	☐	☐
**17. I have found it difficult to feed myself.**	☐	☐	☐	☐	☐
**18. I have had difficulty combing my hair or brushing and/or flossing my teeth.**	☐	☐	☐	☐	☐
**19. I have had difficulty getting dressed.**	☐	☐	☐	☐	☐
**20. I have had difficulty washing at the bathroom sink.**	☐	☐	☐	☐	☐

Please make sure that you have checked ***one box for each question*** *before going on to the next page.*

ALSAQ-40 © Oxford University Innovation Limited, 2000. All rights reserved. Translated from English (UK) to English (USA) by Oxford Outcomes Ltd.

The following statements all refer to certain difficulties that you may have had <u>during the last 2 weeks.</u> Please indicate, by checking the appropriate box, how often the following statements have been true for you.

If you cannot do the activity at all please check **Always/cannot do at all.**

How often <u>during the last 2 weeks</u> have the following been true?

*Please check **one box** for each question*

	Never	Rarely	Some-times	Often	Always or cannot do at all
21. I have had difficulty swallowing.	☐	☐	☐	☐	☐
22. I have had difficulty eating solid food.	☐	☐	☐	☐	☐
23. I have had difficulty drinking liquids.	☐	☐	☐	☐	☐
24. I have had difficulty participating in conversations.	☐	☐	☐	☐	☐
25. I have felt that my speech has not been easy to understand.	☐	☐	☐	☐	☐

*Please make sure that you have checked **one box for each question** before going on to the next page.*

ALSAQ-40 © Oxford University Innovation Limited, 2000. All rights reserved. Translated from English (UK) to English (USA) by Oxford Outcomes Ltd.

The following statements all refer to certain difficulties that you may have had <u>during the last 2 weeks.</u> Please indicate, by checking the appropriate box, how often the following statements have been true for you.

If you cannot do the activity at all please check **Always/cannot do at all.**

How often <u>during the last 2 weeks</u> have the following been true?

Please check ***one box*** *for each question*

	Never	Rarely	Some-times	Often	Always or cannot do at all
26. I have stuttered or slurred my speech.	☐	☐	☐	☐	☐
27. I have had to talk very slowly.	☐	☐	☐	☐	☐
28. I have talked less than I used to do.	☐	☐	☐	☐	☐
29. I have been frustrated with my speech.	☐	☐	☐	☐	☐
30. I have felt self-conscious about my speech.	☐	☐	☐	☐	☐

Please make sure that you have checked ***one box for each question*** *before going on to the next page.*

ALSAQ-40 © Oxford University Innovation Limited, 2000. All rights reserved. Translated from English (UK) to English (USA) by Oxford Outcomes Ltd.

The following statements all refer to certain difficulties that you may have had <u>during the last 2 weeks.</u> Please indicate, by checking the appropriate box, how often the following statements have been true for you.

How often during the last 2 weeks have the following been true?

Please check ***one box*** *for each question*

	Never	Rarely	Some-times	Often	Always
31. I have felt lonely.	☐	☐	☐	☐	☐
32. I have been bored.	☐	☐	☐	☐	☐
33. I have felt embarrassed in social situations.	☐	☐	☐	☐	☐
34. I have felt hopeless about the future.	☐	☐	☐	☐	☐
35. I have worried that I am a burden to other people.	☐	☐	☐	☐	☐

Please make sure that you have checked ***one box for each question*** *before going on to the next page.*

ALSAQ-40 © Oxford University Innovation Limited, 2000. All rights reserved. Translated from English (UK) to English (USA) by Oxford Outcomes Ltd.

The following statements all refer to certain difficulties that you may have had <u>during the last 2 weeks.</u> Please indicate, by checking the appropriate box, how often the following statements have been true for you.

How often <u>during the last 2 weeks</u> have the following been true?

Please check ***one box*** *for each question*

	Never	Rarely	Some-times	Often	Always
36. I have wondered why I keep going.	☐	☐	☐	☐	☐
37. I have felt angry because of the disease.	☐	☐	☐	☐	☐
38. I have felt depressed.	☐	☐	☐	☐	☐
39. I have worried about how the disease will affect me in the future.	☐	☐	☐	☐	☐
40. I have felt as if I have lost my independence	☐	☐	☐	☐	☐

Please make sure that you have checked one ***box for each question.***

Thank you for completing this questionnaire.

ALSAQ-40 © Oxford University Innovation Limited, 2000. All rights reserved. Translated from English (UK) to English (USA) by Oxford Outcomes Ltd.

RGB REGIMEN-SPECIFIC STATISTICAL ANALYSIS PLAN (R-SAP)

**Master Protocol**	Platform Trial for the Treatment of Amyotrophic Lateral Sclerosis (ALS): A perpetual multi-center, multi-regimen, clinical trial evaluating the safety and efficacy of investigational products for the treatment of ALS
**Regimen**	RGB: Verdiperstat
**Regimen Partner**	Biohaven Pharmaceuticals, Inc
**Regulatory Sponsor**	Merit E. Cudkowicz, MD
**Master Protocol Version**	4.0, 31 Aug 2020
**RSA Version**	6.0, 02 Dec 2021
**Master SAP Version**	1.0, 24 Jun 2020
**R-SAP Version**	3.0, 22 Jul 2022

SAP APPROVAL SIGNATURES

Merit E. Cudkowicz, MD, MSc<br>Principal Investigator and Regulatory Sponsor	Date
Sabrina Paganoni, MD, PhD<br>Co-Principal Investigator	Date
Daniel Campbell, PhD<br>Director, Biostatistics, Biohaven Pharmaceuticals, Inc	Date

Ben Saville, PhD | Date
Senior Statistical Scientist, Berry Consultants

Eric A. Macklin, PhD | Date
Study Biostatistician

SAP REVISION HISTORY

Version	Date	Description of Changes
1.0	17 Mar 2022	Initial version
2.0	06 May 2022	Revision of Section 5.9 Survival to specify that both PAV-free survival and overall survival will be evaluated at both the Week 24 Visit time point and the last-participant-last-visit time point and to specify that PAV-free survival to the Week 24 Visit time point is the primary analysis of survival in this analysis plan.<br>Revision of Section 6.5.5 CAFS to specify the following:<br>1. CAFS will be used as a supportive analysis for the secondary efficacy endpoints of HHD upper and lower extremity percentage and SVC,<br>2. Additional CAFS analyses will use multiple imputation to extend follow-up for participants who early terminate, withdraw consent, or are lost to follow-up,<br>3. Additional CAFS analyses will use time to death alone independent of any death equivalent, and<br>4. Primary inference from CAFS analyses will compare survival by time to death or death equivalent and will compare change in function to the last jointly observed time point.
3.0	22 Jul 2022	Revision of Section 4.2 Exploratory Endpoints to identify serum creatinine and serum and CSF neurofilament light chain (NfL) as exploratory biomarkers of neurodegeneration and neuromuscular degeneration and to include ALSAQ-40 domain scores and symptom index (SI) as exploratory endpoints.<br>Revision of Section 5.1 ALSFRS-R to specify details of the calculation of pre-baseline slope.<br>Revision of Section 5.5 Quantitative Voice Characteristics to include predicted vital capacity as an additional metric at the Baseline Visit.<br>Revision of Section 5.6 Biofluid Biomarkers of Neurodegeneration to specify the assay techniques used to quantify serum creatinine and serum and CSF NfL and to specify that levels of serum and CSF NfL will be log-transformed in all analyses.

Version	Date	Description of Changes
3.0 (continued)	22 Jul 2022 (continued)	Revision of Section 5.7 ALSAQ-40 to specify calculation of domain scores and to revise calculation of overall ALSAQ-40 SI.<br><br>Revision of Section 5.8 CNS-BFS to specify that the total score is referenced.Revision of Section 5.9 Survival to specify that time at risk begins at each participant's Baseline Visit and to specify that the date of PAV initiation, where applicable, will be imputed as the fifteenth day of a month if not specified more precisely.<br><br>Revision of Section 6.1 Analysis Sets to add the Efficacy Common Mode of Administration (ECM) analysis set, to remove the restriction on protocol deviations that could be considered for exclusion from the Efficacy Per-protocol (EPP) analysis set must be classified as major protocol deviations, to specify the time point at which data is excluded from the EPP analysis set in the case of time-dependent exclusions, and to specify that data from placebo participants from other regimens would not be excluded from the EPP analysis set due to non-adherence to protocol-specified dosing.<br><br>Revision of Section 6.2 Baseline Characterization to include ALSAQ-40 domain scores and SI.<br><br>Revision of Section 6.5.2 Repeated-measures Model to add a fixed term for treatment group (removing the shared-baseline assumption at the recommendation of the FDA) and to specify a separate supportive analysis that includes fixed terms for centered baseline serum NfL level and centered baseline serum NfL level × visit interaction.<br><br>Revision of Section 6.5.3 Random-slopes Model to add a fixed term for treatment group (removing the shared-baseline assumption) and to specify a separate supportive analysis that includes fixed terms for centered baseline serum NfL level and centered baseline serum NfL level × study month interaction.<br><br>Revision of Section 6.5.4 Survival and Time to Clinical Events to clarify that survival analyses that include follow-up beyond the placebo-controlled period will be analyzed in the ERO analysis set, to include baseline age as an additional covariate in all adjusted models, and to specify an additional adjusted analysis that includes baseline serum NfL level as a covariate.

Version	Date	Description of Changes
3.0 (continued)	22 Jul 2022 (continued)	Revision of Section 6.5.5 CAFS to clarify that the primary CAFS analysis is specified in the ALS Master Protocol Recommended Statistical Analysis, Design and Simulation Report and to add two additional sets of CAFS analyses that adjust rank scores in linear models, one set that adjusts for time from ALS symptom onset, delta-FRS, baseline use of riluzole, and baseline use of edaravone, and one set that adjusts for the same set of covariates plus baseline serum NfL level.<br><br>Revision of Section 6.5.6 HHD0 and $HHD0^2$ to remove reference to the shared-baseline assumption of the repeated-measures mixed model of Section 6.5.2 and to specify a separate analysis that adds baseline serum NfL level as an additional covariate.<br><br>Revision of Section 6.5.7 Quantitative Voice Measures to remove reference to the shared-baseline assumption of the random-slopes mixed model of Section 6.5.3 and to add a fixed term for treatment group (removing the shared-baseline assumption) and to specify a separate analysis that includes fixed terms for centered baseline serum NfL level and centered baseline serum NfL level × B-spline interaction.<br><br>Revision of Section 6.5.8 Placebo Multiple Imputation to specify regression over sequential visits by the fully conditional specification method, to remove reference to the shared-baseline assumption of the repeated-measures mixed model of Section 6.5.2, and to specify a separate analysis that adds baseline serum NfL level as an additional covariate.<br><br>Revision of Section 6.5.11 Comparison of Controls across Regimens to specify separate analyses that add baseline serum NfL level as an additional covariate.

ABBREVIATIONS

ALP	Alkaline Phosphatase
ALS	Amyotrophic Lateral Sclerosis
ALSAQ-40	Amyotrophic Lateral Sclerosis Assessment Questionnaire, 40-item version
ALSFRS-R	Amyotrophic Lateral Sclerosis Functional Rating Scale, Revised
ALT	Alanine Transaminase
AST	Aspartate Transaminase
ATC	WHODrug Anatomical, Therapeutic, and Chemical class
ATS	American Thoracic Society
BLQ	Below the Limit of Quantitation
BMI	Body Mass Index
C-SSRS	Columbia Suicide Severity Rating Scale
CAFS	Combined Assessment of Function and Survival
CBC	Complete Blood Count
CKD	Chronic Kidney Disease
COVID-19	Coronavirus Disease 2019
CNS-BFS	Center for Neurologic Study Bulbar Function Scale
CSF	Cerebrospinal Fluid
CSR	Clinical Study Report
CTCAE	Common Terminology Criteria for Adverse Events
delta-FRS	Pre-baseline Slope in ALSFRS-R
DAP	Data Analysis Plan
DILI	Drug-induced Liver Injury
DNA	Deoxyribonucleic Acid
DRR	Disease Rate Ratio
ECC	Efficacy Concurrent Control
ECG	Electrocardiography or Electrocardiogram
eGFR	Estimated Glomerular Filtration Rate
EPP	Efficacy Per-protocol
ERO	Efficacy Regimen-only
ELISA	Enzyme-linked Immunosorbent Assay
FAS	Full Analysis Set

ABBREVIATIONS (continued)

FVC	Forced Vital Capacity
GLI	Global Lung Initiative
hCG	Human Chorionic Gonadotropin
HHD	Hand-held Dynamometry
HLT	MedDRA High Level Term
ICF	Informed Consent Form
ITT	Intention-to-treat Principle
M-SAP	Master Statistical Analysis Plan
MDRD	Modification of Diet in Renal Disease
MedDRA	Medical Dictionary for Regulatory Activities
MP	Master Protocol
MPRDR	ALS Master Protocol Recommended Statistical Analysis, Design and Simulation Report
NCI	National Cancer Institute
NEALS	Northeast ALS
NfL	Neurofilament Light Chain
NIV	Noninvasive Ventilation
OLE	Open-label Extension
PAV	Permanent Assisted Ventilation
PD	Pharmacodynamics
PK	Pharmacokinetics
PT	MedDRA Preferred Term
RBC	Red Blood Cell
RDW	RBC Distribution Width
RGB	Regimen B (verdiperstat)
RSA	Regimen-specific Appendix
R-SAP	Regimen-specific Statistical Analysis Plan
SAE	Serious Adverse Event
SAP	Statistical Analysis Plan
SGOT	Serum Glutamic Oxaloacetic Transaminase
SGPT	Serum Glutamic Pyruvic Transaminase

ABBREVIATIONS (continued)

SI	Symptom Index
SoA	Schedule of Activities
SOC	MedDRA System Organ Class
SRO	Safety Regimen-only
STF	Safety and Tolerability Full
STN	Safety and Tolerability Narrow
SVC	Slow Vital Capacity
TBL	Total Bilirubin
TEAE	Treatment-emergent Adverse Event
TSH	Thyroid Stimulating Hormone
ULN	Upper Limit of Normal
WBC	White Blood Cell
WHODrug	World Health Organization Drug Dictionary Enhanced

Table of Contents

1. Governing Documents 11
2. Study Design 11
2.1 Overview 11
2.2 Study Objectives 12
2.3 Study Population 12
2.4 Participant Flow 12
2.5 Regimen Allocation 12
2.6 Treatment Allocation 12
2.7 Treatment Administration 13
2.8 Allocation Concealment 13
2.9 RGB Schedule of Activities (SoA) 13
3. General Considerations for Data Analysis 15
3.1 Statistical Software 15
3.2 Summary Statistics 15
3.3 Precision 15
3.4 Transformations 15
3.5 Multiplicity Adjustments 15
3.6 Missing Data 16
4. Study Endpoints 16
4.1 Efficacy Endpoints 16
4.2 Exploratory Endpoints 16
4.3 Safety Endpoints 16
5. Measurement Definitions 17
5.1 ALSFRS-R 17
5.2 SVC 17
5.3 Home Spirometry 17
5.4 HHD and Grip Strength 17
5.5 Quantitative Voice Characteristics 18
5.6 Biofluid Biomarkers of Neurodegeneration 18
5.7 ALSAQ-40 18
5.8 CNS-BFS 18
5.9 Survival 18
5.10 King's ALS Clinical Staging System 19
5.11 Hospitalization and Other Clinical Events 19
5.12 Pharmacodynamic Biomarkers 20
5.13 Clinical Safety Laboratory Tests 20
6. Statistical Methodology 21
6.1 Analysis Sets 21
6.2 Baseline Characterization 22
6.3 Primary Efficacy Analysis and Supportive Analyses 22
6.4 Interim Analysis 22
6.5 Secondary Efficacy Analyses 23
6.5.1 Hierarchical Testing 23
6.5.2 Repeated-measures Model 23
6.5.3 Random-slopes Model 25
6.5.4 Survival and Time to Clinical Events 26
6.5.5 CAFS 27
6.5.6 HHD0 and $HHD0^2$ 27
6.5.7 Quantitative Voice Measures 27
6.5.8 Placebo Multiple Imputation 28
6.5.9 Additional Sensitivity Analyses of Primary and Key Secondary Outcomes 29
6.5.10 Subgroup Analyses 29
6.5.11 Comparison of Controls across Regimens 29
6.5.12 Pharmacokinetic Analyses 30

6.5.13 Pharmacodynamic Biomarker Analyses 30
6.6 Safety Analyses 30
6.6.1 Treatment-emergent Adverse Events 30
6.6.2 Safety Labs 31
6.6.3 ECG Results 31
6.6.4 Vital Signs and Weight 31
6.6.5 Suicidality 31
6.7 Other Analyses 32
6.7.1 Participant Disposition 32
6.7.2 Study Drug Compliance and Tolerance 32
6.7.3 Concomitant Medication Use 33
6.7.4 Medical History 33
6.7.5 Blindedness 33
6.7.6 Protocol Deviations 33
6.7.7 Impact of COVID-19 Pandemic 33
7. Validation 33
7.1 Primary Efficacy Analysis 33
7.2 Secondary, Exploratory, and Safety Analyses 33
8. References 34

1. Governing Documents

This Regimen-specific Statistical Analysis Plan (R-SAP) for the verdiperstat regimen (RGB) specifies any modification from the default outcome measures, analysis samples, and planned analyses for the placebo-controlled period of the HEALEY ALS Platform Trial as specified in the Master SAP (M-SAP). The M-SAP and this R-SAP supplement the Master Protocol, the "ALS Master Protocol Recommended Statistical Analysis, Design and Simulation Report" (Appendix 1 to the Master Protocol), and the RGB Regimen-specific Appendix (RSA). Please refer to the Master Protocol and the RGB RSA for details on the rationale for the study design, eligibility criteria, conduct of the trial, clinical assessments and schedule of assessments, definitions and reporting of adverse events, data management conventions, and regulatory oversight and compliance procedures. The "ALS Master Protocol Recommended Statistical Analysis, Design and Simulation Report" (MPRDR) and any regimen-specific deviations described in the RGB RSA and this R-SAP are authoritative in defining the primary and interim analyses. In case of discrepancies between the RGB RSA and this R-SAP concerning use of shared placebos, this R-SAP is authoritative. In case of discrepancies between either SAP and the Master Protocol and the RGB RSA concerning matters of analysis other than the primary and interim analyses and use of shared placebos, the M-SAP and this R-SAP are authoritative. In case of discrepancies between the M-SAP and this R-SAP, this R-SAP is authoritative. On all matters not related to analysis, the Master Protocol and the RGB RSA are authoritative. The following table describes relationships among the relevant documents in adjudicating possible discrepancies with higher numbers indicating greater authority.

Issues potentially requiring adjudication	Master Protocol	RGB RSA	MPRDR	M-SAP	RGB R-SAP
Use of shared placebos	1	4	2	3	5
Primary and interim analysis specifications not related to use of shared placebo	1	5	4	2	3
Statistical analysis specifications not related to use of shared placebo or primary and interim analyses	1	3	2	4	5
All matters not related to statistical analysis	4	5	1	2	3

2. Study Design

2.1 Overview

The HEALEY ALS Platform Trial is a perpetual multi-center, multi-regimen clinical trial evaluating the safety and efficacy of investigational products for the treatment of ALS. RGB evaluates the safety and efficacy of verdiperstat administered orally at a dosage of 600 mg BID vs. placebo. The RGB RSA describes the nature of the intervention and its mechanism of action, the mode and frequency of administration, additional eligibility criteria beyond those specified in the Master Protocol, additional enrollment procedures, and additions and modifications of safety and efficacy assessments relative to those outlined in the Master Protocol.

2.2 Study Objectives

Primary Efficacy Objective:

- To evaluate the efficacy of verdiperstat as compared to placebo on ALS disease progression.

Secondary Efficacy Objectives:

- To evaluate the effect of verdiperstat on selected secondary measures of disease progression, including survival.

Safety Objectives:

- To evaluate the safety of verdiperstat for ALS patients.

Exploratory Efficacy Objectives:

- To evaluate the effect of verdiperstat on selected biomarkers and endpoints.
- To explore verdiperstat pharmacokinetics (PK) and pharmacodynamic (PD) effects.

2.3 Study Population

In addition to eligibility criteria specified in the Master Protocol, participants in RGB must not be taking strong inhibitors of CYP1A2 (i.e., ciprofloxacin, enoxacin, fluvoxamine, zafirlukast) or CYP3A4 (i.e., conivaptan, itraconazole, ketoconazole, posaconazole, troleandomycin, voriconazole, clarithromycin, diltiazem, idelalisib, nefazodone, and certain antiviral agents [cobicistat, danoprevir, ritonavir, elvitegravir, indinavir, lopinavir, paritaprevir, ombitasavir, dasabuvir, saquinavir, tipranavir, nelfinavir]) for chronic/long-term use, defined as more than two weeks.

Participants will be recruited from approximately 60 centers located throughout the US that are part of the Northeast ALS (NEALS) Consortium.

2.4 Participant Flow

Participants in RGB follow the consenting, Master screening, regimen assignment, regimen-specific screening, randomization to active or placebo treatment, and follow-up procedures and timing described in the M-SAP. Detailed descriptions of study procedures and timing are specified in the Master Protocol and the RGB RSA.

2.5 Regimen Allocation

Participants in RGB are those determined eligible for Master Protocol-level inclusion and exclusion criteria and randomly assigned to RGB, stratified by use of riluzole, edaravone, both, or neither at the time of screening for the Master Protocol. Details of regimen assignment are described in the Platform Trial Regimen Assignment Plan.

2.6 Treatment Allocation

Participants in RGB are randomly allocated in a 3:1 ratio to active or placebo treatment based on a pre-specified permuted-block randomization schedule, stratified by use of riluzole, edaravone, both, or neither at the time of screening for the Master Protocol.

2.7 Treatment Administration

Verdiperstat and placebo are supplied as matching reddish-beige, film coated, oval tablets. Each tablet of active study drug contains 300 mg of verdiperstat as the free base plus excipients generally recognized as safe for oral administration in an extended-release formulation.

The first dose of study drug should be administered while in the office/clinic on the day of the Baseline Visit after all visit assessments are complete. Participants should stay at the office/clinic for monitoring for approximately 30 minutes post-dose. From the Baseline Visit to the end of Week 1, participants should take one tablet of study drug per day. During Week 2, participants should take one tablet of study drug twice per day. Starting at the beginning of Week 3 and throughout the remainder of the study, participants should take two tablets of study drug twice per day. If tolerability issues are experienced, the titration schedule may be modified. If a participant is off study drug for more than 14 consecutive days, the participant should re-escalate using the titration paradigm described above.

Additional details of treatment administration are described in the RGB RSA.

2.8 Allocation Concealment

Allocation concealment is the same as described in the M-SAP.

2.9 RGB Schedule of Activities (SoA)

Activity	MP Scrn[1]	RGB Scrn[1]	Base-line	Week 2[2]	Week 4[18,19]	Week 8[18,19]	Week 12	Week 16[18,19]	Week 20	Week 24[3,18]	Final Call[3,4]
	Cln	Cln	Cln	Phn	Cln[5]	Cln[5]	Phn	Cln[5]	Phn	Cln	Phn
	-42d to -1d	-41d to 0d	Day 0	Day 14±3	Day 28±7	Day 56±7	Day 84±3	Day 112±7	Day 140±3	Day 168±7	28d ±3 ALD
Written Informed Consent[5]	X	X									
Inclusion/Exclusion Review	X	X[6]									
ALS & Medical History	X										
Demographics	X										
Physical Examination	X										
Neurological Exam	X										
Vital Signs[7]	X		X		X	X		X		X	
Slow Vital Capacity	X[20]		X			C		C		X	
Home Spirometry	X[20]		X			X		X		X	
Muscle Strength Assessment			X			C		C		X	
ALSFRS-R	X		X		X	X	X	X	X	X	
ALSAQ-40			X							X	
CNS-BFS			X			X		X		X	
12-Lead ECG	X					C				X	
Clinical Safety Labs[8]	X		X		X	X		X		X	
Verdiperstat PK Samples[10]			X		C	C		C		X	
Verdiperstat PD Samples[10]			X		C	C		C		X	
Biomarker Blood Collection			X			C		C		X	
Biomarker Urine Collection			X			C		C		X	
DNA Collection[11] (optional)			X								
CSF Collection (optional)			X					C[17]			
Concomitant Medication Review	X	X	X	X	X	X	X	X	X	X	
Adverse Event Review[9]	X	X	X	X	X	X	X	X	X	X	X
Suicidality C-SSRS			X		X	X		X		X	
Install Smartphone Apps[21]			X								

Activity	MP Scrn[1]	RGB Scrn[1]	Base-line	Week 2[2]	Week 4[18,19]	Week 8[18,19]	Week 12	Week 16[18,19]	Week 20	Week 24[3,18]	Final Call[3,4]
	Cln	Cln	Cln	Phn	Cln[5]	Cln[5]	Phn	Cln[5]	Phn	Cln	Phn
	-42d to -1d	-41d to 0d	Day 0	Day 14±3	Day 28±7	Day 56±7	Day 84±3	Day 112±7	Day 140±3	Day 168±7	28d ±3 ALD
Smartphone Voice Recording[12]			X		X	X		X		X	
Uninstall Smartphone App										X	
Regimen Assignment	X										
Randomization within RGB			X								
Administer/Dispense Study Drug			X[13]		X	X		X			
Drug Accountability/Compliance				X[2,22]	X	X	X[22]	X	X[22]	X	
Dose Escalation			X[15]	X[15]							
Exit Questionnaire										X	
Vital Status										X[16]	

Abbreviations: ALD = after last dose, ALS = amyotrophic lateral sclerosis, ALSAQ-40 = ALS Assessment Questionnaire, ALSFRS-R = ALS Functional Rating Scale Revised, BP = blood pressure, C = completed only if the visit is conducted in-clinic, CBC = complete blood count, Cln = Clinic visit, CNS-BFS = Center for Neurologic Study Bulbar Function Scale, CSF = cerebrospinal fluid, C-SSRS = Columbia-Suicide Severity Rating Scale, d = day, DNA = deoxyribonucleic acid, ECG = electrocardiogram, LFTs = liver function tests, MP = Master Protocol, PD = pharmacodynamic, Phn = Phone visit, RGB = the verdiperstat regimen, Scrn = Screening Visit.

[1] Master Protocol Screening procedures must be completed within 42 days to 1 day prior to the Baseline Visit. The Regimen-Specific Screening Visit and Baseline Visit should be combined if possible.

[2] At the end of Week 2, an assessment of compliance and tolerance to this dose titration schedule will be conducted. The assessment will be conducted by phone. If tolerability issues are experienced with 300 mg QD or 300 mg BID dosing, the titration schedule may be modified (see RSA Section 5.3.2).

[3] Participants will only have a Follow-Up Safety Call at this time if they do not continue into the OLE or if they discontinue prior to Week 24. Participants who continue into OLE will have a Follow-Up Safety Call after their last dose of study drug during the OLE phase.

[4] Participants who continue into the OLE and then early terminate will be asked to complete an Early Termination Visit and Follow-Up Safety Call as described in the body of this RSA.

[5] During the Master Protocol Screening Visit, participants will be consented via the Master Protocol informed consent form (ICF). After a participant is randomized to a regimen, participants will be consented a second time via the RSA ICF.

[6] At the Regimen Specific Screening Visit, participants will have regimen-specific inclusion and exclusion criteria assessed, if applicable.

[7] Vital signs include weight, systolic and diastolic pressure, respiratory rate, heart rate and temperature. Height is measured at Master Protocol Screening Visit only.

[8] Clinical safety labs include hematology (CBC with differential), complete chemistry panel, thyroid function (TSH) and urinalysis. Serum pregnancy testing will occur in women of child-bearing potential at the Master Protocol Screening Visit and as necessary during the study. Pregnancy testing is only repeated as applicable if there is a concern for pregnancy.

[9] Adverse events that occur after signing the master protocol consent form will be recorded.

[10] Myeloperoxidase protein and activity, and plasma concentrations of verdiperstat will be measured. For each sample, the time of the last verdiperstat or matching placebo dose prior to sample collection, time of the last meal prior to sampling and time of the PD/PK sample collection should be reported on the CRF.

[11] The DNA sample can be collected after baseline if a baseline sample is not obtained or the sample is not usable.

[12] In addition to study visits outlined in the SOA, participants may be asked to complete twice weekly voice recordings at home. During weeks when a participant is doing a voice recording in-clinic, he or she would only do one other voice recording at home that week.

[13] Administer first dose of investigational product (IP) only after Baseline Visit procedures are completed. Participants should take the first dose of IP while in the office/clinic on the day of the Baseline visit and stay at the clinic for approximately 30 minutes post-dose for observation.

[14] Investigational product will only be dispensed at this visit if the participant continues in the OLE.

[15] From start to end of Week 1, participants will ingest either 300 mg QD of verdiperstat or matching placebo QD. From start to end of Week 2, participants will ingest either 300 mg BID of verdiperstat or matching placebo BID. Starting with Week 3 and continuing to Week 24, participants will ingest either 600 mg BID of verdiperstat or matching placebo BID.

[16] Vital status, defined as a determination of date of death or death equivalent or date last known alive, will be determined for each randomized participant at the end of the placebo-controlled portion of their follow-up (generally the Week 24 Visit, as indicated). If at that time the participant is alive, his or her vital status should be determined again at the time of the last participant's last visit of the placebo-controlled portion of a given regimen. We may also ascertain vital status at later time points by using publicly available data sources as described in section 8.15 of the Master Protocol.

[17] If the CSF collection cannot happen at the Week 16 Visit for logistical reasons such as scheduling, it can happen at the Week 24 Visit.

[18] Participants should be instructed to skip the morning dose of study drug on the day of the study visit. Study drug should not be taken until after study visit procedures are complete.

[19] Visit may be conducted via phone or telemedicine with remote services instead of in-person if this is needed to protect the safety of the participant due to a pandemic or other reason.

[20] If required due to pandemic-related restrictions, Forced Vital Capacity (FVC) performed by a Pulmonary Function Laboratory evaluator or with a study-approved home spirometer, or sustained phonation using a study approved method may be used for eligibility (Master Protocol Screening ONLY).

[21] Two smartphone apps should be installed on the participant's phone, one to collect the voice recordings and one to collect home spirometry.

[22] Drug accountability will not be done at phone visits. A drug compliance check in must be held during phone visits to ensure participant is taking drug per dose regimen and to note any report of missed doses.

3. General Considerations for Data Analysis

3.1 Statistical Software

Statistical software use for analyses is the same as described in the M-SAP.

3.2 Summary Statistics

Data summaries are the same as described in the M-SAP.

3.3 Precision

Precision of reported results is the same as described in the M-SAP.

3.4 Transformations

Data transformations are the same as described in the M-SAP.

3.5 Multiplicity Adjustments

Handling of multiplicity adjustments is the same as described in the M-SAP.

3.6 Missing Data

Handling of missing data is the same as described in the M-SAP. Clinic-based assessments that are missing due to COVID-19 restrictions or disruptions are considered missing at random.

4. Study Endpoints

4.1 Efficacy Endpoints

The primary and secondary efficacy endpoints are the same as described in the M-SAP. ALSFRS-R total score is considered the primary efficacy endpoint and hand-held dynamometry (HHD) upper and lower extremity percentages, slow vital capacity (SVC), and survival are considered key secondary efficacy endpoints in RGB.

4.2 Exploratory Endpoints

The following categories of exploratory endpoints will be evaluated:

- Change in ALSFRS-R domain scores
- Change in strength: HHD global percentage, HHD0, and $HHD0^2$,
- Change in quantitative voice characteristics as measured by Aural Analytics: maximum phonation time, pause rate, breathy vocal quality, pitch instability, regulation of voicing, articulatory precision, speaking rate, articulation rate, and monotonicity,
- Change in biofluid biomarkers of neurodegeneration and neuromuscular degeneration: serum creatinine and serum and cerebrospinal fluid (CSF) neurofilament light chain (NfL),
- Change in patient-reported outcomes: ALSAQ-40 physical mobility, independence in activities of daily living, eating and drinking, communications, and emotional reactions domain scores and ALSAQ-40 symptom index, CNS-BFS total score,
- Change in plasma concentrations of verdiperstat,
- Change in verdiperstat PD biomarkers: myeloperoxidase protein, total activity, and concentration-specific activity,
- Change in respiratory function as assessed by home spirometry, and
- Time to clinical events: first hospitalization due to a serious adverse event (SAE), first hospitalization due to an ALS-related SAE, first use of assisted ventilation, first placement of a feeding tube, first time reaching King's stage 4a or 4b, and first instance of any of the following events: hospitalization for an SAE, feeding tube placement, tracheostomy, initiation of permanent assisted ventilation (PAV), or death.

4.3 Safety Endpoints

In addition to the safety endpoints described in the M-SAP, the following RGB regimen-specific safety endpoint will be evaluated:

- Thyroid function: Proportion of participants with TSH ≥10 mIU/L, proportion of participants with signs or symptoms of hypothyroidism, and levels of free T3, free T4, and TSH.

5. Measurement Definitions

5.1 ALSFRS-R

The definitions of ALSFRS-R scores are the same as described in the M-SAP. Pre-baseline slope in ALSFRS-R (delta-FRS) is defined as 48 minus the baseline ALSFRS-R total score then divided by the number of months from onset of symptomatic weakness to the Baseline Visit. The number of months will be calculated as the difference in days from onset of symptomatic weakness to the Baseline Visit multiplied by 12 / 365.25. The date of onset of symptomatic weakness will be imputed as the fifteenth day of a month if not specified more precisely.

ALSFRS-R domain scores are exploratory measures of the primary efficacy endpoint ALSFRS-R total score.

5.2 SVC

The derivation of SVC percent-predicted of normal is the same as described in the M-SAP with age calculated as number of days from date of birth to the date of a given SVC assessment divided by 365.25 and with the following correspondence between self-identified race and race defined by Global Lung Initiative (GLI) classification:

Self-identified Race	GLI-defined Race
American Indian or Alaska Native	Mixed/Other
Asian	South East Asian
Black or African American	African American
Native Hawaiian or Other Pacific Islander	Mixed/Other
White	Caucasian
Unknown	Caucasian
Not reported	Caucasian
More than one race indicated	Mixed/Other

5.3 Home Spirometry

Home spirometry assesses FVC remotely using a smartphone app (ZEPHYRx, Albany, NY) and a handheld spirometer (Spirobank Smart, Medical International Research, Rome, Italy). Coordinators guide participants through 3 to 8 maneuvers with live-video coaching using the ZEPHYRx platform. Flow loops are classified for acceptability and repeatability using American Thoracic Society (ATS) criteria and are manually reviewed by the NEALS Outcomes Center (Barrow Neurological Institute, Phoenix, AZ). The maximum FVC accepted by the NEALS Outcomes Center is converted to percent of predicted normal using GLI norms based on sex, age at time of assessment, height at time of screening, and race. Age is calculated as number of days from date of birth to the date of a given home spirometry assessment divided by 365.25. Higher values indicate greater respiratory function.

5.4 HHD and Grip Strength

The derivation of HHD upper and lower extremity scores and HHD0 are the same as described in the M-SAP with the revision that HHD0 is a composite endpoint with death or death equivalent, whichever occurs first.

A second HHD time-to-event endpoint is defined as the time from the Baseline Visit to the second post-baseline occurrence of a muscle with a strength recording of 0 among those muscles that were non-zero at baseline or time to death or death equivalent, whichever occurs first ($HHD0^2$).

Time at risk for HHD0 and $HHD0^2$ will be censored at the last date at an HHD assessment was performed up to the end of the Week 24 Visit window.

HHD global average percentage, HHD0, and $HHD0^2$ are exploratory measures of the secondary endpoint HHD and grip strength.

5.5 Quantitative Voice Characteristics

Voice samples will be collected using the Aural Analytics app installed on either an Android or iOS-based smartphone. At each assessment, participants perform a set of speaking tasks: reading 5 prespecified sentences, reading 5 sentences chosen at random from a large sentence bank, repeating a consonant-vowel sequence, producing a sustained phonation, and counting on a single breath. Speech analysis will be performed by Aural Analytics to derive the following quantitative voice characteristics: maximum phonation time, pause rate, breathy vocal quality, pitch instability, regulation of voicing, articulatory precision, speaking rate, articulation rate, and monotonicity. Aural Analytics will use data on quantitative voice characteristics and participant age, sex, race, height, and weight to derive a prediction of vital capacity at the Baseline Visit.

5.6 Biofluid Biomarkers of Neurodegeneration

Blood biomarkers of neurodegeneration, including biomarkers of neuromuscular dysfunction, will be assayed. These will include serum creatinine and serum and CSF neurofilament light chain (NfL). Serum creatinine will be assayed by the kinetic Jaffe method (test 001370, Labcorp, Burlington, NC). NfL will be assayed by single-molecule array (Simoa; Quanterix, Billerica, MA). Levels of serum and CSF NfL that are reported to be below the limit of quantitation will be imputed at the limit of quantitation. Levels of serum and CSF NfL will be log-transformed in all analyses.

5.7 ALSAQ-40

The description of the ALSAQ-40 instrument and item-level scores are the same as described in the M-SAP. Each of the five domains will be scored as the mean of all domain-specific items multiplied by 25 (range 0 to 100). An overall symptom index (SI) will be scored as the mean of the five domain scores. A domain score will be missing if more than 20% of the items are missing; otherwise, item non-response will be mean-imputed from other completed items from the same assessment. The ALSAQ-40 SI will be missing if any domain scores are missing. Higher scores indicate worse quality of life.

5.8 CNS-BFS

The definition of CNS-BFS total score is the same as described in the M-SAP.

5.9 Survival

The primary definition of survival time is the same as described in the M-SAP with the clarification that PAV is defined as more than 22 hours per day of noninvasive or invasive

mechanical ventilation for more than seven consecutive days. The date of PAV initiation, where applicable, will be imputed as the fifteenth day of a month if not specified more precisely. A secondary survival endpoint of death alone, independent of any death equivalent, is also defined.

Time at risk for the composite endpoint of death or death equivalent and time at risk for the endpoint of death alone will be measured from each participant's Baseline Visit. Time at risk will be censored at two time points: (1) at the Week 24 Visit as defined in the M-SAP, and (2) at a subsequent assessment of death or death equivalent scheduled approximately at the end of placebo-controlled follow-up of the last RGB participant. The primary analysis of survival will evaluate PAV-free survival to the Week 24 Visit time point.

5.10 King's ALS Clinical Staging System

The King's ALS Clinical Staging System (Roche et al. 2012) is a 4-level ordinal scale with the first three levels indicating the number (1, 2, or 3) of distinct central nervous system regions (bulbar, upper limb, and lower limb) with neuromuscular dysfunction and levels 4a and 4b indicating nutritional or respiratory failure secondary to ALS, respectively.

Participants will be classified to King's stage 1, 2, 3, 4a, or 4b based on scores from ALSFRS-R assessments according to a published derivation (Balendra et al. 2014). Bulbar involvement is defined as a score less than 4 on any of the ALSFRS-R questions in the bulbar domain (questions 1, 2, and 3). Upper limb involvement is defined as a score less than 4 on either of the ALSFRS-R questions related to hand function (questions 4 and 5A). Lower limb involvement is defined as a score less than 4 on the ALSFRS-R question about walking (question 8). Nutritional failure is defined as responding that the participant uses gastrostomy for greater than 50% of their nutrition. Respiratory failure is defined as a score of 0 on the ALSFRS-R question addressing dyspnea (question 10 or R-1) or a score less than 4 on the ALSFRS-R question about use of mechanical ventilation (question 12 or R-3). Participants without evidence by ALSFRS-R scores of involvement of any of the three central nervous system regions will be scored as King's stage 1 due to their confirmed diagnosis with ALS. Participant may meet criteria for both King's stage 4a and 4b.

5.11 Hospitalization and Other Clinical Events

Times to the following clinically relevant events are defined:

- Time to first hospitalization due to a serious adverse event (SAE),
- Time to first hospitalization due to an ALS-related SAE,
- Time to first use of assisted ventilation,
- Time to first placement of a feeding tube,
- Time to King's stage 4a or 4b, and
- Time to first instance of hospitalization for an SAE, feeding tube placement, tracheostomy, initiation of PAV, or death.

Time at risk for each event will be measured from each participant's Baseline Visit. Time to first hospitalization excludes hospitalizations for elective procedures. ALS-related SAEs are those indicated as related to ALS disease progression by the site investigator. Participants who are already using assisted ventilation or have a feeding tube at the time of the Baseline Visit will be

excluded from analysis of those endpoints. Death or death equivalent will be considered an outcome for each of the events listed, forming a composite endpoint.

Time at risk for these events will be censored at the Week 24 Visit, if completed, the date of consent withdrawal, if withdrawn, or the last date at which the status of each endpoint is known prior to the end of the Week 24 Visit window for participants lost to follow-up. Time to King's stage 4a or 4b is interval censored between ALSFRS-R assessments.

5.12 Pharmacodynamic Biomarkers

Myeloperoxidase protein and activity levels in plasma samples collected at Baseline and Weeks 4, 8, 16, and 24 will be assayed among participants randomized to active treatment. Details of the assay techniques will be specified when known.

5.13 Clinical Safety Laboratory Tests

Clinical safety labs include hematology, blood chemistry panel, liver function tests, thyroid function, urinalysis, and pregnancy testing in women of childbearing potential as specified in Section 9.1.2 Clinical Safety Laboratory Tests of the Master Protocol:

- Hematology: hematocrit, hemoglobin, platelet count, red blood cell (RBC) count, mean corpuscular volume, mean corpuscular hemoglobin, mean corpuscular hemoglobin concentration, RBC distribution width (RDW), RBC morphology, white blood cell (WBC) count, and counts and percentages of basophils, eosinophils, lymphocytes, monocytes, and neutrophils;
- Blood chemistry panel: bicarbonate, chloride, potassium, sodium, calcium, magnesium, phosphate, blood urea nitrogen, creatinine, estimated glomerular filtration rate (eGFR) calculated using the Modification of Diet in Renal Disease (MDRD) four-variable equation, creatinine clearance calculated using the Cockcroft-Gault equation, and glucose;
- Liver function tests: alanine aminotransferase (ALT [SGPT]), aspartate aminotransferase (AST [SGOT]), alkaline phosphatase (ALP), albumin, total protein, total bilirubin (TBL);
- Thyroid function tests: thyroid-stimulating hormone (TSH), reflex T3 and T4 when TSH levels are abnormal;
- Urinalysis: clarity, color, specific gravity, pH, microalbumin, protein, glucose, ketones, bilirubin, urobilinogen, nitrite, leukocyte esterase, and blood; and
- Pregnancy: qualitative and quantitative serum human chorionic gonadotropin (hCG).

Clinical safety labs will also include derived measures of potential drug-induced liver injury (DILI), including those that potentially meet the Hy’s law criteria, as distinct safety lab outcomes.

Three potential DILI criteria will be defined:

- ALT or AST >3x ULN with TBL >1.5x ULN
- AST or ALT >3x ULN with TBL >2x ULN
- AST or ALT >3x ULN with TBL >2x ULN and ALP <2x ULN (potential Hy’s Law cases)

where ULN is upper level of normal and all levels are measured on the same day.

6. Statistical Methodology

6.1 Analysis Sets

The ITT analysis set is henceforth referred to as the Full Analysis Set (FAS) and defined as follows:

- Full Analysis Set (FAS): Participants who were randomized within RGB plus placebo participants from specified regimens, classified according to their randomized treatment assignment. Observations made after premature permanent discontinuation of study drug are included in this sample, should such participants remain on study. Observations completed after regimen data lock are excluded. Participants determined to not meet ALS diagnostic criteria are excluded.

The definition of the STF analysis set is revised as follows:

- Safety Full (STF) Set: Participants who initiated treatment within RGB plus placebo participants from specified regimens who are not known to be ineligible for RGB and who initiated treatment in their respective regimen, classified according to the treatment they actually received. Observations made after premature permanent discontinuation of study drug are included in this sample, should such participants remain on study. Observations completed after regimen data lock are excluded.

An analysis set restricting shared placebo participants to those regimens in which study drug is administered by the same route as RGB is defined as follows:

- Efficacy Common Mode of Administration (ECM) Set: The subset of participants in the FAS analysis set who are in regimens in which study drug is administered by the same route as RGB.

The definitions of the ECC, ERO, STN, and SRO analysis sets are the same as described in the M-SAP with reference to the ITT analysis set now referencing the FAS analysis set. The following analysis set is specific to RGB:

Efficacy Per-protocol (EPP) Set: The subset of participants in the FAS analysis set who initiated study treatment and who were not involved in protocol deviations that affected the scientific integrity of the trial as documented prior to data lock, classified according to the treatment they actually received. Inclusion or exclusion from the EPP analysis set of any participant for whom treatment assignment was unblinded prior to data lock will be governed by the prespecified criteria above. If a participant's data is truncated for inclusion in the EPP analysis set due to non-adherence to protocol-specified dosing, clinical events observed up to 28 days after the censoring event will be included in the EPP analysis set. For all other events leading to truncation of a participant's data, no events beyond that date will be included. Data from placebo participants shared from other regimens will not be truncated due to non-adherence to protocol-specified dosing.

-

Applicable analysis sets (FAS, ECM, EPP, STF, and STN) will include shared placebo participants from regimens A and C only. Data from shared placebo participants will include visits and events that occurred on or before the date of the final placebo-controlled period follow-up of a regimen A, B, or C participant. As only concurrently enrolling regimens are

contributing to efficacy analyses, the FAS and ECC analysis sets are synonymous and only the FAS analysis set will be referenced. As regimen A is administered by subcutaneous injection and regimen C is administered orally, only regimen C will contribute shared placebo participants for the ECM and STN analysis sets.

6.2 Baseline Characterization

The baseline characteristics summarized for participants randomized within RGB are the same as specified in the M-SAP with the addition of ALSAQ-40 domain scores and SI, CNS-BFS total score, King's stage, weight, body mass index (BMI), serum urate concentration, serum creatinine concentration, and serum NfL concentration.

6.3 Primary Efficacy Analysis and Supportive Analyses

The primary analysis for RGB is a Bayesian shared-parameter, repeated-measures model of ALSFRS-R that accounts for loss of follow-up due to mortality. Details of the model, including documentation of operating characteristics under a range of scenarios, are provided in the "ALS Master Protocol Recommended Statistical Analysis, Design and Simulation Report" (Appendix 1 to the Master Protocol). The Bayesian shared-parameter, repeated-measures model will be applied to the FAS analysis set as the primary analysis, to the ECM and ERO analysis sets as sensitivity analyses, and to the EPP analysis sets as a supportive analysis.

The estimand of the primary analysis is the relative rate of disease progression (the "disease rate ratio" or DRR) of active treatment relative to placebo in the FAS population under an assumption that active treatment slows mean time to death or death equivalent by the same proportion as treatment slows the mean rate of functional progression as measured by change in ALSFRS-R total score over time. The estimand is defined by the following attributes:

- Treatment: verdiperstat administered orally at a dosage of 600 mg BID vs. placebo.
- Population: FAS population as defined in Section 6.1.
- Variables: time to death or death equivalent and rate of change in ALSFRS-R total score from baseline to the Week 24 Visit.
- Intercurrent event 1: treatment discontinuation due to death: no ALSFRS-R data from participants who reach the death or death equivalent endpoint are included in the analysis, handled via mortality component in model, composite variable strategy approach.
- Intercurrent event 2: treatment discontinuation not due to death: handled via treatment policy approach, all data will be used including data collected during the placebo-controlled period after treatment discontinuation regardless of concomitant medication, for those participants who have not been censored due to mortality. Missing data post-treatment will not be imputed, handled via missing at random assumption.
- Population-level summary: mean ratio of hazard or progression rate of active treatment relative to placebo.

6.4 Interim Analysis

RGB will be considered for early stopping for futility according to the interim analysis schedule and definition specified in the "ALS Master Protocol Recommended Statistical Analysis, Design

and Simulation Report" (Appendix 1 to the Master Protocol). RGB will not be stopped early for success.

6.5 Secondary Efficacy Analyses

6.5.1 Hierarchical Testing

Primary inference for secondary efficacy endpoints will be based on analysis of the FAS analysis set using a repeated-measures linear mixed model for functional endpoints (see Section 6.5.2 below) and by Kaplan-Meier product-limit estimates and log-rank test for the primary survival endpoint (see Section 6.5.4 below). The sequence for testing secondary efficacy endpoints is the following:

1. HHD upper extremity percentage,
2. SVC,
3. HHD lower extremity percentage, and
4. Survival.

If the primary analysis indicates a significant slowing in disease progression from the Bayesian shared-parameter, repeated-measures model of ALSFRS-R and mortality, then each secondary efficacy endpoint in succession would be declared significant in the specified sequence using a comparison-wise criterion of two-tailed $p < 0.05$. After the first failure to declare significance, no endpoints lower in the hierarchy can be significant. This sequential closed-testing procedure controls the overall type 1 error rate at 5%. Nominal comparison-wise p-values for secondary efficacy endpoints will also be reported.

6.5.2 Repeated-measures Model

The specification of the repeated-measures linear mixed model and the primary linear contrast for estimating differences in 24-week change from baseline in a given continuous efficacy endpoint (ALSFRS-R total and domain scores, HHD upper extremity, lower extremity, and global average percentages, SVC, FVC by home spirometry, serum creatinine, serum NfL, ALSAQ-40 domain scores and SI, and CNS-BFS total score) are revised from those specified in the M-SAP to include a main effect of treatment.

The model will include fixed terms for discrete visit, treatment group, treatment group × visit interaction, centered time since symptom onset and centered time since symptom onset × visit interaction, centered delta-FRS and centered delta-FRS × visit interaction, centered baseline riluzole use and centered baseline riluzole × visit interaction, and centered baseline edaravone use and centered baseline edaravone × visit interaction. The following equations describe the model with regimen random effects:

$$\begin{gathered} Y_{ij} = a_{k(i)} + \gamma_1 t_i + \gamma_{2,j} v_j + \boldsymbol{\gamma}_3' \boldsymbol{z}_i + \gamma_{4,j}\, t_i v_j + \boldsymbol{\gamma}_{5,j}' \boldsymbol{z}_i\, v_j + \epsilon_{ij} \\ a_k \sim N(0, \sigma_r^2), \boldsymbol{\epsilon}_{i\cdot} \sim N(\boldsymbol{0}, \boldsymbol{R}), \mathrm{Cov}\left(b_{k(i)}, \epsilon_{ij}\right) = 0 \end{gathered} \quad \text{(eqn. 1)}$$

where Y_{ij} is a given efficacy endpoint measured in participant i at visit j, $a_{k(i)}$ is a random intercept for regimen k to which participant i was assigned, v_j is an indicator variable for visit j, $\boldsymbol{z}_i$ is the vector of covariates (centered time since onset, centered delta-FRS, centered baseline riluzole use, and centered baseline edaravone use) for participant i, t_i is an indicator variable for treatment t to which participant i was assigned, γ_1, $\gamma_{2,j}$, $\boldsymbol{\gamma}_3$, $\gamma_{4,j}$, and $\boldsymbol{\gamma}_{5,j}$ are estimated parameters

and vectors of parameters for the fixed effects, and ϵ_{ij} is the residual for participant *i* at visit *j*. The regimen-specific random effects are normally distributed with mean 0 and variance σ^2_r. The vector of residuals for a given participant are normally distributed with mean **0** and an unstructured covariance matrix **R**. The regimen-specific random effect for a given participant and residuals for that participant are uncorrelated.

The following SAS code specifies the model:

```
proc mixed data=xxx method=reml;
  class regimen id trtrnd visit;
  model Value = trtrnd|visit
              sx2bl|visit dFRS|visit rlz|visit edv|visit / solution cl;
  random intercept / subject=regimen type=vc;
  repeated visit / subject=id type=un;
```

where `id` is a participant study identifier, `trtrnd` is the randomly assigned treatment group, `visit` is the visit identifier, `Value` is value of the efficacy endpoint being tested for a given participant at a given visit, `sx2bl` is years since ALS symptom onset centered at the sample median, `dFRS` is pre-baseline slope centered at the sample median, `rlz` is an indicator of riluzole use at baseline, and `edv` is an indicator of edaravone use at baseline. The primary estimate will be the treatment-dependent difference in change from baseline to the Week 24 Visit. The estimate and its 95% Wald confidence bounds will be obtained by a linear contrast of adjusted means. The following SAS code specifies the linear contrast for a regimen with one active treatment assuming an endpoint measured every 8 weeks and that the sort order for treatment group has the active group last and visits are sorted chronologically:

```
  estimate "3|Act vs Plb|dWk 24" trtrnd*visit 1 0 0 -1  -1 0 0 1 / cl;
```

A significant difference in 24-week change from baseline in the direction of greater improvement or less worsening among participants randomized to active treatment would support inference of benefit from active treatment for the efficacy endpoint being tested.

The estimand estimated by the primary linear contrast of the repeated-measures linear mixed model is the mean difference in 24-week change from baseline of a given continuous efficacy endpoint in the active treatment group relative to the placebo group in the FAS population. The estimand is defined by the following attributes:

- Treatment: verdiperstat administered orally at a dosage of 600 mg BID vs. placebo.
- Population: FAS population as defined in Section 6.1.
- Variables: absolute change in endpoint from baseline to the Week 24 Visit.
- Intercurrent event: treatment discontinuation: handled via treatment policy approach, all data will be used including data collected during the placebo-controlled period after treatment discontinuation. Missing data post-treatment, including data missing due to death, will not be imputed, handled via missing at random assumption.
- Population-level summary: difference in conditional means of active treatment relative to placebo.

Inference from this analysis is supportive of inference from the Bayesian shared-parameter, repeated-measures model for the primary endpoint and is the primary analysis for secondary

endpoints. A separate supportive analysis will include centered baseline serum NfL level and the interaction of centered baseline serum NfL level and visit as additional covariates.

6.5.3 Random-slopes Model

The specification of the random-slopes linear mixed model and the primary linear contrast for estimating differences in mean rate of progression in a given continuous efficacy endpoint (ALSFRS-R total and domain scores, HHD upper extremity, lower extremity, and global average percentages, SVC, FVC by home spirometry, quantitative voice characteristics, serum creatinine, serum NfL, ALSAQ-40 domain scores and SI, and CNS-BFS total score) are revised from those specified in the M-SAP to include a main effect of treatment and to specify that study months are calculated as the difference in days from the Baseline Visit to the date of assessment of a given endpoint multiplied by 12 / 365.25.

The model will include fixed terms for month since the Baseline Visit, treatment group, treatment group × month interaction, centered years since ALS symptom onset and centered years since ALS symptom onset × month interaction, centered delta-FRS and centered delta-FRS × month interaction, centered baseline riluzole use and centered baseline riluzole use × month interaction, and centered baseline edaravone use and centered baseline edaravone use × month interaction. The following equations describe the model with regimen random effects:

$$\begin{gathered} Y_{ij} = \gamma_1 + a^0_{k(i)} + b^0_i + \gamma_2 t_i + \boldsymbol{\gamma}'_3 \boldsymbol{z_i} \\ + \left(\gamma_4 + a^1_{k(i)} + b^1_i + \gamma_5 t_i + \boldsymbol{\gamma}'_6 \boldsymbol{z_i}\right) m_{ij} + \epsilon_{ij} \\ \{a^0_k, a^1_k\} \sim N(\mathbf{0}, \boldsymbol{\Sigma_r}), \{b^0_k, b^1_k\} \sim N\left(\mathbf{0}, \boldsymbol{\Sigma_p}\right), \epsilon_{ij} \sim N(0, \sigma^2_\epsilon) \\ \text{Cov}(\boldsymbol{a}_k, \boldsymbol{b}_k) = \mathbf{0}, \text{Cov}(\boldsymbol{a}_k, \boldsymbol{\epsilon}_{i\cdot}) = \mathbf{0}, \text{and } \text{Cov}(\boldsymbol{b}_k, \boldsymbol{\epsilon}_{i\cdot}) = \mathbf{0} \end{gathered} \qquad \text{(eqn. 2)}$$

where Y_{ij} is a given efficacy endpoint measured in participant *i* at visit *j*, $a^0_{k(i)}$ and $a^1_{k(i)}$ are random intercept and slope for regimen *k* to which participant *i* was assigned, b^0_i and b^1_i are random intercept and slope for participant *i*, $\boldsymbol{z_i}$ is the vector of covariates (centered time since onset, centered delta-FRS, centered baseline riluzole use, and centered baseline edaravone use) for participant *i*, m_{ij} is the time from baseline to observation *j* for participant *i* in months calculated as days x 12 / 365.25, t_i is an indicator variable for treatment *t* to which participant *i* was assigned, γ_1, γ_2, $\boldsymbol{\gamma_3}$, γ_4, γ_5, and $\boldsymbol{\gamma_6}$ are estimated parameters and vectors of parameters for the fixed effects, and ϵ_{ij} is the residual for observation *j* for participant *i*. The regimen-specific random effects are normally distributed with mean **0** and unstructured covariance matrix $\boldsymbol{\Sigma_r}$. The participant-specific random effects are normally distributed with mean **0** and unstructured covariance matrix $\boldsymbol{\Sigma_p}$. The residuals for a given participant are normally distributed with mean 0 and variance σ^2_ϵ. The regimen-specific random effects, participant-specific random effects, and residuals are uncorrelated.

The following SAS code specifies the model:

```
proc mixed data=xxx method=reml;
  class regimen id trtrnd;
  model Value = trtrnd|month
              sx2bl|month dFRS|month rlz|month edv|month / solution cl;
  random intercept month / subject=regimen type=un;
  random intercept month / subject=id type=un;
```

where `month` is time in months from the Baseline Visit (assuming 12 months in an average of 365.25 days per year) and other fields are the same as identified above in Section 6.5.2. The primary estimand will be the treatment-dependent difference in slopes. The estimate and its 95% Wald confidence bounds will be obtained by a linear contrast of adjusted means. The following SAS code specifies the linear contrast for a regimen with one active treatment assuming that the sort order for treatment group has the active group last:

```
estimate "3|Act vs Plb|Slope (/mn)" month 0 trtrnd*month -1 1 / cl;
```

A significant difference in slopes in the direction of greater improvement or less worsening among participants randomized to active treatment would support inference of benefit from active treatment for the efficacy endpoint being tested.

The estimand estimated by the primary linear contrast of the random-slopes linear mixed model is the difference in mean rate of progression of a given continuous efficacy endpoint in the active treatment group relative to the placebo group in the FAS population. The estimand is defined by the following attributes:

Treatment: verdiperstat administered orally at a dosage of 600 mg BID vs. placebo.

Population: FAS population as defined in Section 6.1.

Variables: mean rate of change in endpoint from baseline to the Week 24 Visit.

Intercurrent event: treatment discontinuation: handled via treatment policy approach, all data will be used including data collected during the placebo-controlled period after treatment discontinuation. Missing data post-treatment will not be imputed, handled via missing at random assumption.

Population-level summary: difference in conditional mean slopes of active treatment relative to placebo.

Inference from these analyses is supportive of inference from the Bayesian shared-parameter, repeated-measures model for the primary endpoint and inference from the repeated-measures linear mixed model for secondary endpoints. A separate supportive analysis will include centered baseline serum NfL level and the interaction of centered baseline serum NfL level and study month as additional covariates.

6.5.4 Survival and Time to Clinical Events

Survival and time to hospitalizations and clinical events will be analyzed in the FAS, ECM, ERO, EPP, STF, SFN, and SRO analysis sets. Survival analyses that include follow-up beyond the placebo-controlled period will be analyzed in the ERO analysis set. The summaries and analyses of time to death or death equivalent are the same as specified in the M-SAP with the revision to include baseline age as an additional covariate in adjusted models, with the addition that the endpoints of time to death independent of occurrence of death equivalents and time to each of the hospitalization and clinical events will be separately analyzed using the same models, and with an additional adjusted analysis that includes baseline serum NfL level as a covariate. Analysis of time to King's stage 4a or 4b will accommodate interval censoring between ALSFRS-R assessments and will be stratified by baseline King's stage.

6.5.5 CAFS

The primary CAFS analysis is as specified in the MPRDR. Additional, unadjusted CAFS analyses are the same as specified in the M-SAP, including specification that pair-wise comparison of change in ALSFRS-R total score for participants who cannot be ranked by time to death or death equivalent is to the maximum follow-up time at which both participants have an observation, and with the following additions:

1. HHD upper and lower extremity percentage and SVC will be analyzed by CAFS by substituting change from baseline for those secondary efficacy endpoints in place of ALSFRS-R total score,
2. An additional set of CAFS analyses will use multiple imputation to extend follow-up of ALSFRS-R total score, HHD upper and lower extremity percentage and SVC for participants who early terminate, withdraw consent, or are lost to follow-up,
3. An additional set of CAFS analyses for ALSFRS-R total score, HHD upper and lower extremity percentage and SVC will use time to death alone independent of any death equivalent,
4. An additional set of CAFS analyses for ALSFRS-R total score, HHD upper and lower extremity percentage, and SVC will adjust rank scores in a linear model with the following covariates: time from ALS symptom onset, delta-FRS, baseline use of riluzole, and baseline use of edaravone, and
5. An additional set of CAFS analyses for ALSFRS-R total score, HHD upper and lower extremity percentage, and SVC will adjust rank scores in a linear model with the following covariates: time from ALS symptom onset, delta-FRS, baseline use of riluzole, baseline use of edaravone, and baseline serum NfL level.

The multiple imputation model used to extend follow-up of functional scores for participants who early terminate, withdraw consent, or are lost to follow-up will use linear regression with covariates of time since symptom onset, delta-FRS, baseline riluzole use, baseline edaravone use, and each observed functional score prior to a missing assessment.

Inference from CAFS analyses is supportive of inference from the Bayesian shared-parameter, repeated-measures model for the primary outcome and supportive of inference from the repeated-measures model for the secondary outcomes of HHD upper extremity score, SVC, and HHD lower extremity score. Primary inference from CAFS analyses will compare survival by time to death or death equivalent, will compare change in function to the last jointly observed time point, and will adjust for the specified covariates.

6.5.6 HHD0 and $HHD0^2$

Analyses of HHD0 are the same as specified in the M-SAP with the addition of parallel analyses of $HHD0^2$, with a separate analysis that includes baseline serum NfL level as an additional covariate, and with the clarification that time to zero strength for both analyses is interval censored between HHD assessments.

Inference from these analyses is supportive of inference from the repeated-measures linear mixed model for HHD upper and lower extremity scores.

6.5.7 Quantitative Voice Measures

Given the high frequency of voice recordings, a repeated-measures analysis with unstructured covariance is overly flexible but the assumption of linear change required by the random-slopes

model may be overly rigid. To complement estimates from the random-slopes linear mixed model, quantitative voice characteristics will be analyzed in a linear mixed model in which the temporal profile for both fixed and random terms is modeled using cubic B-splines with knots at 8 and 16 weeks. The model will include fixed terms for B-splines (4 terms), treatment group (2 levels), treatment group × B-spline interaction, centered time since symptom onset and centered time since symptom onset × B-spline interaction, centered delta-FRS and centered delta-FRS × B-spline interaction, centered baseline riluzole use and centered baseline riluzole × B-spline interaction, and centered baseline edaravone use and centered baseline edaravone × B-spline interaction The model will include random regimen-specific intercepts and slopes with unstructured covariance, random participant-specific B-splines (5 terms) with unstructured covariance, and a first-order autoregressive structure for residuals. A simplified covariance structure assuming no regimen-level covariance, heterogeneous compound symmetric covariance among the random B-splines, conditional independence of residuals, or a combination of the three simplifying assumptions will be used if the full model fails to converge. The primary estimand will be the treatment-dependent difference in 24-week change from baseline. The estimate and its 95% Wald confidence bounds will be obtained by a linear contrast of adjusted means. A separate analysis will include centered baseline serum NfL level and the interaction of centered baseline serum NfL level and B-splines as additional covariates.

6.5.8 Placebo Multiple Imputation

Placebo multiple imputation analyses are the same as specified in the M-SAP and will be applied to ALSFRS-R total score, HHD upper and lower extremity percentages, and SVC.

The following SAS code specifies the imputation for an endpoint measured every 8 weeks:

```
proc mi data=work.outcomes seed=xxx nimpute=50 out=work.out_mi01
        minimum=. . . . . x x x x maximum=. . . . . y y y y minmaxiter=1000;
  class rlz edv trtrnd;
  fcs reg(Wk08 = sx2bl dFRS rlz edv Wk00);
  fcs reg(Wk16 = sx2bl dFRS rlz edv Wk00 Wk08);
  fcs reg(Wk24 = sx2bl dFRS rlz edv Wk00 Wk08 Wk16);
  mnar model( Wk08 Wk16 Wk24 / modelobs=(trtrnd="0"));
  var sx2bl dFRS rlz edv Wk00 Wk08 Wk16 Wk24;
run;
```

where `Wk00`, `Wk08`, `Wk16`, and `Wk24` are the values of a given efficacy endpoint at the Baseline, Week 8, Week 16, and Week 24 Visits, respectively, `trtrnd` has a value of zero (0) for participants randomized to placebo, and `x` and `y` take appropriate values to specify the range of a given outcome measure (i.e., 0 and 48 for ALSFRS-R total score; 0 and . for HHD upper and lower extremity percentages and SVC).

Inference from these analyses is supportive of inference from the Bayesian shared-parameter, repeated-measures model for the primary endpoint and assess sensitivity to the missing data assumption of the repeated-measures linear mixed model for secondary endpoints in the FAS analysis set. A separate analysis will include centered baseline serum NfL level as an additional covariate in both imputation stages.

6.5.9 Additional Sensitivity Analyses of Primary and Key Secondary Outcomes

Sensitivity analyses of primary and key secondary efficacy outcomes are the same as specified in the M-SAP.

6.5.10 Subgroup Analyses

In addition to the subgroups specified in the M-SAP, the following additional subgroups will be analyzed in the random-slope model (see Section 6.5.3) for primary and secondary efficacy endpoints in the FAS analysis set:

- Baseline use of riluzole and edaravone (neither, riluzole only, edaravone only, both),
- Age (less than 65 years vs. 65 years or older),
- Sex (female vs. male),
- Race (white vs. any minority race with greater than 5% prevalence in the sample),
- Ethnicity (Hispanic or Latino vs. non-Hispanic or Latino),
- Weight (less than 70 kg, 70 to less than 85 kg, 85 kg or more),
- BMI (less than 18.5 kg/m^2, 18.5 to less than 25 kg/m^2, 25 kg/m^2 or more),
- Chronic kidney disease (CKD) stage (stage 1 or better [eGFR 90 mL/min/1.73m^2 or more], stage 2 [eGFR 60 to 89 mL/min/1.73m^2], stage 3 or worse [eGFR less than 60 mL/min/1.73m^2]),
- Time since onset of weakness (less than 18 months vs. 18 months or longer),
- Baseline serum NfL concentration (by median split), and
- Site (individual sites with at least 5 participants per treatment group and all participants from sites with fewer than 5 participants per treatment group pooled).

For each classification, unknown, not reported, and missing will be considered one group. All individuals not included in a specified subgroup will be combined into a mixed, "other" group. The "other" group will be included in analyses if its prevalence is greater than 5%; otherwise, the "other" group will be excluded.

In cases where a model for a given subgroup and endpoint fails to converge, the covariance terms for the regimen-specific random effects will be simplified from unstructured covariance of intercepts and slopes to separate, uncorrelated variance components for intercepts and slopes. If convergence still fails, regimen-specific intercepts and slopes will be modeled as fixed effects. If convergence still fails, the participant-specific random effects will be simplified from unstructured covariance of intercepts and slopes to separate, uncorrelated variance components for intercepts and slopes.

6.5.11 Comparison of Controls across Regimens

Comparisons of placebo participants across regimens are the same as specified in the M-SAP with separate analyses that include baseline serum NfL level as an additional covariate in adjusted analyses plus applicable interaction terms as relevant to a given model.

6.5.12 Pharmacokinetic Analyses

Pre-dose concentrations of verdiperstat in plasma will be summarized by treatment group and visit in the ERO sample. Concentrations below the limit of quantitation (BLQ) will be replaced with one half of the lower limit of quantitation. Summaries will include number of observations, number and percentage with concentrations BLQ, arithmetic mean, median, standard deviation, minimum, maximum, geometric mean, geometric coefficient of variation (calculated as sqrt(exp(variance of log-transformed concentrations) – 1)), and 95% confidence bounds for the geometric mean assuming log-normally distributed data.

Plasma concentration data of verdiperstat may be subjected to population pharmacokinetic analysis to derive population estimates of pharmacokinetic parameters and test the effect of various covariates such as age, weight, and sex. Details of the analysis will be described in a separate data analysis plan (DAP). This analysis may be performed by combining data from the current study with data from other studies of verdiperstat, if deemed appropriate. The population pharmacokinetic analysis will be performed by Biohaven and reported in a separate modelling report.

6.5.13 Pharmacodynamic Biomarker Analyses

Change in myeloperoxidase protein and activity levels in plasma will be summarized by treatment group and visit in the ERO sample. Summary statistics will be provided for the values, change from baseline, and percent change from baseline at each scheduled assessment time point. Myeloperoxidase protein and activity levels in plasma will be analyzed in a repeated-measures linear mixed model that includes fixed terms for visit, time from last dose of verdiperstat, and time from last meal.

6.6 Safety Analyses

6.6.1 Treatment-emergent Adverse Events

Summaries and analyses of treatment-emergent adverse events (TEAE) are the same as specified in the M-SAP with the following revisions.

TEAEs are defined as those adverse events with onset dates in the interval from double-blind treatment initiation to the earliest of the Final Safety Visit, the date the participant dies, early terminates, or is lost to follow-up, 28 days after last dose of study drug, or the date of first dose of study drug during participation in the OLE, if so exposed. Adverse events with onset on the day of double-blind treatment initiation and adverse events with incompletely specified onset date where the ambiguous date spans the day of double-blind treatment initiation or the earliest of the events above that define the end of the treatment-emergence interval will be assumed to be treatment emergent except for those known to precede first exposure to study drug.

In addition to summaries specified in the M-SAP, the following categories of adverse events will be summarized by Medical Dictionary for Regulatory Activities (MedDRA) system organ class and preferred term: (a) all adverse events, including those not classified as TEAEs, (b) fatal TEAEs, and (c) TEAEs that occurred during a participant's COVID-19 infection (defined as 5 days prior to symptom onset to end of COVID-19 symptoms or end of double-blind follow-up, if ongoing).

TEAEs indicating COVID-19 infection are the following: Asymptomatic COVID-19, COVID-19, COVID-19 pneumonia, COVID-19 treatment, Post-acute COVID-19 syndrome, SARS-CoV-

2 antibody test positive, SARS-CoV-2 RNA increased, SARS-CoV-2 sepsis, SARS-CoV-2 test false negative, SARS-CoV-2 test positive, SARS-CoV-2 viraemia, Suspected COVID-19.

Treatment-dependent differences in the proportion of participants experiencing a given type of TEAE will not be tested. Treatment-dependent differences in TEAE incidence rates in units of number per 100 participant years will be estimated as differences rather than ratios, will include comparison-wise 95% confidence intervals with variance estimates obtained by the delta method, and will be provided for overall classes of TEAEs not further classified by MedDRA term (all TEAEs, serious TEAEs, severe TEAEs, TEAEs leading to discontinuation of study drug, TEAEs resulting in death, and TEAEs of special interest) and TEAEs with at least 5% prevalence in the active arm.

Listings will document all adverse events (including those not classified as treatment emergent), TEAEs, serious TEAEs, severe TEAEs, related TEAEs, TEAEs leading to discontinuation of study drug, TEAEs resulting in death, and TEAEs of special interest.

6.6.2 Safety Labs

Summaries and analyses of clinical safety labs are the same as specified in the M-SAP with the revision that lab results collected more than 28 days after last dose of study drug will not be tabulated, that abnormal levels will be classified to a toxicity grade based on quantitative grading using National Cancer Institute (NCI) Common Terminology Criteria for Adverse Events (CTCAE) version 5.0, and with the addition that maximum toxicity over all post-baseline visits that occur within 28 days or fewer after last dose of study drug will be included in shift tables along with visit-specific shifts.

The proportion of participants with TSH ≥10 mIU/L and the proportion with signs or symptoms of hypothyroidism will be presented as shift tables vs. the status of each participant at baseline for each visit and over all post-baseline visits that occur within 28 days or fewer after last dose of study drug by treatment group in all safety samples. The absolute level and the absolute change from baseline for free T3, free T4, and TSH will be summarized as means, standard deviations, medians, and ranges at each visit by treatment group in all safety samples.

6.6.3 ECG Results

Summaries of ECG parameters and findings are the same as specified in the M-SAP with the revision that ECG parameters and findings collected more than 28 days after last dose of study drug will not be tabulated.

6.6.4 Vital Signs and Weight

Summaries and analyses of vital signs and weight are the same as specified in the M-SAP with the revision that vital signs and weight collected more than 28 days after last dose of study drug will not be tabulated.

6.6.5 Suicidality

Summaries of suicidality are the same as specified in the M-SAP with the revision that suicidality noted more than 28 days after last dose of study drug will not be tabulated.

6.7 Other Analyses

6.7.1 Participant Disposition

All participants consented to the Master Protocol between the time of the first and last consent of a participant assigned to a regimen included in the FAS analysis set will be summarized as a single set for the following events: consented to the Master Protocol, failed screening for the Master Protocol, other reasons not assigned to a regimen (including timing out of the screening window, death, withdrawal of consent, early termination, and administrative termination), and assigned to a regimen. Reasons for Master Protocol screen failure will be summarized.

All participants in the above sample assigned to a regimen will be summarized as two sets (final screening for RGB vs. final screening for a non-RGB regimen) for the following events: consented to a regimen, failed screening for a regimen, other reasons not randomized within a regimen (including timing out of the screening window, death, withdrawal of consent, early termination, and administrative termination), and randomized within a regimen. If a given individual is screened multiple times prior to randomization within a regimen, then the final screening experience of that individual will be summarized. Reasons for RGB screen failure will be summarized separately for all participants screened for RGB whether that was their final screening experience or not.

All participants in the FAS analysis set will be summarized as two sets (randomization to active study drug vs. randomization to placebo) for the following events: initiated regimen-specific study drug, prematurely terminated study participation due to death, withdrawal of consent, early termination, loss to follow-up, or administrative termination, completed 24-week follow up, and completed a safety follow-up visit vs. continued into the OLE. Reasons for withdrawal of consent or early termination after randomization will be summarized.

Any randomized participants excluded from the FAS and EPP analysis sets or included in the FAS analysis set but not contributing to the primary analysis will be identified in a listing together with the reason for their exclusion.

6.7.2 Study Drug Compliance and Tolerance

Summaries of study drug compliance and tolerance are the same as specified in the M-SAP with the clarification that summaries will be reported for the ERO and SRO analysis sets and that date of permanent discontinuation of study drug is the date of last use of double-blind study drug among all participants in a given analysis set.

The number of days of exposure to study drug will be calculated in three ways:

- as the number of days from dose initiation to the final safety assessment during the placebo-controlled period, inclusive,
- as the number of days from dose initiation to drug withdrawal, inclusive, less any interval during which use of study drug was interrupted (individual missed doses will not be subtracted unless noted in the dosage management log), and
- as the number of days from dose initiation to the earlier of final contact during the placebo-controlled period or 28 days after last dose of study drug, inclusive.

The proportion of participants who interrupted study drug or reduced study drug dosage and the time to first study drug interruption or dosage reduction will be summarized. The number of days of exposure to a reduced dosage of study drug will be summarized.

6.7.3 Concomitant Medication Use

Summaries of concomitant medication use are the same as specified in the M-SAP with the clarification that medications taken at baseline and those initiated after first dose of study drug will be separately summarized and will be classified by ATC Therapeutic class and WHODrug Preferred base name.

6.7.4 Medical History

Medical histories will be summarized by MedDRA system organ class, high level term, and preferred term in the STF and SRO analysis sets.

6.7.5 Blindedness

The proportions of participants and site investigators who report on the Exit Questionnaire a guess of active vs. placebo treatment assignment, each level of surety of that guess, and each of five pre-specified reasons for making a treatment assignment will be summarized by treatment group in the FAS and ERO analysis sets. Treatment-dependent differences in the proportion guessing active treatment assignment will be tested among all respondents and among those stating they are at least somewhat sure of their guess by Fisher's exact test and the difference in proportion guessing active treatment assignment will be estimated with confidence bounds.

6.7.6 Protocol Deviations

The number of major and minor protocol deviations will be summarized by type of deviation and treatment group in all analysis sets. Listings of all protocol deviations will be produced.

6.7.7 Impact of COVID-19 Pandemic

The proportions of planned assessments missed due to COVID-19 restrictions or disruptions will be summarized by treatment group, visit, and type of assessment in the FAS and ERO analysis sets. Protocol deviations that resulted from COVID-19 restrictions or disruptions will be summarized by treatment group and type of deviation in the FAS and ERO analysis sets.

A listing will document participants with assessments missed due to COVID-19 restrictions or disruptions and those experiencing protocol deviations that resulted from COVID-19 restrictions or disruptions and will describe the manner affected by COVID-19.

7. Validation

7.1 Primary Efficacy Analysis

Validation of the primary efficacy analysis is the same as specified in the M-SAP.

7.2 Secondary, Exploratory, and Safety Analyses

Validation of secondary, exploratory, and safety analyses are the same as specified in the M-SAP.

8. References

The following references are cited in addition to those specified in the M-SAP:

Balendra R, Jones A, Jivraj N, Knights C, Ellis CM, Burman R, Turner MR, Leigh PN, Shaw CE, Al-Chalabi A. Estimating clinical stage of amyotrophic lateral sclerosis from the ALS Functional Rating Scale. Amyotroph Lateral Scler Frontotemporal Degener. 2014 Jun;15(3-4):279-84.

Roche JC, Rojas-Garcia R, Scott KM, Scotton W, Ellis CE, Burman R, Wijesekera L, Turner MR, Leigh PN, Shaw CE, Al-Chalabi A. A proposed staging system for amyotrophic lateral sclerosis. Brain. 2012 Mar;135(Pt 3):847-52.
