# Supplementary material for: Verdiperstat in Amyotrophic Lateral Sclerosis: Results From the Randomized HEALEY ALS Platform Trial
Source: JAMA Neurol. 2025 Feb 17;82(4):333–43. doi: 10.1001/jamaneurol.2024.5249 (PMC11833655; doi:10.1001/jamaneurol.2024.5249)
Supplement: Supplement 3. — eMethods. eTable 1. ALSFRS-R by Visit eTable 2. Summary of Treatment-Emergent Adverse Events [file jamaneurol-e245249-s003.pdf]

Supplementary Online Content

Writing Committee for the HEALEY ALS Platform Trial; HEALY ALS Platform Trial Study Group. Verdiperstat in amyotrophic lateral sclerosis: results from the randomized HEALEY ALS Platform Trial. *JAMA Neurol*. Published online February 17, 2025. doi:10.1001/jamaneurol.2024.5249

eMethods.

eTable 1. ALSFRS-R by Visit

eTable 2. Summary of Treatment-Emergent Adverse Events

This supplementary material has been provided by the authors to give readers additional information about their work.

© 2025 Writing Committee for the HEALEY ALS Platform Trial. *JAMA Neurology.*

eMethods

Trial Design and Oversight

The HEALEY ALS Platform Trial is a novel, efficient, perpetual design, platform trial designed to test multiple investigational products against placebo in parallel. In this platform trial, each new investigational product is tested as a regimen specific appendix, or "Regimen" to the master protocol. Verdiperstat was evaluated as Regimen B of the HEALEY ALS Platform Trial. The trial was conducted in accordance with Good Clinical Practice guidelines of the International Conference on Harmonization and ethical principles of the Declaration of Helsinki. Protocol approval was provided for all trial sites by a central Institutional Review Board, the Massachusetts General Brigham Human Research Committee. All participants provided written informed consent prior to screening.

Trial Participants

Eligibility criteria for the platform trial included adults with a diagnosis of clinically possible, probable, laboratory-supported probable, or definite ALS defined by the revised El Escorial criteria. Key inclusion criteria were a disease duration ≤ 36 months, a vital capacity ≥ 50% predicted for age, height, and sex, the ability to swallow pills and liquids, and either no use of riluzole and/or edaravone or both or stable dosing of riluzole and/or edaravone for more than 30 days or one cycle respectively. The verdiperstat regimen was conducted prior to the FDA approval of sodium phenylbutyrate /taurorsodiol. Exclusion criteria for the verdiperstat regimen included concomitant chronic/long-term use (defined as more than two weeks use) of strong inhibitors of CYP1A2 or CYP3A4 or regular consumption of more than 8 oz per day of grapefruit juice on a regular basis. Full inclusion and exclusion criteria can be viewed in the trial protocol (Master Protocol - Supplement 2, Regimen Specific Appendix - Supplement 3).

Randomization techniques

Both the randomization to regimen, which was not blinded, and the randomization to active drug or placebo within a regimen, which was blinded to both participant and investigator, were done using as pre-determined randomization

© 2025 Writing Committee for the HEALEY ALS Platform Trial. *JAMA Neurology.*

schedule. In order to ensure balance in regimens and treatment groups by current medication use, both randomization schedules were stratified by baseline use of riluzole and edaravone (4 strata total).

Random assignment to a regimen was done. Random assignment to a regimen within a stratum uses a block urn design (Zhao and Wang; Block urn design - a new randomization algorithm for sequential trials with two or more treatments and balanced or unbalanced allocation. Contemp Clin Trials. 2011 Nov;32(6):953-61) with one instance of each regimen in a given block and modified to remove from consideration any regimens to which the participant cannot be assigned (termed "unavailable regimens").

Within each strata a blocking schema was used to create the randomization schedule. Randomization schedules were created using SAS software and validated to ensure no discernable patterns were seen before implementation.

Trial Interventions and Procedures

Eligible participants were randomized in a 3:1 ratio to receive oral verdiperstat 600 mg BID or matching placebo within strata of edaravone and riluzole use for a planned placebo-controlled duration of 24 weeks. Participants who were determined eligible after Master Protocol screening were randomly assigned with equal probability to all available regimens active at the participant's site and to which the participant had not previously been determined in eligible or previously randomized within. Participants were stratified into four strata as follows: (1) no use of riluzole or edaravone, (2) use of riluzole alone, (3) use of edaravone alone, (4) use of both riluzole and edaravone. Clinic or phone visits were conducted at baseline and every four weeks thereafter through week 24, with a final phone follow-up at week 28. Participants who completed the placebo-controlled trial were eligible to enroll in an open-label extension trial evaluating the long-term effects of verdiperstat. The open-label extension was terminated in September 2022 following the announcement of the top line results.

Outcomes

Primary and Secondary Efficacy Outcomes:

The primary efficacy outcome was the change from baseline through week 24 in disease severity, as measured by a joint model of ALS Functional Rating Scale- Revised (ALSFRS-R), and survival outcomes. The ALSFRS-R is a 12-item instrument that assesses four subdomains of bodily function (i.e., bulbar, fine motor, gross motor, and breathing),

© 2025 Writing Committee for the HEALEY ALS Platform Trial. *JAMA Neurology.*

with each item being scored on an ordinal scale (0 = total loss of function, 4 = no loss of function, maximum 48, lower scores indicating greater functional difficulty). The scale, validated for administration in person or by telephone, has shown high inter- and intra-rater reliability.

Secondary clinical efficacy outcomes (in hierarchical order) were analyzed in the FAS dataset and included 24-week change in isometric muscle strength as measured by Hand-Held Dynamometry (HHD) of upper limb muscles and grip strength; 24-week change in slow vital capacity (SVC), Hand-Held Dynamometry (HHD) of lower limb muscles; and time to death or death-equivalent events (tracheostomy or permanent assisted ventilation more than 22 hours daily for more than 7 consecutive days). Isometric muscle strength of seven pairs of upper extremity and four pairs of lower extremity muscle groups was assessed using HHD, with at least two and up to three trials of each muscle group. In addition, bilateral hand grip strength was assessed and was included in the upper extremity muscle strength endpoint. Respiratory muscle function was assessed by SVC, measured in an upright position for at least three trials per assessment or for up to five trials when the highest and second highest of the first three measurements differed by 10% or more. SVC volumes were standardized to percent predicted based on normal values calculated on age, sex, and height. The highest recorded SVC score from all attempts was utilized for analysis.

Safety and Tolerability Outcomes:

Safety was assessed via documentation of treatment-emergent adverse events (TEAEs) at each trial visit. Symptoms of ALS disease progression were also recorded as TEAEs. Any worsening of a disease progression measure that was being recorded and analyzed separately (i.e., ALSFRS-R, HHD, and SVC) was not recorded as a TEAE. Hypothyroidism, an adverse event of special interest due to the possibility of off-target thyroid peroxidase (TPO) inhibition (due to the similarity of TPO to MPO), was assessed as follows: proportion of participants with thyroid-stimulating hormone (TSH) ≥10 mIU/L, proportion of participants with signs or symptoms of hypothyroidism, and abnormal levels of free T3, free T4, and TSH. Based on prior trials, a small percentage of participants was expected to develop mild (subclinical) hypothyroidism generally with serum TSH < 10 mIU/L. The American Thyroid Association and American Association of Clinical Endocrinology use TSH ≥10mIU/L to distinguish between overt hypothyroidism versus subclinical hypothyroidism and recommend starting levothyroxine therapy at or above this level. Safety was additionally assessed using laboratory assays, electrocardiograms, suicidality and changes in vital

© 2025 Writing Committee for the HEALEY ALS Platform Trial. *JAMA Neurology.*

signs described in more detail in the trial protocol (Master Protocol - Supplement 2, Regimen Specific Appendix - Supplement 3).

An exit questionnaire was administered at the final trial visit (week 24 or at early discontinuation) to evaluate the blinding of participants and investigators to treatment allocation by asking whether they thought the participant was on active treatment or placebo.

A hierarchy was prepared for secondary outcomes for inference testing, available in the Statistical Analysis Plan (Master SAP- Supplement 2, Regimen Specific SAP, Supplement 3).

Analysis Populations

The primary analysis population, referred to as the Full Analysis Set (FAS) included all participants randomized to the active treatment arm in the verdiperstat regimen, all participants randomized to the placebo arm within the verdiperstat regimen, and placebo participants from contributing regimens, referred to as shared placebo. Observations completed after regimen database lock and participants determined to not meet ALS diagnostic criteria were excluded. Observations made after premature permanent discontinuation of the study drug were included in this dataset, should such participants have remained in the study. Additional prespecified exploratory efficacy analyses were performed in the (a) efficacy regimen-only population, which limits placebos to only those randomized with the verdiperstat regimen and (b) subset of participants in the FAS analysis set who are in regimens in which study drug is administered by the same route as the verdiperstat regimen. Safety analyses included all active arm participants in the verdiperstat regimen who initiated treatment and all placebo participants from the verdiperstat regimen and contributing regimens who initiated treatment.

Primary analysis methods

The primary analysis is a Bayesian shared parameter model of function and survival that provides an integrated estimate of the increase or decrease in the rate of disease progression on treatment relative to control. The analysis model has components for each and rationale for analyses of functional change among survivors as previous published. The treatment effect was quantified by the disease rate ratio (DRR). The disease rate ratio (DRR) represents the

© 2025 Writing Committee for the HEALEY ALS Platform Trial. *JAMA Neurology.*

slowing in the rate of decline of ALSFRS-R and the rate of mortality of the treated group, relative to placebo group. For example: DRR of 0.75 corresponds to 25% slowing in the rate of decline in ALSFRS-R and time to mortality. A DRR of 1 corresponds to no difference between treatments. Values of DRR less than 1 indicate a slowing in disease progression on treatment relative to control. In the functional component, ALSFRS-R data were analyzed from those who had survived using a linear repeated measure model that adjusts for baseline covariates and accommodates potential differences in the shared control across regimens. The survival component is estimated through an exponential proportional hazards model. The shared treatment effect between the ALSFRS-R and mortality components allows the participants who were lost to follow-up due to mortality to inform treatment effect estimates beyond their censored ALSFRS-R longitudinal data. The degree to which treatment effects on mortality inform the shared-treatment effect parameter depends on the mortality rate within the study. The primary analysis includes concurrent shared controls across regimens. It is expected that there will not be systematic differences in rates of progression in controls across regimens. The platform enrols participants under the same master protocol, across the same sites, during the same time period, and with only very minor differences in regimen-specific inclusion/exclusion. However, minor differences across the shared controls may be present due to these minor differences in inclusion/exclusion and due to differences in the modes of administration. To account for these potential differences, the primary analysis is conducted within a Bayesian hierarchical meta-analytic framework. As such, the regimen-specific random effects on the rate of progression for controls are assumed to come from an underlying hierarchical distribution with unknown population mean and variability. The population mean and variability (hyper-parameters) within the hierarchical distribution are given a prior distribution, and posterior estimates are obtained from the data. If the variability in the rate of progression is estimated to be small, the model will borrow more information across regimens and the analysis will be similar to a pooled analysis across regimens. If the variability in the rate of progression is estimated to be large across regimens, the model will borrow less across regimens and the analysis will be similar to an independent analysis across regimens.

The Bayesian posterior distribution of all model parameters is calculated using Markov chain Monte Carlo (MCMC). We drew at least 500,000 samples from the posterior distribution with a thinning of 10. The exact number of samples from the MCMC was determined based on convergence diagnostics to ensure an adequate effective sample size of the posterior distribution.

© 2025 Writing Committee for the HEALEY ALS Platform Trial. *JAMA Neurology.*

Prespecified sensitivity analyses, including combined analysis of function and survival (CAFS), which was employed as a sensitivity analysis, were performed to assess the sensitivity of the results to the primary analysis population, modeling assumptions, and missing data assumptions as described in Statistical Analysis Plan (Master SAP-Supplement 2, Regimen Specific SAP, Supplement 3).

Pre-specified interim analyses were conducted within the master protocol with the potential for a regimen to stop early for futility. Interim analyses evaluating futility were conducted every 12 weeks for all regimens with at least 40 participants (30 active and 10 control) with 24 weeks of follow-up or earlier death or termination. Interim analyses occurred simultaneously for all actively enrolling regimens with sufficient data. Non-binding futility was to be declared if the posterior probability that verdiperstat slowed disease progression by at least 10% was less than 5%. Stopping due to early success was not considered. The trial did not meet the cut-off for early futility and was completed as planned.

Analyses were performed using SAS (version 9.4, SAS Institute, Cary, NC), R (v4.1.2), and JAGS (v 4.2.0). For the final primary analysis, a one-tailed posterior probability of DRR < 1 greater than 0.979 was prespecified for declaring significance to control type 1 error rate based on simulations.

Secondary efficacy analysis methods

For continuous outcomes such as HHD and SVC, a linear mixed effects model with a fixed terms for months since baseline, treatment group, centered time symptom onset, pre-baseline slope, baseline edaravone, and riluzole use and their interaction with time was used. For the voice characteristics, a more flexible linear mixed model using cubic B-splines, with knots at 8 and 16 weeks, in both fixed and random terms accommodated ad-hoc at-home assessments and non-linear temporal change.

Primary inference for secondary efficacy endpoints was based on analysis of the FAS analysis set using a repeated-measures linear mixed model for functional endpoints and by Kaplan-Meier product-limit estimates and log-rank test for the primary survival endpoint. The sequence for testing secondary efficacy endpoints was in the following descending order of importance: HHD upper extremity percentage, SVC, HHD lower extremity percentage and survival. If the primary analysis indicated a significant slowing in disease progression from the Bayesian shared-

© 2025 Writing Committee for the HEALEY ALS Platform Trial. *JAMA Neurology.*

parameter, repeated-measures model of ALSFRS-R and mortality, then each secondary efficacy endpoint in succession was planned to be declared significant in the specified sequence using a comparison-wise criterion of two-tailed $p < 0.05$. After the first failure to declare significance, no endpoints lower in the hierarchy would be deemed significant. This sequential closed-testing procedure controlled the overall type 1 error rate at 5%. Each participant's vital status was accounted for at the end of the trial.

© 2025 Writing Committee for the HEALEY ALS Platform Trial. *JAMA Neurology.*

eTables

eTable 1. ALSFRS-R by visit

Visit	Verdiperstat		Shared Placebo		Regimen Placebo	
	N	mean (SD)	n	mean (SD)	n	mean (SD)
Baseline	126	34.3 (6.5)	122	35.2 (6.6)	41	34.3 (6.9)
Week 4	122	33.1 (7.1)	121	34.4 (7.2)	41	33.5 (6.9)
Week 8	117	32.0 (7.6)	117	33.4 (7.6)	40	32.5 (7.6)
Week 12	111	31.1 (8.0)	115	32.6 (8.1)	38	31.7 (7.4)
Week 16	103	30.0 (8.4)	110	31.8 (8.1)	38	30.7 (8.1)
Week 20	100	29.6 (8.5)	107	31.4 (8.6)	37	29.6 (8.8)
Week 24	99	28.5 (9.3)	105	30.2 (8.7)	35	28.9 (8.6)

© 2025 Writing Committee for the HEALEY ALS Platform Trial. *JAMA Neurology.*

eTable 2. Summary of Treatment-Emergent Adverse Events

Treatment Emergent Adverse Events (TEAE)	Verdiperstat (N=126)	Shared placebo (N=122)	Regimen-specific Placebo (N=41)
**Any TEAE**	114 (90.5%), 753	112 (91.8%), 649	37 (90.2%), 204
**Mild**	32 (25.4%), 478	48 (39.3%), 464	17 (41.5%), 156
**Moderate**	55 (43.7%), 233	42 (34.4%), 144	15 (36.6%), 43
**Severe**	27 (21.4%), 42	22 (18.0%), 41	5 (12.2%), 5
**Serious TEAEs**	17 (13.5%), 20	12 (9.8%), 18	2 (4.9%), 2
**Treatment-Related Serious TEAE**	2 (1.6%), 2	2 (1.6%), 3	1 (2.4%), 1
**Deaths**	4 (3.2%), 4	4 (3.3%), 4	0 (0.0%), 0
**TEAEs Leading to Study Drug Withdrawal**	21 (16.7%), 37	8 (6.6%), 14	3 (7.3%), 7
**TEAEs Leading to Study Drug Interruption**	14 (11.1%), 24	11 (9.0%), 23	2 (4.9%), 3
**TEAEs Leading to Study Drug Dose Reduction**	14 (11.1%), 24	3 (2.5%), 6	2 (4.9%), 5
**TEAEs with ≥5% incidence in either group**			
**MedDRA SOC Preferred Term**		**Incidence — no. of participants (%), No. of events**	
Nausea	35 (27.8%), 40	12 (9.8%), 14	3 (7.3%), 3
Headache	27 (21.4%), 31	17 (13.9%), 21	9 (22.0%), 11
Muscular weakness	22 (17.5%), 35	32 (26.2%), 46	12 (29.3%), 18
Fall	24 (19.0%), 50	31 (25.4%), 51	9 (22.0%), 16
Constipation	24 (19.0%), 26	14 (11.5%), 17	8 (19.5%), 8
Fatigue	20 (15.9%), 21	25 (20.5%), 28	11 (26.8%), 14

© 2025 Writing Committee for the HEALEY ALS Platform Trial. *JAMA Neurology.*

Insomnia	25 (19.8%), 28	2 (1.6%), 2	0 (0.0%), 0
Neuromyopathy	16 (12.7%), 22	19 (15.6%), 30	8 (19.5%), 9
Tension headache	13 (10.3%), 17	9 (7.4%), 14	3 (7.3%), 7
Dysphagia	12 (9.5%), 13	13 (10.7%), 16	4 (9.8%), 6
Dizziness	13 (10.3%), 15	7 (5.7%), 9	3 (7.3%), 3
Decreased appetite	13 (10.3%), 13	10 (8.2%), 11	2 (4.9%), 2
Urinary tract infection	10 (7.9%), 11	10 (8.2%), 10	4 (9.8%), 4
Dyspnoea	10 (7.9%), 11	8 (6.6%), 9	2 (4.9%), 2
Salivary hypersecretion	10 (7.9%), 10	7 (5.7%), 7	2 (4.9%), 2
Diarrhoea	10 (7.9%), 12	8 (6.6%), 9	1 (2.4%), 1
Blood thyroid stimulating hormone increased	11 (8.7%), 12	1 (0.8%), 1	0 (0.0%), 0
Oedema peripheral	7 (5.6%), 8	9 (7.4%), 11	4 (9.8%), 4
Anxiety	9 (7.1%), 9	4 (3.3%), 4	1 (2.4%), 1
Back pain	7 (5.6%), 9	2 (1.6%), 8	2 (4.9%), 8
Arthralgia	7 (5.6%), 11	6 (4.9%), 6	1 (2.4%), 1
Cough	7 (5.6%), 7	3 (2.5%), 3	1 (2.4%), 1
Urine odour abnormal	8 (6.3%), 8	0 (0.0%), 0	0 (0.0%), 0
Dysarthria	4 (3.2%), 4	7 (5.7%), 8	3 (7.3%), 4
Pain in extremity	4 (3.2%), 4	0 (0.0%), 0	3 (7.3%), 4

* presented as number of participants, (%), number of events

© 2025 Writing Committee for the HEALEY ALS Platform Trial. *JAMA Neurology.*
